# *One-pot* dearomatizative telescoped addition of *C*-nucleophiles to fluorinated 1,2,4-oxadiazoles followed by regioselective *N*-functionalization

**DOI:** 10.1039/d5qo01707f

**Published:** 2026-01-09

**Authors:** Davide Castiglione, Sara Amata, Federica Lauria, Andrea Maranzana, Salvatore Baldino, Alexander Prado-Roller, Laura Castoldi, Antonio Palumbo Piccionello, Vittorio Pace, Eisuke I. Comas Iwasita

**Affiliations:** a University of Turin, Department of Chemistry Via Giuria 7 10125 Turin Italy; b University of Palermo, Department of Biological, Chemical and Pharmaceutical Sciences and Technologies Viale delle Scienze Ed. 17 90128 Palermo Italy antonio.palumbopiccionello@unipa.it; c University of Vienna, Department of Inorganic Chemistry, Functional Materials Waehringerstrasse 42 1090 Vienna Austria; d University of Milan, Department of Pharmaceutical Sciences, General and Organic Chemisty Section “A. Marchesini” Via Venezian 21 20133 Milan Italy; e University of Rome “La Sapienza”, Department of Chemistry P.le A. Moro 5 00185 Rome Italy vittorio.pace@uniroma1.it; f University of Vienna, Department of Pharmaceutical Sciences Josef-Holaubek-Platz 2 1090 Vienna Austria vittorio.pace@univie.ac.at

## Abstract

The constitutive low aromaticity of easily accessible 5-trifluoromethyl-1,2,4-oxadiazoles is explored to enable editing modification to the corresponding unprecedented *gem*-disubstituted 1,2,4-oxadiazolines. The operation proceeds *via* the nucleophilic addition of diverse carbon-centered nucleophiles with excellent regiocontrol (in almost all cases), thus selectively furnishing either the 2,5-dihydro or 4,5-dihydro isomers. The process, which also exhibits high chemocontrol, enables further derivatization of the intermediate anion with externally added electrophilic platforms. Calculations support the experimental evidence and identify intrinsic steric properties of the nucleophiles as a key factor controlling regioselectivity, thus rationalizing the non-optimal outcome observed in particular circumstances (*i.e.* LiCH_2_Br).

## Abstract 2

The constitutive low aromaticity of easily accessible 5-trifluoromethyl-1,2,4-oxadiazoles is explored to enable editing modification to the corresponding unprecedented *gem*-disubstituted 1,2,4-oxadiazolines.

## Graphical abstract

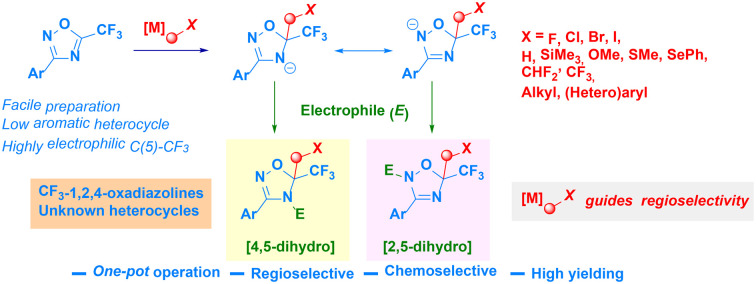

## Introduction

Dihydro-1,2,4-oxadiazoles (*i.e.* 1,2,4-oxadiazolines) constitute an important class of *non*-aromatic five-membered heterocycles that demonstrate significant adaptability in medicinal chemistry.^1^ In particular, these motifs are found in biologically active substances, including anticancer,^2^ anti-diabetic,^3^ anti-Alzheimer,^4^ kinase-inhibitory^5^ and nicotinic receptor-antagonist agents.^6^ In synthetic methodology, 1,2,4-oxadiazolines are suited for heterocyclic skeletal modifications, leading to imidazoles,^7^ isoxazoles^8^ and pyrroles.^9^ First introduced by Tiemann in 1889,^10^ the cyclocondensative approach–between amidoximes and carbonyls-continues to represent the canonical logic for assembling dihydro-1,2,4-oxadiazoles (Scheme 1.1).^11^ Though significant progresses have been documented, the relatively limited regiocontrol and substrate specificity (*i.e.* lack of generality) continue to plague this conceptually straightforward technique.^12^ Unfortunately, regioselectivity issues are pervasive and eclipse the potential of generating the heterocycle through [3 + 2]- or [1,3]-cycloadditive strategies involving nitrile oxides and imines (Scheme 1.2a).^13^ Although 4,5-dihydro-1,2,4-oxadiazole analogues are usually the predominant products, the switching to the combination of nitrones and nitriles could furnish 2,5-dihydro isomers in specific circumstances (Scheme 1.2b).^14^ However, the development of methodologies with high selectivity to the latter heterocyclic arrangement (*i.e.* 2,5-dihydro) is extremely rare and has practical limitations. Xuan and Xiao demonstrated the effectiveness of a visible-light-promoted [3 + 2] cycloaddition between nitroso-arenes and 2*H*-azirines (Scheme 1.3a).^15^ In this case, the difficulties in the access and manipulation of both precursors were successfully overcome *via* the Li–Walsh strategy by relying on a transition-metal-free SET activation of less problematic (and common organic feedstocks) nitroarenes and imines (Scheme 1.3b).^13*f*,16^ Accordingly, under basic conditions, the generation of 2-azaallyl anion enables electron transfer to the nitro group, thus reducing it to the corresponding nitroso analogue. Concomitantly, this SET pathway produces the 2-azaallyl radical, which couples with the nitroso species to deliver 1,2,4-oxadiazolines.

**Scheme 1:**
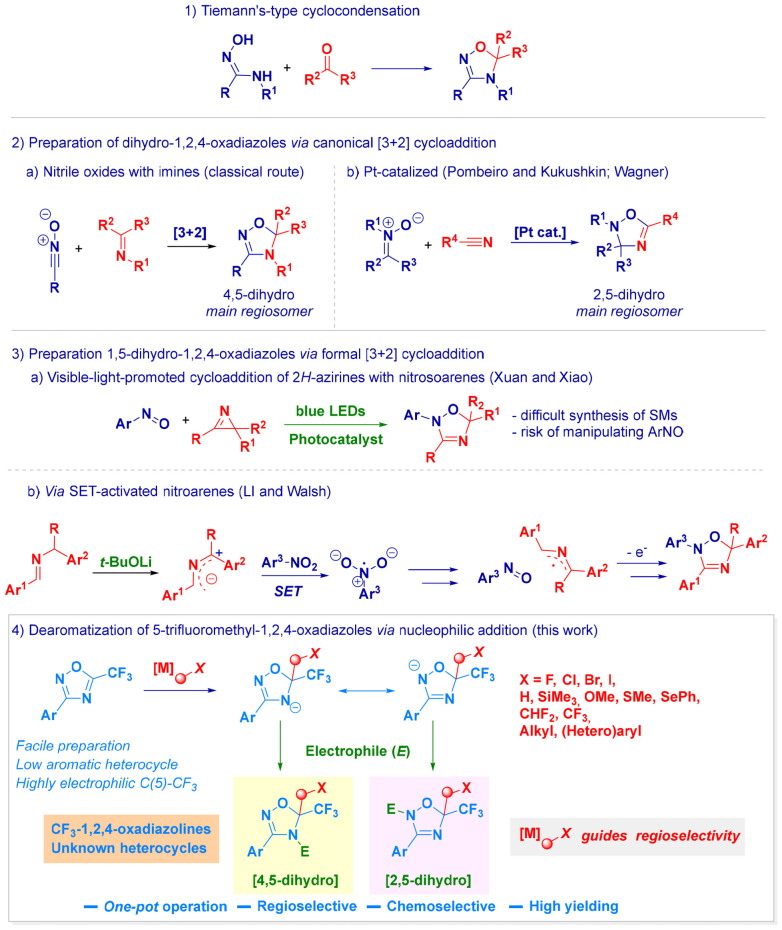
General context of the presented work.

A conceptually distinct approach would rely on the regioselective editing of an easily accessible, preformed heterocycle (Scheme 1.4). The grounding elements underpinning the reactivity of 1,2,4-oxadiazoles assign a prominent electrophilic behaviour to the C5 carbon, which would be implemented by the simultaneous installation of an adequate electron-withdrawing functionality. As recently demonstrated by our group for imine surrogates,^17^ the trifluoromethyl (CF_3_) group is particularly suited for this purpose and, if successful, would enable exploration of the unknown chemical space of trifluoromethyl-1,2,4-oxa*diazolines*. We would foresee unique properties of this new class of heterocycles as a consequence of the modulation imparted by the trifluoromethyl group in terms of physical–chemical parameters.^18^ The following considerations are pertinent: (a) the effectiveness of the strategy would depend on controlling the regiochemical outcome of the electrophilic trapping of the heterocyclic anion, potentially occurring at the N2 and N4 positions; (b) the intrinsically low aromaticity of the starting 1,2,4-oxadiazoles^11*b*,19^ (index of aromaticity *I*_5_ = 39 ^20^) would support the feasibility of the approach; (c) the straightforward well-established preparative methods for these starting materials^21^ would constitute a significant advantage compared to strategies that rely on precursors of limited accessibility. Ideally, regiocontrol should be predictable and tunable by the nature of the nucleophile used, as well as ring-opening/ring-closing rearrangements (ANCORC type)^11*b*^ should not come into play.

Herein, we describe the addition of variously functionalized nucleophilic elements to the C5-carbon of 1,2,4-oxadiazoles, thus selectively generating one of the two possible products, 2,5-dihydro or 4,5-dihydro regioisomers. Notably, the nucleophilic addition event leads to a geminally disubstituted cluster, with CF_3_ as a constitutive element, whereas the installed C1 synthon can be varied depending on the operator's needs. Calculations support the experimental evidence of attributing the attacking nucleophile a pivotal role in governing the regioselectivity of the process.

## Results and discussion

**Scheme 2:**
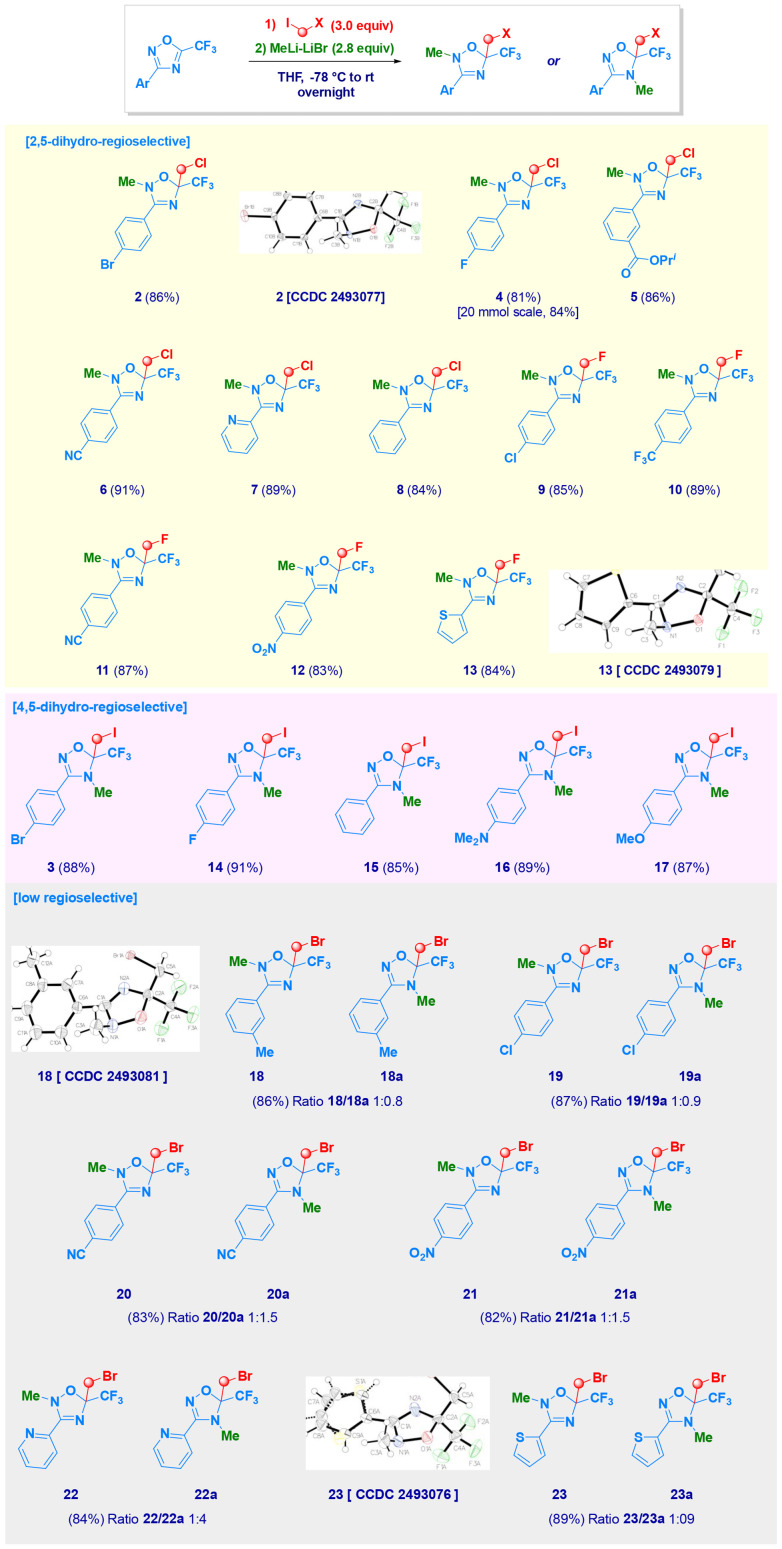
Scope of the method with different LiCH_2_X reagents.

3-(4-Bromophenyl)-5-(trifluoromethyl)-1,2,4-oxadiazole (1) was selected as the model substrate for the addition of LiCH_2_Cl generated *in situ* from chloroiodomethane and MeLi–LiBr (Table 1).^22^ By running the reaction at −78 °C in THF for 1 h, the generation of 5-chloromethyl-2-methyl-2,5-dihydro-1,2,4-oxadiazole derivative 2 was observed as the major product (49% yield, entry 1), being its structure unambiguously confirmed by X-ray analysis (see Scheme 2). Presumably, the anionic intermediate generated after the carbenoid attack is sufficiently reactive to trap, at low temperature, the electrophilic MeI formed during carbenoid generation (ICH_2_Cl + MeLi–LiBr → LiCH_2_Cl + MeI) *via* Li–I exchange.^23^ The effectiveness of the *N*-methylation was implemented by prolonging the reaction time and allowing the mixture to slowly reach rt (entries 2 and 3). The progressive increase in nucleophile loading was critical to secure the maximization of the yield up to 86% (entries 4 and 5). The positive effect of employing a supra-stoichiometric amount of carbenoid is related to its intrinsic tendency to undergo the degradative Kirmse α-elimination processes.^24^ As a matter of fact, *coeteris paribus*, reactions run in less coordinative solvents (2-MeTHF, CPME and TBME) were dramatically unproductive, as a consequence of the facilitated decomposition of the carbenoid. Augmenting the nucleophile loading to 2.2 equiv. promoted the reaction, and 2 was isolated in 78% yield (entry 4); however, to maximise the efficiency, it was essential to use 2.8 equiv., which allowed the isolation of 2 in excellent 86% yield. By running the reaction in more sustainable but less-coordinating solvents (known to promote Kirmse α-elimination), such as diethyl ether, 2-methyltetrahydrofuran (2-MeTHF),^25^ cyclopentyl methyl ether (CPME)^26^ or *tert*-butyl methyl ether (TBME),^27^ only trace amounts of product were observed (entries 6–9). Finally, a brief screening of the use of carbenoids of different natures was carried out. The reaction with the less nucleophilic magnesium carbenoid ClMgCH_2_Cl^28^ did not generate isolable products, regardless of the adoption of Barbier and *non*-Barbier conditions (entries 10 and 11). Conversely, when 1 was reacted with LiCH_2_I,^17*a*^ generated from CH_2_I_2_ and MeLi–LiBr, the regioisomer 4-methyl-4,5-dihydro-1,2,4-oxadiazole derivative 3 was surprisingly isolated in 88% yield, as the unique reaction product (entry 12). It should be emphasized that, to the best of our knowledge, C1-halocarbenoids have not previously been employed in nucleophilic additions to heterocycles.^22*b*^

**Table 1:** Reaction optimization

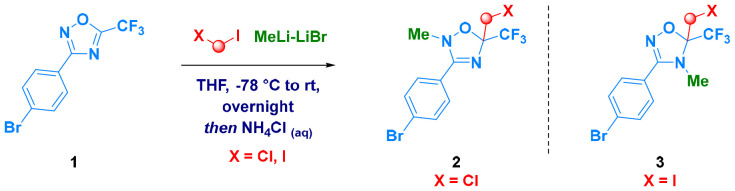				
Entry	Homologating agent (equiv.)	Solvent	Yield of 2 a (%)	Yield of 3 a (%)
1^b^	LiCH_2_Cl (1.6)	THF	49	—
2^c^	LiCH_2_Cl (1.6)	THF	63	—
3	LiCH_2_Cl (1.6)	THF	71	—
4	LiCH_2_Cl (2.2)	THF	78	—
5	LiCH_2_Cl (2.8)	THF	86	—
6	LiCH_2_Cl (2.8)	Et_2_O	37	—
7	LiCH_2_Cl (2.8)	2-MeTHF	Traces	—
8	LiCH_2_Cl (2.8)	CPME	Traces	—
9	LiCH_2_Cl (2.8)	TBME	Traces	—
10^d^	ClMgCH_2_Cl (2.8)	THF	—	—
11^e^	ClMgCH_2_Cl (2.8)	THF	—	—
12	LiCH_2_I (2.8)	THF	—	88

a Isolated yields.

b Reaction time = 1 h, temperature = −78 °C.

c Reaction time = 5 h.

d Reaction was run starting from ICH_2_Cl and i-PrMgCl–LiCl under Barbier conditions.

e Reaction was run starting from ICH_2_Cl and i-PrMgCl–LiCl under *non*-Barbier conditions.

With the optimal conditions in hand, we next explored the scope of the reaction, with the primary goal of confirming the halocarbenoid-imparted regioselectivity. Indeed, the addition of both LiCH_2_Cl and LiCH_2_F^29^ afforded 5-halomethyl-2-methyl-2,5-dihydro-oxadiazoles (2, 4–13) in yields up to 91%, with almost complete regiocontrol, also in the case of scaling up to 20 mmol (4). By contrast, nucleophilic addition of LiCH_2_I was terminated with the selective methylation at N4, thus yielding 5-iodomethyl-4-methyl-4,5-dihydro derivatives (3, 14–17) as the exclusive reaction products. The installation of the bromomethyl chain *via* LiCH_2_Br^30^ was particularly intriguing, as mixtures of the two possible regioisomers (18/18a–23/23a), corresponding to N2 and N4 methylation, were obtained in relative ratios ranging from 1 : 0.8 to 1 : 4, as deducted by integrated structural analysis (^1^H-, ^13^C-NMR and X-rays) of selected compounds (2, 13, 18, 23 and 24). *Vide infra* for a plausible rationalization of this unexpected halocarbenoid-imparted regioselectivity. The protocol was, however, highly flexible for the introduction of carbenoids into variously functionalized 1,2,4-oxadiazoles. Thus, the presence of halogens (2, 3, 9, 10 and 19/19a) did not interfere with the transformation, despite the well-known possibility of undergoing collateral halogen-metal exchange. This is particularly relevant to sp^2^-hybridized carbons featuring chlorine (9, 19/19a) or (2–3) bromine substituents.^31^ Electrophilic functional groups [*e.g.* ester (5) and nitrile (6, 11, 20)], potentially sensitive to organolithiums, were perfectly tolerated, thus conferring an excellent chemocontrol. Moreover, the basicity of MeLi and lithium carbenoids did not affect the susceptible of aromatic heterocycles to lithiation [pyridine^32^ (7, 22/22a) and thiophene^33^ (13, 23–23a)]. Halomethylation of oxadiazoles featuring the often problematic (with RLi reagents) nitro group^34^ could be successfully accomplished (12 and 21/21a) in high chemical yield. The presence of electron-donating functionalities on the phenyl ring at C3 was not detrimental, as indicated in the cases of a tertiary amine (16) and an ethereal functionality (17). Though the electrophilicity of 1,2,4-oxadiazoles could sensitively be affected, the overall result is negligible, being their reactivity comparable to systems in which more inert substituents were present [*e.g.* Me (18/18a)].

**Scheme 3:**
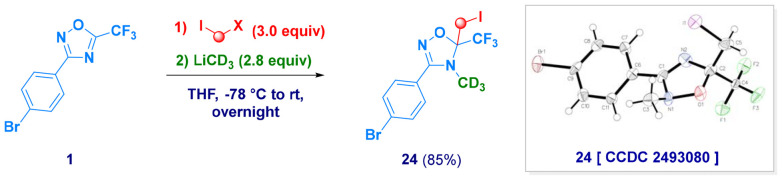
Carbenoid generation with MeLi-*d*_3_ for validating the mechanistic hypothesis.

Experimental evidence of the hypothesized mechanism was ascertained by generating *d*_3_-labeled methyl iodide during the carbenoid formation event, starting from CH_2_I_2_ and CD_3_Li.^23,35^ While the iodomethyl fragment was introduced in the standard C*H*_2_I form, the methyl group attacked by the heteroaromatic anion was deuterated (C*D*_3_), thus confirming both the origin of the electrophilic moiety and the site of functionalization (N4, 24; Scheme 3).

**Scheme 4:**
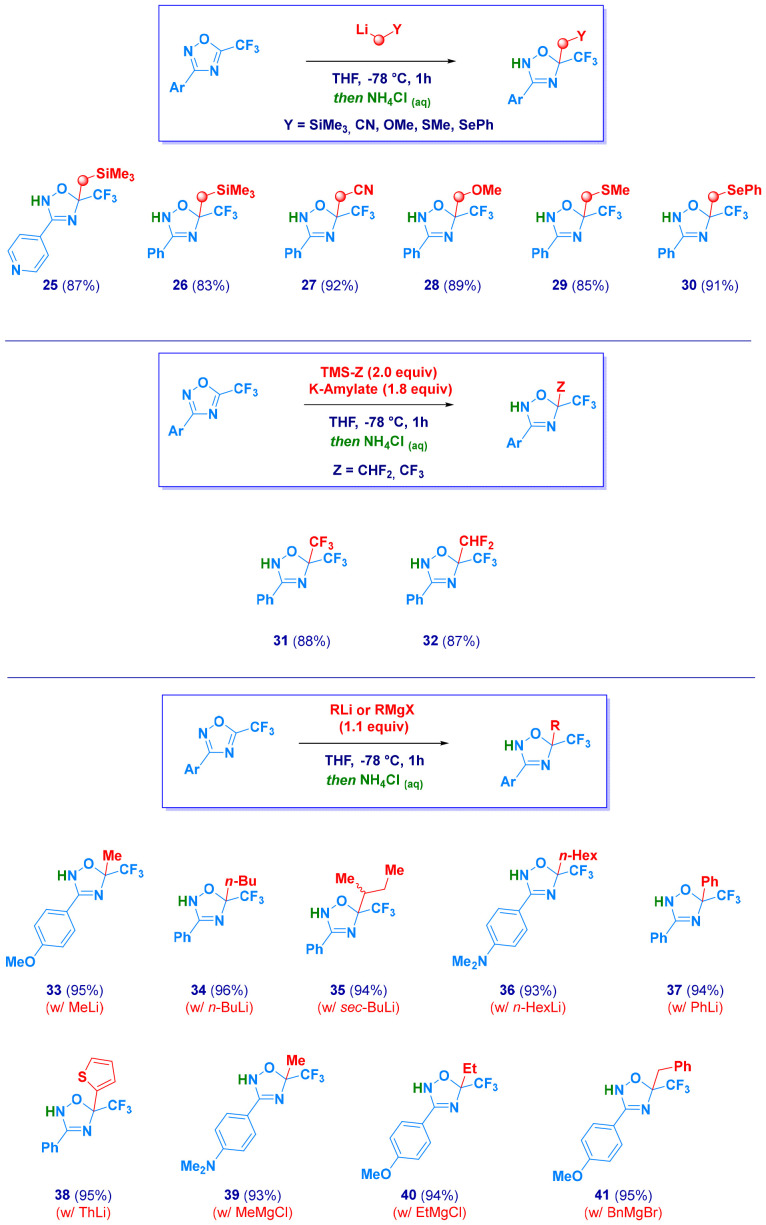
Generalization of the method with different (functionalized) carbon nucleophiles.

With the aim of expanding the dearomatization concept to different α-substituted methyl-type carbanions, we were delighted in validating the protocol with LiCH_2_SiMe_3_ (25–26), LiCH_2_CN^36^ (27), LiCH_2_OMe^37^ (28), LiCH_2_SMe^38^ (29) and LiCH_2_SePh^39^ (30). As a consequence of the generation of these nucleophiles through distinct methods rather than I/Li exchange (which forms electrophilic MeI), acidic quenching provides *N*–*H* dihydro-1,2,4-oxadiazoles (Scheme 4).

Furthermore, *gem*-functionalization could be effectively accomplished with polyfluorinated C1 units. Therefore, upon the Lewis-base-mediated activation of TMSCF_3_ (the Ruppert–Prakash reagent)^40^ and TMSCHF_2_,^41^*gem*-bis(trifluoromethyl) 31 and *gem*-difluoromethyl-trifluoromethyl 32 derivatives were prepared in high yields. Collectively, the protocol constitutes a versatile tool *en route* to rare systems presenting two distinct fluorinated chains on the same carbon atom. Finally, the addition of *non*-functionalized organolithiums^42^ and organomagnesiums^42*b*^ enabled the preparation of 5-alkyl and 5-aryl 2,5-dihydro-1,2,4-oxadiazoles (33–41). These results highlight the generality of the methodology, which allows the productive use not only of linear alkyl elements but also of more sterically hindered ones, such as the *sec*-butyl fragment (35) or less nucleophilic (hetero)aromatic rings (36 and 37).

**Scheme 5:**
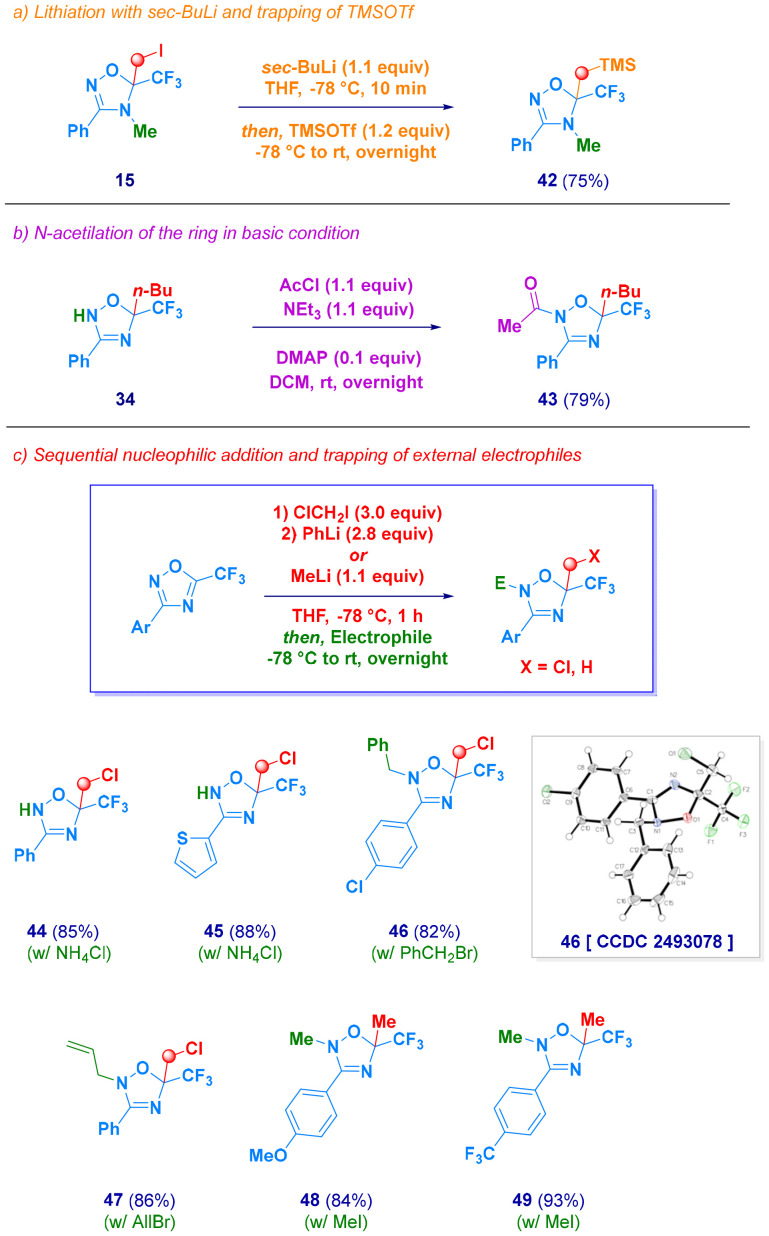
Synthetic manipulation of dihydro-1,2,4-oxadiazoles and sequential nucleophile addition/trapping with external electrophiles.

Newly synthesized dihydro-1,2,4-oxadiazoles can be surmised to undergo further derivatization (Scheme 5). For example, lithiation of compound 15 followed by the addition of TMSOTf afforded compound 42, thus showing the reactivity of the previously introduced C(sp^3^)–I bond (path *a*). Additionally, the N–H functionality can be subjected to amidation to yield the *N*-acyl derivative 43 (*path b*). Mechanistic studies, conferring the key role of carbenoid, suggests that forming it without releasing an electrophilic exchange collateral product (*e.g.* MeI) can offer the opportunity to engage an externally added functionalizing element. PhLi proved to be an excellent alternative to MeLi–LiBr for generating LiCH_2_Cl, and the PhI obtained did not interfere with electrophilic quenching, as initially endorsed with simple acidic quenching (44–45, NH_4_Cl aq.). More interestingly, this was also the case with externally added reactive carbon electrophiles [benzyl bromide (46), allyl bromide (47) and methyl iodide (48–49, *path c*)]. In agreement with the results shown above, *N*-alkylation of these anionic intermediates, generated by the installation of *non*-bulky fragments (*e.g.* –CH_2_Cl and Me-), occurs with full regioselectivity at position 2, as again confirmed by the X-ray analysis of compound 46.

The selectivity of the process was rationalized with a DFT study (see SI for more details). Comprehensive evaluations were conducted to identify the optimal strategy for an accurate description of the reacting system. This included a conformational search (using the CREST program)^43^ to determine the most stable starting molecule, prediction of the most nucleophilic sites, and evaluation of the effect of solvation to quantify steric effects (using the Multiwfn software).^44^ Figures were obtained by the Jmol program.^45^

**Scheme 6:**
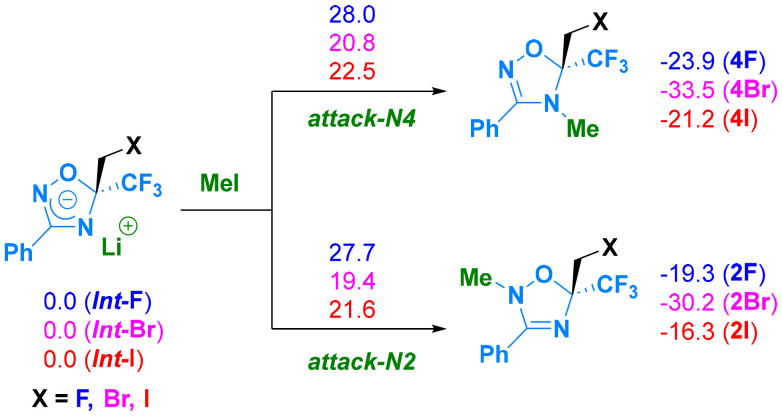
Reaction mechanisms for compounds with different halogen atoms (X = F, Br, or I). Fluorine is shown in blue, bromine is shown in pink, and iodine is shown in red. Free energies are given in kcal mol^−1^ at 195 K, calculated using CCSD(T)/def2-TZVP//M06-2X/def2-TZVP in PCM (THF).

**Fig. 1:**
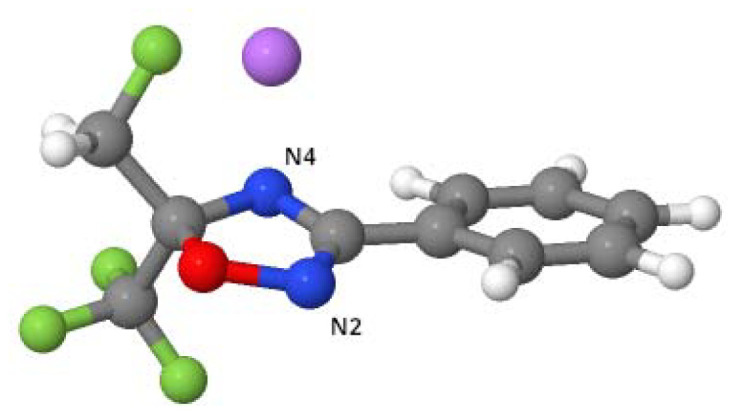
*Int*-F molecule, in which the nitrogen atom near the oxygen in the oxadiazole ring is referred to as N2, as described in this section, whereas the nitrogen at position 4, coordinated with the Li atom (pink), is referred to as N4.

**Scheme 7:**
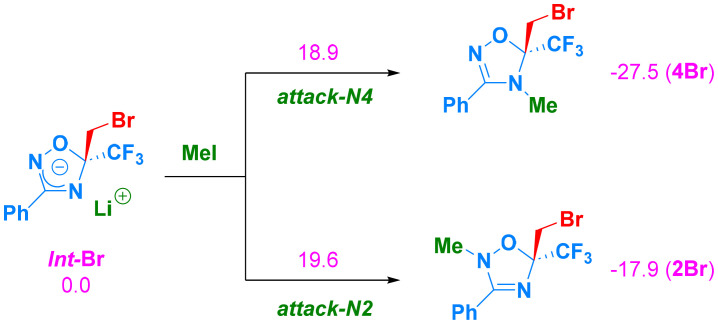
Reaction mechanisms for the compound with bromine and three explicit solvent molecules (not shown for clarity; see Fig. S13 in the SI). Free energies are given in kcal mol^−1^ at 195 K, calculated using CCSD(T)/def2-TZVP//M06-2X/def2-TZVP in PCM(THF).

Calculations using the PCM Jmol (Scheme 6) indicate that anions generated from 1,2,4-oxadiazole, bearing either fluoride or iodine (*Int*-F and *Int*-I), react with MeI (formed as a collateral product during carbenoid preparation) *via* an S_N_2-type mechanism. Because nucleophilicity is exhibited at both N2 and N4, regioisomers 4F and 2F (with fluorine) and 4I and 2I (with iodine) could be obtained upon the reaction with MeI (Fig. 1). Experimentally, only compounds 2F and 4I were generated, whereas in the presence of bromine, both 2Br and 4Br were observed. However, in the case of fluorine, the transition state (TS*Int*-F → 2F) leading to the experimental product 2F does not differ significantly from TS*Int*-I → 4I, from which 4I is obtained. Regarding reaction energies, the experimental product (2F) is 4.6 kcal mol^−1^ higher in energy than 4F (−19.3 kcal mol^−1^*vs.* −23.9 kcal mol^−1^). For iodine, the transition state leading to the experimentally observed product (TS*Int*-I v 2I) is 0.9 kcal mol^−1^ higher than TS*Int*-I → 4I, despite the reaction energy of 4I (the experimental product) being more stable than that of 2I (−21.2 kcal mol^−1^*vs.* −16.3 kcal mol^−1^). In the case of bromine, the transition states leading to the two different products differ by 1.4 kcal mol^−1^ (TS*Int*-Br → 4Br and TS*Int*-Br → 2Br). With such an energy difference, only 2Br would be observed. PCM simulation of the solvent could not explain the observed product distribution in the case of bromine. However, when three molecules of THF were explicitly included in the calculations, the energy difference between the two barriers decreased to 0.8 kcal mol^−1^, which is qualitatively in (better) agreement with the experimental findings (Scheme 7).

To explain the regioselectivity, an electronic effect was ruled out because the nucleophilicity of the two nitrogen atoms was unchanged regardless of the orientation of the halogen atom in the molecule and the two different lithium coordination sites; as also indicated in Table 2, the nitrogen at the 2-position consistently exhibits higher nucleophilicity, irrespective of the lithium position.

**Table 2:** Condensed nucleophilicity indices, N^A^_Nu_, for the nitrogen atoms at the 2- and 4-positions, expressed as *e* × eV, where *e* is the elementary charge. The Fig. 1 shows the *Int*-F molecule, in which the nitrogen atom near the oxygen in the isoxazole ring is referred to as N2, while the nitrogen at position 4 is referred to as N4. Only the most stable structures were considered. For more details, see the SI

Compound	N2	N4
F	0.62602	0.51844
I	0.6361	0.53307
Br	0.57182	0.49387

**Fig. 2:**
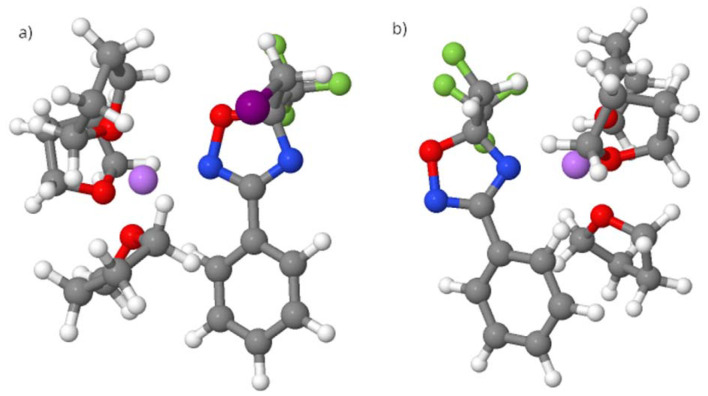
Structures of the (a) *Int*-I compound and (b) *Int*-F compound, in which the iodine atom is replaced with fluorine (I → F). Structure (b) is 0.4 kcal mol^−1^ less stable than (a).

**Fig. 3:**
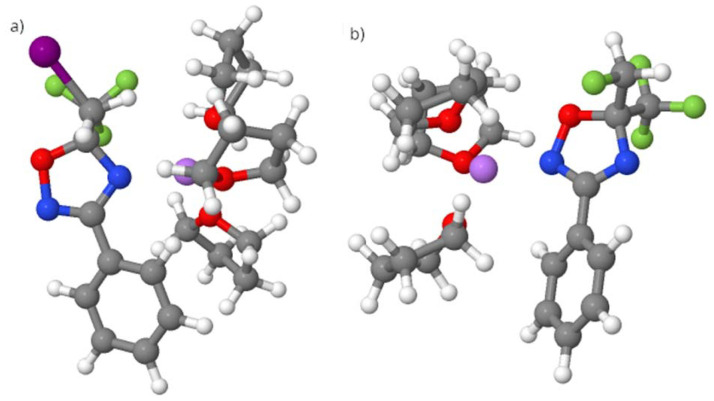
Structure of the (a) *Int*-F compound and (b) *Int*-I compound, in which the fluorine atom is replaced with iodine (F → I). Structure (b) is 4.7 kcal mol^−1^ less stable than (a).

The most stable conformation for each compound (with Li bound to N2 or N4; Fig. 2 and 3) containing fluorine or iodine was considered. The geometries were optimized, and the energies were decomposed into three terms according to the EDA-SBL approach (Table 2).

For the product with iodine, the conformation with Li bound to N2 (Fig. 2a) is the most stable, and energy decomposition analysis (EDA-SBL) suggests that the larger steric bulk of iodine influences its conformation, leading to different products under identical conditions. As shown in Table S9 (SI), when iodine was replaced with fluorine (I → F, Fig. 2b) while keeping the same conformation, the steric energy was 9.3 kcal mol^−1^. In contrast, when fluorine was replaced with iodine (F → I) (Fig. 3), the steric energy increased significantly to 19.4 kcal mol^−1^.

This scenario suggests significant steric bulk in the second case, which could explain why iodine adopts a specific conformation, leading to different products under the same conditions. The atomic radius of bromine is intermediate between those of F and I, and the steric effect is no longer dominant over the stability of the two intermediates; therefore, both products are observed.

## Conclusions

In summary, we introduced a novel synthetic approach for the preparation of dihydro-1,2,4-oxadiazoles through the direct addition of carbon nucleophiles to 5-trifluoromethyl-1,2,4-oxadiazoles. The dearomatization process occurs *via* the regioselective nucleophilic attack at C5, and in the case of halogenated C1-species (*i.e.* carbenoids), the nature of the competent halogen is the key factor imparting regioselectivity to the process. Thus, in the case of using chloromethyl- and fluoromethyl-lithiums, only 2,5-dihydro isomers were generated, whereas switching to iodomethyl-lithium yielded 4,5-dihydro isomers. The nucleophilicity of the anionic intermediate enables further functionalization of the ring with externally added electrophilic partners. The portfolio of carbon nucleophiles amenable to the transformation is wide, as demonstrated with diverse (α-substituted) carbanions and fragments released from the proper genesis of *ate* complexes (*e.g.* activated TMSCHF_2_ and TMSCF_3_). The transformation takes place under full chemocontrol and maintains the chemical integrity of sensitive functionalities (halogens, nitriles, esters, pyridines and thiophenes). Unambiguous assignment of regioisomers was deduced by X-ray analysis and the use of deuterium-labelled reagents, whereas DFT calculations supported the experimental observations.

## Conflicts of interest

The authors declare no competing interests.

## Supplementary Material

QO-013-D5QO01707F-s001

QO-013-D5QO01707F-s002

## Data Availability

The data that support the findings of this study are available from the corresponding author upon reasonable request. The data supporting this article have been included as part of the supplementary information (SI). Supporting Information includes Instrumentation and General Analytical Methods, Synthesis, characterization and Spectral Data of the Compounds, X-Ray Analysis, Computational Details and Cartesian coordinates of the optimized structures. See DOI: https://doi.org/10.1039/d5qo01707f. CCDC 2493076–2493081 (23, 2, 46, 13, 24 and 18) contain the supplementary crystallographic data for this paper.^46*a*–*f*^
