# Supplementary material for: One-pot dearomatizative telescoped addition of C-nucleophiles to fluorinated 1,2,4-oxadiazoles followed by regioselective N-functionalization
Source: Org Chem Front. 2026 Jan 9;13(8):2401–9. doi: 10.1039/d5qo01707f (PMC12937061; doi:10.1039/d5qo01707f)
Supplement: QO-013-D5QO01707F-s001 [file QO-013-D5QO01707F-s001.pdf]

Supplementary Information (SI) for Organic Chemistry Frontiers.
This journal is © the Partner Organisations 2026

ELECTRONIC SUPPORTING INFORMATION
For

One-pot dearomatizative telescoped addition of *C*-nucleophiles to fluorinated 1,2,4-oxadiazoles followed by regioselective *N*-functionalization

Davide Castiglione,[a] Sara Amata,[b] Federica Lauria,[a] Andrea Maranzana,[a] Salvatore Baldino,[a] Alexander Roller-Prado,[c] Laura Castoldi,[d*] Antonio Palumbo Piccionello,[b*] Vittorio Pace[e,f*] and Eisuke I. Comas Iwasita[a]

[a] University of Turin – Department of Chemistry, Via Giuria 7, 10125 Turin, Italy.

[b] University of Palermo – Department of Biological, Chemical and Pharmaceutical Sciences and Technologies, Viale delle Scienze Ed.17, 90128, Palermo, Italy. E-mail: antonio.palumbopiccionello@unipa.it

[c] University of Vienna - Department of Inorganic Chemistry – Functional Materials, Waehringerstrasse 42, 1090, Vienna, Austria.

[d] University of Milan – Department of Pharmaceutical Sciences, General and Organic Chemistry Section "A. Marchesini" – Via Venezian 21, 20133 Milan, Italy

[e] University of Rome "La Sapienza" - Department of Chemistry - P.le A. Moro, 5, 00185 Rome (Italy). E-mail: vittorio.pace@uniroma1.it

[f] University of Vienna - Department of Pharmaceutical Sciences, Josef-Holaubek-

Table of contents

Instrumentation and General Analytical Methods 2

Synthesis and characterization of Starting Materials 3

General procedures for the synthesis of substituted 5-trifluoromethyl-dihydro-1,2,4-oxadiazoles 6

Characterization and Spectral Data of the Compounds 7

Synthetic manipulation of products 24

Copies of ^{1}H, ^{13}C and ^{19}F-NMR Spectra of all compounds 28

X-Ray Analysis for compounds 2, 13, 18, 23, 24 and 46 109

Computational Details 124

References 141

Instrumentation and General Analytical Methods

^{1}H and ^{13}C-NMR spectra were recorded on a Jeol ECZR600 spectrometer operating at 600 MHz (600 MHz for ^{1}H, 150 MHz for ^{13}C, 565 MHz for ^{19}F) and on a Bruker Avance Neo 400 spectrometer (400 MHz for ^{1}H, 100 MHz for ^{13}C, 377 MHz for ^{19}F). The center of the (residual) solvent signal was used as an internal standard which was related to TMS with δ 7.26 ppm (^{1}H in $CDCl_3$), δ 77.2 ppm (^{13}C in $CDCl_3$), 2.05 ppm (^{1}H in acetone-d_6) and 202.26 – 29.84 ppm (^{13}C in acetone-d_6). Spin-spin coupling constants (J) are given in Hz. Mass data were obtained on Agilent UHD accurate-mass Q-TOF LC/MS.
Starting materials were prepared as detailed below; those ones not reported in the manuscript are indicated with the descriptor **nn-*sm***, being **nn** the corresponding final compound in the manuscript. All reactions were performed under an inert atmosphere of argon using standard Schlenk techniques. THF and Et_2O were distilled over Na/benzophenone. Chemicals and dry solvents were purchased from SigmaAldrich, Acros, Alfa Aesar, Fluorochem and TCI Europe. Solutions were evaporated under reduced pressure with a rotary evaporator. Melting points were determined on a Reichert–Kofler hot-stage microscope and are uncorrected. For column chromatography, silica Gel 60 (0.04-0.063 mm) was used. TLC was carried out on aluminium sheets precoated with silica gel 60F254 (Merchery-Nagel, Merk); the spots were visualized under UV light (λ = 254 nm) and/or $KMnO_{4\ (aq.)}$ was used as revealing system.

Synthesis and characterization of Starting Materials

Most of 3-aryl-5-(trifluoromethyl)-1,2,4-oxadiazoles employed as starting materials are known substrates and were prepared as previously reported (see Table below).

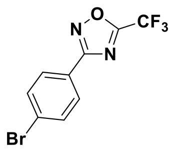

Y. Huang *et al.*
Org. Lett. 2022, **24**, 2055

1

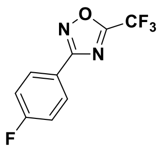

Ali Darehkordi *et al.*
J. Heterocycl. Chem.
2018, **55**, 1702

4-*sm*

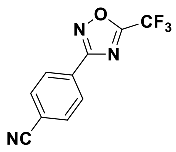

Y. Huang *et al.*
Org. Lett. 2022, **24**, 2055

6-*sm*

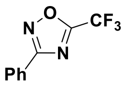

Buscemi, S. *et al.*
*J. Org. Chem. 2003, **68**, 2, 605*

8-*sm*

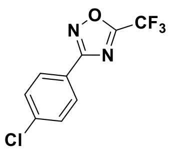

Y. Huang *et al.*
Org. Lett. 2022, **24**, 2055

9-*sm*

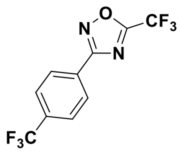

P. P. Onys'ko. *et al.*
Tetrahedron 2014, **70**, 2928

10-*sm*

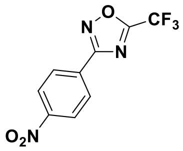

Buscemi, S. *et al.*
Eur. J. Org. Chem. 2004, **5**, 974

12-*sm*

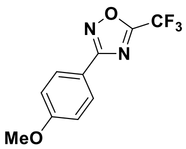

Buscemi, S. *et al.*
Eur. J. Org. Chem. 2004, **5**, 974

17-*sm*

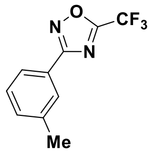

E. Camaioni *et al.*
Bioorg. Med. Chem.
2020, **28**, 115731

18-*sm*

Table 1. Previously reported starting materials.

Unknown analogues were obtained adapting the reported procedure[1], according to the general protocol:

A solution of the aryl-amidoxime (1.0 mmol, 1.0 equiv) and trifluoroacetic anhydride (1.2 mmol, 1.2 equiv) in anhydrous toluene (10 mL) was refluxed for 8 h. After removal of the solvent, the residue was treated with water and the organic phase was extracted with EtOAc, washed with brine, dried over Na_2SO_4 and concentrated under reduced pressure. The final 3-aryl-5-trifluoromethyl-1,2,4-oxadiazole was obtained after purification by column chromatography on silica gel, as reported below.

Isopropyl 3-(5-(trifluoromethyl)-1,2,4-oxadiazol-3-yl)benzoate (5-*sm*)

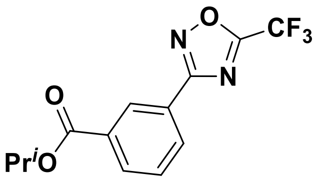

By following the general protocol, compound **5-*sm*** was obtained in 69% yield as colorless oil after chromatography on silica gel (50:1 v/v, petroleum ether/ethyl acetate).
^{1}H-NMR (400 MHz, $CDCl_3$) δ: 8.75 (t, 1H, J=1.8 Hz), 8.28 (dt, 1H, J=7.9, 1.5 Hz), 8.24 (dt, 1H, J=7.8, 1.5 Hz), 7.60 (t, 1H, J=7.8 Hz), 5.30 (hept, 1H, J=6.3 Hz), 1.40 (d, 6H, J=6.3 Hz).
^{13}C-NMR (100 MHz, $CDCl_3$) δ: 168.6 (s), 166.06 (q, J=44.6 Hz), 165.0 (s), 133.1 (s), 132.1(s), 131.6 (s), 129.3 (s), 128.7 (s), 125.3 (s), 116.0 (q, J=273.7 Hz), 69.1 (s), 21.9 (s).
^{19}F-NMR (377 MHz, $CDCl_3$) δ: -65.4 (s).
GC/MS distribution for $C_{13}H_{11}N_2O_3F_3$ 69(100%), 241 (100%), 259 (40%), 300 (5%)

3-(pyridin-2-yl)-5-(trifluoromethyl)-1,2,4-oxadiazole (7-*sm*)

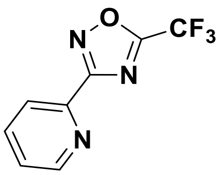

By following the general protocol, compound **7-*sm*** was obtained in 33% yield as yellow oil after chromatography on silica gel (50:1 v/v, petroleum ether/ethyl acetate).
^{1}H-NMR (600 MHz, $CDCl_3$) δ: 8.81 (ddd, 1H, J=4.8, 1.7, 0.9 Hz), 8.15 (dt, 1H, J=7.9, 1.1 Hz), 7.89 (td, 1H, J=7.8, 1.8 Hz), 7.48 (ddd, 1H, J=7.7, 4.8, 1.2 Hz).
^{13}C-NMR (150 MHz, $CDCl_3$) δ: 168.8 (s), 166.4 (q, J=44.7 Hz), 150.7 (s), 144.6 (s), 137.3 (s), 126.4 (s), 123.6 (s), 115.8 (q, J=274.0 Hz).
^{19}F-NMR (565 MHz, $CDCl_3$) δ: -65.2 (s).
HRMS (ESI) $[M+H]^+$: m/z for $C_8H_5N_3OF_3^+$ theoretical: 216.0379; found: 216.0396.

3-(thiophen-2-yl)-5-(trifluoromethyl)-1,2,4-oxadiazole (13-*sm*)

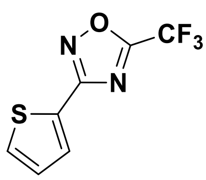

By following the general protocol, compound **13-*sm*** was obtained in 41% yield as yellow oil after chromatography on silica gel (50:1 v/v, petroleum ether/ethyl acetate).
^{1}H-NMR (600 MHz, $CDCl_3$) δ: 7.88 (dd, J=3.7, 1.2 Hz), 7.59 (dd, J=5.0, 1.2 Hz), 7.19 (dd, J=5.0, 3.7 Hz).
^{13}C-NMR (150 MHz, $CDCl_3$) δ: 165.6 (q, J=44.5 Hz), 165.2 (s), 131.2 (s), 130.8 (s), 128.3 (s), 126.1 (s), 115.9 (q, J=273.9 Hz).
^{19}F-NMR (565 MHz, $CDCl_3$) δ: -65.3 (s).
GC/MS distribution for $C_7H_3N_2OSF_3$ 69(100%), 125(25%), 220 (50%).

N,N-dimethyl-4-(5-(trifluoromethyl)-1,2,4-oxadiazol-3-yl)aniline (16-*sm*)

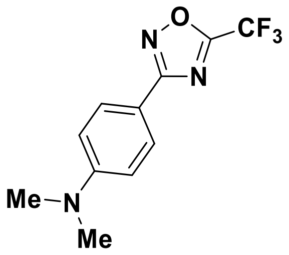

By following the general protocol, compound **16-*sm*** was obtained in 62% yield as white solid (m.p.: 95-96 °C) after chromatography on silica gel (50:1 v/v, petroleum ether/ethyl acetate).
^{1}H-NMR (600 MHz, $CDCl_3$) δ: 7.95 (m, 2H), 6.74 (m, 2H), 3.06 (s, 6H).
^{13}C-NMR (150 MHz, $CDCl_3$) δ: 169.2 (s), 165.0 (q, J=43.9 Hz), 152.7 (s), 129.0 (s), 116.2 (q, J=273.5 Hz), 111.7 (s), 111.6 (s), 40.0 (s).
^{19}F-NMR (565 MHz, $CDCl_3$) δ: -65.4 (s).
HRMS (ESI) $[M+H]^+$: m/z for $C_{11}H_{11}N_3OF_3^+$ theoretical 258.0849; found: 258.0853

3-(pyridin-4-yl)-5-(trifluoromethyl)-1,2,4-oxadiazole (24-*sm*)

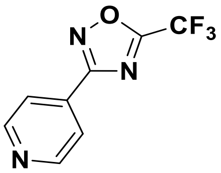

By following the general protocol, compound **24-*sm*** was obtained in 55% yield as yellow oil after chromatography on silica gel (50:1 v/v, petroleum ether/ethyl acetate).
^{1}H-NMR (600 MHz, $CDCl_3$) δ: 8.89 (d, 2H, J=6.3 Hz), 8.14 (d, 2H, J=6.3 Hz).
^{13}C-NMR (150 MHz, $CDCl_3$) δ: 167.2 (s), 166.9 (d, q=45.0 Hz), 149.1 (s), 134.2 (s), 122.2 (s), 115.7 (q, J=274.1 Hz).
^{19}F-NMR (565 MHz, $CDCl_3$) δ: -65.3 (s).
HRMS (ESI) $[M+H]^+$: m/z for $C_8H_5N_3OF_3^+$ theoretical: 216.0379; found 216.0396

General procedures for the synthesis of substituted 5-trifluoromethyl-dihydro-1,2,4-oxadiazoles

General procedure for the addition of lithium halocarbenoids and trapping of MeI: (GP1)
Under Ar atmosphere, to the solution of 3-aryl-5-(trifluoromethyl)-1,2,4-oxadiazoles (1.0 equiv) in dry THF (THF/Et_2O 1:1 for fluoromethylation), the competent dihalomethane (3 equiv) was added and the resulting solution was cooled down to -78 °C. Then MeLi-LiBr (2.2 M in Et_2O, 2.8 equiv) was added dropwise and the mixture was stirred overnight and allowed to gradually reach room temperature; subsequently, a saturated solution of $NH_4Cl_{(aq.)}$ was added. The organic phase was extracted with EtOAc (3 x 10 mL) and was washed with brine then, was dried over anhydrous Na_2SO_4 and the solvent was removed under reduced pressure. The final product was obtained after purification by column chromatography on silica gel.

General procedure for the addition of organolithium and Grignard reagents (GP2)
Under Ar atmosphere, to the solution of 3-aryl-5-(trifluoromethyl)-1,2,4-oxadiazoles (1.0 equiv) in dry THF, cooled to -78 °C, the competent organolithium or Grignard reagent (2.8 equiv) was added dropwise and, the mixture was stirred for 1 hour; subsequently, a saturated solution of $NH_4Cl_{(aq.)}$ was added. The organic phase was extracted with EtOAc (3 x 10 mL) and was washed with brine then, was dried over anhydrous Na_2SO_4 and the solvent was removed under reduced pressure. The final product was obtained after filtration on silica plug.

General procedure for the addition of lithium halocarbenoids and protonation at position 2: (GP3)
Under Ar atmosphere, to the solution of 3-aryl-5-(trifluoromethyl)-1,2,4-oxadiazoles (1.0 equiv) in dry THF (or dry 1:1 THF/EtO_2 for fluoromethylation), the competent dihalomethane (3 equiv) was added and the resulting solution was cooled down to -78 °C. Then PhLi (1.9 M in dibutyl ether, 2.8 equiv) was added dropwise and, the mixture was stirred for 1 hour; subsequently, a saturated solution of $NH_4Cl_{(aq.)}$ was added. The organic phase was extracted with EtOAc (3 x 10 mL) and was washed with brine then, was dried over anhydrous Na_2SO_4 and the solvent was removed under reduced pressure. The final product was obtained after purification by column chromatography on silica gel.

General procedure for the addition of lithium halocarbenoids and trapping of an external electrophile: (GP4)
Under Ar atmosphere, to the solution of 3-aryl-5-(trifluoromethyl)-1,2,4-oxadiazoles (1.0 equiv) in dry THF (or dry 1:1 THF/EtO_2 for fluoromethylation), the competent dihalomethane (3 equiv) was added and the resulting solution was cooled down to -78 °C. Then PhLi (1.9 M in dibutyl ether, 2.8 equiv) was added dropwise and, the mixture was stirred for 1 hour, then the electrophile was added dropwise to the solution. The mixture was stirred overnight and allowed to gradually reach room temperature. Subsequently, a saturated solution of $NH_4Cl_{(aq.)}$ was added. The organic phase was extracted with EtOAc (3 x 10 mL) and was washed with brine then, was dried over anhydrous Na_2SO_4 and the solvent was removed under reduced pressure. The final product was obtained after purification by column chromatography on silica gel.

Characterization and Spectral Data of the Compounds

3-(4-bromophenyl)-5-(chloromethyl)-2-methyl-5-(trifluoromethyl)-2,5-dihydro-1,2,4-oxadiazole (2)

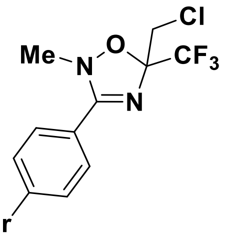

By following the general procedure 1 (GP1), starting from 3-(4-bromophenyl)-5-(trifluoromethyl)-1,2,4-oxadiazole (293.4 mg, 1.0 mmol, 1.0 equiv), chloroiodomethane (529.1 mg, 0.22 mL, 3.0 mmol, 3.0 equiv) and MeLi-LiBr complex (1.27 mL of a solution 2.2 M in Et_2O, 2.8 mmol, 2.8 equiv) in THF (5 mL), compound **2** was obtained in 86% yield (307.5 mg) as white solid (m.p.: 92 °C) after chromatography on silica gel (97:3 v/v, petroleum ether/ethyl acetate).
^{1}H-NMR (600 MHz, $CDCl_3$) δ: 7.62 (br s, 4H), 4.02 (d, 1H, J = 12.6 Hz), 3.82 (d, 1H, J = 12.2 Hz), 3.16 (s, 3H).
^{13}C-NMR (150 MHz, $CDCl_3$) δ: 168.7 (s), 132.3 (s), 130.3 (s), 127.2 (s), 124.7 (s), 121.9 (q, J = 288.0 Hz), 105.7 (q, J = 30.1 Hz), 43.4 (s), 42.5 (s).
^{19}F-NMR (565 MHz, $CDCl_3$) δ: -80.8 (s).
HRMS (ESI), m/z: calc. for $C_{11}H_9BrClF_3N_2O^+$: 356,9612 $[M+H]^+$; found: 356,9617.

3-(4-bromophenyl)-5-(iodomethyl)-4-methyl-5-(trifluoromethyl)-4,5-dihydro-1,2,4-oxadiazole (3)

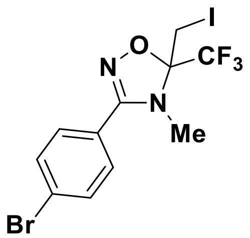

By following the general procedure 1 (GP1), starting from 3-(4-bromophenyl)-5-(trifluoromethyl)-1,2,4-oxadiazole (293.4 mg, 1.0 mmol, 1.0 equiv), diiodomethane (903.5 mg, 0.24 mL, 3.0 mmol, 3.0 equiv) and MeLi-LiBr complex (1.27 mL of a solution 2.2 M in Et_2O, 2.8 mmol, 2.8 equiv) in THF (5 mL), compound **3** was obtained in 88% yield (395.1 mg) as white solid (m.p.: 70 °C) after chromatography on silica gel (97:3 v/v, petroleum ether/ethyl acetate).
^{1}H-NMR (600 MHz, $CDCl_3$) δ: 7.63 (br s, 4H), 3.84 (d, 1H, J = 11.4 Hz), 3.60 (d, 1H, J = 11.5 Hz), 3.23 (s, 3H).
^{13}C-NMR (150 MHz, $CDCl_3$) δ: 168.2 (s), 132.3 (s), 130.3 (s), 127.2 (s), 124.8 (s), 119.7 (q, J = 288.8 Hz), 104.9 (q, J = 30.4 Hz), 42.2 (s), 7.8 (s).
^{19}F-NMR (565 MHz, $CDCl_3$) δ: -80.6 (s).
HRMS (ESI), m/z: calc. for $C_{11}H_{10}BrF_3IN_2O^+$: 448,8968 $[M+H]^+$; found: 448,8970.

5-(chloromethyl)-3-(4-fluorophenyl)-2-methyl-5-(trifluoromethyl)-2,5-dihydro-1,2,4-oxadiazole (4)

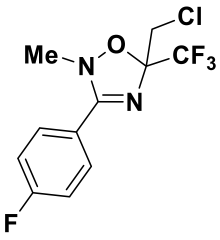

By following the general procedure 1 (GP1), starting from 3-(4-fluorophenyl)-5-(trifluoromethyl)-1,2,4-oxadiazole (232.1 mg, 1.0 mmol, 1.0 equiv), chloroiodomethane (529.1 mg, 0.22 mL, 3.0 mmol, 3.0 equiv) and MeLi-LiBr complex (1.27 mL of a solution 2.2 M in Et_2O, 2.8 mmol, 2.8 equiv) in THF (5 mL), compound **4** was obtained in 81% yield (240.2 mg) as yellow solid (m.p.: 70 °C) after chromatography on silica gel (98:2 v/v, petroleum ether/ethyl acetate).

Scale-up to 20 mmol:

By following the general procedure 1 (GP1), starting from 3-(4-fluorophenyl)-5-(trifluoromethyl)-1,2,4-oxadiazole (4.64 g, 20.0 mmol, 1.0 equiv), chloroiodomethane (10.58 g, 4.4 mL, 60.0 mmol, 3.0 equiv) and MeLi-LiBr complex (25.4 mL of a solution 2.2 M in Et_2O, 56.0 mmol, 2.8 equiv) in THF (100 mL), compound **4** was obtained in 84% yield (4.98 g) as yellow solid (m.p.: 70 °C) after chromatography on silica gel (98:2 v/v, petroleum ether/ethyl acetate).

^{1}H-NMR (600 MHz, $CDCl_3$) δ: 7.77 (m, 2H), 7.19 - 7.13 (m, 2H), 4.02 (d, 1H, J=12.2 Hz), 3.82 (d, 1H, J=12.2 Hz), 3.17 (s, 3H).

^{13}C-NMR (150 MHz, $CDCl_3$) δ: 168.6 (s), 165.2 (d, J=254.2 Hz), 131.26 (d, J=8.9 Hz), 121.9 (q, J=287.7 Hz), 122.00 (d, J=2.7 Hz), 116.32 (s), 116.17 (s), 105.65 (q, J=30.1 Hz), 43.5 (s), 42.6 (s).

^{19}F-NMR (565 MHz, $CDCl_3$) δ: -80.8 (s).

HRMS (ESI), m/z: calc. for $C_{11}H_9ClF_4N_2O^+$: 297,0412 $[M+H]^+$; found: 297,0430.

Isopropyl 3-(5-(chloromethyl)-2-methyl-5-(trifluoromethyl)-2,5-dihydro-1,2,4-oxadiazol-3-yl)benzoate (5)

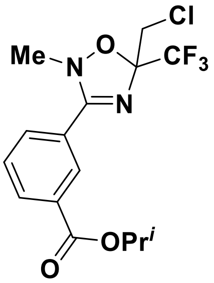

By following the general procedure 1 (GP1), starting from isopropyl 3-(5-(trifluoromethyl)-1,2,4-oxadiazol-3-yl)benzoate (300.2 mg, 1.0 mmol, 1.0 equiv), chloroiodomethane (529.1 mg, 0.22 mL, 3.0 mmol, 3.0 equiv) and MeLi-LiBr complex (1.27 mL of a solution 2.2 M in Et_2O, 2.8 mmol, 2.8 equiv) in THF (5 mL), compound **5** was obtained in 86% yield (312.6 mg) as yellow oil after chromatography on silica gel (92:8 v/v, petroleum ether/ethyl acetate).

^{1}H-NMR (400 MHz, $CDCl_3$) δ: 8.35 (s, 1H), 8.21 (d, 1H, J=7.8 Hz), 7.93 (d, 1H, J=7.7 Hz), 7.55 (t, 1H, J=7.8 Hz), 5.26 (hept, 1H, J=6.3 Hz, 4.02 (d, 1H, J=12.2 Hz), 3.82 (d, 1H, J=12.3 Hz), 3.17 (s, 3H), 1.37 (d, 6H, J=6.4 Hz).

^{13}C-NMR (100 MHz, $CDCl_3$) δ: 168.8 (s), 164.7 (s), 133.3 (s), 132.8 (s), 131.8 (s), 129.7 (s), 129.1 (s), 126.2 (s), 121.9 (q, J=287.9 Hz), 105.7 (q, J=30.2 Hz), 69.1 (s), 43.4 (s), 42.4 (s), 21.8 (s).

^{19}F-NMR (377 MHz, $CDCl_3$) δ: -80.9 (s).

HRMS (ESI), m/z: calc. for $C_{15}H_{17}ClF_3N_2O_3^+$: 365,0874 $[M+H]^+$; found: 365,0875.

4-(5-(chloromethyl)-2-methyl-5-(trifluoromethyl)-2,5-dihydro-1,2,4-oxadiazol-3-yl) benzonitrile (6)

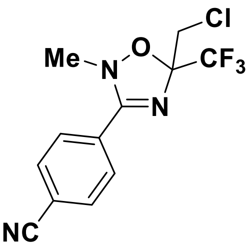

By following the general procedure 1 (GP1), starting from 4-(5-(trifluoromethyl)-1,2,4-oxadiazol-3-yl)benzonitrile (239.2 mg, 1.0 mmol, 1.0 equiv), chloroiodomethane (529.1 mg, 0.22 mL, 3.0 mmol, 3.0 equiv) and MeLi-LiBr complex (1.27 mL of a solution 2.2 M in Et_2O, 2.8 mmol, 2.8 equiv) in THF (5 mL), compound **6** was obtained in 91% yield (276.4 mg) as white solid (m.p.: 118 °C) after chromatography on silica gel (89:11 v/v, petroleum ether/ethyl acetate).
^{1}H-NMR (600 MHz, $CDCl_3$) δ: 7.90 - 7.82 (m, 2H), 7.81 - 7.73 (m, 2H), 4.02 (d, 1H, J=12.3 Hz), 3.82 (d, 1H, J=12.3 Hz), 3.16 (s, 3H).
^{13}C-NMR (150 MHz, $CDCl_3$) δ: 168.0 (s), 132.8 (s), 130.2 (s), 129.7 (s), 121.9 (q, J = 287.7 Hz), 117.7 (s), 116.2 (s), 106.1 (q, J = 30.3 Hz), 43.5 (s), 42.4 (s).
^{19}F-NMR (565 MHz, $CDCl_3$) δ: -80.8 (s).
HRMS (ESI), m/z: calc. for $C_{12}H_9ClF_3N_3O^+$: 304,0459 $[M+H]^+$; found: 304,0460.

5-(chloromethyl)-2-methyl-3-(pyridin-2-yl)-5-(trifluoromethyl)-2,5-dihydro-1,2,4-oxadiazole (7)

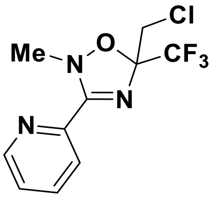

By following the general procedure 1 (GP1), starting from 3-(pyridin-2-yl)-5-(trifluoromethyl)-1,2,4-oxadiazole (215.1 mg, 1.0 mmol, 1.0 equiv), chloroiodomethane (529.1 mg, 0.22 mL, 3.0 mmol, 3.0 equiv) and MeLi-LiBr complex (1.27 mL of a solution 2.2 M in Et_2O, 2.8 mmol, 2.8 equiv) in THF (5 mL), compound **7** was obtained in 89% yield (248.8 mg) as white solid (m.p.: 33 °C) after chromatography on silica gel (87:13 v/v, petroleum ether/ethyl acetate).
^{1}H-NMR (600 MHz, $CDCl_3$) δ: 8.67 (ddd, 1H, J = 4.8, 1.7, 0.9 Hz), 8.14 (dt, 1H, J = 7.9, 1.0 Hz), 7.82 (td, 1H, J = 7.8, 1.8 Hz), 7.44 (ddd, 1H J = 7.7, 4.8, 1.2 Hz), 4.01 (d, 1H, J = 12.5 Hz), 3.83 (d, 1H J = 12.2 Hz), 3.51 (s, 3H).
^{13}C-NMR (150 MHz, $CDCl_3$) δ: 166.5 (s), 149.3 (s), 145.5 (s), 137.0 (s), 126.3 (s), 125.2 (s), 122.0 (q, J = 288.1 Hz), 105.0 (q, J = 30.2 Hz), 43.4 (s), 42.2 (s).
^{19}F-NMR (565 MHz, $CDCl_3$) δ: -80.8 (s).
HRMS (ESI), m/z: calc. for $C_{10}H_{10}ClF_3N_3O^+$: 280,0459 $[M+H]^+$; found: 280,0464.

5-(chloromethyl)-2-methyl-3-phenyl-5-(trifluoromethyl)-2,5-dihydro-1,2,4-oxadiazole (8)

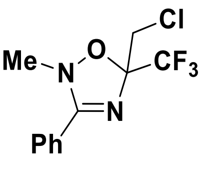

By following the general procedure 1 (GP1), starting from 3-(phenyl)-5-(trifluoromethyl)-1,2,4-oxadiazole (214.2 mg, 1.0 mmol, 1.0 equiv), chloroiodomethane (529.1 mg, 0.22 mL, 3.0 mmol, 3.0 equiv) and MeLi-LiBr complex (1.27 mL of a solution 2.2 M in Et_2O, 2.8 mmol, 2.8 equiv) in THF (5 mL), compound **8** was obtained in 84% yield (234.0 mg) as white solid (m.p.: 61 °C) after chromatography on silica gel (98:2 v/v, petroleum ether/ethyl acetate).

^{1}H-NMR (600 MHz, $CDCl_3$) δ: 7.77-7.74 (m, 2H), 7.58-7.54 (m, 1H), 7.47 (m, 2H), 4.04 (d, 1H, J=12.1 Hz), 3.84 (d, 1H J=12.2 Hz), 3.19 (s, 3H).

^{13}C-NMR (150 MHz, $CDCl_3$) δ: 169.6 (s), 132.4 (s), 128.9 (s), 125.9 (s), 122.0 (q, J = 287.9 Hz), 105.6 (q, J = 30.0 Hz), 43.5 (s), 42.5 (s).

^{19}F-NMR (565 MHz, $CDCl_3$) δ: -80.8 (s).

HRMS (ESI), m/z: calc. for $C_{11}H_{11}ClF_3N_2O^+$: 279,0507 $[M+H]^+$; found: 279,0518.

3-(4-chlorophenyl)-5-(fluoromethyl)-2-methyl-5-(trifluoromethyl)-2,5-dihydro-1,2,4-oxadiazole (9)

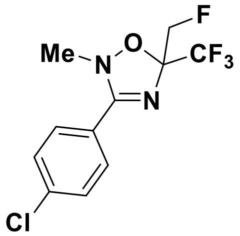

By following the general procedure 1 (GP1), starting from 3-(4-chlorophenyl)-5-(trifluoromethyl)-1,2,4-oxadiazole (248.6 mg, 1.0 mmol, 1.0 equiv), fluoroiodomethane (479.8 mg, 0.20 mL, 3.0 mmol, 3.0 equiv) and MeLi-LiBr complex (1.27 mL of a solution 2.2 M in Et_2O, 2.8 mmol, 2.8 equiv) in THF/ Et_2O 1:1 (5 mL), compound **9** was obtained in 85% yield (151.0 mg) as white solid (m.p.: 80 °C) after chromatography on silica gel (97:3 v/v, petroleum ether/ethyl acetate).

^{1}H-NMR (600 MHz, $CDCl_3$) δ: 7.71 (m, 2H), 7.46 (m, 2H), 4.75 (ddd, 1H, J=45.9, 10.1, 0.6 Hz), 4.63 (dd, 1H, J=46.9, 10.2 Hz), 3.14 (s, 3H).

^{13}C-NMR (150 MHz, $CDCl_3$) δ: 168.9 (s), 138.9 (s), 130.3 (s), 129.3 (s), 124.2 (s), 121.6 (qd, J=286.5, 4.2 Hz), 105.20 (m), 79.8 (d, J=179.5 Hz), 43.1 (s).

^{19}F-NMR (565 MHz, $CDCl_3$) δ: -80.5 (s), -239.6 (t, J=46.9 Hz).

HRMS (ESI), m/z: calc. for $C_{11}H_{10}ClF_4N_2O^+$: 297,0412 $[M+H]^+$; found: 297,0412.

5-(fluoromethyl)-2-methyl-5-(trifluoromethyl)-3-(4-(trifluoromethyl)phenyl)-2,5-dihydro-1,2,4-oxadiazole (10)

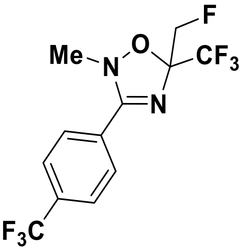

By following the general procedure 1 (GP1), starting from 5-(trifluoromethyl)-3-(4-(trifluoromethyl)phenyl)-1,2,4-oxadiazole (282.1 mg, 1.0 mmol, 1.0 equiv), fluoroiodomethane (479.8 mg, 0.20 mL, 3.0 mmol, 3.0 equiv) and MeLi-LiBr complex (1.27 mL of a solution 2.2 M in Et_2O, 2.8 mmol, 2.8 equiv) in THF/ Et_2O 1:1 (5 mL), compound **10** was obtained in 89% yield (293.9 mg) as colorless oil after chromatography on silica gel (98:2 v/v, petroleum ether/ethyl acetate).

^{1}H-NMR (600 MHz, $CDCl_3$) δ: 7.90 (d, 2H, J=8.1 Hz), 7.75 (d, 2H, J=8.3 Hz), 4.77 (dd, 1H, J=46.0, 10.2 Hz), 4.64 (dd, 1H, J=46.9, 10.2 Hz), 3.16 (s, 3H).

^{13}**C-NMR** (150 MHz, $CDCl_3$) δ: 168.6 (s), 134.3 (q, J=33.1 Hz), 129.5 (s), 129.3 (s), 129.3 (s), 128.5 (q, J = 276.0 Hz), 126.0 (q, J=11.0 Hz), 123.5 (qd, J=285.7, 4.7 Hz), 105.4 (m), 79.7 (d, J=179.5 Hz), 42.9 (s).
^{19}**F-NMR** (565 MHz, $CDCl_3$) δ: -63.1 (s), -80.5 (s), -239.8 (t, J=46.5 Hz).
HRMS (ESI), m/z: calc. for $C_{12}H_{10}F_7N_2O^+$: 331,0676 $[M+H]^+$; found: 331,0676.

4-(5-(fluoromethyl)-2-methyl-5-(trifluoromethyl)-2,5-dihydro-1,2,4-oxadiazol-3-yl)benzonitrile (11)

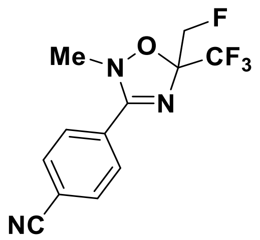

By following the general procedure 1 (GP1), starting from 4-(5-(trifluoromethyl)-1,2,4-oxadiazol-3-yl)benzonitrile (239.2 mg, 1.0 mmol, 1.0 equiv), fluoroiodomethane (479.8 mg, 0.20 mL, 3.0 mmol, 3.0 equiv) and MeLi-LiBr complex (1.27 mL of a solution 2.2 M in Et_2O, 2.8 mmol, 2.8 equiv) in THF/ Et_2O 1:1 (5 mL), compound **11** was obtained in 81% yield (249.7 mg) as yellow solid (m.p.: 88 °C) after chromatography on silica gel (90:10 v/v, petroleum ether/ethyl acetate).
^{1}**H-NMR** (600 MHz, $CDCl_3$) δ: 7.90-7.87 (m, 2H), 7.80-7.77 (m, 2H), 4.77 (dd, 1H, J=45.8, 10.3 Hz), 4.63 (dd, 1H, J=46.9, 10.2 Hz), 3.15 (s, 3H).
^{13}**C-NMR** (150 MHz, $CDCl_3$) δ: 168.1 (s), 132.6 (s), 130.0 (s), 129.6 (s), 121.4 (qd, J=286.5, 4.2 Hz), 117.6 (s), 116.1 (s), 105.5 (m), 79.6 (d, J=179.6 Hz), 42.8 (s).
^{19}**F-NMR** (565 MHz, $CDCl_3$) δ: -80.4 (s), -234.0 (t, J=46.9 Hz).
HRMS (ESI), m/z: calc. for $C_{12}H_{10}F_4N_3OH^+$: 288,0755 $[M+H]^+$; found: 288,0755.

5-(fluoromethyl)-2-methyl-3-(4-nitrophenyl)-5-(trifluoromethyl)-2,5-dihydro-1,2,4-oxadiazole (12)

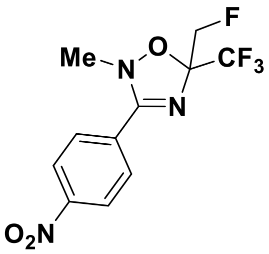

By following the general procedure 1 (GP1), starting from 3-(4-nitrophenyl)-5-(trifluoromethyl)-1,2,4-oxadiazole (259.1 mg, 1.0 mmol, 1.0 equiv), fluoroiodomethane (479.8 mg, 0.20 mL, 3.0 mmol, 3.0 equiv) and MeLi-LiBr complex (1.27 mL of a solution 2.2 M in Et_2O, 2.8 mmol, 2.8 equiv) in THF/ Et_2O 1:1 (5 mL), compound **12** was obtained in 83% yield (254.8 mg) as yellow solid (m.p.: 61 °C) after chromatography on silica gel (92:8 v/v, petroleum ether/ethyl acetate).
^{1}**H-NMR** (600 MHz, $CDCl_3$) δ: 8.36-8.33 (m, 2H), 7.98-7.95 (m, 2H), 4.78 (ddd, 1H, J=45.9, 10.2, 0.8 Hz), 4.64 (dd, 1H, J=46.9, 10.2 Hz), 3.16 (s, 3H).
^{13}**C-NMR** (150 MHz, $CDCl_3$) δ: 167.9 (s), 150.2 (s), 131.6 (s), 130.2 (s), 124.1 (s), 121.41 (qd, J = 286.7, 4.8 Hz), 105.61 (m), 79.57 (d, J = 179.7 Hz), 42.81 (s).
^{19}**F-NMR** (565 MHz, $CDCl_3$) δ -84.4 (s), -219.1 (t, J = 46.7 Hz).
HRMS (ESI), m/z: calc. for $C_{11}H_{10}F_4N_3O_3^+$: 308,0653 $[M+H]^+$; found: 308,0653.

5-(fluoromethyl)-2-methyl-3-(thiophen-2-yl)-5-(trifluoromethyl)-2,5-dihydro-1,2,4-oxadiazole (13)

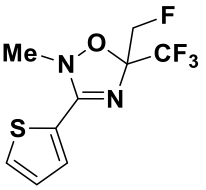

By following the general procedure 1 (GP1), starting from 3-(thiophen-2-yl)-5-(trifluoromethyl)-1,2,4-oxadiazole (220.2 mg, 1.0 mmol, 1.0 equiv), fluoroiodomethane (479.8 mg, 0.20 mL, 3.0 mmol, 3.0 equiv) and MeLi-LiBr complex (1.27 mL of a solution 2.2 M in Et_2O, 2.8 mmol, 2.8 equiv) in THF/ Et_2O 1:1 (5 mL), compound **13** was obtained in 84% yield (225.3 mg) as white solid (m.p.: 78 °C) after chromatography on silica gel (92:8 v/v, petroleum ether/ethyl acetate).

^{1}H-NMR (600 MHz, $CDCl_3$) δ: 7.60 (dd, 1H, J=5.0, 1.1 Hz), 7.57 (dd, 1H, J=3.8, 1.1 Hz), 7.15 (dd, 1H, J=5.0, 3.8 Hz), 4.78-4.58 (m, 2H), 3.28 (s, 3H).

^{13}C-NMR (150 MHz, $CDCl_3$) δ: 164.1 (s), 131.9 (s), 131.6 (s), 128.0 (s), 127.2 (s), 121.6 (qd, J=286.6, 4.0 Hz), 104.8 (m), 79.8 (d, J=179.8 Hz), 44.1 (s).

^{19}F-NMR (565 MHz, $CDCl_3$) δ: -80.4 (s), -239.2 (t, J=46.8 Hz).

HRMS (ESI), m/z: calc. for $C_9H_9F_4N_2OS^+$: 269,0366 $[M+H]^+$; found: 269,0368.

3-(4-fluorophenyl)-5-(iodomethyl)-4-methyl-5-(trifluoromethyl)-4,5-dihydro-1,2,4-oxadiazole (14)

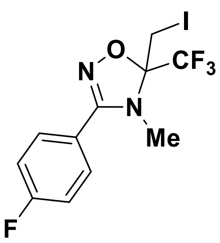

By following the general procedure 1 (GP1), starting from 3-(4-fluorophenyl)-5-(trifluoromethyl)-1,2,4-oxadiazole (232.1 mg, 1.0 mmol, 1.0 equiv), diiodomethane (903.5 mg, 0.24 mL, 3.0 mmol, 3.0 equiv) and MeLi-LiBr complex (1.27 mL of a solution 2.2 M in Et_2O, 2.8 mmol, 2.8 equiv) in THF (5 mL), compound **14** was obtained in 91% yield (353.2 mg) as yellow solid (m.p.: 62 °C) after chromatography on silica gel (97:3 v/v, petroleum ether/ethyl acetate).

^{1}H-NMR (600 MHz, $CDCl_3$) δ: 7.78 – 7.75 (m, 2H), 7.20 – 7.15 (m, 2H), 3.84 (d, 1H, J = 11.5 Hz), 3.60 (d, 1H, J = 11.5 Hz), 3.23 (s, 3H).

^{13}C-NMR (150 MHz, $CDCl_3$) δ: 168.1 (s), 165.1 (d, J = 253.8 Hz), 131.2 (d, J = 8.8 Hz), 122.1 (d, J = 2.7 Hz), 119.8 (q, J = 288.9 Hz), 116.3 (d, J = 21.9 Hz), 104.8 (q, J = 30.2 Hz), 42.3 (s), 7.9 (s).

^{19}F-NMR (565 MHz, $CDCl_3$) δ: -80.6 (s), -105.7 (s).

HRMS (ESI), m/z: calc. for $C_{11}H_{10}F_4IN_2O^+$: 388,9768 $[M+H]^+$; found: 388,9781.

5-(iodomethyl)-4-methyl-3-phenyl-5-(trifluoromethyl)-4,5-dihydro-1,2,4-oxadiazole (15)

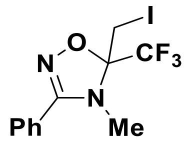

By following the general procedure 1 (GP1), starting from 3-(phenyl)-5-(trifluoromethyl)-1,2,4-oxadiazole (214.2 mg, 1.0 mmol, 1.0 equiv), diiodomethane (903.5 mg, 0.24 mL, 3.0 mmol, 3.0 equiv) and MeLi-LiBr complex (1.27 mL of a solution 2.2 M in Et_2O, 2.8 mmol, 2.8 equiv) in THF (5 mL), compound **15** was obtained

in 85% yield (314.6 mg) as white solid (m.p.: 77 °C) after chromatography on silica gel (98:2 v/v, petroleum ether/ethyl acetate).

^{1}H-NMR (600 MHz, $CDCl_3$) δ: 7.75 (dd, 2H, J = 8.3, 1.4 Hz), 7.60 - 7.54 (m, 1H), 7.51 - 7.46 (m, 2H), 3.86 (d, 1H, J = 11.4 Hz), 3.61 (d, 1H, J = 11.4 Hz), 3.25 (s, 3H).

^{13}C-NMR (150 MHz, $CDCl_3$) δ: 169.1 (s), 132.4 (s), 128.9 (s), 128.9 (s), 125.9 (s), 119.8 (q, J = 289.0 Hz), 104.75 (q, J = 30.4 Hz), 42.2 (s), 8.03 (s).

^{19}F-NMR (565 MHz, $CDCl_3$) δ: -80.6 (s).

HRMS (ESI), m/z: calc. for $C_{11}H_{11}F_3IN_2O^+$: 370,9863 $[M+H]^+$; found: 370,9876.

4-(5-(iodomethyl)-4-methyl-5-(trifluoromethyl)-4,5-dihydro-1,2,4-oxadiazol-3-yl)-*N*,*N*-dimethylaniline (16)

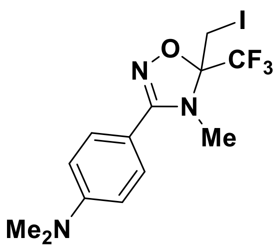

By following the general procedure 1 (GP1), starting from *N*,*N*-dimethyl-4-(5-(trifluoromethyl)-1,2,4-oxadiazol-3-yl)aniline (214.2 mg, 1.0 mmol, 1.0 equiv), diiodomethane (903.5 mg, 0.24 mL, 3.0 mmol, 3.0 equiv) and MeLi-LiBr complex (1.27 mL of a solution 2.2 M in Et_2O, 2.8 mmol, 2.8 equiv) in THF (5 mL), compound **16** was obtained in 89% yield (367.7 mg) as yellow solid (m.p.: 112 °C) after chromatography on silica gel (90:10 v/v, petroleum ether/ethyl acetate).

^{1}H-NMR (600 MHz, $CDCl_3$) δ: 7.71 – 7.54 (m, 2H), 6.73 – 6.64 (m, 2H), 3.83 (d, 1H, J = 11.4 Hz), 3.60 (d, 1H, J = 11.4 Hz), 3.25 (s, 3H), 3.03 (s, 3H).

^{13}C-NMR (150 MHz, $CDCl_3$) δ: 169.3 (s), 152.8 (s), 130.4 (s), 119.9 (q, J = 289.3 Hz), 112.0 (s), 111.2 (s), 104.3 (q, J = 29.7 Hz), 43.22 (s), 40.0 (s), 8.4 (s).

^{19}F-NMR (565 MHz, $CDCl_3$) δ: -80.5 (s).

HRMS (ESI), m/z: calc. for $C_{13}H_{16}F_3IN_3O^+$: 414,0285 $[M+H]^+$; found: 414,0288.

5-(iodomethyl)-3-(4-methoxyphenyl)-4-methyl-5-(trifluoromethyl)-4,5-dihydro-1,2,4-oxadiazole (17)

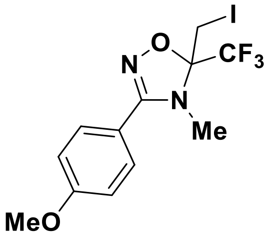

By following the general procedure 1 (GP1), starting from 3-(4-methoxyphenyl)-5-(trifluoromethyl)-1,2,4-oxadiazole (244.1 mg, 1.0 mmol, 1.0 equiv), diiodomethane (903.5 mg, 0.24 mL, 3.0 mmol, 3.0 equiv) and MeLi-LiBr complex (1.27 mL of a solution 2.2 M in Et_2O, 2.8 mmol, 2.8 equiv) in THF (5 mL), compound **17** was obtained in 87% yield (348.1 mg) as white solid (m.p.: 98 °C) after chromatography on silica gel (97:3 v/v, petroleum ether/ethyl acetate).

^{1}H-NMR (600 MHz, $CDCl_3$) δ: 7.71 (d, 2H, J = 8.7 Hz), 6.97 (d, 2H, J = 8.8 Hz), 3.86 (s, 3H), 3.83 (d, 1H, J = 11.4 Hz), 3.60 (d, 1H, J = 11.4 Hz), 3.24 (s, 3H).

^{13}C-NMR (150 MHz, $CDCl_3$) δ: 168.8 (s), 162.8 (s), 130.7 (s), 119.8 (q, J = 288.9 Hz), 118.0 (s), 114.3 (s), 104.6 (q, J = 29.8 Hz), 55.5 (s), 42.7 (s), 8.2 (s).

^{19}F-NMR (565 MHz, $CDCl_3$) δ: -80.6 (s).

HRMS (ESI), m/z: calc. for $C_{12}H_{13}F_3IN_2O_2^+$: 400,9968 $[M+H]^+$; found: 400,9978.

5-(bromomethyl)-2-methyl-3-(3-methylphenyl)-5-(trifluoromethyl)-2,5-dihydro-1,2,4-oxadiazole (18) and 5-(bromomethyl)-4-methyl-3-(3-methylphenyl)-5-(trifluoromethyl)-4,5-dihydro-1,2,4-oxadiazole (18a)

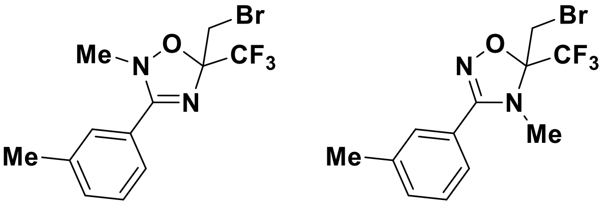

By following the general procedure 1 (GP1), starting from 3-(3-methylphenyl)-5-(trifluoromethyl)-1,2,4-oxadiazole (228.1 mg, 1.0 mmol, 1.0 equiv), bromoiodomethane (662.5 mg, 0.22 mL, 3.0 mmol, 3.0 equiv) and MeLi-LiBr complex (1.27 mL of a solution 2.2 M in Et_2O, 2.8 mmol, 2.8 equiv) in THF (5 mL), compounds **18-18a** was obtained in 86% yield (289.9 mg) as white solid (m.p.: 57-73°C) after chromatography on silica gel (97:3 v/v, petroleum ether/ethyl acetate). Ratio 1:0.8

^{1}H-NMR (600 MHz, $CDCl_3$) δ: 7.59 (br s, 1.7 H in total), 7.53 – 7.49 (m, 1.7 H in total), 7.38 – 7.33 (m, 3.4 H in total), 3.93 (d, 0.7 H, J = 11.5 Hz), 3.85 (d, 1 H, J = 11.4 Hz), 3.71 (d, 0.7 H, J = 11.6 Hz), 3.61 (d, 1 H, J = 11.5 Hz), 3.24 (s, 3 H), 3.21 (s, 2.3 H), 2.41 (br s, 5.4 H in total).

^{13}C-NMR (150 MHz, $CDCl_3$) δ: 169.6 (s), 169.2 (s), 139.0 (s), 133.2 (s), 133.2 (s), 129.4 (s), 129.3 (s), 128.8 (s), 125.9 (s), 125.9 (s), 125.8 (s), 125.8 (s), 120.6 (m), 104.82 (m), 42.4 (s), 42.2 (s), 32.4 (s), 21.2 (s), 8.0 (s).

^{19}F-NMR (565 MHz, $CDCl_3$) δ: -80.7 (s), -80.7 (s).

HRMS (ESI), m/z: calc. for $C_{12}H_{13}BrF_3N_2O^+$: 337,0158 $[M+H]^+$; found: 337,0159.

5-(bromomethyl)-3-(4-chlorophenyl)-2-methyl-5-(trifluoromethyl)-2,5-dihydro-1,2,4-oxadiazole (19) and 5-(bromomethyl)-3-(4-chlorophenyl)-4-methyl-5-(trifluoromethyl)-4,5-dihydro-1,2,4-oxadiazole (19a)

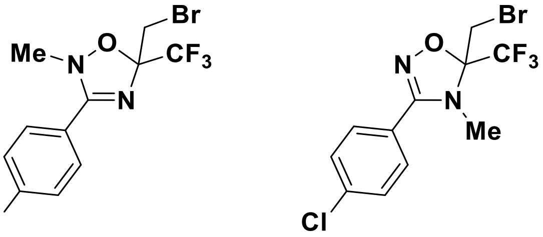

By following the general procedure 1 (GP1), starting from 3-(4-chlorophenyl)-5-(trifluoromethyl)-1,2,4-oxadiazole (248.6 mg, 1.0 mmol, 1.0 equiv), bromoiodomethane (662.5 mg, 0.22 mL, 3.0 mmol, 3.0 equiv) and MeLi-LiBr complex (1.27 mL of a solution 2.2 M in Et_2O, 2.8 mmol, 2.8 equiv) in THF (5 mL), compound **19-19a** was obtained in 87% yield (311.1 mg) as yellow solid (m.p.: 61-71 °C) after chromatography on silica gel (98:2 v/v, petroleum ether/ethyl acetate). Ratio 1:0.9

^{1}H-NMR (600 MHz, $CDCl_3$) δ: 7.72 – 7.68 (m, 3.8 H in total), 7.48 – 7.44 (m, 3.8 H in total), 3.92 (d, 0.9 H, J = 10.8 Hz), 3.83 (d, 1 H, J = 11.8 Hz), 3.70 (d, 0.9 H, J = 11.6 Hz), 3.60 (d, 1 H, J = 11.6 Hz), 3.23 (s, 3 H), 3.19 (s, 2.9 H).

^{13}C-NMR (150 MHz, $CDCl_3$) δ: 168.4 (s), 168.1 (s), 138.8 (s), 138.8 (s), 130.2 (s), 130.2 (s), 129.3 (s), 124.4 (s), 124.3 (s), 120.0 (m), 105.0 (m), 42.4 (s), 42.2 (s), 32.3 (s), 7.8 (s).

^{19}F-NMR (565 MHz, $CDCl_3$) δ: -80.6 (s), -80.7 (s).

HRMS (ESI), m/z: calc. for $C_{11}H_{10}BrClF_3N_2O^+$: 356,9612 $[M+H]^+$; found: 356,9614.

4-(5-(bromomethyl)-2-methyl-5-(trifluoromethyl)-2,5-dihydro-1,2,4-oxadiazol-3-yl)benzonitrile (20) and 4-(5-(bromomethyl)-4-methyl-5-(trifluoromethyl)-4,5-dihydro-1,2,4-oxadiazol-3-yl)benzonitrile (20a)

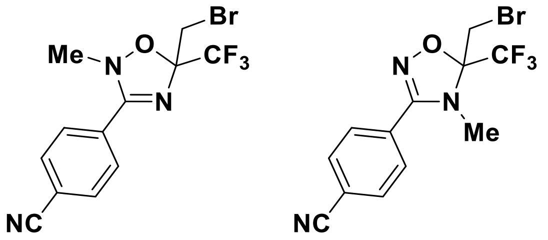

By following the general procedure 1 (GP1), starting from 4-(5-(trifluoromethyl)-1,2,4-oxadiazol-3-yl)benzonitrile (239.2 mg, 1.0 mmol, 1.0 equiv), bromoiodomethane (662.5 mg, 0.22 mL, 3.0 mmol, 3.0 equiv) and MeLi-LiBr complex (1.27 mL of a solution 2.2 M in Et_2O, 2.8 mmol, 2.8 equiv) in THF (5 mL), compound **20-20a** was obtained in 83% yield (288.9 mg) as yellow solid (m.p.: 110-116 °C) after chromatography on silica gel (90:10 v/v, petroleum ether/ethyl acetate). Ratio 1:1.5

^{1}H-NMR (600 MHz, $CDCl_3$) δ: 7.89 – 7.86 (m, 3.6 H in total), 7.79 (m, 3.6 H in total), 3.92 (d, 1 H, J = 11.6 Hz), 3.84 (d, 0.8 H, J = 11.6 Hz), 3.71 (d, 1 H, J = 11.6 Hz), 3.60 (d, 0.8 H, J = 11.5 Hz), 3.24 (s, 2.4 H), 3.20 (s, 3 H).

^{13}C-NMR (150 MHz, $CDCl_3$) δ: 167.6 (s), 167.3 (s), 132.7 (s), 130.2 (s), 130.1 (s), 129.5 (s), 129.5 (s), 121.3 (q, J = 288.8 Hz), 117.6 (s), 116.1 (s), 116.1 (s), 105.5 (q, J = 30.4 Hz), 42.1 (s), 41.9 (s), 32.1 (s), 7.5 (s).

^{19}F-NMR (565 MHz, $CDCl_3$) δ: -80.6 (s).

HRMS (ESI), m/z: calc. for $C_{12}H_{10}BrF_3N_3O^+$: 347,9954 $[M+H]^+$; found: 347,9956.

5-(bromomethyl)-2-methyl-3-(4-nitrophenyl)-5-(trifluoromethyl)-2,5-dihydro-1,2,4-oxadiazole (21) and 5-(bromomethyl)-4-methyl-3-(4-nitrophenyl)-5-(trifluoromethyl)-4,5-dihydro-1,2,4-oxadiazole (21a)

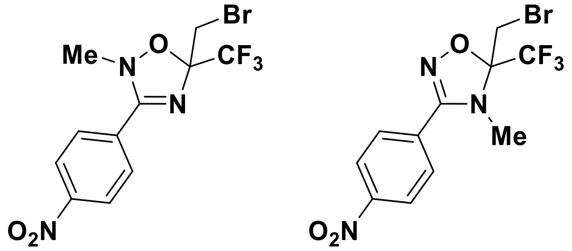

By following the general procedure 1 (GP1), starting from 3-(4-nitrophenyl)-5-(trifluoromethyl)-1,2,4-oxadiazole (259.1 mg, 1.0 mmol, 1.0 equiv), bromoiodomethane (662.5 mg, 0.22 mL, 3.0 mmol, 3.0 equiv) and MeLi-LiBr complex (1.27 mL of a solution 2.2 M in Et_2O, 2.8 mmol, 2.8 equiv) in THF (5 mL), compound **21-21a** was obtained in 82% yield (301.8 mg) as orange solid (m.p.: 84-91 °C) after chromatography on silica gel (92:8 v/v, petroleum ether/ethyl acetate). Ratio 1:1.5

^{1}H-NMR (600 MHz, $CDCl_3$) δ: 8.36 – 8.32 (m, 3.2 H in total), 7.97 – 7.94 (m, 3.2 H in total), 3.94 (dd, 1H, J = 11.6, 0.5 Hz), 3.86 (dd, 0.7 H, J = 11.6, 0.5 Hz), 3.72 (d, 1H, J = 11.4 Hz), 3.62 (d, 0.7 H, J = 11.6 Hz), 3.25 (s, 2.1 H), 3.22 (s, 3H).

^{13}C-NMR (150 MHz, $CDCl_3$) δ: 167.3 (s), 167.0 (s), 150.1 (s), 131.8 (s), 131.7 (s), 130.1 (s), 130.0 (s), 124.1 (s), 120.4 (m), 106.5 – 104.0 (m), 42.1 (s), 41.9 (s), 32.1 (s), 7.5 (s).

^{19}F-NMR (565 MHz, $CDCl_3$) δ: -80.6 (s), -80.6 (s).

HRMS (ESI), m/z: calc. for $C_{11}H_{10}BrF_3N_3O_3^+$: 367,9852 $[M+H]^+$; found: 367,9850.

5-(bromomethyl)-2-methyl-3-(pyridin-2-yl)-5-(trifluoromethyl)-2,5-dihydro-1,2,4-oxadiazole (22) and 5-(bromomethyl)-4-methyl-3-(pyridin-2-yl)-5-(trifluoromethyl)-4,5-dihydro-1,2,4-oxadiazole (22a)

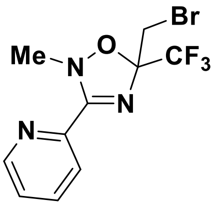

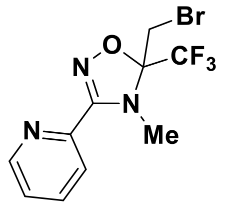

By following the general procedure 1 (GP1), starting from 3-(pyridin-2-yl)-5-(trifluoromethyl)-1,2,4-oxadiazole (215.1 mg, 1.0 mmol, 1.0 equiv), bromoiodomethane (662.5 mg, 0.22 mL, 3.0 mmol, 3.0 equiv) and MeLi-LiBr complex (1.27 mL of a solution 2.2 M in Et_2O, 2.8 mmol, 2.8 equiv) in THF (5 mL), compound **22-22a** was obtained in 84% yield (272.2 mg) as yellow oi after chromatography on silica gel (87:13 v/v, petroleum ether/ethyl acetate). Ratio 1:4

^{1}H-NMR (600 MHz, $CDCl_3$) δ: 8.69 (d, 1.2 H in total, J = 4.7 Hz), 8.16 (d, 1.2 H in total, J = 7.9 Hz), 7.87 – 7.80 (m, 1.2 H in total), 7.48 – 7.43 (m, 1.2 H in total), 3.91 (d, 1 H, J = 11.6 Hz), 3.83 (d, 0.2 H, J = 11.4 Hz), 3.72 (d, 1 H, J = 11.6 Hz), 3.62 (d, 0.2 H, J = 11.5 Hz), 3.58 (s, 0.7 H), 3.55 (s, 3 H).

^{13}C-NMR (150 MHz, $CDCl_3$) δ: 166.3 (s), 165.9 (s), 149.3 (s), 145.7 (s), 145.6 (s), 137.0 (s), 126.3 (s), 125.3 (s), 121.5 (q, J = 288.9 Hz), 104.5 (q, J = 30.4 Hz), 42.0 (s), 41.8 (s), 32.3 (s).

^{19}F-NMR (565 MHz, $CDCl_3$) δ: -80.7 (s).

HRMS (ESI), m/z: calc. for $C_{10}H_{10}BrF_3N_3O^+$: 323,9954 $[M+H]^+$; found: 323,9972.

5-(bromomethyl)-2-methyl-3-(thiophen-2-yl)-5-(trifluoromethyl)-2,5-dihydro-1,2,4-oxadiazole (23) and 5-(bromomethyl)-4-methyl-3-(thiophen-2-yl)-5-(trifluoromethyl)-4,5-dihydro-1,2,4-oxadiazole (23a)

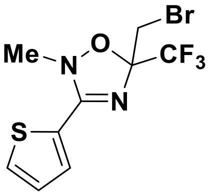

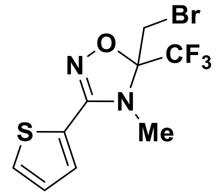

By following the general procedure 1 (GP1), starting from 3-(thiophen-2-yl)-5-(trifluoromethyl)-1,2,4-oxadiazole (220.2 mg, 1.0 mmol, 1.0 equiv), bromoiodomethane (662.5 mg, 0.22 mL, 3.0 mmol, 3.0 equiv) and MeLi-LiBr complex (1.27 mL of a solution 2.2 M in Et_2O, 2.8 mmol, 2.8 equiv) in THF (5 mL), compound **23-23a** was obtained in 89% yield (292.9 mg) as white solid (m.p.: 65-69 °C) after chromatography on silica gel (95:5 v/v, petroleum ether/ethyl acetate). Ratio 1:0.9

^{1}H-NMR (600 MHz, $CDCl_3$) δ: 7.60 (m, 1.7 in total), 7.57 – 7.55 (m, 1.7 in total), 7.17 – 7.13 (m, 1.7 in total), 3.90 (d, 0.7 H, J = 11.6 Hz), 3.82 (d, 1 H, J = 11.5 Hz), 3.70 (d, 0.7 H, J = 11.6 Hz), 3.60 (d, 1 H J = 11.5 Hz), 3.37 (s, 3 H), 3.34 (s, 2.1 H).

^{13}C-NMR (150 MHz, $CDCl_3$) δ: 163.6 (s), 163.3 (s), 131.7 (s), 131.6 (s), 131.5 (s), 131.4 (s), 128.0 (s), 127.1 (s), 127.0 (s), 120.6 (m), 104.7 (m), 43.4 (s), 43.3 (s), 32.1 (s), 7.6 (s).

^{19}F-NMR (565 MHz, $CDCl_3$) δ: -80.5 (s), -80.5 (s).

HRMS (ESI), m/z: calc. for $C_9H_9BrF_3N_2OS^+$: 328,9566 $[M+H]^+$; found: 328,9566.

3-(4-bromophenyl)-5-(iodomethyl)-4-(methyl-d3)-5-(trifluoromethyl)-4,5-dihydro-1,2,4-oxadiazole (24)

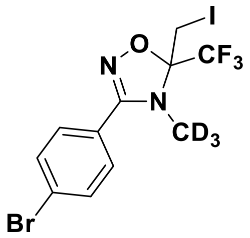

By following the general procedure 1 (GP1), starting from 3-(4-bromophenyl)-5-(trifluoromethyl)-1,2,4-oxadiazole (293.4 mg, 1.0 mmol, 1.0 equiv), diiodomethane (903.5 mg, 0.24 mL, 3.0 mmol, 3.0 equiv) and CD_3Li-LiI complex (5.6 mL of a solution 0.5 M in Et_2O, 2.8 mmol, 2.8 equiv) in THF (5 mL), compound **24** was obtained in 85% yield (384.2 mg) as yellow solid (m.p.: 70 °C) after chromatography on silica gel (97:3 v/v, petroleum ether/ethyl acetate).

^{1}H-NMR (600 MHz, $CDCl_3$) δ: 7.63 (br s, 4H), 3.84 (d, 1H, J = 11.4 Hz), 3.60 (d, 1H, J = 11.5 Hz), 3.23 (br, residual non-deuterated -CH_3).

^{13}C-NMR (150 MHz, $CDCl_3$) δ: 168.2 (s), 132.3 (s), 130.3 (s), 127.2 (s), 124.8 (s), 119.7 (q, J = 288.8 Hz), 104.9 (q, J = 30.4 Hz), 42.2 (br, CD_3), 7.8 (s).

^{19}F-NMR (565 MHz, $CDCl_3$) δ: -80.6 (s).

HRMS (ESI), m/z: calc. for $C_{11}H_7D_3BrF_3IN_2O^+$: 451,9156 $[M+H]^+$; found: 451,9157.

3-(pyridin-4-yl)-5-(trifluoromethyl)-5-((trimethylsilyl)methyl)-2,5-dihydro-1,2,4-oxadiazole (25)

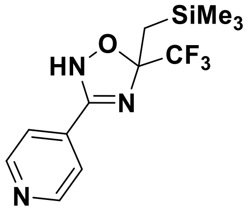

By following the general procedure 4 (GP4), starting from 3-(pyridin-4-yl)-5-(trifluoromethyl)-1,2,4-oxadiazole (215.1 mg, 1.0 mmol, 1.0 equiv), and (trimethylsilyl)methyllithium solution (1.1 mL of a solution 1.0 M in pentane, 1.1 mmol, 1.1 equiv) in THF (5 mL), compound **25** was obtained in 87% yield (264.3 mg) as yellow solid (m.p.: 89°C) after chromatography on silica gel (8:2 v/v, petroleum ether/ethyl acetate).

^{1}H-NMR (600 MHz, $CDCl_3$) δ: 8.57 (d, 2H, J = 5.9 Hz), 7.55 (dd, 2H, J = 4.5, 1.7 Hz), 6.92 (br, 1H), 1.54 (d, 1H, J = 15.4 Hz), 1.34 (d, 1H, J = 15.4 Hz), 0.09 (s, 9H).

^{13}C-NMR (150 MHz, $CDCl_3$) δ: 153.9 (s), 15.0 (s), 132.6 (s), 123.2 (Q, J = 289.8 Hz), 120.7 (s), 97.8 (q, J = 32.2 Hz), 21.3 (s), -0.7 (s).

^{19}F-NMR (565 MHz, $CDCl_3$) δ: -87.0 (s).

HRMS (ESI), m/z: calc. for $C_{12}H_{17}F_3N_3OSi^+$: 304,1087 $[M+H]^+$; found: 304,1088.

3-(4-methoxyphenyl)-5-methyl-5-(trifluoromethyl)-2,5-dihydro-1,2,4-oxadiazole (26)

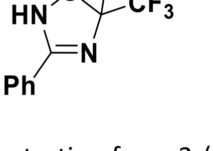

By following the general procedure 4 (GP4), starting from 3-(phenyl)-5-(trifluoromethyl)-1,2,4-oxadiazole (214.2 mg, 1.0 mmol, 1.0 equiv), and (trimethylsilyl)methyllithium solution (1.1 mL of a solution 1.0 M in pentane, 1.1 mmol, 1.1 equiv), compound **26** was obtained in 83% yield (251.0 mg) as white solid (m.p.: 141°C) after chromatography on silica gel (9:1 v/v, petroleum ether/ethyl acetate).

^{1}H-NMR (600 MHz, $CDCl_3$) δ: 7.66 - 7.63 (m, 2H), 7.47 (t, 1H, J = 7.5 Hz), 7.39 (t, 2H, J = 7.7 Hz), 5.21 (br, 1H), 1.57 (d, 1H, J = 15.4 Hz), 1.38 (d, 1H, J = 15.4 Hz), 0.13 (s, 9H).
^{13}C-NMR (150 MHz, $CDCl_3$) δ: 155.3 (s), 131.4 (s), 128.9 (s), 126.6 (s), 124.2 (s), 123.3 (q, J = 289.9 Hz), 96.7 (q, J = 32.2 Hz), 21.4 (s), -0.6 (s).
^{19}F-NMR (565 MHz, $CDCl_3$) δ: -86.9 (s).
HRMS (ESI), m/z: calc. for $C_{13}H_{17}F_3N_2OSi^+$: 303,1135 $[M+H]^+$; found: 303,1137.

2-(3-phenyl-5-(trifluoromethyl)-2,5-dihydro-1,2,4-oxadiazol-5-yl)acetonitrile (27)

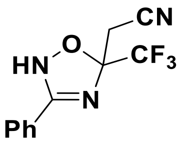

To a solution of dry acetonitrile (82.1 mg, 0.10 mL, 2.0 mmol, 2.0 equiv) in anhydrous THF cooled at -78 °C, MeLi-LiBr (0.68 mL of a solution 2.2 M in THF, 1.5 mmol, 1.5 equiv) was added dropwise during 5 min and the resulting mixture was stirred for 30 min. Then, a solution of 3-(phenyl)-5-(trifluoromethyl)-1,2,4-oxadiazole (214.2 mg, 1.0 mmol, 1.0 equiv) in THF (3 mL) was added and the stirring was continued for additional 1.5 hour at -78 °C. A solution of saturated aqueous NH_4Cl was added and after removing of the cooling-bath the system was allowed to reach rt. The resulting mixture was extracted 3 times with Et_2O, the organic phase was washed with brine, dried over anhydrous Na_2SO_4 and concentrated in vacuo. Compound **27** was obtained in 92% yield (234.8 mg) as white solid (m.p.: 175 °C) after chromatography on silica gel (85:15 v/v, petroleum ether/ethyl acetate).
^{1}H-NMR (400 MHz, Acetone-d_6) δ: 8.13 (br, 1H), 7.82 - 7.75 (m, 2H), 7.59 - 7.46 (m, 3H), 3.49 (s, 2H).
^{13}C-NMR (100 MHz, Acetone-d_6) δ: 156.5 (s), 132.4 (s), 129.8 (s), 127.5 (s), 124.5 (s), 123.2 (q, J = 288.3 Hz), 114.7 (s), 95.0 (q, J = 33.1 Hz), 24.2 (s).
^{19}F-NMR (377 MHz, Acetone-d_6) δ: -85.0 (s).
HRMS (ESI), m/z: calc. for $C_{13}H_{17}F_3N_2O^+$: 303,1135 $[M+H]^+$; found: 303,1137.

5-(methoxymethyl)-3-phenyl-5-(trifluoromethyl)-2,5-dihydro-1,2,4-oxadiazole (28)

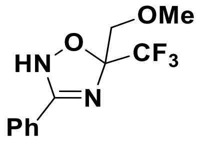

Lithium metal (48.6 mg, 7.0 mmol, 7.0 equiv) was suspended in dry THF and DTBB (13.3 mg, 0.05 mml, 0.05 equiv) was added and the mixture was stirred until the appearance of the dark green colour. This suspension was cooled down to -78 °C and then $ClCH_2OCH_3$ (161.0 mg, 0.15 mL, 2.0 mmol, 2.0 equiv) was added dropwise. A 1.0 M solution of 3-(phenyl)-5-(trifluoromethyl)-1,2,4-oxadiazole (214.2 mg, 1.0 mmol, 1.0 equiv) in dry THF (2 mL) was added and the mixture was stirred at -78 °C for 3 hours. A solution of saturated aqueous NH_4Cl was added and after removing of the cooling-bath the system was allowed to reach rt. The resulting mixture was extracted 3 times with Et_2O, the organic phase was washed with brine, dried over anhydrous Na_2SO_4 and concentrated in vacuo. Compound **28** was obtained in 89% yield (231.6 mg) as white solid (m.p.: 133 °C) after chromatography on silica gel (88:12 v/v, petroleum ether/ethyl acetate).
^{1}H-NMR (400 MHz, Acetone-d_6) δ: 7.82 – 7.76 (m, 2H), 7.57 – 7.45 (m, 3H), 7.38 (br, 1H), 3.89 (d, J = 11.4 Hz, 1H), 3.78 (d, J = 11.4 Hz, 1H), 3.43 (s, 3H).
^{13}C-NMR (100 MHz, Acetone-d_6) δ: δ 156.6 (s), 132.0 (s), 129.7 (s), 127.6 (s), 125.7 (s), 124.0 (q, J = 288.2 Hz), 97.2 (q, J = 30.9 Hz), 70.5 (s), 60.1 (s).
^{19}F-NMR (377 MHz, Acetone-d_6) δ: -84.2 (s).
HRMS (ESI), m/z: calc. for $C_{11}H_{11}F_3N_2O_2^+$: 261,0845 $[M+H]^+$; found: 261,0845.

5-((methylthio)methyl)-3-phenyl-5-(trifluoromethyl)-2,5-dihydro-1,2,4-oxadiazole (29)

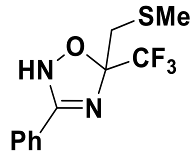

Lithium metal (48.6 mg, 7.0 mmol, 7.0 equiv) was suspended in dry THF and DTBB (13.3 mg, 0.05 mml, 0.05 equiv) was added and the mixture was stirred until the appearance of the dark green colour. This suspension was cooled down to -78 °C and then $ClCH_2SCH_3$ (197.2 mg, 0.17 mL, 2.0 mmol, 2.0 equiv) was added dropwise and the solution was stirred for 10 min. A 1.0 M solution of 3-(phenyl)-5-(trifluoromethyl)-1,2,4-oxadiazole (214.2 mg, 1.0 mmol, 1.0 equiv) in dry THF (2 mL) was added and the mixture was stirred at -78 °C for 3 hours. A solution of saturated aqueous NH_4Cl was added and after removing of the cooling-bath the system was allowed to reach rt. The resulting mixture was extracted 3 times with Et_2O, the organic phase was washed with brine, dried over anhydrous Na_2SO_4 and concentrated in vacuo. Compound **29** was obtained in 85% yield (234.9 mg) as white solid (m.p.: 177 °C) after chromatography on silica gel (9:1 v/v, petroleum ether/ethyl acetate).
^{1}H-NMR (400 MHz, Acetone-*d*$_6$) δ: 7.78 (m, 2H), 7.52 (m, 3H), 7.43 (br, 1H), 3.21 (d, J = 15.2 Hz, 1H), 3.10 (d, J = 15.1 Hz, 1H), 2.27 (s, 3H).
^{13}C-NMR (100 MHz, Acetone-*d*$_6$) δ: 156.8 (s), 132.0 (s), 129.6 (s), 127.4 (s), 125.4 (s), 124.1 (q, J = 288.9 Hz), 98.5 (q, J = 30.7 Hz), 36.6 (s), 17.5 (s).
^{19}F-NMR (377 MHz, Acetone-*d*$_6$) δ: -84.7 (s).
HRMS (ESI), m/z: calc. for $C_{11}H_{11}F_3N_2OS^+$: 277,0617 $[M+H]^+$; found: 277,0617.

3-phenyl-5-((phenylselanyl)methyl)-5-(trifluoromethyl)-2,5-dihydro-1,2,4-oxadiazole (30)

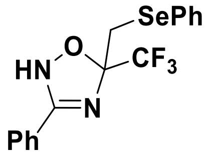

To a solution of 1,1′-(methylenediselanyl)dibenzene (0.212 g, 0.65 mmol, 1.3 equiv) in dry Et_2O (2 mL) at -78°C under argon was added dropwise *n*-BuLi (0.5 mL of a solution 2.5 M in hexane, 1.25 mmol, 1.25 equiv) and the reaction mixture was stirred at -78°C for 1 hour. Afterwards, a solution of 3-(phenyl)-5-(trifluoromethyl)-1,2,4-oxadiazole (214.2 mg, 1.0 mmol, 1.0 equiv) in dry Et_2O (2 mL) was added and the mixture was stirred at -78 °C for 1 hour. A solution of saturated aqueous NH_4Cl was added and after removing of the cooling-bath the system was allowed to reach rt. The resulting mixture was extracted 3 times with Et_2O, the organic phase was washed with brine, dried over anhydrous Na_2SO_4 and concentrated in vacuo. Compound **30** was obtained in 91% yield (350.6 mg) as white solid (m.p.: 121 °C) after chromatography on silica gel (9:1 v/v, petroleum ether/ethyl acetate).
^{1}H-NMR (400 MHz, Acetone-*d*$_6$) δ: 7.78 – 7.73 (m, 2H), 7.68 – 7.59 (m, 2H), 7.59 – 7.46 (m, 4H), 7.32 – 7.26 (m, 3H), 3.65 (d, J = 13.9 Hz, 1H), 3.58 (d, J = 13.9 Hz, 1H).
^{13}C-NMR (100 MHz, Acetone-*d*$_6$) δ: 156.5 (s), 133.8 (s), 132.1 (s), 130.7 (s), 130.0 (s), 129.7 (s), 128.2 (s), 127.5 (s), 125.4 (s), 123.9 (q, J = 289.2 Hz), 97.4 (q, J = 31.2 Hz), 31.3.
^{19}F-NMR (377 MHz, Acetone-*d*$_6$) δ: -84.4 (s).
HRMS (ESI), m/z: calc. for $C_{16}H_{14}F_3N_2OSe^+$: 387,0218 $[M+H]^+$; found: 387,0214.

3-phenyl-5,5-bis(trifluoromethyl)-2,5-dihydro-1,2,4-oxadiazole (31)

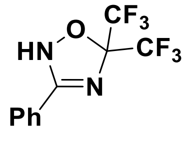

To a solution of 3-(phenyl)-5-(trifluoromethyl)-1,2,4-oxadiazole (214.2 mg, 1.0 mmol, 1.0 equiv) in dry THF (5 mL) cooled at 0°C (trifluoromethyl)trimethylsilane (284.4 mg, 0.29 mL, 2.0 mmol, 2.0 equiv) was added under Argon atmosphere. Then potassium tert-pentoxide (0.9 mL of a solution 2.0 M in THF, 1.8 mmol, 1.8 equiv) was added dropwise at 0 °C over 15 min and the reaction mixture was further stirred for additional 4 hours at the same temperature. A solution of saturated aqueous NH_4Cl was added and the resulting mixture was extracted 3 times with Et_2O, the organic phase was washed with brine, dried over anhydrous Na_2SO_4 and concentrated in vacuo. Compound **31** was obtained in 88% yield (250.1 mg) as white solid (m.p.: 129 °C) after chromatography on silica gel (9:1 v/v, petroleum ether/ethyl acetate).

^{1}H-NMR (400 MHz, Acetone-d_6) δ: 9.64 (br, 1H), 7.95 – 7.88 (m, 2H), 7.67 (t, J = 7.4 Hz, 1H), 7.56 (t, J = 7.8 Hz, 2H).

^{13}C-NMR (100 MHz, Acetone-d_6) δ: 158.7 (s), 134.0 (s), 132.7 (s), 129.7 (s), 128.7 (s), 121.7 (q, J = 289.2 Hz), 86.9 (br).

^{19}F-NMR (377 MHz, Acetone-d_6) δ: -79.7 (s), -80.6 (s)

HRMS (ESI), m/z: calc. for $C_{10}H_7F_6N_2O^+$: 285,0457 $[M+H]^+$; found: 285,0456.

5-(difluoromethyl)-3-phenyl-5-(trifluoromethyl)-2,5-dihydro-1,2,4-oxadiazole (32)

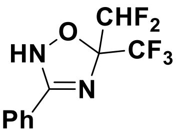

To a solution of 3-(phenyl)-5-(trifluoromethyl)-1,2,4-oxadiazole (214.2 mg, 1.0 mmol, 1.0 equiv) in dry THF (5 mL) cooled at 0°C (difluoromethyl)trimethylsilane (248.4 mg, 0.28 mL, 2.0 mmol, 2.0 equiv) was added under Argon atmosphere. Then potassium tert-pentoxide (0.9 mL of a solution 2.0 M in THF, 1.8 mmol, 1.8 equiv) was added dropwise at 0 °C over 15 min and the reaction mixture was further stirred for additional 4 hours at the same temperature. A solution of saturated aqueous NH_4Cl was added and the resulting mixture was extracted 3 times with Et_2O, the organic phase was washed with brine, dried over anhydrous Na_2SO_4 and concentrated in vacuo. Compound **32** was obtained in 87% yield (231.6 mg) as white solid (m.p.: 121 °C) after chromatography on silica gel (9:1 v/v, petroleum ether/ethyl acetate).

^{1}H-NMR (400 MHz, Acetone-d_6) δ: 8.32 (br, 1H), 7.84 – 7.78 (m, 2H), 7.63 – 7.45 (m, 3H), 6.46 (t, J = 53.1 Hz, 1H).

^{13}C-NMR (100 MHz, Acetone-d_6) δ: 156.7 (s), 132.6 (s), 129.9 (s), 127.8 (s), 124.4 (s), 122.8 (q, J = 289.8 Hz), 111.9 (t, J = 249.9 Hz), 94.9 (br, m).

^{19}F-NMR (377 MHz, Acetone-d_6) δ: -81.6 (s) -134.3 (dd, J = 299.5, 53.4 Hz), -136.7 (dd, J = 299.5, 53.4 Hz).

HRMS (ESI), m/z: calc. for $C_{10}H_8F_5N_2O^+$: 267,0551 $[M+H]^+$; found: 267,0550.

3-(4-methoxyphenyl)-5-methyl-5-(trifluoromethyl)-2,5-dihydro-1,2,4-oxadiazole (33)

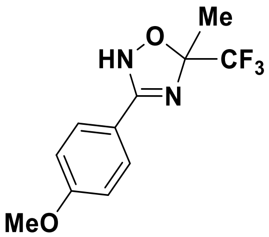

By following the general procedure 2 (GP2), starting from 3-(4-methoxyphenyl)-5-(trifluoromethyl)-1,2,4-oxadiazole (244.2 mg, 1.0 mmol, 1.0 equiv), and MeLi (0.68 mL of a solution 1.6 M in Et_2O, 1.1 mmol, 1.1 equiv) in THF (5 mL), compound **33** was obtained in 95% yield (247.2 mg) as white solid (m.p.: 86 °C) after filtration on silica pad.

^{1}H-NMR (600 MHz, Acetone-d_6) δ: 7.70-7.65 (m, 2H), 7.24 (br, 1H), 7.05-6.99 (m, 2H), 3.85 (s, 3H), 1.74-1.70 (br q, 3H).

^{13}**C-NMR** (150 MHz, Acetone-d_6) δ: 162.9 (s), 155.9 (s), 129.1 (s), 124.6 (q, J = 288.1 Hz), 118.0 (s), 115.0 (s), 95.3 (q, J = 32.0 Hz), 55.8 (s), 19.6 (s).
^{19}**F-NMR** (565 MHz, Acetone-d_6) δ: -86.7 (s).
HRMS (ESI), m/z: calc. for $C_{11}H_{12}F_3N_2O_2^+$: 261,0845 [M+H]$^+$; found: 261,0845.

5-butyl-3-phenyl-5-(trifluoromethyl)-2,5-dihydro-1,2,4-oxadiazole (34)

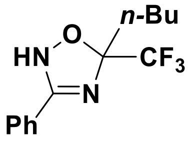

By following the general procedure 2 (GP2), starting from 3-(phenyl)-5-(trifluoromethyl)-1,2,4-oxadiazole (214.2 mg, 1.0 mmol, 1.0 equiv), and *n*-BuLi (0.44 mL of a solution 2.5 M in hexane, 1.1 mmol, 1.1 equiv) in THF (5 mL), compound **34** was obtained in 96% yield (261.4 mg) as white solid (m.p.: 148 °C) after filtration on silica pad.
^{1}**H-NMR** (600 MHz, Acetone-d_6) δ: 7.76 (m, 2H), 7.56-7.50 (m, 1H), 7.50-7.45 (m, 2H), 7.39 (br, 1H), 2.03-1.98 (m, 2H), 1.52 (m, 2H), 1.46-1.33 (m, 2H), 0.90 (t, 3H, J= 7.3 Hz).
^{13}**C-NMR** (150 MHz, Acetone-d_6) δ: 156.2 (s), 131.9 (s), 129.6 (s), 127.4 (s), 125.6 (s), 124.6 (q, J=288.7 Hz), 97.8 (q, J=31.0 Hz), 31.4 (s), 24.2 (s), 23.0 (s), 14.2 (s).
^{19}**F-NMR** (565 MHz, Acetone-d_6) δ: -86.0 (s).
HRMS (ESI), m/z: calc. for $C_{13}H_{16}F_3N_2O^+$: 273,1209 [M+H]$^+$; found: 273,1209.

5-(*sec*-butyl)-3-phenyl-5-(trifluoromethyl)-2,5-dihydro-1,2,4-oxadiazole (35)

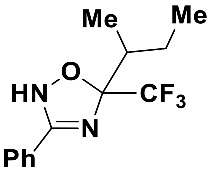

By following the general procedure 2 (GP2), starting from 3-(phenyl)-5-(trifluoromethyl)-1,2,4-oxadiazole (214.2 mg, 1.0 mmol, 1.0 equiv), and *sec*-BuLi (0.78 mL of a solution 1.4 M in cyclohexane, 1.1 mmol, 1.1 equiv) in THF (5 mL), compound **35** was obtained in 94% yield (256.0 mg) as white solid (m.p.: 134-138 °C) after filtration on silica pad.
^{1}**H-NMR** (400 MHz, Acetone-d_6) δ: 7.50 (m, 2H), 7.21 (m, 3H), 7.07 (br, 1H), 1.84 (m, 1H), 1.55 (m, 1H), 1.12 - 0.92 (m, 1H), 0.90 - 0.81 (m, 3H), 0.70 (m ,3H).
^{13}**C-NMR** (100 MHz, Acetone-d_6) δ: 156.21 (s), 156.18 (s), 131.88 (s), 129.56 (s), 127.49 (s), 125.67 (s), 125.1 (q, J = 209.7 Hz), 125.0 (q, J = 209.7 Hz), 99.9 (q, J = 29.4 Hz), 100.1 (q, J = 29.4 Hz), 39.51 (s), 39.38 (s), 23.21 (br q, J = 2.1 Hz), 23.03 (br q, J = 1.5 Hz), 12.78 (q, J = 1.9 Hz), 12.25 (q, J = 1.8 Hz), 12.02 (s), 11.90 (s).
^{19}**F-NMR** (377 MHz, Acetone-d_6) δ: -80.8 (s), 81.3 (s).
HRMS (ESI), m/z: calc. for $C_{13}H_{16}F_3N_2O^+$: 273,1209 [M+H]$^+$; found: 273,1209.

4-(5-hexyl-5-(trifluoromethyl)-2,5-dihydro-1,2,4-oxadiazol-3-yl)-*N*,*N*-dimethylaniline (36)

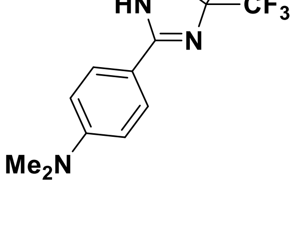

By following the general procedure 2 (GP2), starting from *N*,*N*-dimethyl-4-(5-phenyl-5-(trifluoromethyl)-2,5-dihydro-1,2,4-oxadiazol-3-yl)aniline (335.3 mg, 1.0 mmol, 1.0 equiv), and n-HexLi (0.48 mL of a solution 2.3 M in hexane, 1.1 mmol, 1.1 equiv) in THF (5 mL), compound **36** was obtained in 93% yield (319.4 mg) as white solid (m.p.: 184 °C) after filtration on silica pad.
^{1}H-NMR (600 MHz, Acetone-d_6) δ: 7.58 - 7.53 (m, 2H), 7.09 (br, 1H), 6.78 - 6.71 (m, 2H), 3.00 (s, 3H), 2.00 - 1.90 (m, 2H), 1.60 - 1.45 (m, 2H), 1.42 - 1.32 (m, 2H), 1.31 - 1.23 (m, 2H), 0.86 (t, 3H, J = 7.1 Hz).
^{13}C-NMR (150 MHz, Acetone-d_6) δ: 156.2 (s), 153.1 (s), 128.5 (s), 124.8 (q, J = 288.9 Hz), 112.3 (s), 97.0 (q, J = 30.6 Hz), 40.1 (s), 32.3 (s), 31.7 (s), 29.6 (s), 23.1 (s), 22.0 (s), 14.2 (s).
^{19}F-NMR (565 MHz, Acetone-d_6) δ: -85.8 (s).
HRMS (ESI), m/z: calc. for $C_{17}H_{25}F_3N_3O^+$: 344,1944 [M+H]$^+$; found: 344,1946.

3,5-diphenyl-5-(trifluoromethyl)-2,5-dihydro-1,2,4-oxadiazole (37)

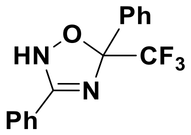

By following the general procedure 2 (GP2), starting from 3-(phenyl)-5-(trifluoromethyl)-1,2,4-oxadiazole (214.2 mg, 1.0 mmol, 1.0 equiv), and PhLi (0.58 mL of a solution 1.9 M in Bu_2O, 1.1 mmol, 1.1 equiv) in THF (5 mL), compound **37** was obtained in 94% yield (274.8 mg) as white solid (m.p.: 182 °C) after filtration on silica pad.
^{1}H-NMR (600 MHz, Acetone-d_6) δ: 8.03 (br, 1H), 7.87 - 7.83 (m, 2H), 7.79 - 7.75 (m, 2H), 7.56 - 7.45 (m, 6H).
^{13}C-NMR (150 MHz, Acetone-d_6) δ: 156.2 (s), 135.7 (s), 132.1 (s), 130.7 (s), 129.6 (s), 129.4 (s), 127.5 (s), 127.0 (s), 125.3 (s), 124.4 (q, J = 289.1 Hz), 97.2 (q, J = 32.0 Hz).
^{19}F-NMR (565 MHz, Acetone-d_6) δ: -83.4 (s).
HRMS (ESI), m/z: calc. for $C_{15}H_{12}F_3N_2O^+$: 293,0896 [M+H]$^+$; found: 293,0896.

3-phenyl-5-(thiophen-2-yl)-5-(trifluoromethyl)-2,5-dihydro-1,2,4-oxadiazole (38)

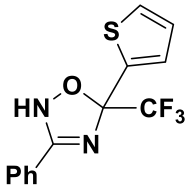

By following the general procedure 2 (GP2), starting from -(phenyl)-5-(trifluoromethyl)-1,2,4-oxadiazole (214.2 mg, 1.0 mmol, 1.0 equiv), and 2-thienyllithium (0.11 mL of a solution 1.0 M in THF, 1.1 mmol, 1.1 equiv) in THF (5 mL), compound **38** was obtained in 95% yield (298.3 mg) as white solid (m.p.: 122 °C) after filtration on silica pad.
^{1}H-NMR (400 MHz, Acetone-d_6) δ: 8.13 (br, 1H), 7.87 - 7.77 (m, 2H), 7.66 (dd, 1H, J = 5.1, 1.3 Hz), 7.60 - 7.42 (m, 4H), 7.15 (dd, 1H, J = 5.1, 3.7 Hz).
^{13}C-NMR (100 MHz, Acetone-d_6) δ: 156.0 (s), 138.5 (s), 132.3 (s), 129.7 (s), 129.0 (s), 128.5 (s), 127.6 (s), 125.1 (s), 124.1 (d, J = 288.1 Hz), 95.9 (q, J = 33.5 Hz).
^{19}F-NMR (377 MHz, Acetone-d_6) δ: -83.5 (s).
HRMS (ESI), m/z: calc. for $C_{13}H_{10}F_3N_2OS^+$: 299,0460 [M+H]$^+$; found: 299,0460.

N,N-dimethyl-4-(5-methyl-5-(trifluoromethyl)-2,5-dihydro-1,2,4-oxadiazol-3-yl)aniline (39)

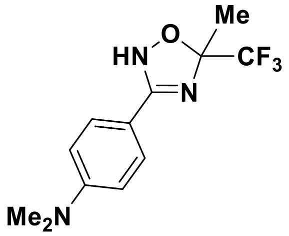

By following the general procedure 2 (GP2), starting from N,N-dimethyl-4-(5-phenyl-5-(trifluoromethyl)-2,5-dihydro-1,2,4-oxadiazol-3-yl)aniline (335.3 mg, 1.0 mmol, 1.0 equiv), and MeMgCl (0.37 mL of a solution 3.0 M in THF, 1.1 mmol, 1.1 equiv) in THF (5 mL), compound **39** was obtained in 93% yield (254.2 mg) as white solid (m.p.: 187 °C) after filtration on silica pad.

^{1}H-NMR (600 MHz, Acetone-d_6) δ: 7.56 - 7.53 (m, 2H), 7.08 (br), 6.77 - 6.73 (m, 2H), 3.01 (s, 3H), 1.69 (q, 3H, J = 1.2 Hz).

^{13}C-NMR (150 MHz, Acetone-d_6) δ: 156.2 (s), 153.2 (s), 128.5 (s), 124.7 (q, J = 288.3 Hz), 112.4 (s), 112.3 (s), 40.1 (s), 19.6 (s).

^{19}F-NMR (565 MHz, Acetone-d_6) δ: -86.6 (s).

HRMS (ESI), m/z: calc. for $C_{12}H_{15}F_3N_3O^+$: 274,1162 [M+H]$^+$; found: 274,1163.

3-(4-methoxyphenyl)-5-ethyl-5-(trifluoromethyl)-2,5-dihydro-1,2,4-oxadiazole (40)

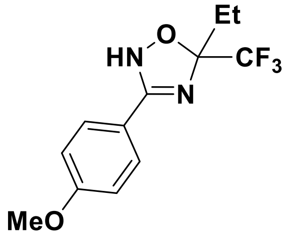

By following the general procedure 2 (GP2), starting from 3-(4-methoxyphenyl)-5-(trifluoromethyl)-1,2,4-oxadiazole (244.2 mg, 1.0 mmol, 1.0 equiv), and EtMgCl (0.55 mL of a solution 2.0 M in THF, 1.1 mmol, 1.1 equiv) in THF (5 mL), compound **40** was obtained in 94 (257.8 mg) as white solid (m.p.: 198 °C) after filtration on silica pad.

^{1}H-NMR (600 MHz, Acetone-d_6) δ: 7.71 - 7.67 (m, 2H), 7.24 (br, 1H), 7.04 - 7.00 (m, 2H), 3.85 (s, 3H), 2.00 (qd, 2H, J = 7.4, 0.7 Hz), 1.04 (td, 3H, J = 7.4, 0.5 Hz).

^{13}C-NMR (150 MHz, Acetone-d_6) δ: 162.4 (s), 155.5 (s), 128.6 (s), 124.3 (q, J = 288.6 Hz), 117.4 (s), 114.6 (s), 97.3 (q, J = 30.8 Hz), 55.4 (s), 24.3 (s), 5.4 (s).

^{19}F-NMR (565 MHz, Acetone-d_6) δ: -85.8 (s).

HRMS (ESI), m/z: calc. for $C_{12}H_{14}F_3N_2O_2^+$: 275,1002 [M+H]$^+$; found: 275,1002.

5-benzyl-3-(4-methoxyphenyl)-5-(trifluoromethyl)-2,5-dihydro-1,2,4-oxadiazole (41)

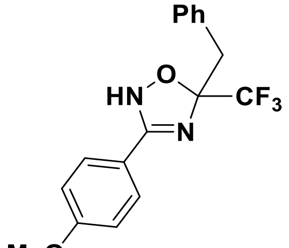

By following the general procedure 2 (GP2), starting from 3-(4-methoxyphenyl)-5-(trifluoromethyl)-1,2,4-oxadiazole (244.2 mg, 1.0 mmol, 1.0 equiv), and benzylmagnesium chloride (0.55 mL of a solution 2.0 M in

Et_2O, 1.1 mmol, 1.1 equiv) in THF (5 mL), compound **41** was obtained in 95% yield (319.5 mg) as white solid (m.p.: 161 °C) after filtration on silica pad.
^{1}H-NMR (600 MHz, Acetone-d_6) δ: 7.51 - 7.47 (m, 2H), 7.43 - 7.40 (m, 2H), 7.28 (br, 1H), 7.27 - 7.22 (m, 2H), 7.21 (m, 1H), 6.94 - 6.90 (m, 2H), 3.79 (s, 3H), 3.37 (d, 1H, J = 14.7 Hz), 3.26 (d, 1H, J = 14.7 Hz).
^{13}C-NMR (150 MHz, Acetone-d_6) δ: 162.7 (s), 155.6 (s), 133.5 (s), 131.8 (s), 128.8 (s), 128.7 (s), 124.5 (q, J = 289.2 Hz), 117.4 (s), 114.8 (s), 97.4 (q, J = 30.7 Hz), 55.7 (s), 37.7 (s).
^{19}F-NMR (565 MHz, Acetone-d_6) δ: -84.9 (s).
HRMS (ESI), m/z: calc. for $C_{17}H_{16}F_3N_2O_2^+$: 337,1158 $[M+H]^+$; found: 337,1158.

Synthetic manipulation of products

4-methyl-3-phenyl-5-(trifluoromethyl)-5-((trimethylsilyl)methyl)-4,5-dihydro-1,2,4-oxadiazole (42)

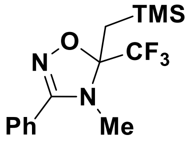

To a solution of 5-(iodomethyl)-4-methyl-3-phenyl-5-(trifluoromethyl)-4,5-dihydro-1,2,4-oxadiazole (**15**) (370.1 mg, 1 mmol, 1.0 equiv) in THF (5 mL) cooled at -78 °C, *sec*-BuLi (0.78 mL of a solution 1.4 M in cyclohexane, 1.1 mmol, 1.1 equiv) was added dropwise and the resulting mixture was stirred at low temperature for 10 minutes. TMSOTf (266.7 mg, 0.21 mL, 1.2 mmol, 1.2 equiv) was added dropwise and the solution was stirred overnight and allowed to slowly reach room temperature. After quenching with a saturated solution of $NH_4Cl_{(aq)}$ (3 mL), the resulting mixture was extracted 3 times with Et_2O, the organic phase was washed with brine, dried over anhydrous Na_2SO_4 and concentrated in vacuo. Compound **42** was obtained in 75% yield (237.3 mg) as yellow solid (m.p.: 50 °C) after chromatography on silica gel (97:3 v/v, petroleum ether/ethyl acetate).
^{1}H-NMR (600 MHz, $CDCl_3$) δ: 7.73 – 7.70 (m, 2H), 7.56 – 7.51 (m, 1H), 7.48 – 7.44 (m, 2H), 3.12 (s, 3H), 1.58 (dd, 1H, J = 15.1, 0.9 Hz), 1.46 (d, 1H, J = 15.1 Hz), 0.11 (s, 9H).
^{13}C-NMR (150 MHz, $CDCl_3$) δ: 167.4 (s), 132.0 (s), 128.9 (s), 128.7 (s), 126.7 (s), 122.8 (q, J = 288.2 Hz), 106.6 (q, J = 30.1 Hz), 42.2 (s), 22.2 (s), -0.3 (s).
^{19}F-NMR (565 MHz, $CDCl_3$) δ: -84.4 (s).
HRMS (ESI), m/z: calc. for $C_{14}H_{20}F_3N_2OSi^+$: 317,1292 $[M+H]^+$; found: 317,1292.

1-(5-butyl-3-phenyl-5-(trifluoromethyl)-1,2,4-oxadiazol-2(5H)-yl)ethan-1-one (43)

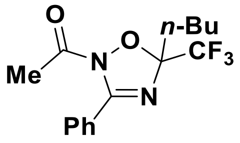

To a solution of 5-butyl-3-phenyl-5-(trifluoromethyl)-2,5-dihydro-1,2,4-oxadiazole (**34**) (272.3 mg, 1 mmol, 1.0 equiv) and triethylamine (111.3 mg, 0.10 mL, 1.1 mmol, 1.1 equiv) and DMAP (12.2 mg, 0.1 mmol, 0.01 equiv) in DCM (5 mL) cooled at 0 °C, acetic anhydride (112.3 mg, 0.15 mL, 1.1 mmol, 1.1 equiv) was added dropwise and the resulting mixture was stirred overnight and allowed to slowly reach room temperature. After quenching with a saturated solution of $NaHCO_{3\,(aq)}$ (3 mL), the resulting mixture was extracted 3 times with Et_2O, the organic phase was washed with brine, dried over anhydrous Na_2SO_4 and concentrated in vacuo. Compound **43** was obtained in 79% yield (248.3 mg) as yellow oil after chromatography on silica gel (95:5 v/v, petroleum ether/ethyl acetate).

^{1}H-NMR (400 MHz, $CDCl_3$) δ: 7.83 – 7.76 (m, 2H), 7.59 – 7.50 (m, 1H), 7.44 (t, 2H, J = 7.7 Hz), 2.30 (s, 3H), 2.16 (m, 1H), 2.05 (m, 1H), 1.40 (m, 4H), 0.93 (m, 3H).
^{13}C-NMR (100 MHz, $CDCl_3$) δ: 169.6 (s), 158.5 (s), 132.5 (s), 130.1 (s), 128.0 (s), 127.0 (s), 122.6 (q, J = 286.3 Hz), 105.8 (q, J = 27.2 Hz), 31.1 (s), 23.5 (s), 22.8 (s), 22.4 (s), 13.8 (s).
^{19}F-NMR (377 MHz, $CDCl_3$) δ: -82.7 (s).
HRMS (ESI), m/z: calc. for $C_{15}H_{18}F_3N_2O_2^+$: 315,1315 $[M+H]^+$; found: 315,1316.

5-(chloromethyl)-3-phenyl-5-(trifluoromethyl)-2,5-dihydro-1,2,4-oxadiazole (44)

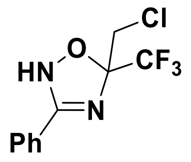

By following the general procedure 3 (GP3), starting from 3-(phenyl)-5-(trifluoromethyl)-1,2,4-oxadiazole (214.2 mg, 1.0 mmol, 1.0 equiv), chloroiodomethane (529.1 mg, 0.22 mL, 3.0 mmol, 3.0 equiv) and PhLi (1.47 mL of a solution 1.9 M in Bu_2O, 2.8 mmol, 2.8 equiv) in THF (5 mL), compound **44** was obtained in 85% yield (224.9 mg) as white solid (m.p.: 125 °C) after chromatography on silica gel (90:10 v/v, petroleum ether/ethyl acetate).
^{1}H-NMR (600 MHz, Acetone-d_6) δ: 7.84 (br, 1H), 7.79 – 7.76 (m, 2H), 7.55 (m, 1H), 7.52 – 7.48 (m, 2H), 4.12 (s, 2H).
^{13}C-NMR (150 MHz, Acetone-d_6) δ: 156.3 (s), 132.2 (s), 129.7 (s), 127.4 (s), 125.0 (s), 123.6 (q, J = 288.8 Hz), 96.9 (q, J = 31.4 Hz), 43.7 (s).
^{19}F-NMR (565 MHz, Acetone-d_6) δ: -83.4 (s).
HRMS (ESI), m/z: calc. for $C_{10}H_9ClF_3N_2O^+$: 265,0350 $[M+H]^+$; found: 265,0350.

5-(chloromethyl)-3-(thiophen-2-yl)-5-(trifluoromethyl)-2,5-dihydro-1,2,4-oxadiazole (45)

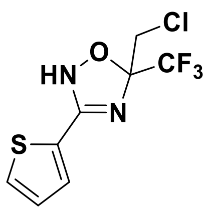

By following the general procedure 3 (GP3), starting from 3-(thiophen-2-yl)-5-(trifluoromethyl)-1,2,4-oxadiazole (220.2 mg, 1.0 mmol, 1.0 equiv), chloroiodomethane (529.1 mg, 0.22 mL, 3.0 mmol, 3.0 equiv) and PhLi (1.47 mL of a solution 1.9 M in Bu_2O, 2.8 mmol, 2.8 equiv) in THF (5 mL), compound **45** was obtained in 88% yield (238.1 mg) as white solid (m.p.: 132°C) after chromatography on silica gel (85:15 v/v, petroleum ether/ethyl acetate).
^{1}H-NMR (600 MHz, Acetone-d_6) δ: 8.07 (br, 1H), 7.71 (dd, 1H, J = 5.1, 1.1 Hz), 7.59 (dd, 1H, J = 3.7, 1.1 Hz), 7.18 (dd, 1H, J = 5.1, 3.7 Hz), 4.11 (s, 2H).
^{13}C-NMR (150 MHz, Acetone-d_6) δ: 152.5 (s), 130.5 (s), 130.3 (s), 128.5 (s), 125.6 (s), 123.5 (q, J = 288.9 Hz), 96.9 (q, J = 31.6 Hz), 43.5 (s).
^{19}F-NMR (565 MHz, Acetone-d_6) δ: -83.3 (s).
HRMS (ESI), m/z: calc. for $C_8H_7ClF_3N_2OS^+$: 270,9914 $[M+H]^+$; found: 270,9914.

2-benzyl-5-(chloromethyl)-3-(4-chlorophenyl)-5-(trifluoromethyl)-2,5-dihydro-1,2,4-oxadiazole (46)

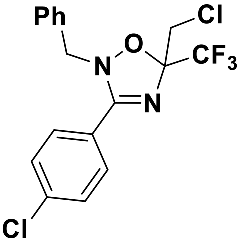

By following the general procedure 3 (GP3), starting from 3-(4-chlorophenyl)-5-(trifluoromethyl)-1,2,4-oxadiazole (248.6 mg, 1.0 mmol, 1.0 equiv), chloroiodomethane (529.1 mg, 0.22 mL, 3.0 mmol, 3.0 equiv), PhLi (1.47 mL of a solution 1.9 M in Bu_2O, 2.8 mmol, 2.8 equiv) and benzyl bromide (188.1 mg, 0.13 mL, 1.1 mmol, 1.1 equiv), in THF (5 mL), compound **46** was obtained in 82% yield (319.1 mg) as white solid (m.p.: 110 °C) after chromatography on silica gel (97:3 v/v, petroleum ether/ethyl acetate).
^{1}H NMR (600 MHz, $CDCl_3$) δ: 7.77 (m, 2H), 7.49 (m, 2H), 7.43 – 7.34 (m, 5H), 4.59 (s, 2H), 3.93 (d, 1H, J = 12.2 Hz), 3.80 (d, 1H, J = 12.3 Hz).
^{13}C NMR (150 MHz, $CDCl_3$) δ: 167.4 (s), 138.9 (s), 134.9 (s), 130.2 (s), 129.4 (s), 128.6 (s), 128.1 (s), 127.9 (s), 124.6 (s), 121.9 (q, J = 287.8 Hz), 105.8 (q, J = 30.2 Hz), 59.1 (s), 43.4 (s).
^{19}F NMR (565 MHz, $CDCl_3$) δ: -80.3 (s).
HRMS (ESI), m/z: calc. for $C_{17}H_{14}Cl_2F_3N_2O^+$: 389,0430 $[M+H]^+$; found: 389,0430.

2-allyl-5-(chloromethyl)-3-phenyl-5-(trifluoromethyl)-2,5-dihydro-1,2,4-oxadiazole (47)

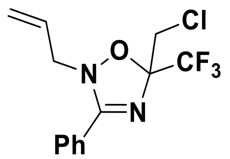

By following the general procedure 3 (GP3), starting from 3-(4-chlorophenyl)-5-(trifluoromethyl)-1,2,4-oxadiazole (248.6 mg, 1.0 mmol, 1.0 equiv), chloroiodomethane (529.1 mg, 0.22 mL, 3.0 mmol, 3.0 equiv), PhLi (1.47 mL of a solution 1.9 M in Bu_2O, 2.8 mmol, 2.8 equiv) and allyl iodide (184.8 mg, 0.10 mL, 1.1 mmol, 1.1 equiv), in THF (5 mL), compound **47** was obtained in 86% yield (262.0 mg) as orange oil after chromatography on silica gel (97:3 v/v, petroleum ether/ethyl acetate).
^{1}H-NMR (600 MHz, $CDCl_3$) δ: 7.75 – 7.73 (m, 2H), 7.58 – 7.55 (m, 1H), 7.50 – 7.45 (m, 2H), 5.96 (ddt, 1H, J = 17.1, 10.3, 5.8 Hz), 5.30 (dq, 1H, J = 17.1, 1.5 Hz), 5.26 (dq, 1H, J = 10.3, 1.3 Hz), 4.05 – 4.00 (m, 3H), 3.86 (d, 1H, J = 12.2 Hz).
^{13}C-NMR (150 MHz, $CDCl_3$) δ: 168.5 (s), 132.5 (s), 131.6 (s), 129.0 (s), 128.8 (s), 126.2 (s), 122.1 (q, J = 288.1 Hz), 118.9 (s), 105.6 (q, J = 30.1 Hz), 58.0 (s), 43.7 (s).
^{19}F-NMR (565 MHz, $CDCl_3$) δ: -80.5 (s).
HRMS (ESI), m/z: calc. for $C_{13}H_{13}ClF_3N_2O^+$: 305,0663 $[M+H]^+$; found: 305,0663.

3-(4-methoxyphenyl)-2,5-dimethyl-5-(trifluoromethyl)-2,5-dihydro-1,2,4-oxadiazole (48)

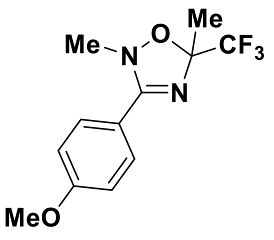

To a solution of 5-(trifluoromethyl)-3-(4-(methoxy)phenyl)-1,2,4-oxadiazole (282.2 mg, 1.0 mmol, 1.0 equiv) in THF (5 mL) cooled at -78 °C, MeLi (0.68 mL of a solution 1.6 M in Et_2O, 1.1 mmol, 1.1 equiv) was added dropwise and the mixture was stirred at low temperature for 1 hour. Then MeI (156.1 mg, 0.07 mL, 1.1 mmol, 1.1 equiv) was added dropwise and the mixture was stirred overnight and allowed to gradually reach room temperature; subsequently, a saturated solution of $NH_4Cl_{(aq.)}$ was added. The organic phase was extracted with EtOAc (3 x 10 mL) and was washed with brine then, was dried over anhydrous Na_2SO_4 and the solvent was removed under reduced pressure. Compound **48** was obtained in 84% yield (230.3 mg) as white solid (m.p.: 36 °C) after chromatography on silica gel (90:10 v/v, petroleum ether/ethyl acetate).
^{1}H-NMR (600 MHz, $CDCl_3$) δ: 7.71 – 7.67 (m, 2H), 6.97 – 6.94 (m, 2H), 3.86 (s, 3H), 3.11 (s, 3H), 1.73 (br q, 3H).
^{13}C-NMR (150 MHz, $CDCl_3$) δ: 167.6 (s), 162.7 (s), 130.6 (s), 122.7 (q, J = 286.8 Hz), 118.5 (s), 114.2 (s), 104.4 (q, J = 30.6 Hz), 55.5 (s), 43.4 (s), 21.4 (s).
^{19}F-NMR (565 MHz, $CDCl_3$) δ: -83.6 (s).
HRMS (ESI), m/z: calc. for $C_{12}H_{14}F_3N_2O_2^+$: 275,1002 $[M+H]^+$; found: 275,1002.

2,5-dimethyl-5-(trifluoromethyl)-3-(4-(trifluoromethyl)phenyl)-2,5-dihydro-1,2,4-oxadiazole (49)

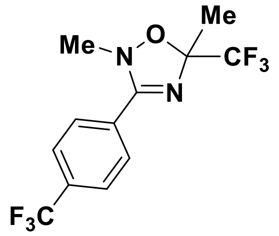

To a solution of 5-(trifluoromethyl)-3-(4-(trifluoromethyl)phenyl)-1,2,4-oxadiazole (282.2 mg, 1.0 mmol, 1.0 equiv) in THF (5 mL) cooled at -78 °C, MeLi (0.68 mL of a solution 1.6 M in Et_2O, 1.1 mmol, 1.1 equiv) was added dropwise and the mixture was stirred at low temperature for 1 hour. Then MeI (156.1 mg, 0.07 mL, 1.1 mmol, 1.1 equiv) was added dropwise and the mixture was stirred overnight and allowed to gradually reach room temperature; subsequently, a saturated solution of $NH_4Cl_{(aq.)}$ was added. The organic phase was extracted with EtOAc (3 x 10 mL) and was washed with brine then, was dried over anhydrous Na_2SO_4 and the solvent was removed under reduced pressure. Compound **49** was obtained in 93% yield (290.3 mg) as white solid (m.p.: 42 °C) after chromatography on silica gel (97:3 v/v, petroleum ether/ethyl acetate).
^{1}H-NMR (600 MHz, $CDCl_3$) δ: 7.88 – 7.85 (m, 2H), 7.75 – 7.71 (m, 2H), 3.12 (s, 3H), 1.76 (br q, 3H, J = 1.1 Hz).
^{13}C-NMR (150 MHz, $CDCl_3$) δ: 166.8 (s), 133.9 (q, J = 32.9 Hz), 129.9 (s), 129.3 (s), 125.9 (q, J = 3.6 Hz), 123.9 (q, J = 135.8 Hz), 122.1 (q, J = 149.9 Hz), 104.6 (q, J = 30.9 Hz), 42.8 (s), 21.2 (s).
^{19}F-NMR (565 MHz, $CDCl_3$) δ: -63.1 (s), -83.7 (s).
HRMS (ESI), m/z: calc. for $C_{12}H_{11}F_6N_2O^+$: 313,0770 $[M+H]^+$; found: 313,0770.

Copies of ^{1}H, ^{13}C and ^{19}F-NMR Spectra of all compounds

Isopropyl 3-(5-(trifluoromethyl)-1,2,4-oxadiazol-3-yl)benzoate (5-*sm*)

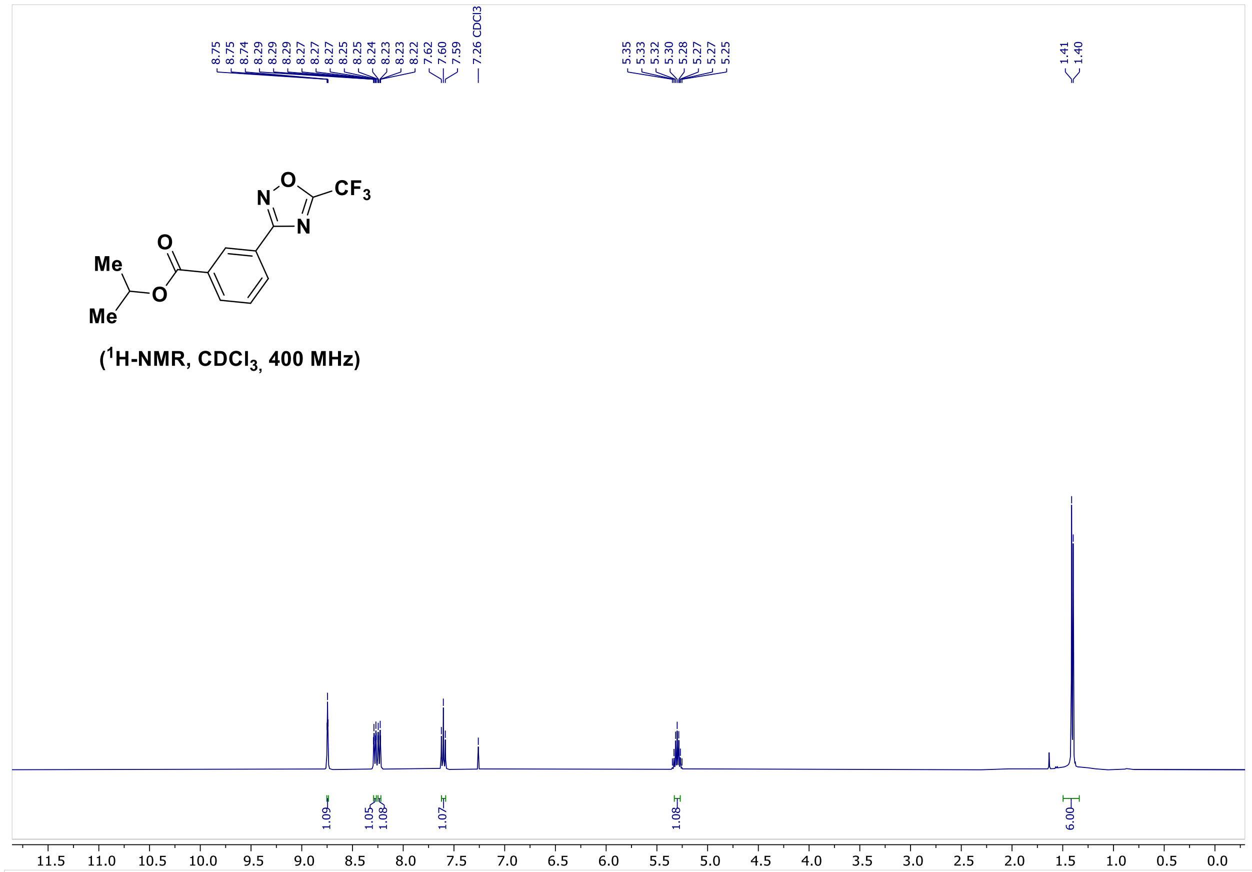
8.75 8.75 8.74 8.29 8.29 8.29 8.27 8.27 8.27 8.25 8.25 8.24 8.23 8.23 8.22 7.62 7.60 7.59
7.26 CDCl3
5.35 5.33 5.32 5.30 5.28 5.27 5.27 5.25
1.41 1.40
CF3
O
N
N
O
Me
O
Me
(1H-NMR, CDCl3, 400 MHz)
1.09 1.05 1.08 1.07 1.08 6.00
11.5 11.0 10.5 10.0 9.5 9.0 8.5 8.0 7.5 7.0 6.5 6.0 5.5 5.0 4.5 4.0 3.5 3.0 2.5 2.0 1.5 1.0 0.5 0.0

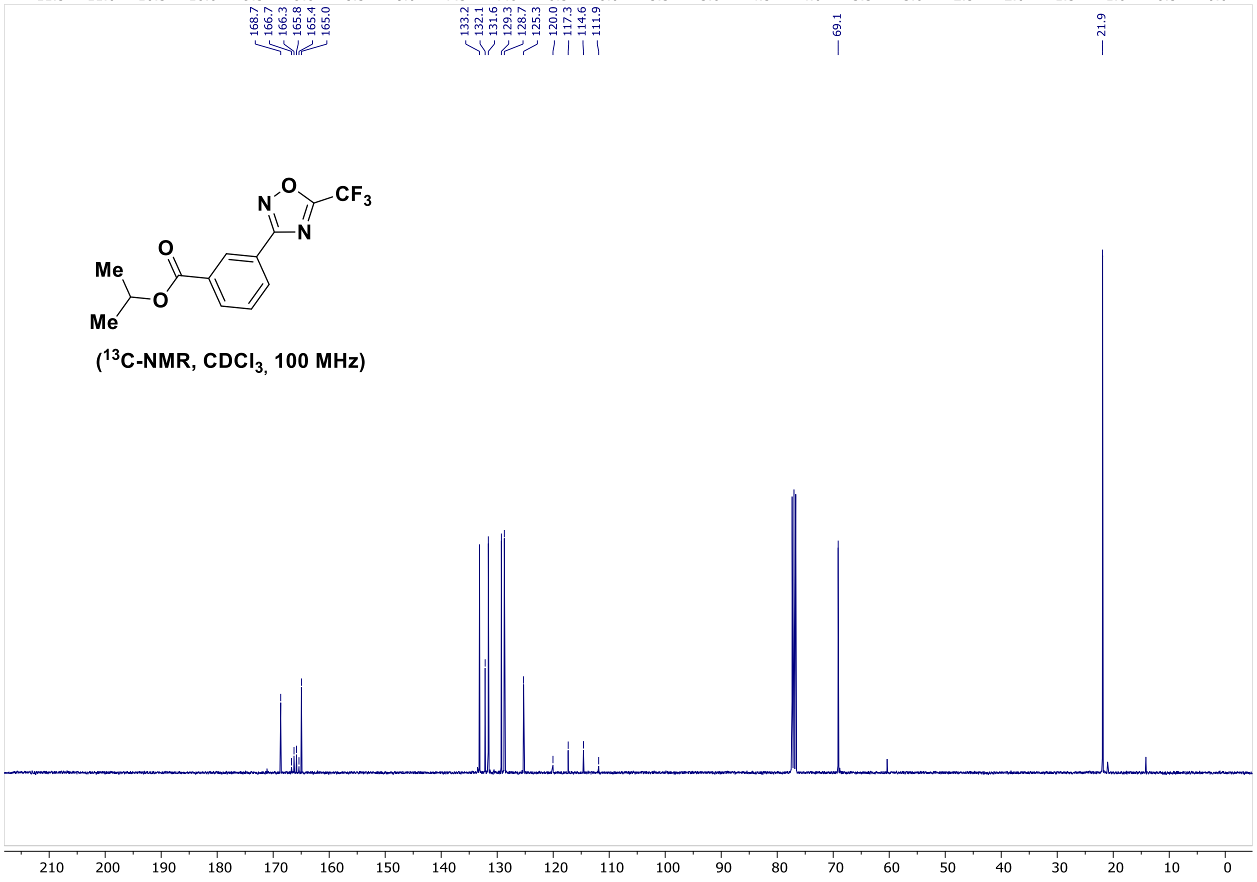
168.7 166.7 166.3 165.8 165.4 165.0
133.2 132.1 131.6 129.3 128.7 125.3 120.0 117.3 114.6 111.9
69.1
21.9
CF3
O
N
N
O
Me
O
Me
(13C-NMR, CDCl3, 100 MHz)
210 200 190 180 170 160 150 140 130 120 110 100 90 80 70 60 50 40 30 20 10 0

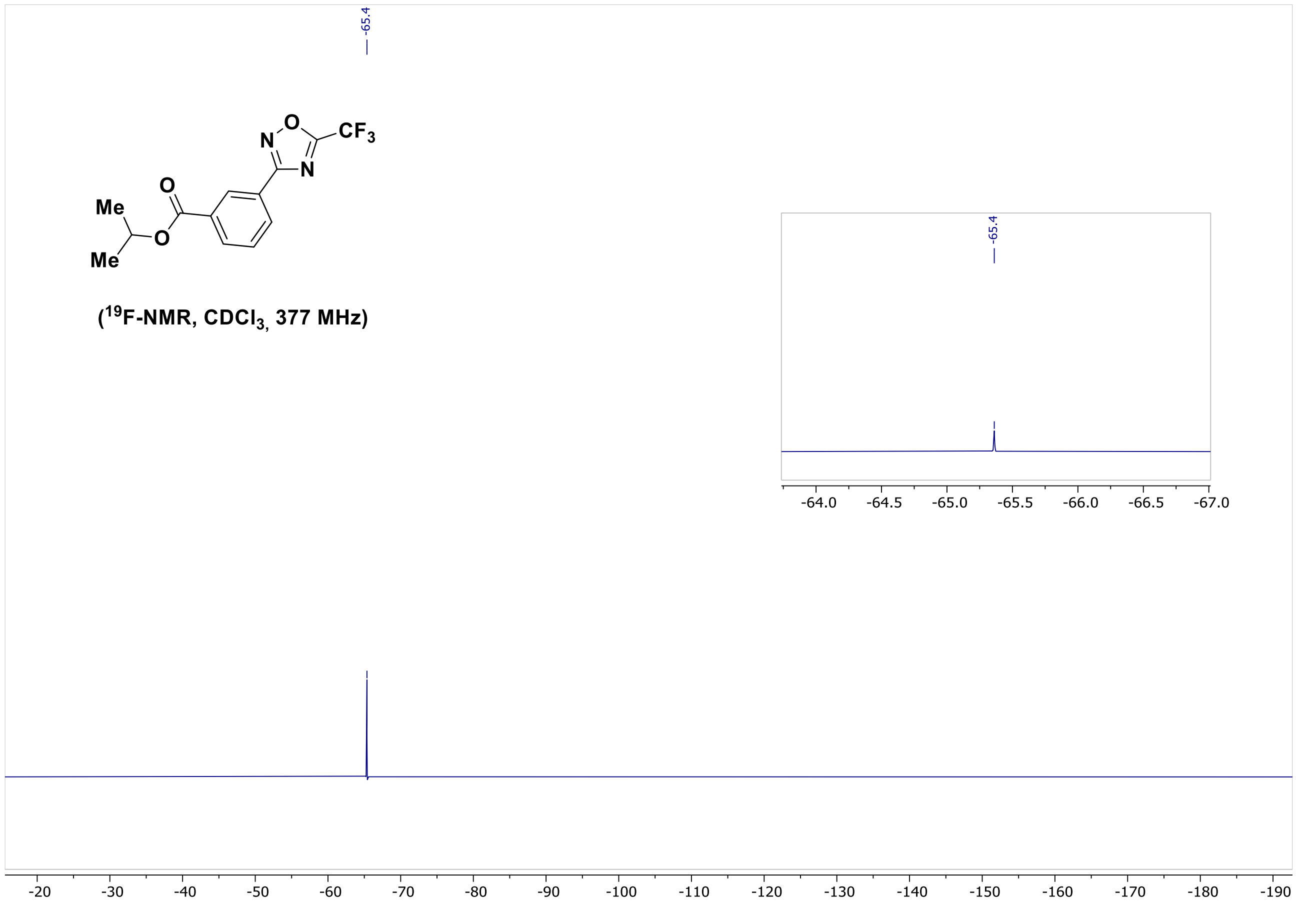

3-(pyridin-4-yl)-5-(trifluoromethyl)-1,2,4-oxadiazole (7-*sm*)

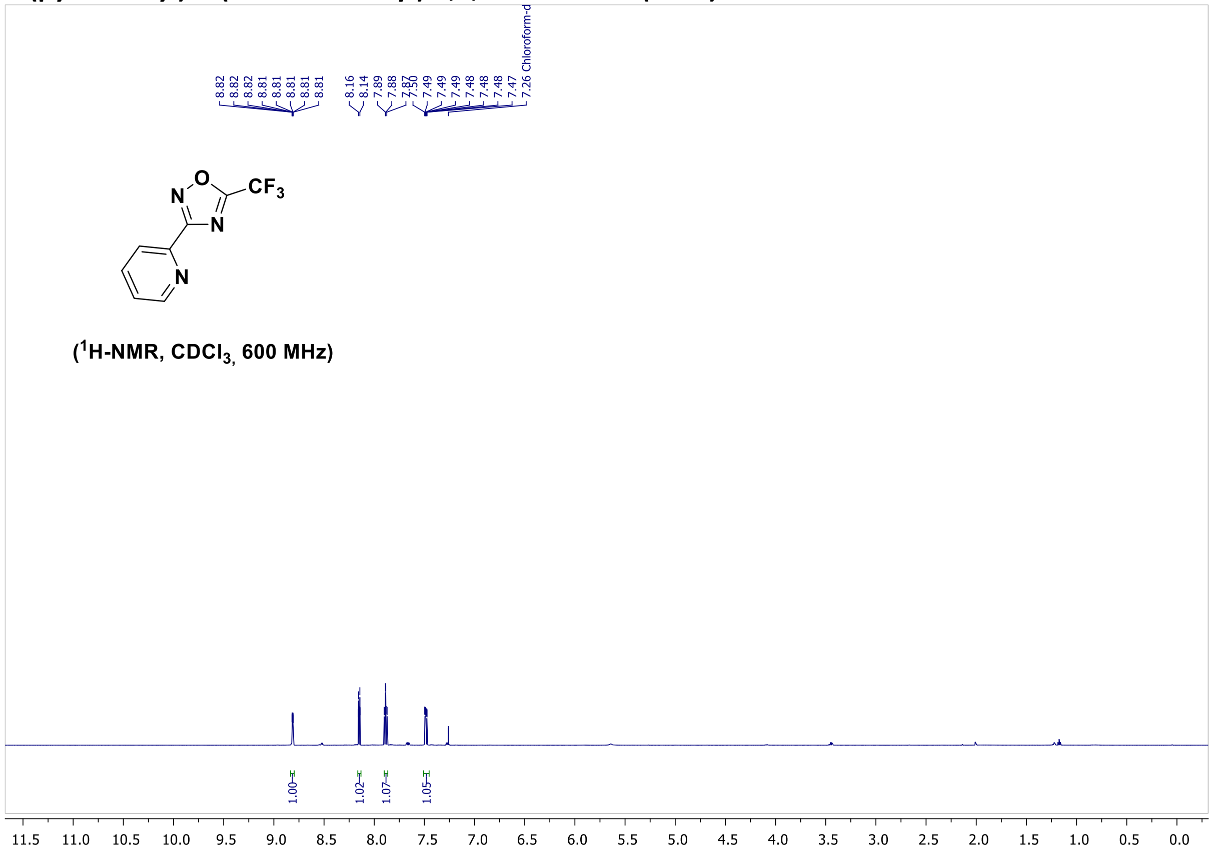

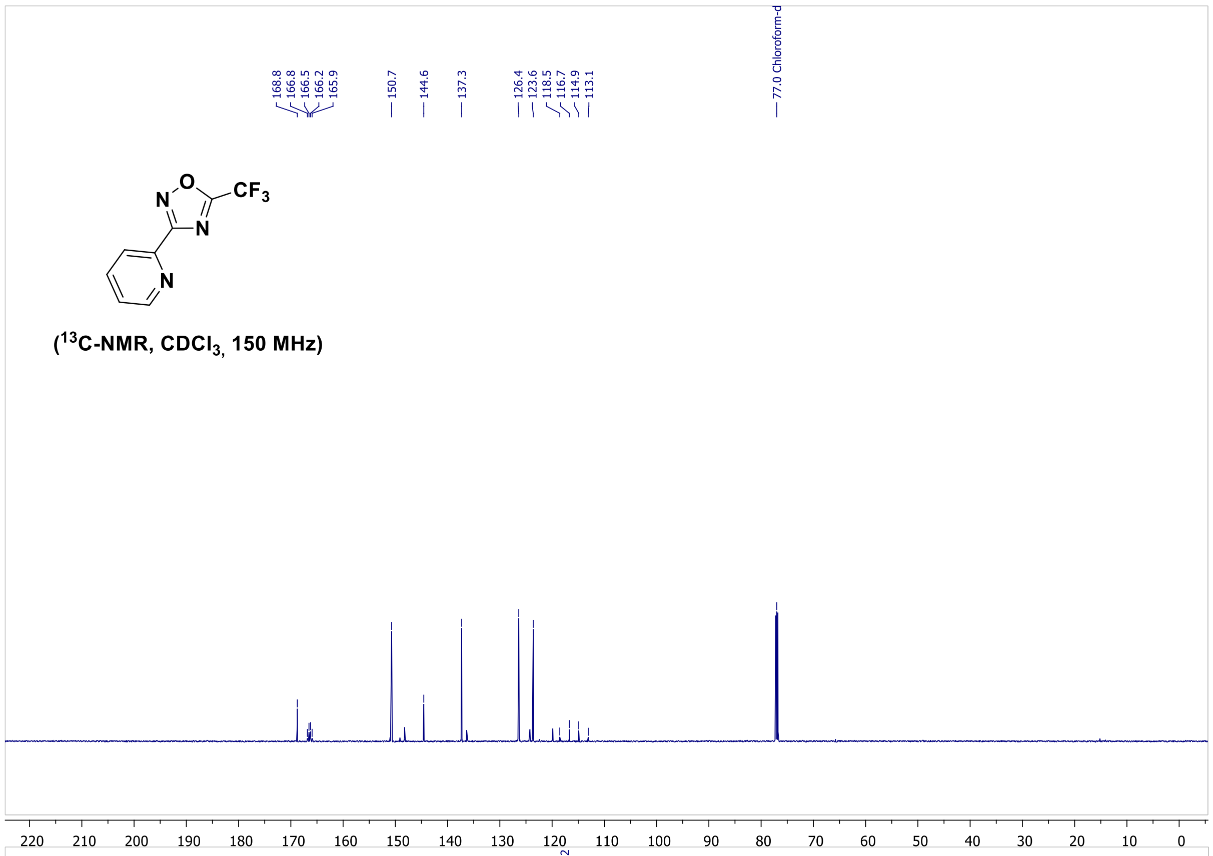
168.8
166.8
166.5
166.2
165.9
150.7
144.6
137.3
126.4
123.6
118.5
116.7
114.9
113.1
77.0 Chloroform-d
N
O
CF3
N
N
(^{13}C-NMR, $CDCl_3$, 150 MHz)
220 210 200 190 180 170 160 150 140 130 120 110 100 90 80 70 60 50 40 30 20 10 0

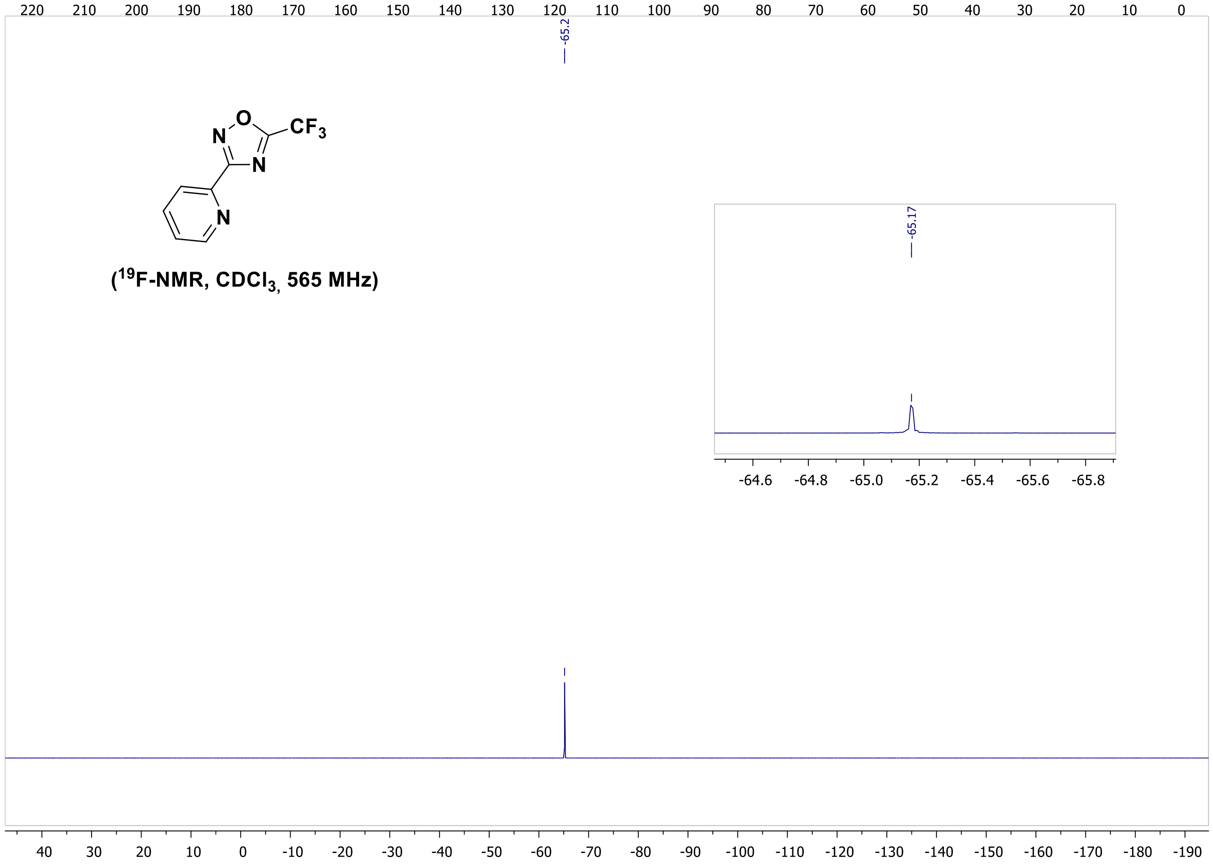
-65.2
N
O
CF3
N
N
(^{19}F-NMR, $CDCl_3$, 565 MHz)
-65.17
-64.6 -64.8 -65.0 -65.2 -65.4 -65.6 -65.8
40 30 20 10 0 -10 -20 -30 -40 -50 -60 -70 -80 -90 -100 -110 -120 -130 -140 -150 -160 -170 -180 -190

3-(thiophen-2-yl)-5-(trifluoromethyl)-1,2,4-oxadiazole (13-*sm*)

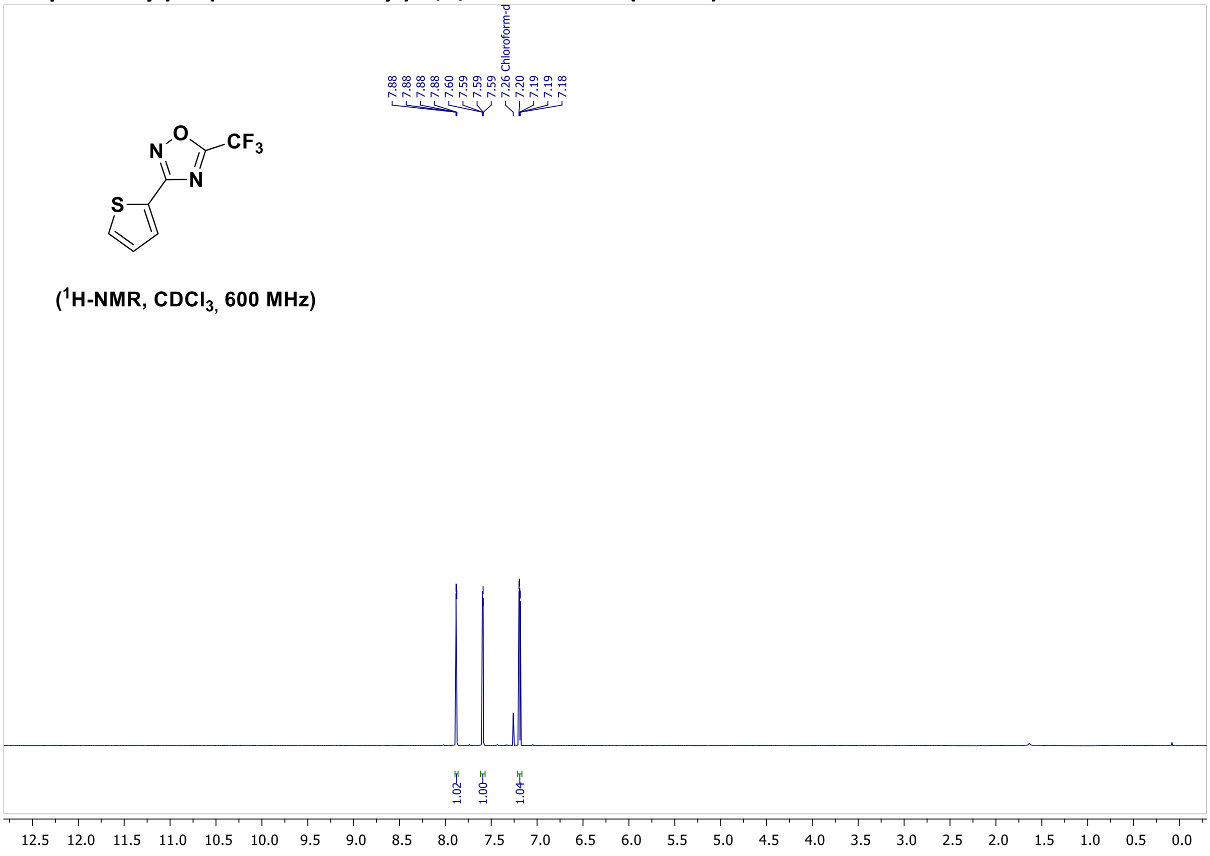

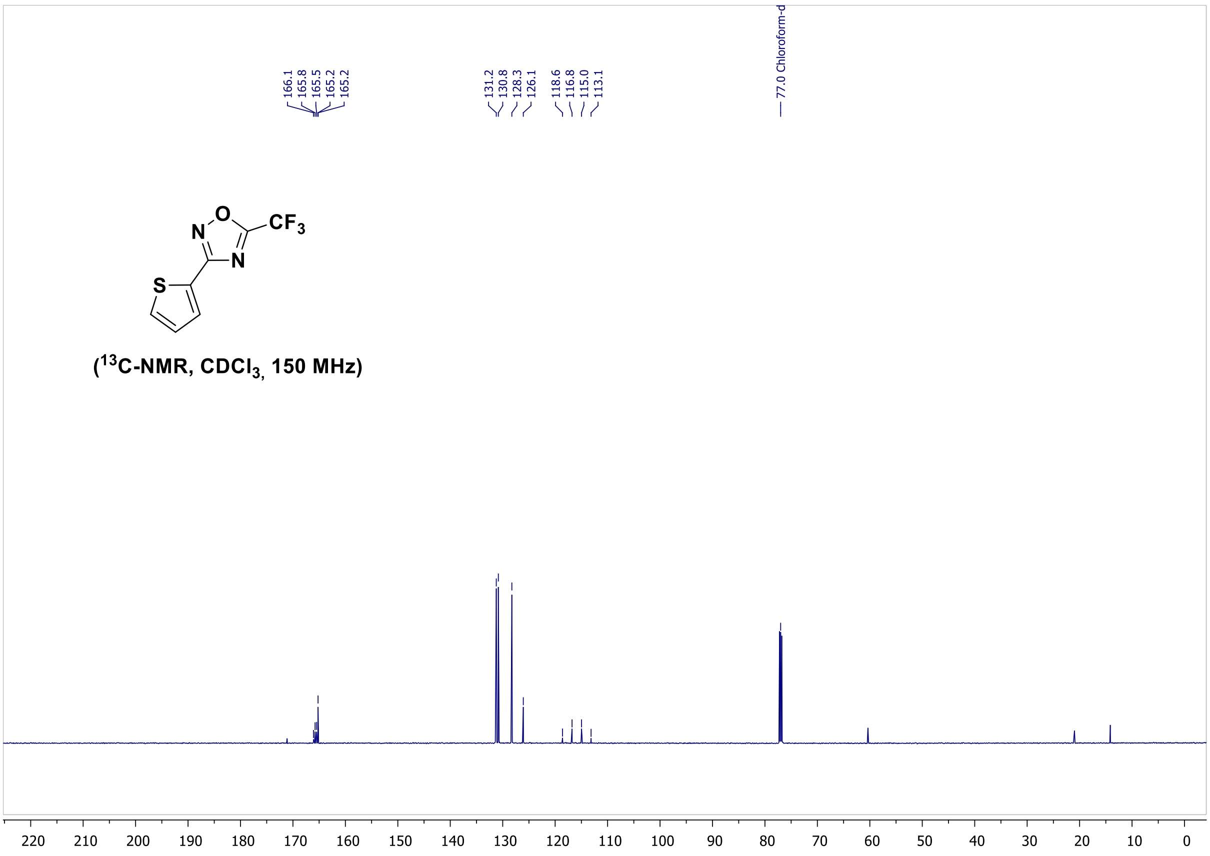

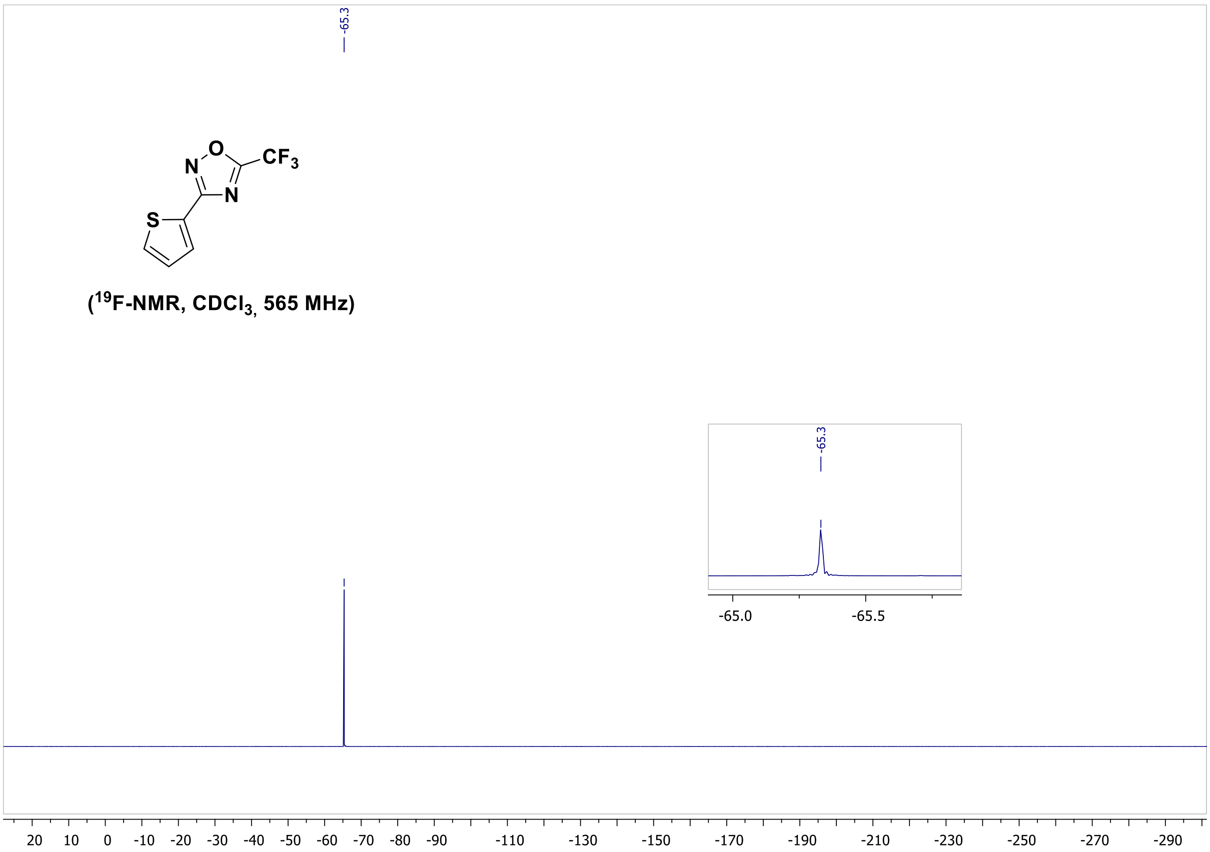

N,N-dimethyl-4-(5-(trifluoromethyl)-1,2,4-oxadiazol-3-yl)aniline (16-*sm*)

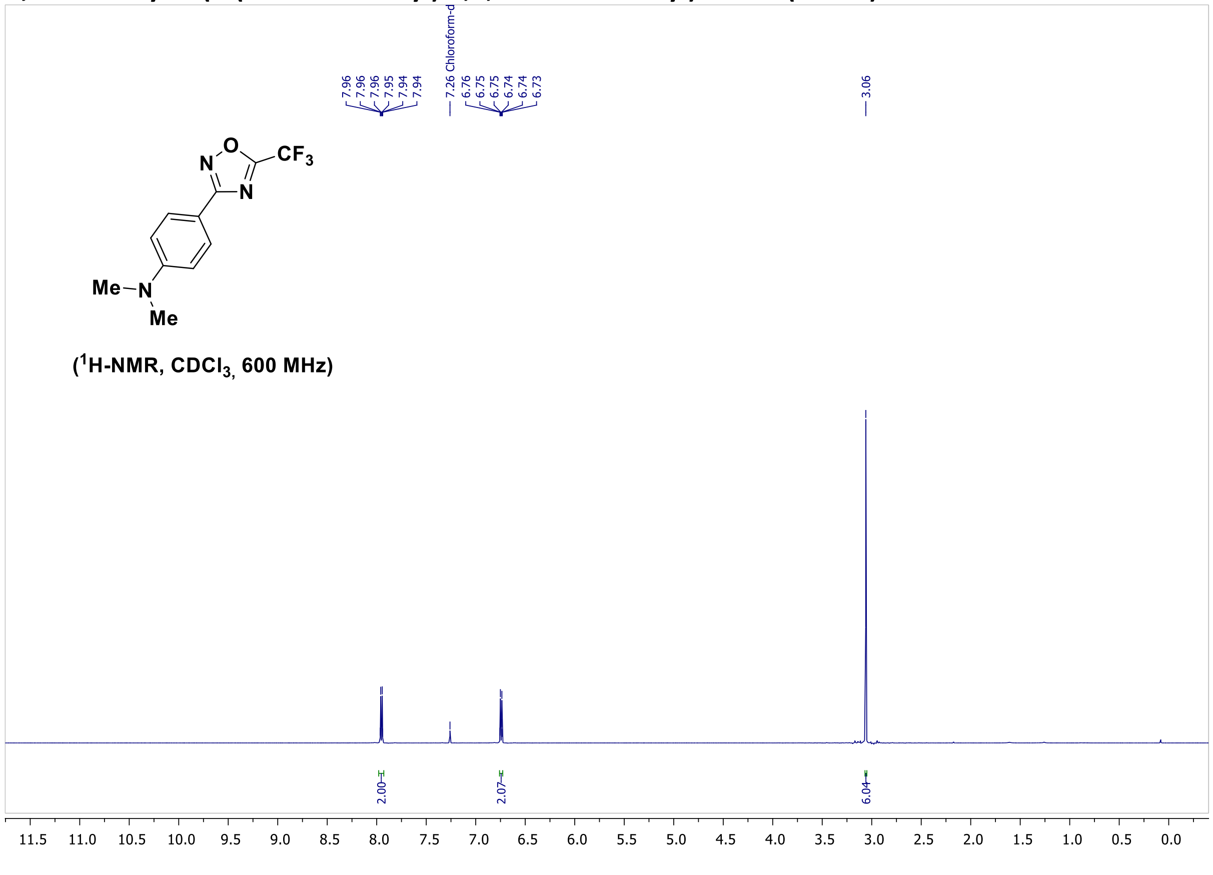

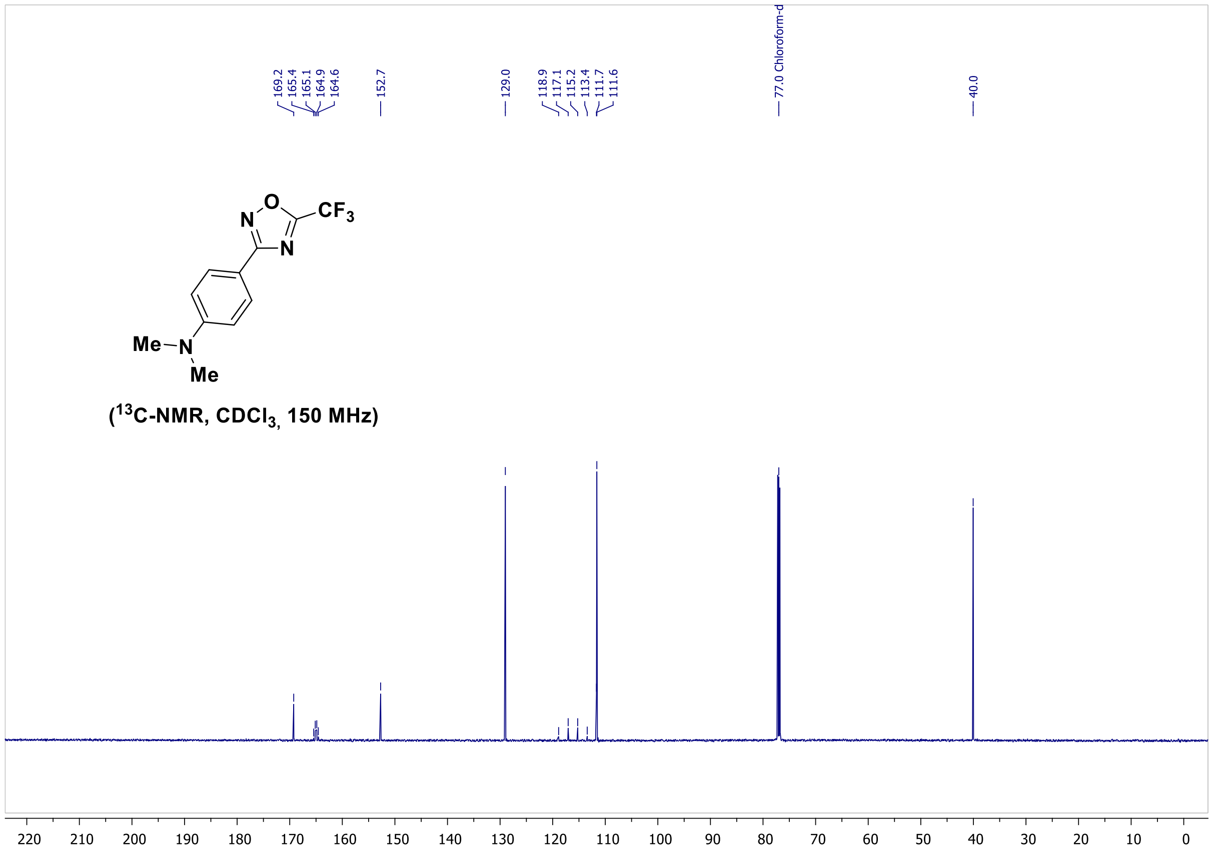

169.2
165.4
165.1
164.9
164.6
152.7
129.0
118.9
117.1
115.2
113.4
111.7
111.6
77.0 Chloroform-d
40.0
N
O
CF3
N
Me
N
Me
(13C-NMR, CDCl3, 150 MHz)
220 210 200 190 180 170 160 150 140 130 120 110 100 90 80 70 60 50 40 30 20 10 0

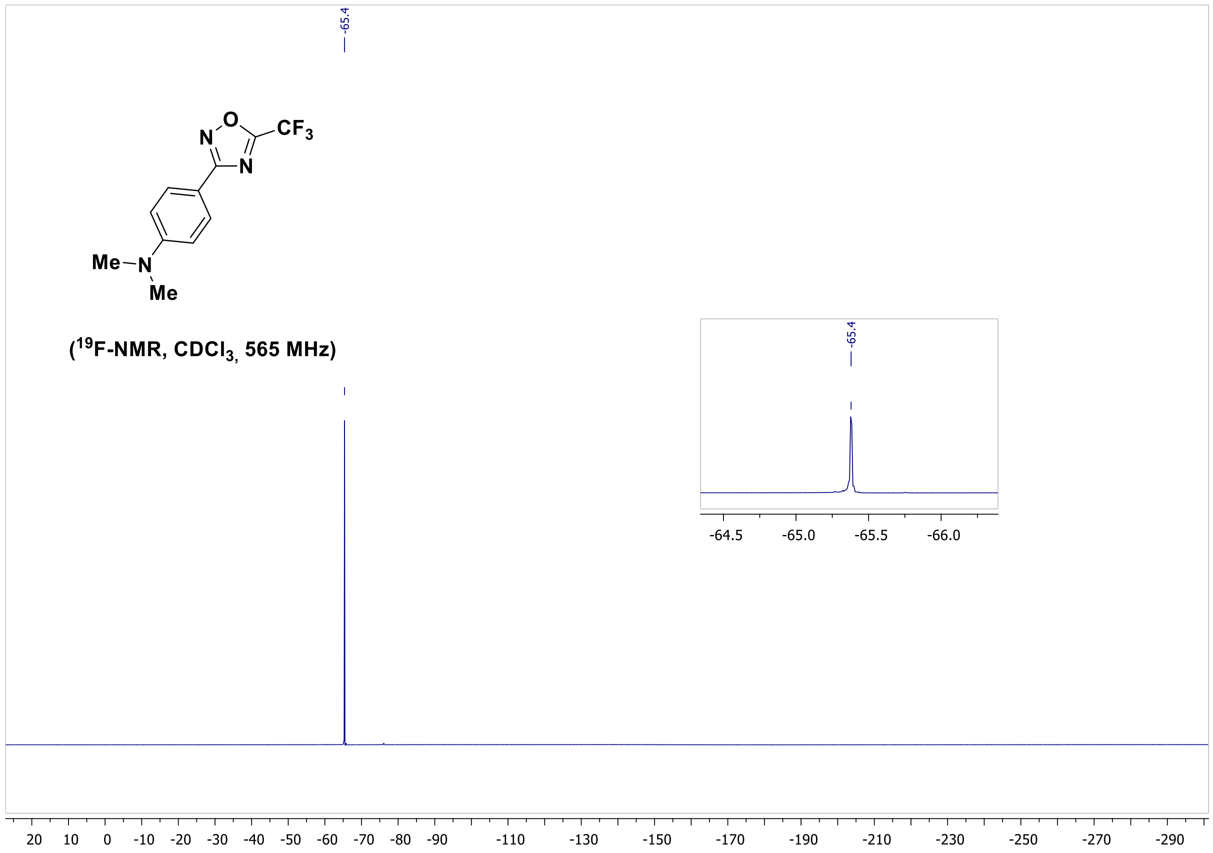

-65.4
N
O
CF3
N
Me
N
Me
(19F-NMR, CDCl3, 565 MHz)
-65.4
-64.5 -65.0 -65.5 -66.0
20 10 0 -10 -20 -30 -40 -50 -60 -70 -80 -90 -110 -130 -150 -170 -190 -210 -230 -250 -270 -290

3-(pyridin-4-yl)-5-(trifluoromethyl)-1,2,4-oxadiazole (24-*sm*)

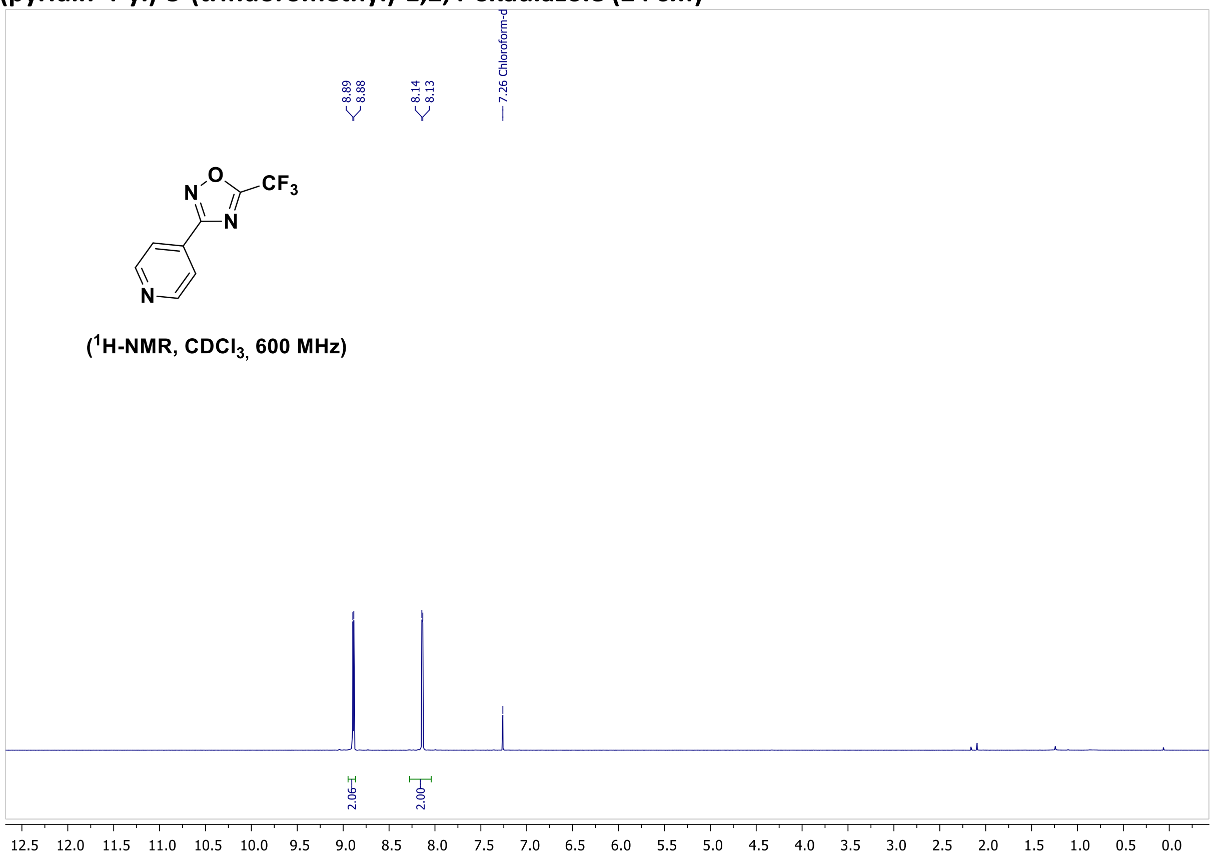

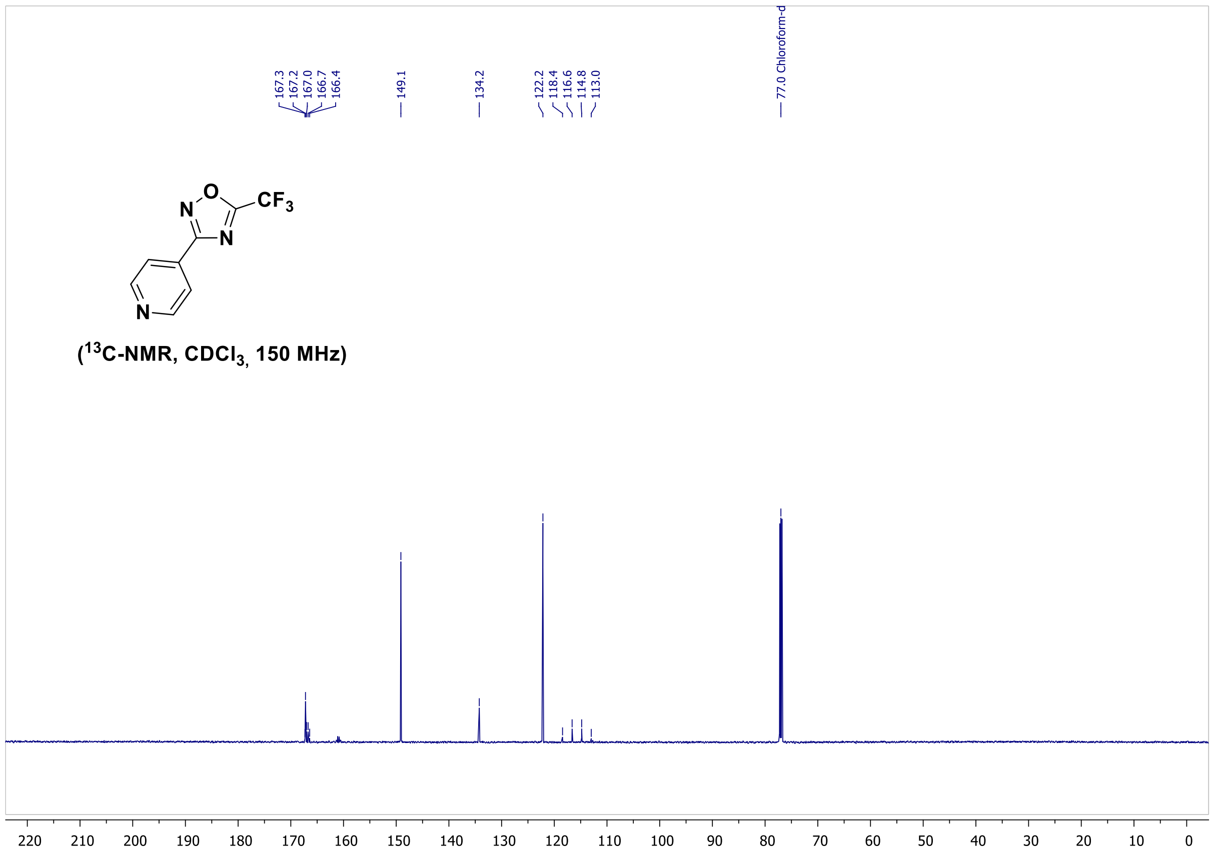

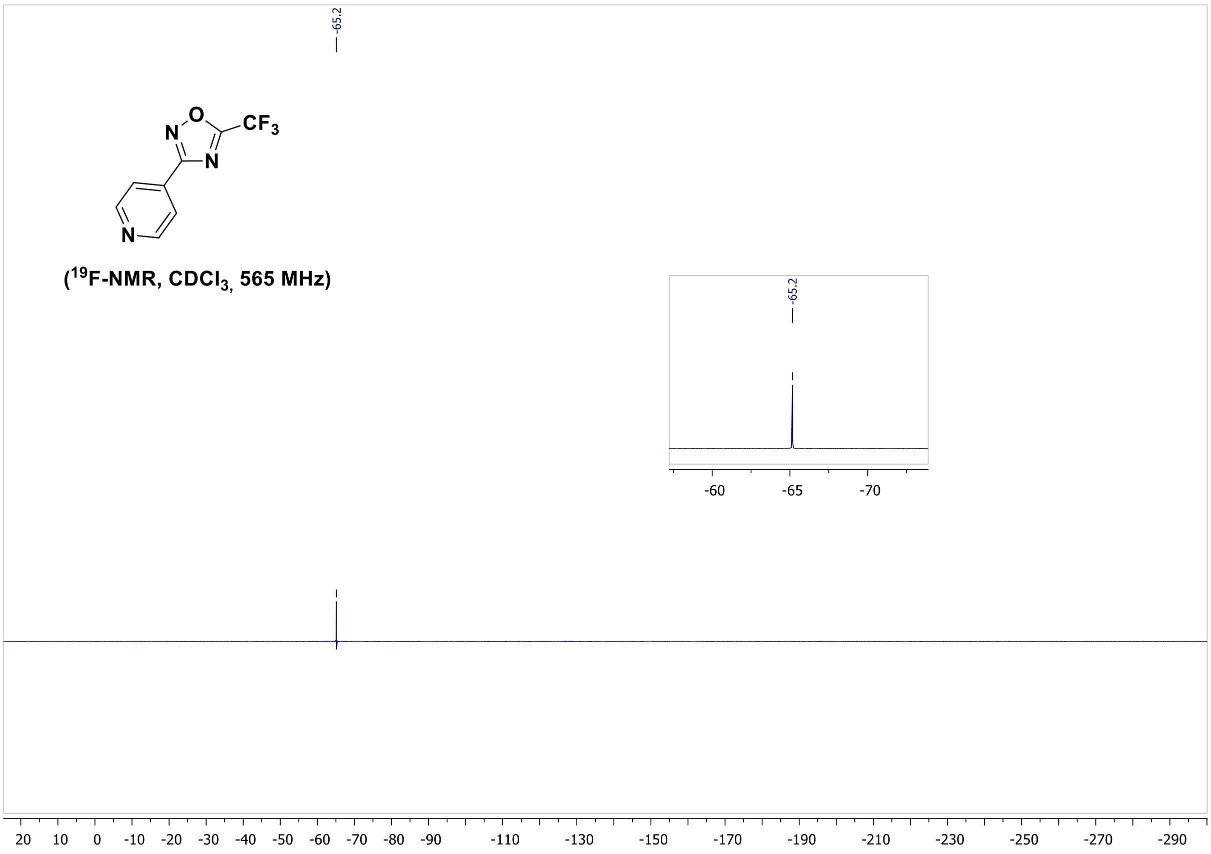

3-(4-bromophenyl)-5-(chloromethyl)-2-methyl-5-(trifluoromethyl)-2,5-dihydro-1,2,4-oxadiazole (2)

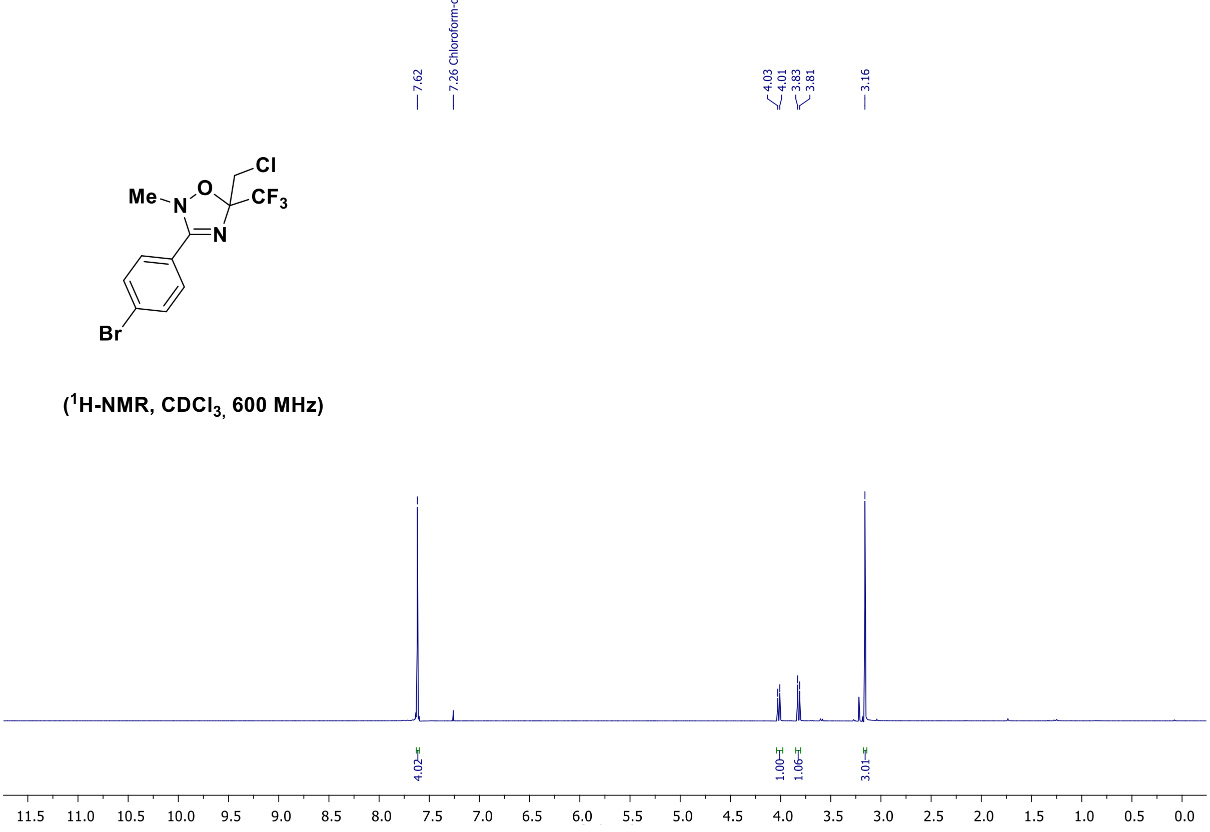

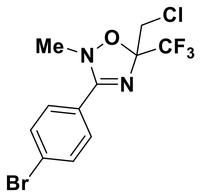

(^{13}C-NMR, $CDCl_3$, 150 MHz)

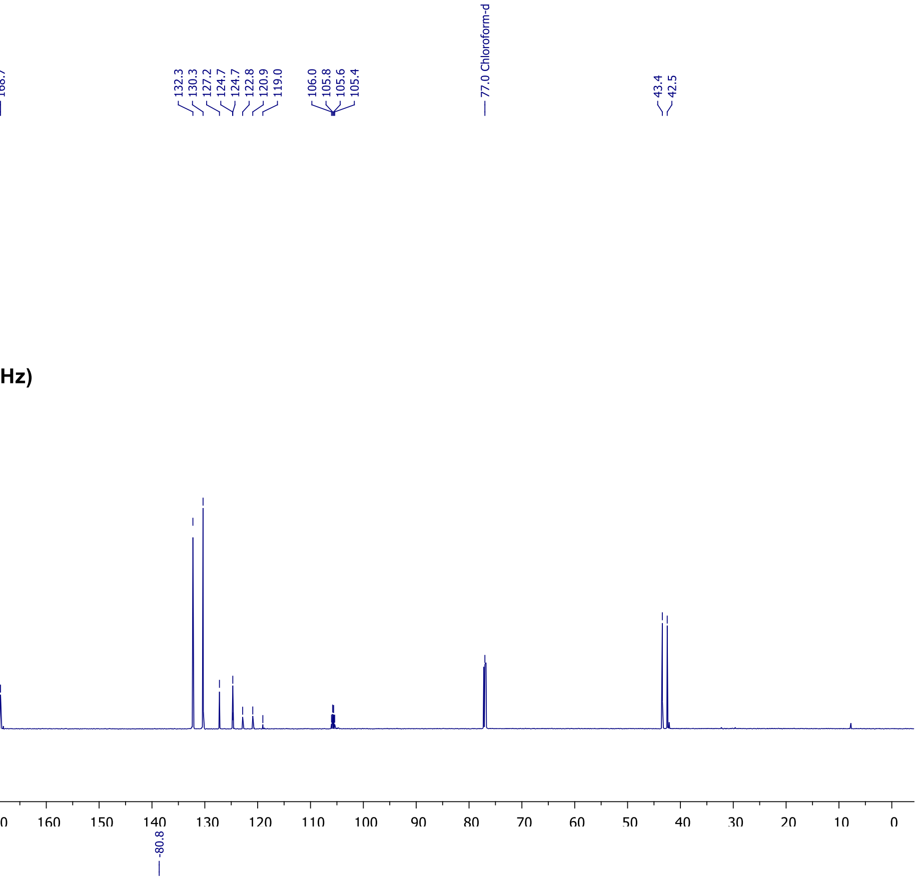

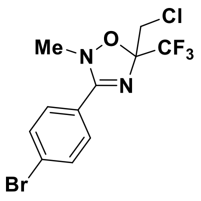

(^{19}F-NMR, $CDCl_3$, 565 MHz)

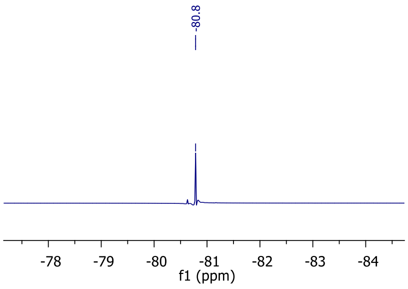

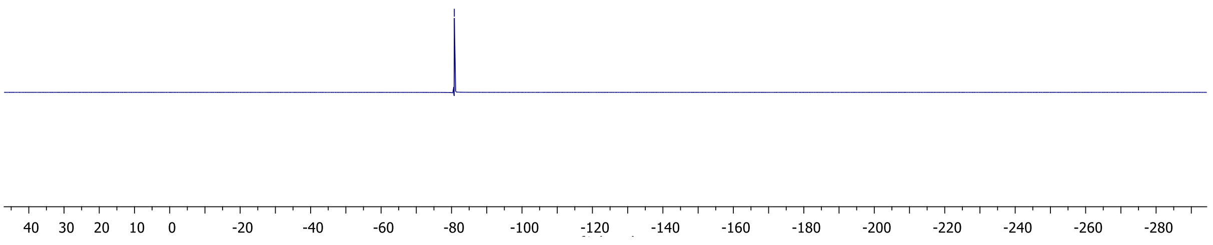

5-(chloromethyl)-3-(4-fluorophenyl)-2-methyl-5-(trifluoromethyl)-2,5-dihydro-1,2,4-oxadiazole (4)

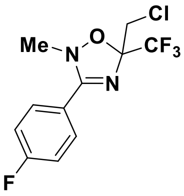

(^{1}H-NMR, $CDCl_3$, 600 MHz)

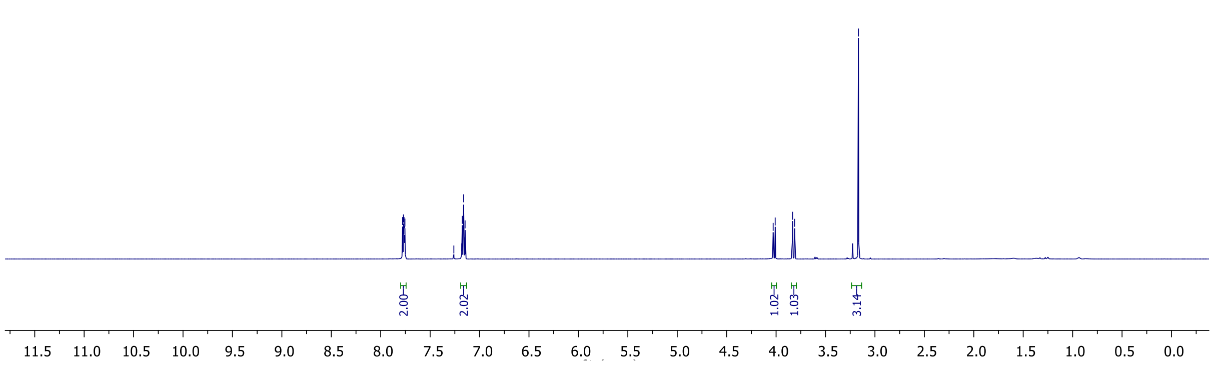

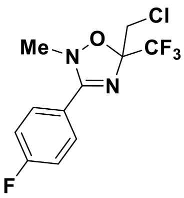

(^{13}C-NMR, $CDCl_3$, 150 MHz)

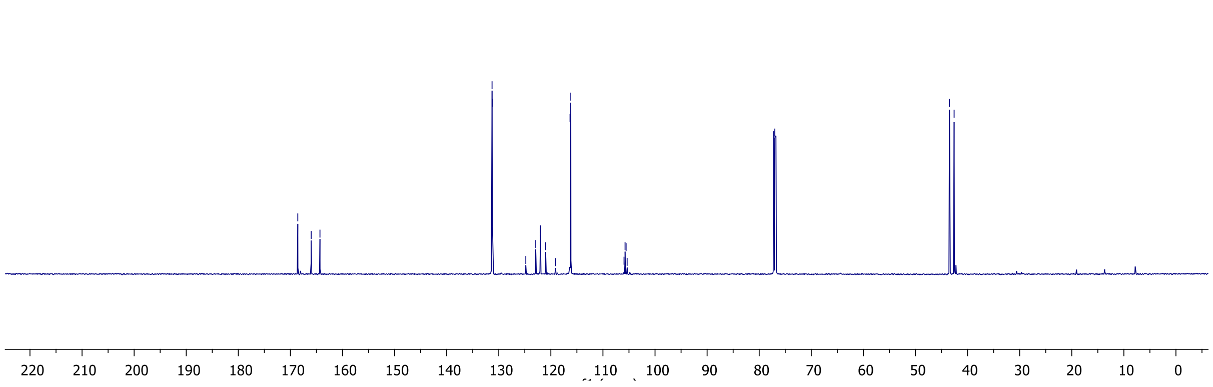

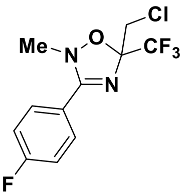

(^{19}F-NMR, $CDCl_3$, 565 MHz)

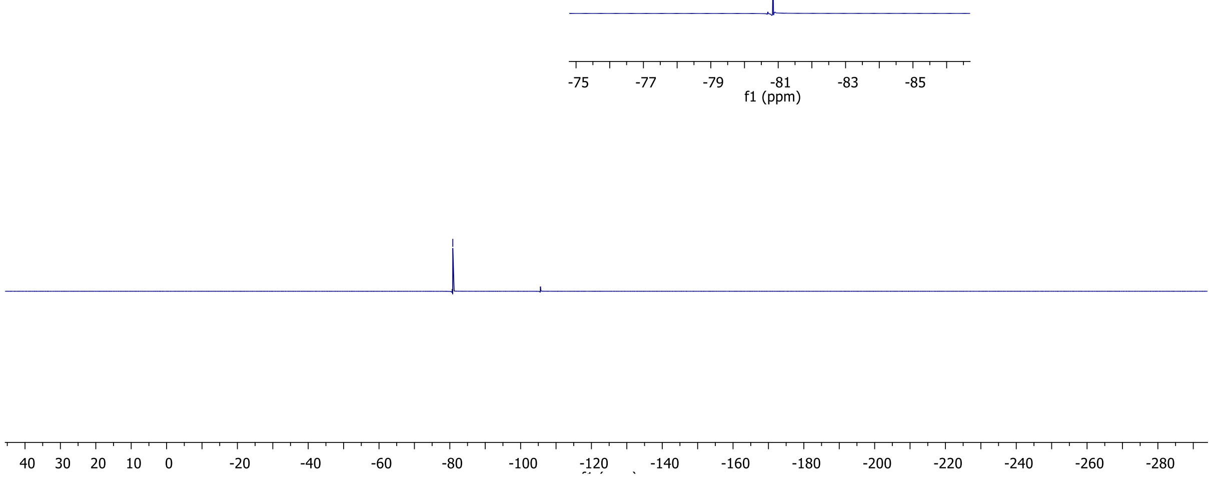

Isopropyl 3-(5-(chloromethyl)-2-methyl-5-(trifluoromethyl)-2,5-dihydro-1,2,4-oxadiazol-3-yl)benzoate (5)

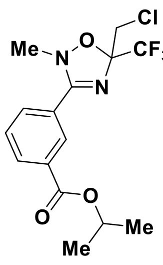

(^{1}H-NMR, $CDCl_3$, 400 MHz)

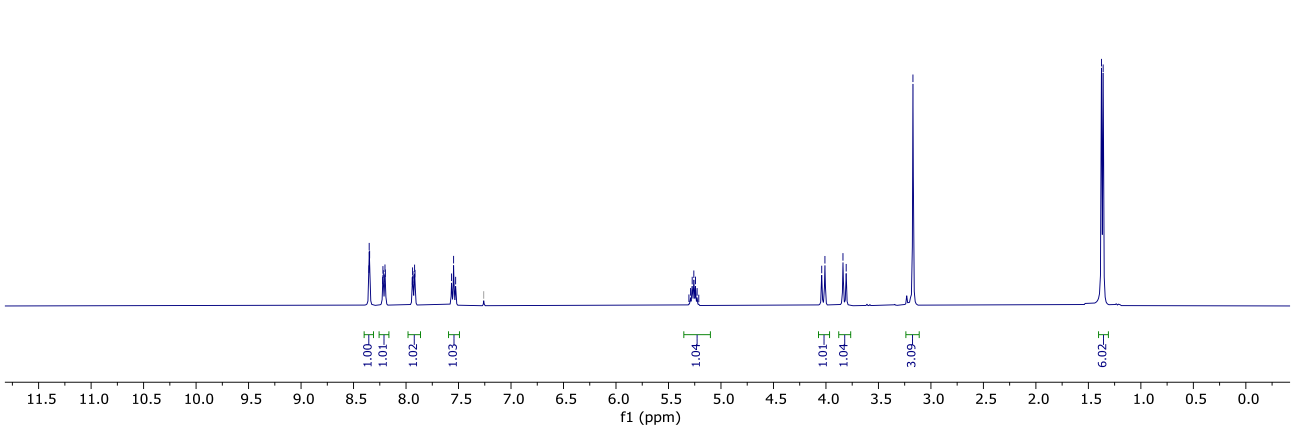

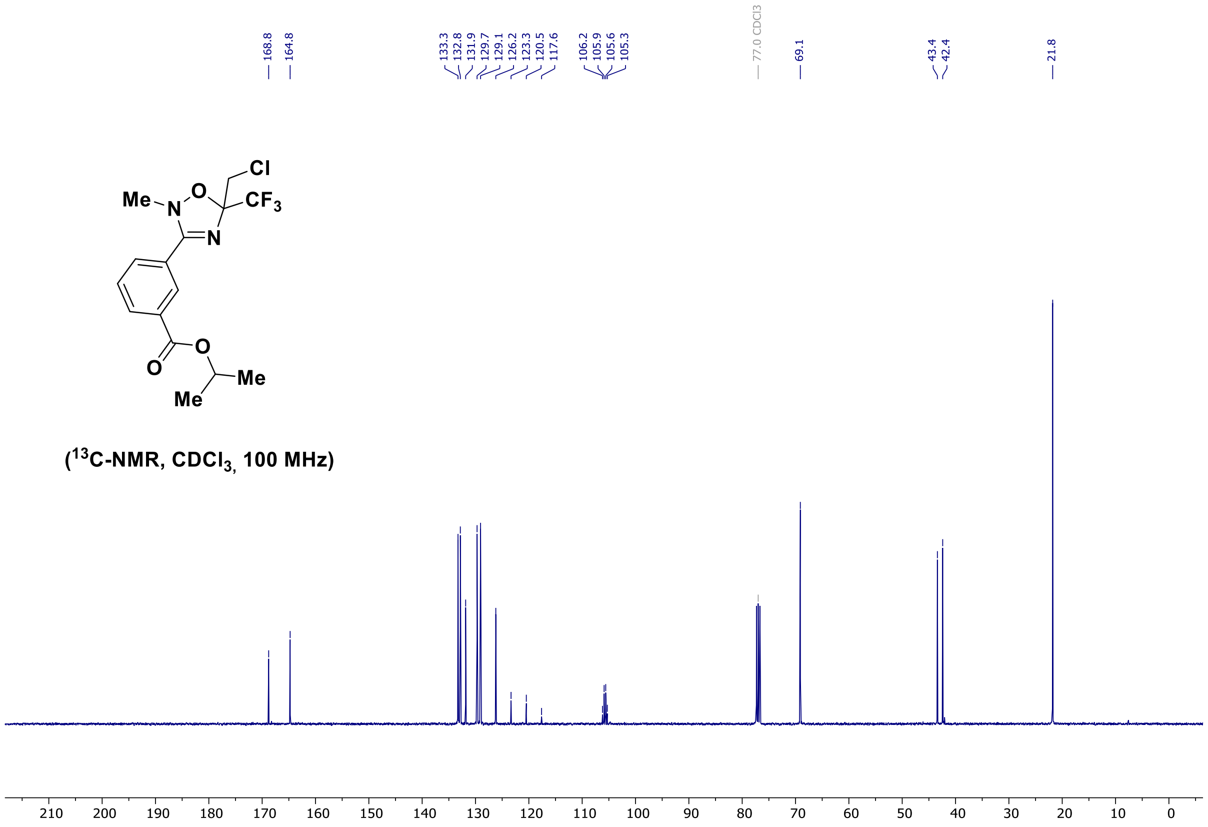

(^{13}C-NMR, $CDCl_3$, 100 MHz)

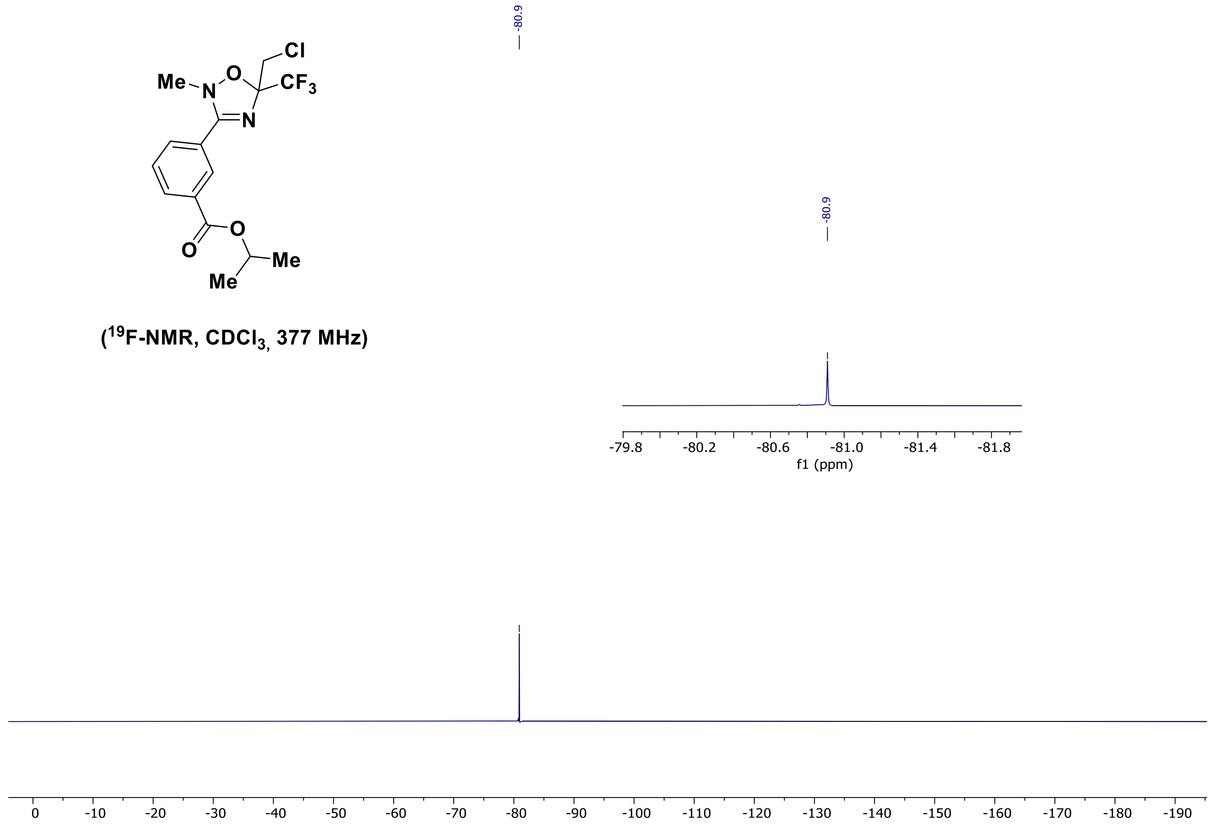

(^{19}F-NMR, $CDCl_3$, 377 MHz)

4-(5-(chloromethyl)-2-methyl-5-(trifluoromethyl)-2,5-dihydro-1,2,4-oxadiazol-3-yl) benzonitrile (6)

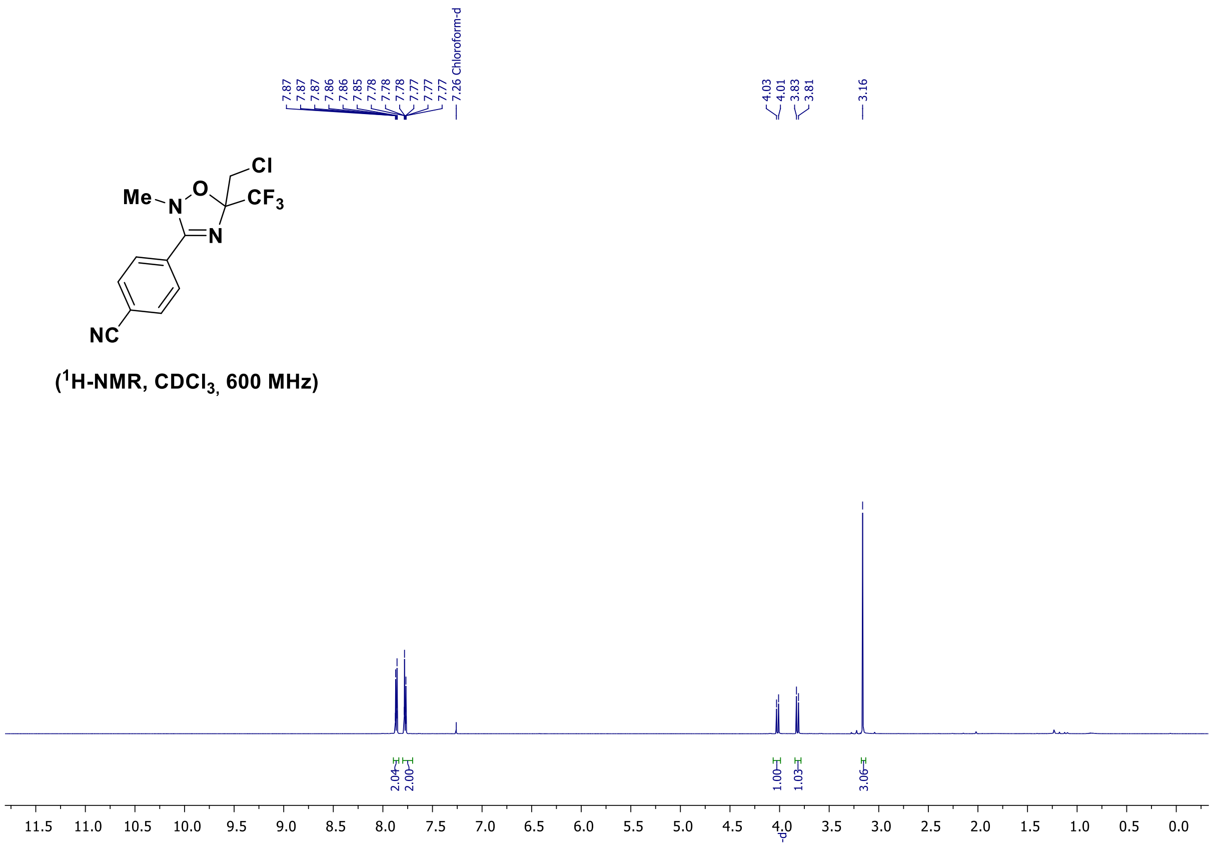

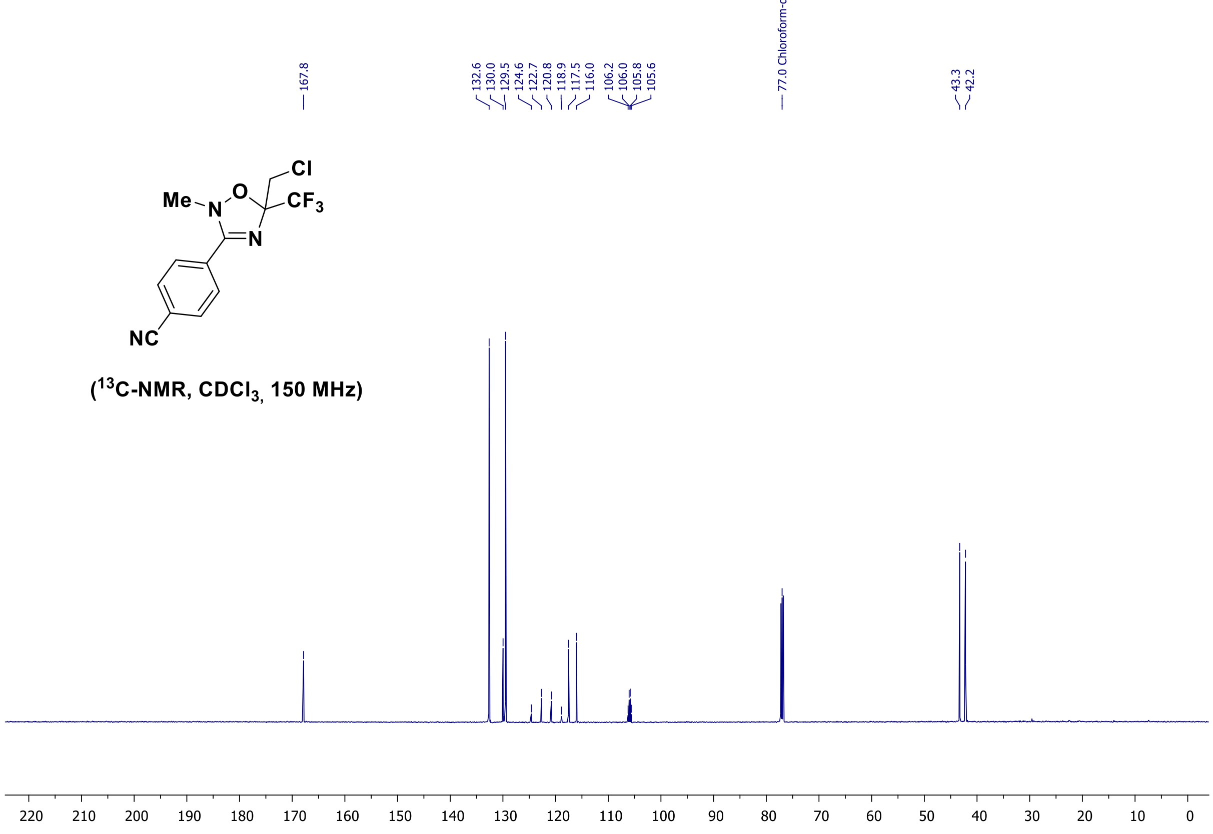

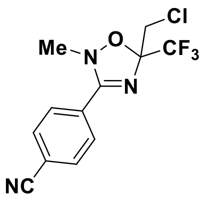

(^{19}F-NMR, $CDCl_3$, 565 MHz)

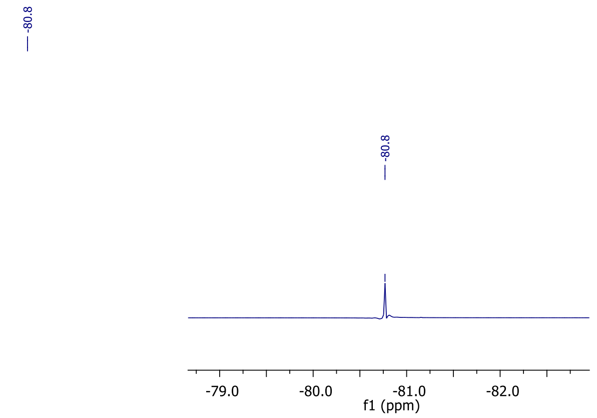
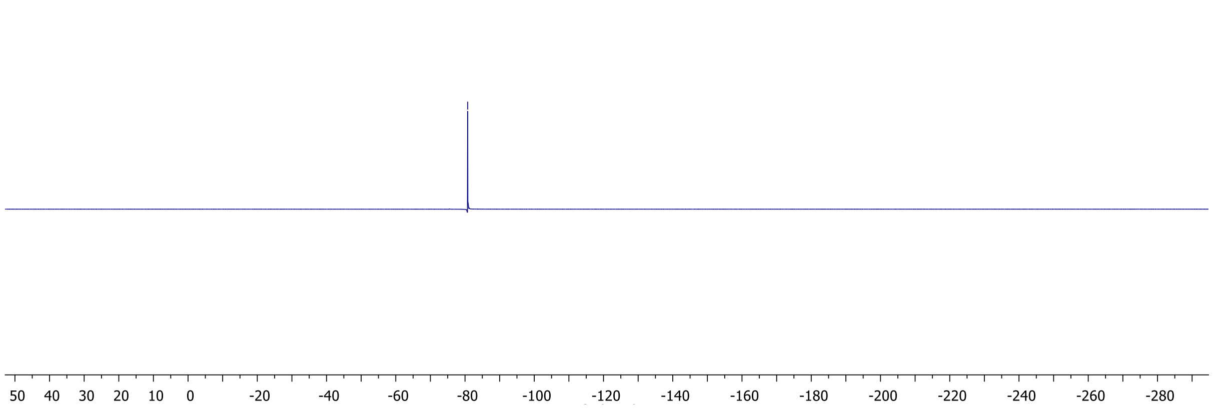

5-(chloromethyl)-2-methyl-3-(pyridin-2-yl)-5-(trifluoromethyl)-2,5-dihydro-1,2,4-oxadiazole (7)

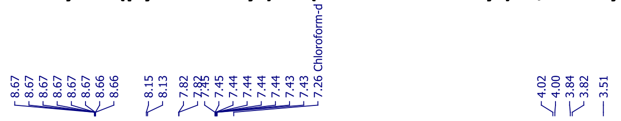
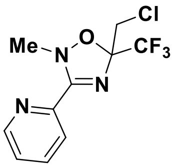

(^{1}H-NMR, $CDCl_3$, 600 MHz)

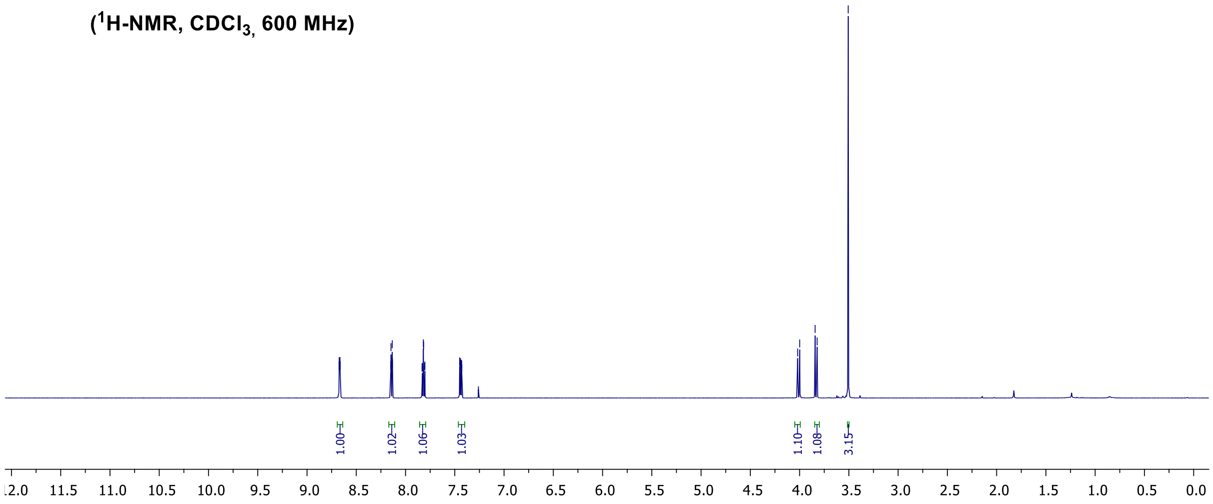

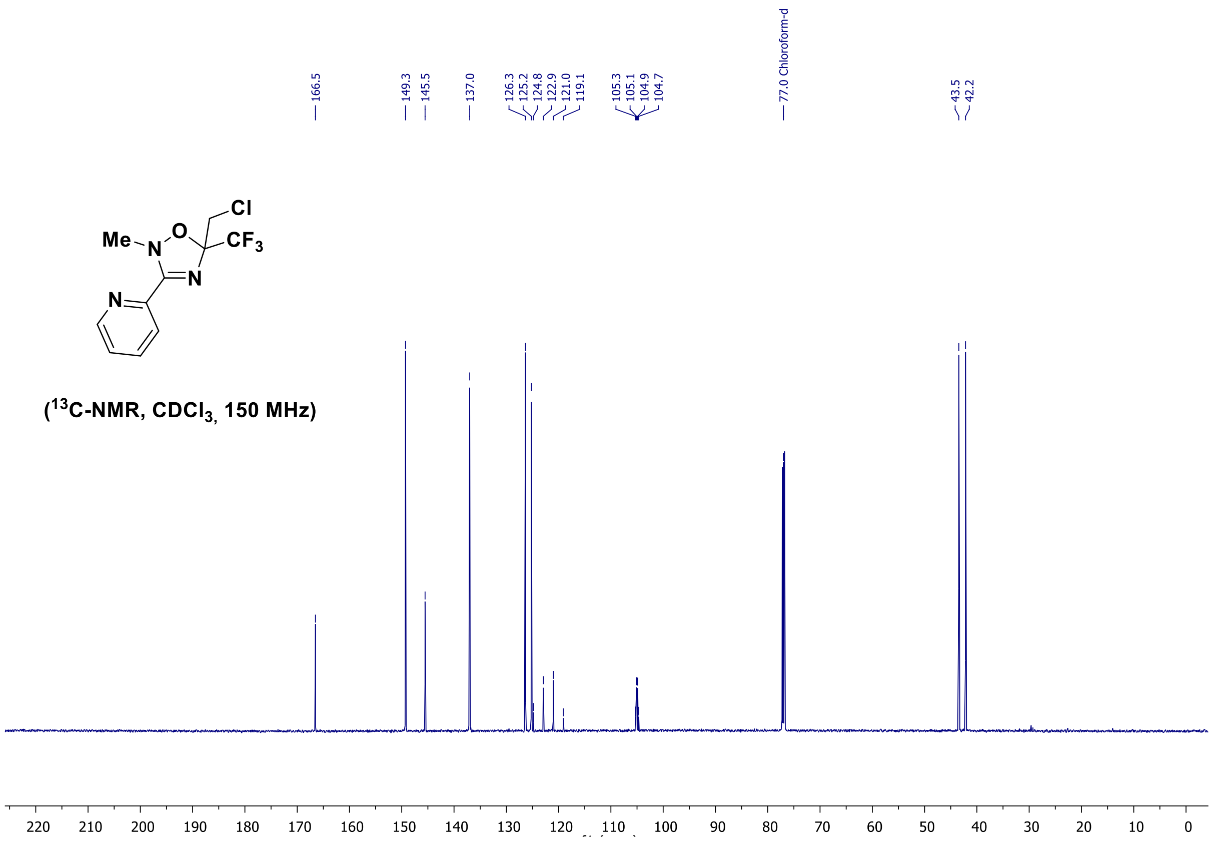
166.5
149.3
145.5
137.0
126.3
125.2
124.8
122.9
121.0
119.1
105.3
105.1
104.9
104.7
77.0 Chloroform-d
43.5
42.2
Me
N
O
Cl
CF3
N
N
(13C-NMR, CDCl3, 150 MHz)
220 210 200 190 180 170 160 150 140 130 120 110 100 90 80 70 60 50 40 30 20 10 0

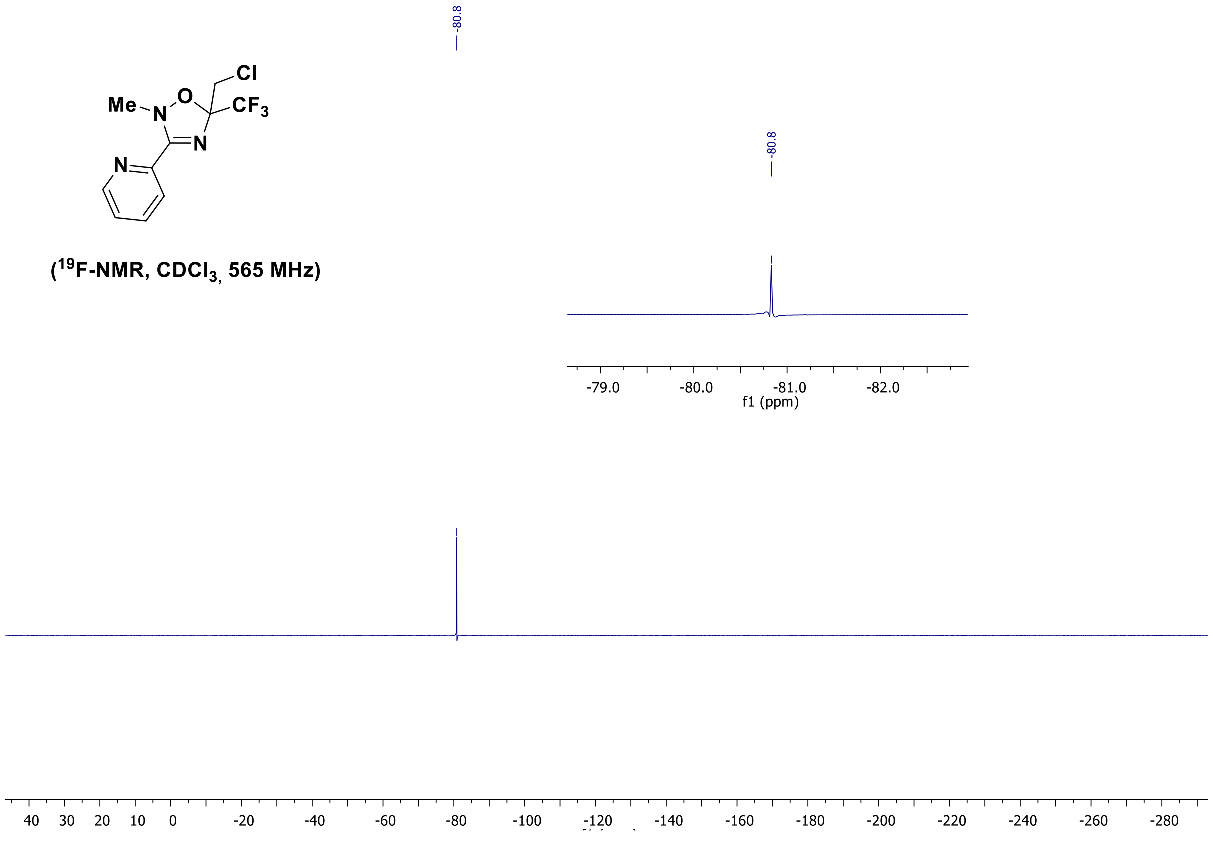
-80.8
Me
N
O
Cl
CF3
N
N
(19F-NMR, CDCl3, 565 MHz)
-80.8
-79.0 -80.0 -81.0 -82.0
f1 (ppm)
40 30 20 10 0 -20 -40 -60 -80 -100 -120 -140 -160 -180 -200 -220 -240 -260 -280

5-(chloromethyl)-2-methyl-3-phenyl-5-(trifluoromethyl)-2,5-dihydro-1,2,4-oxadiazole (8)

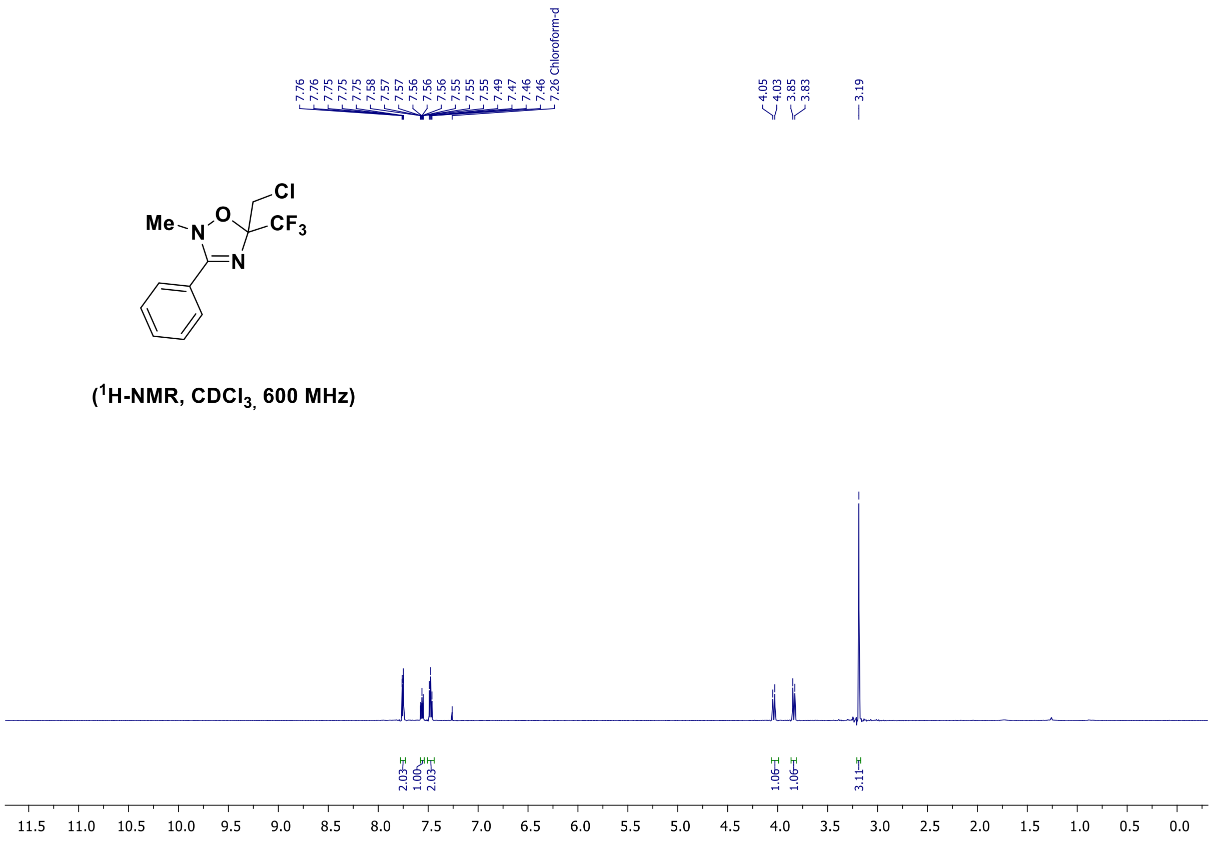

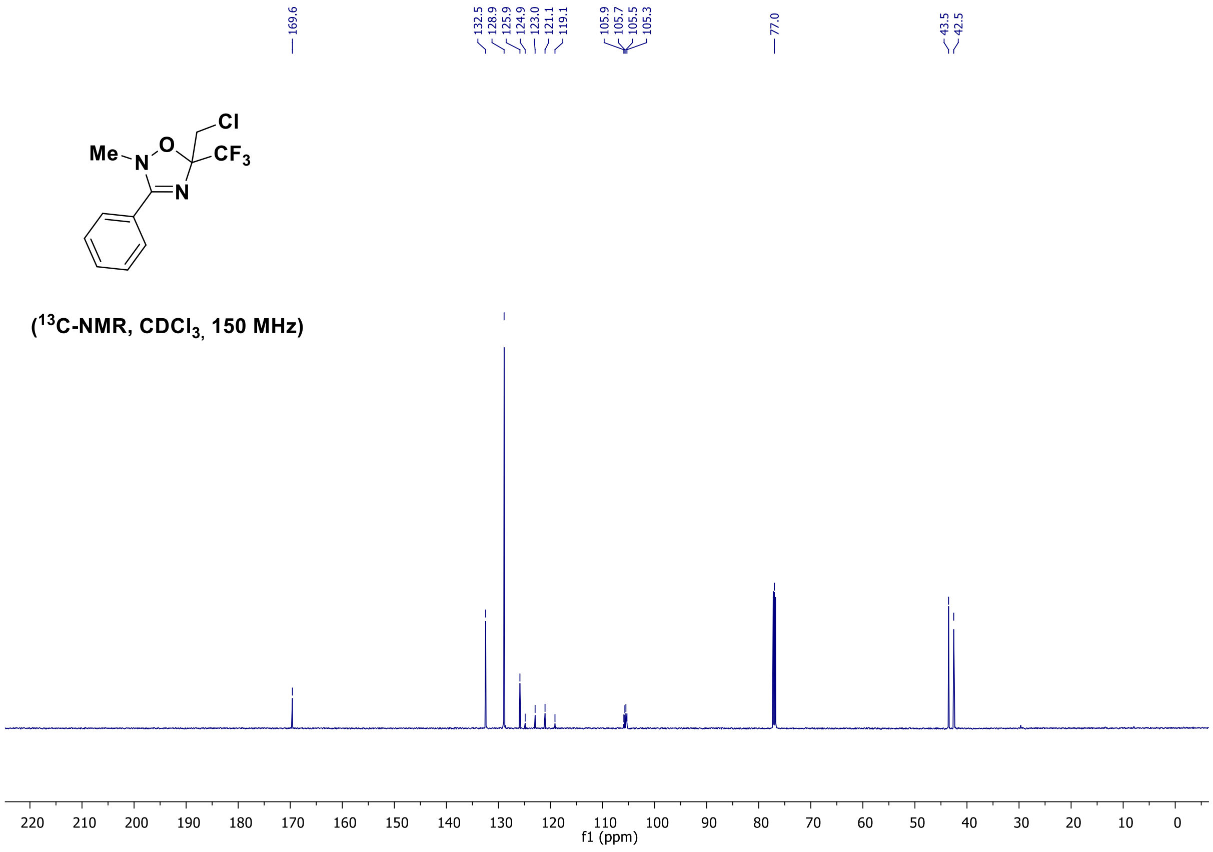

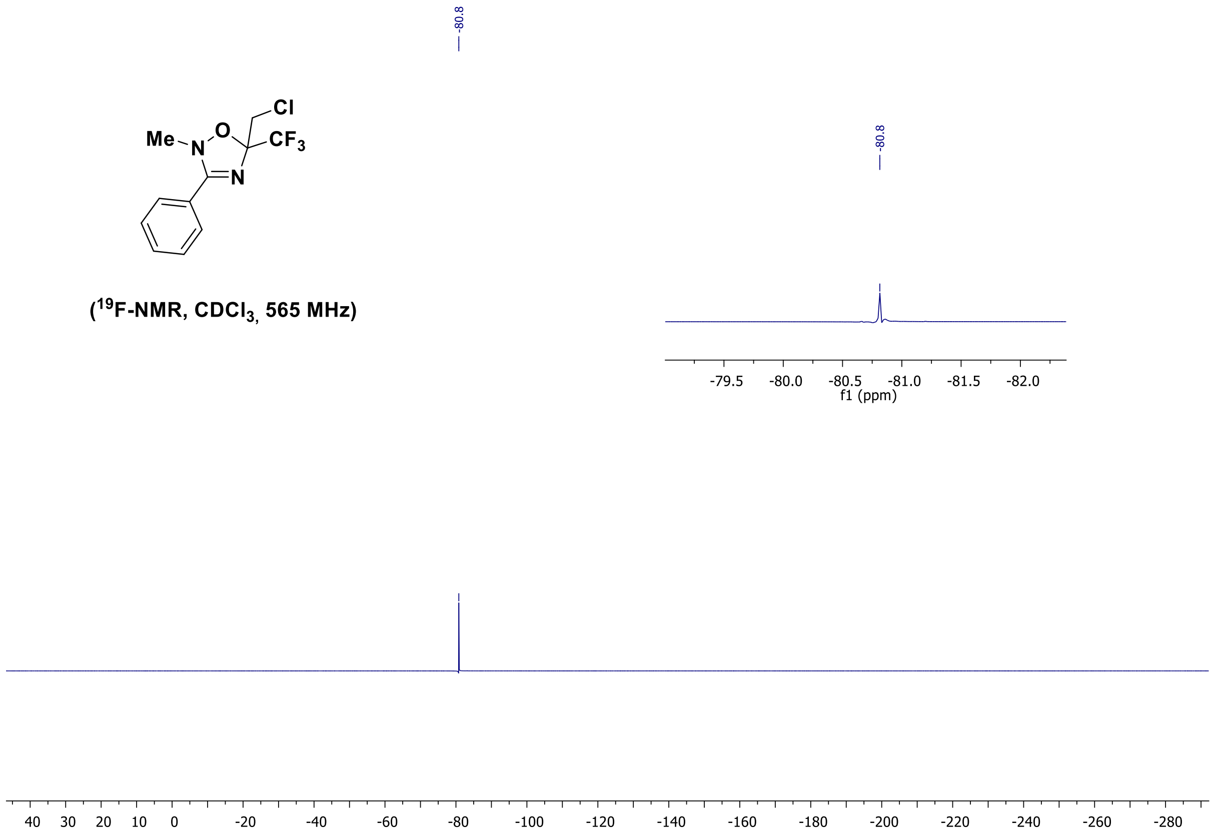

3-(4-chlorophenyl)-5-(fluoromethyl)-2-methyl-5-(trifluoromethyl)-2,5-dihydro-1,2,4-oxadiazole (9)

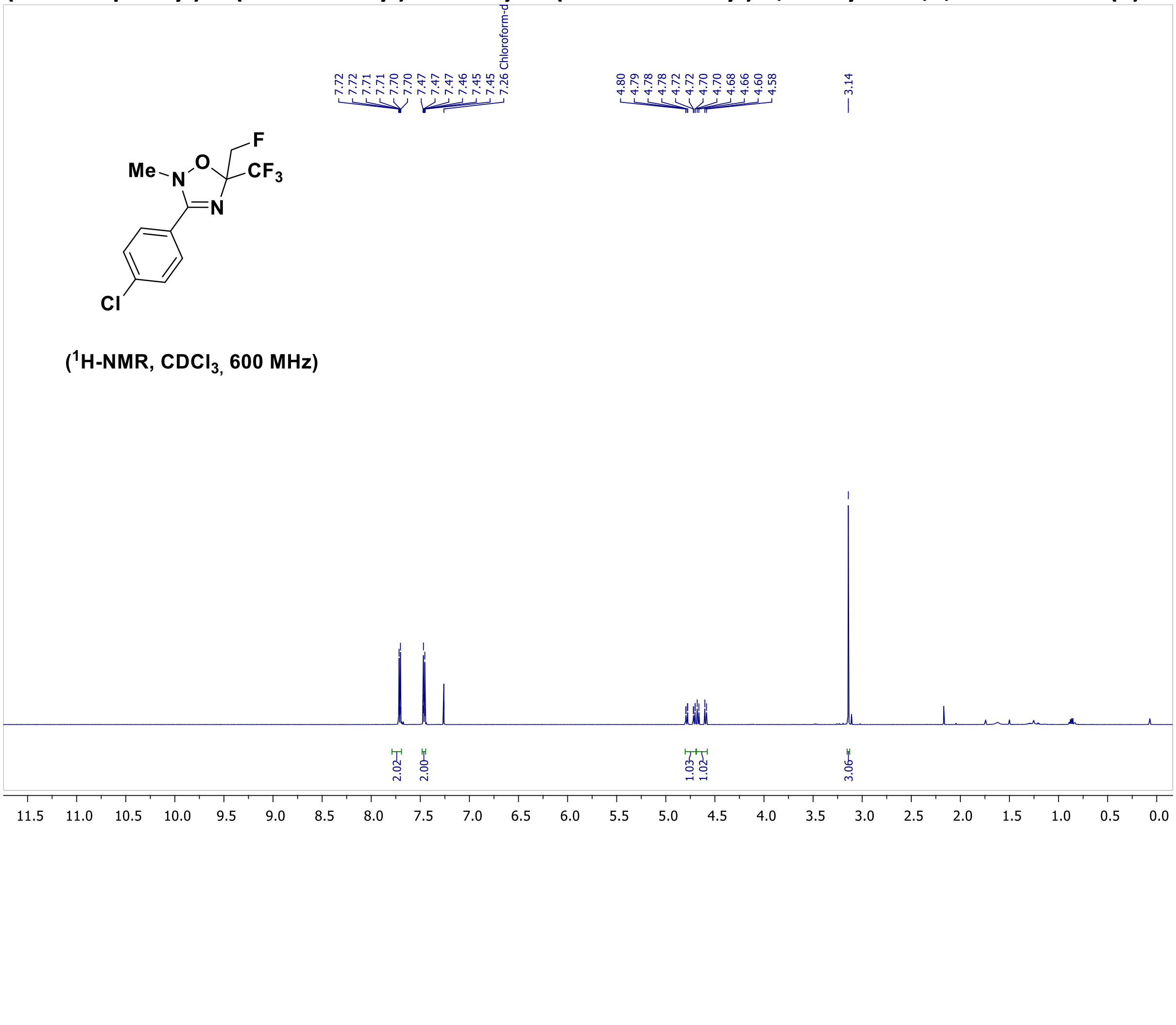

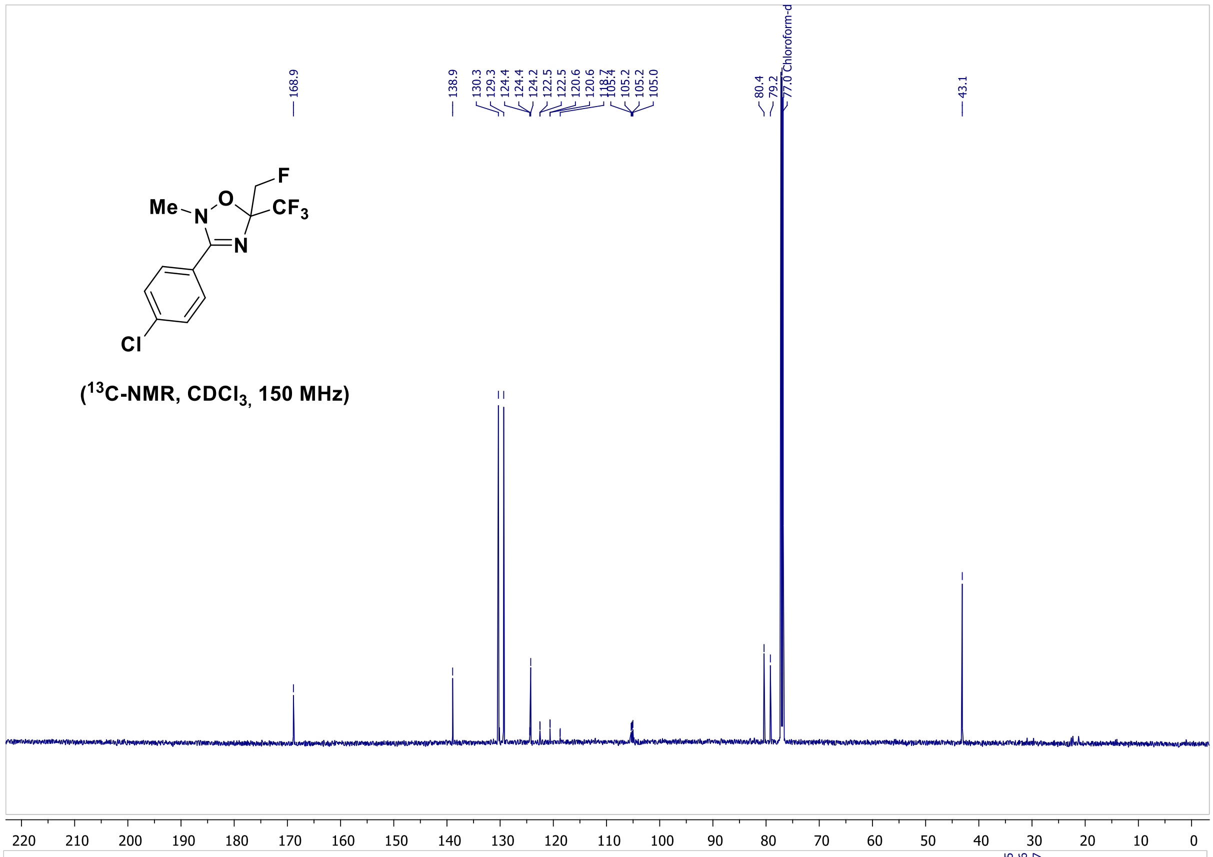
168.9
138.9
130.3
129.3
124.4
124.4
124.2
122.5
122.5
120.6
120.6
118.7
105.4
105.2
105.2
105.0
80.4
79.2
77.0 Chloroform-d
43.1
Me
N
O
F
CF3
N
Cl
(13C-NMR, CDCl3, 150 MHz)
220 210 200 190 180 170 160 150 140 130 120 110 100 90 80 70 60 50 40 30 20 10 0

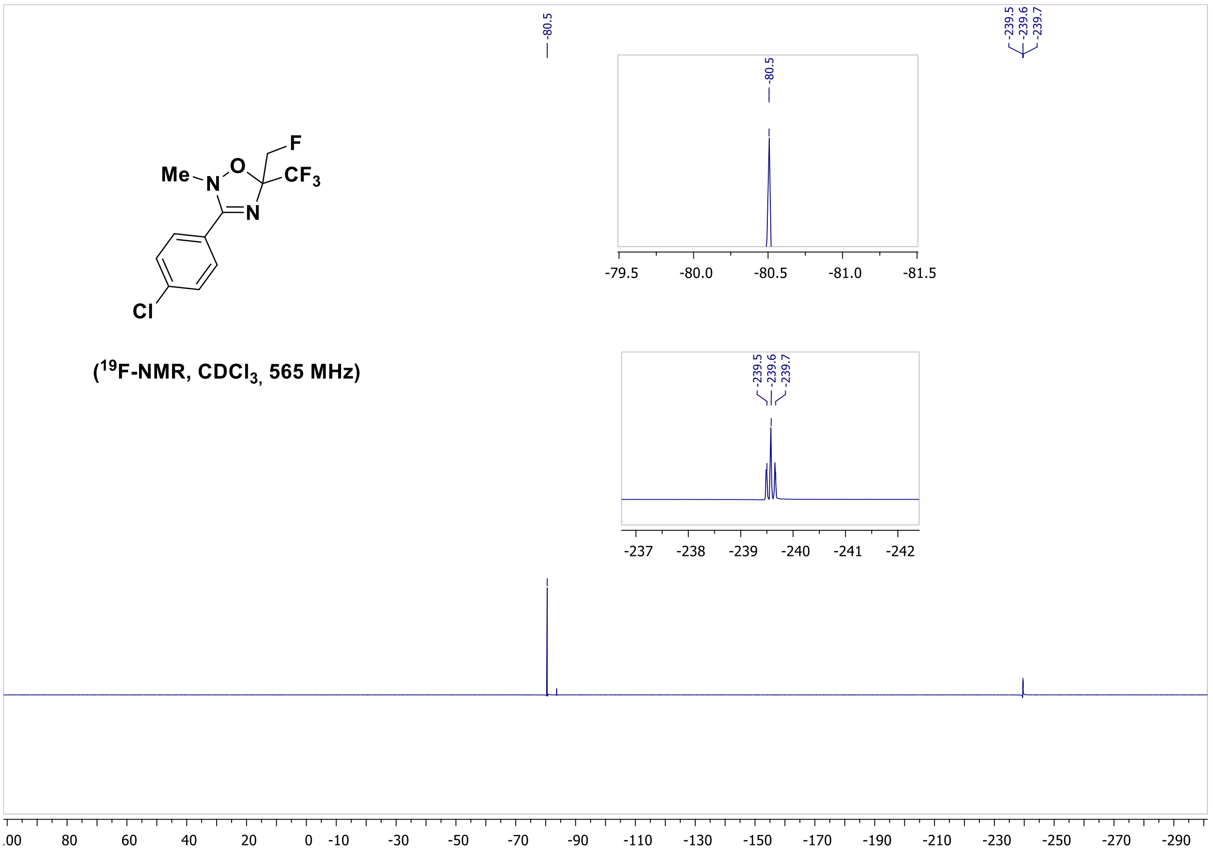
-80.5
-239.5
-239.6
-239.7
Me
N
O
F
CF3
N
Cl
(19F-NMR, CDCl3, 565 MHz)
-79.5 -80.0 -80.5 -81.0 -81.5
-237 -238 -239 -240 -241 -242
00 80 60 40 20 0 -10 -30 -50 -70 -90 -110 -130 -150 -170 -190 -210 -230 -250 -270 -290

5-(fluoromethyl)-2-methyl-5-(trifluoromethyl)-3-(4-(trifluoromethyl)phenyl)-2,5-dihydro-1,2,4-oxadiazole (10)

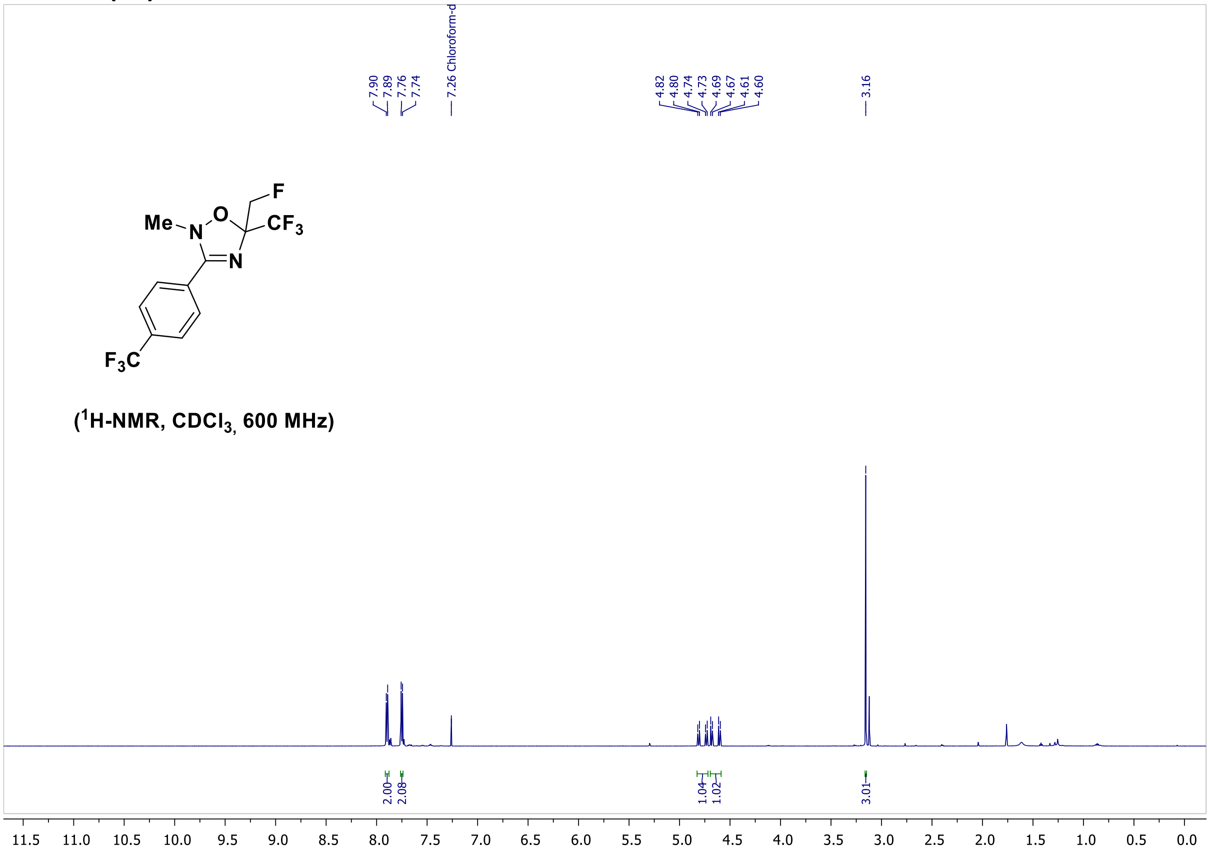

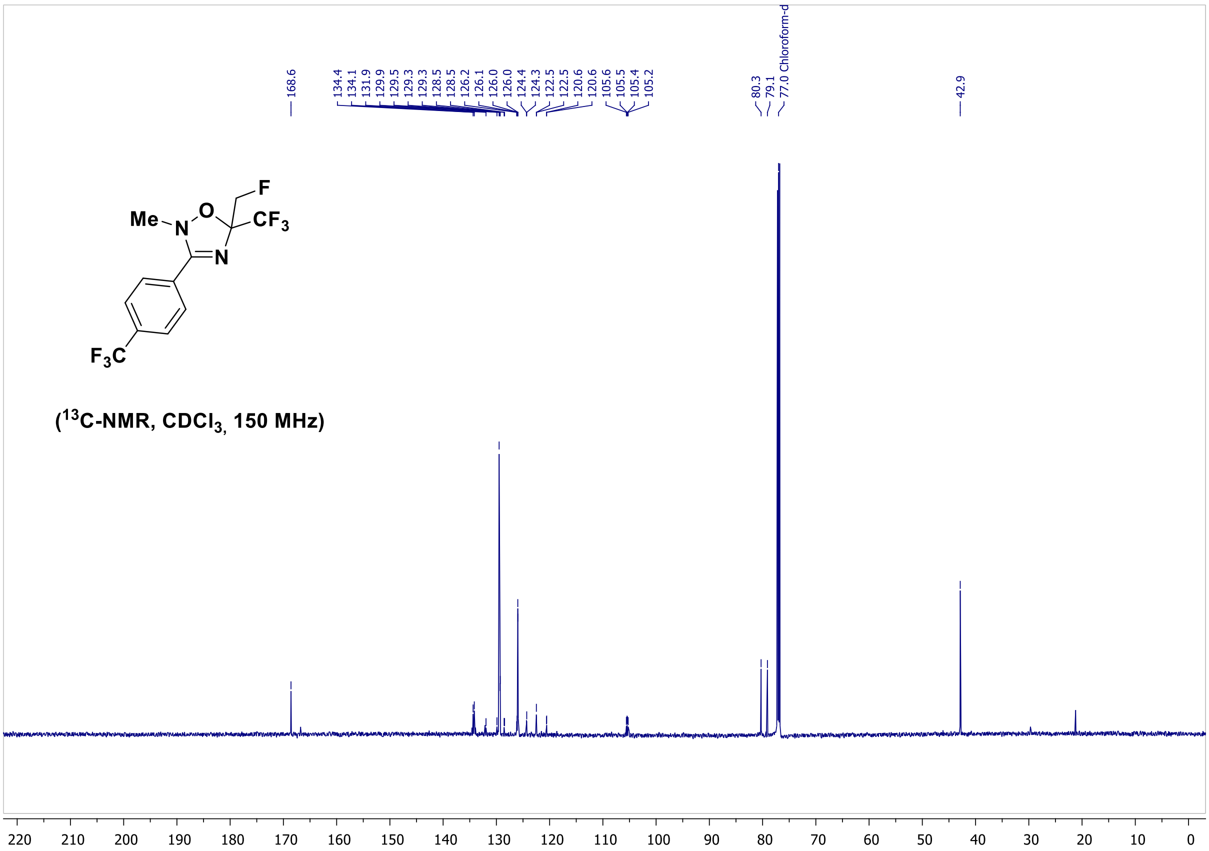

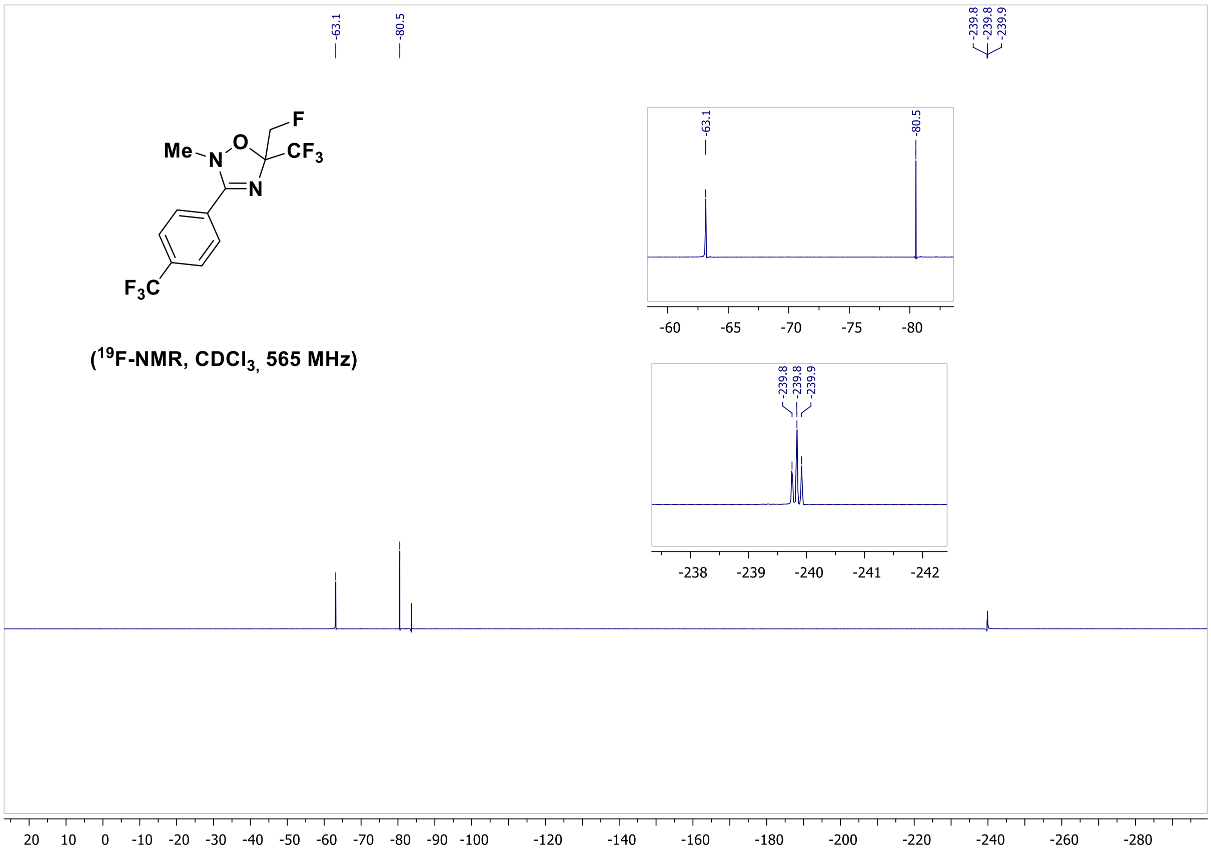

4-(5-(fluoromethyl)-2-methyl-5-(trifluoromethyl)-2,5-dihydro-1,2,4-oxadiazol-3-yl)benzonitrile (11)

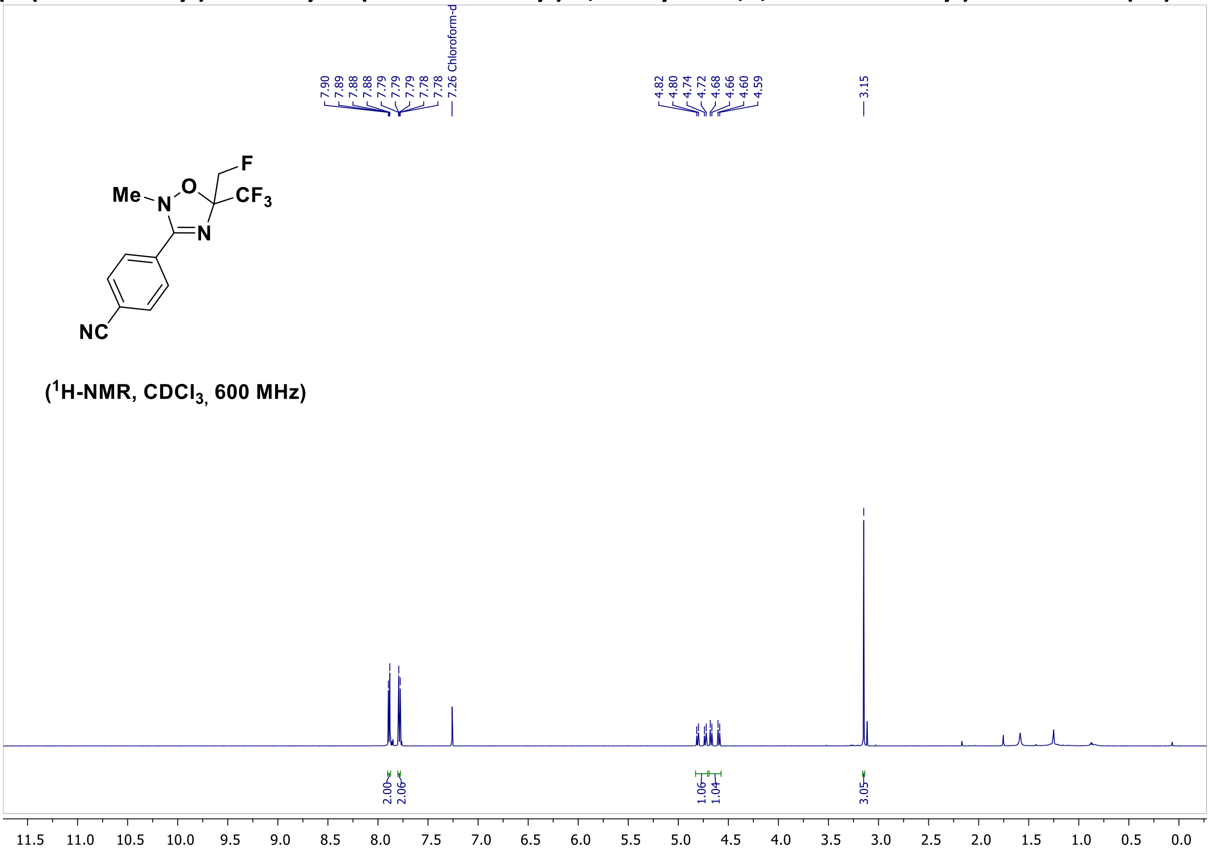

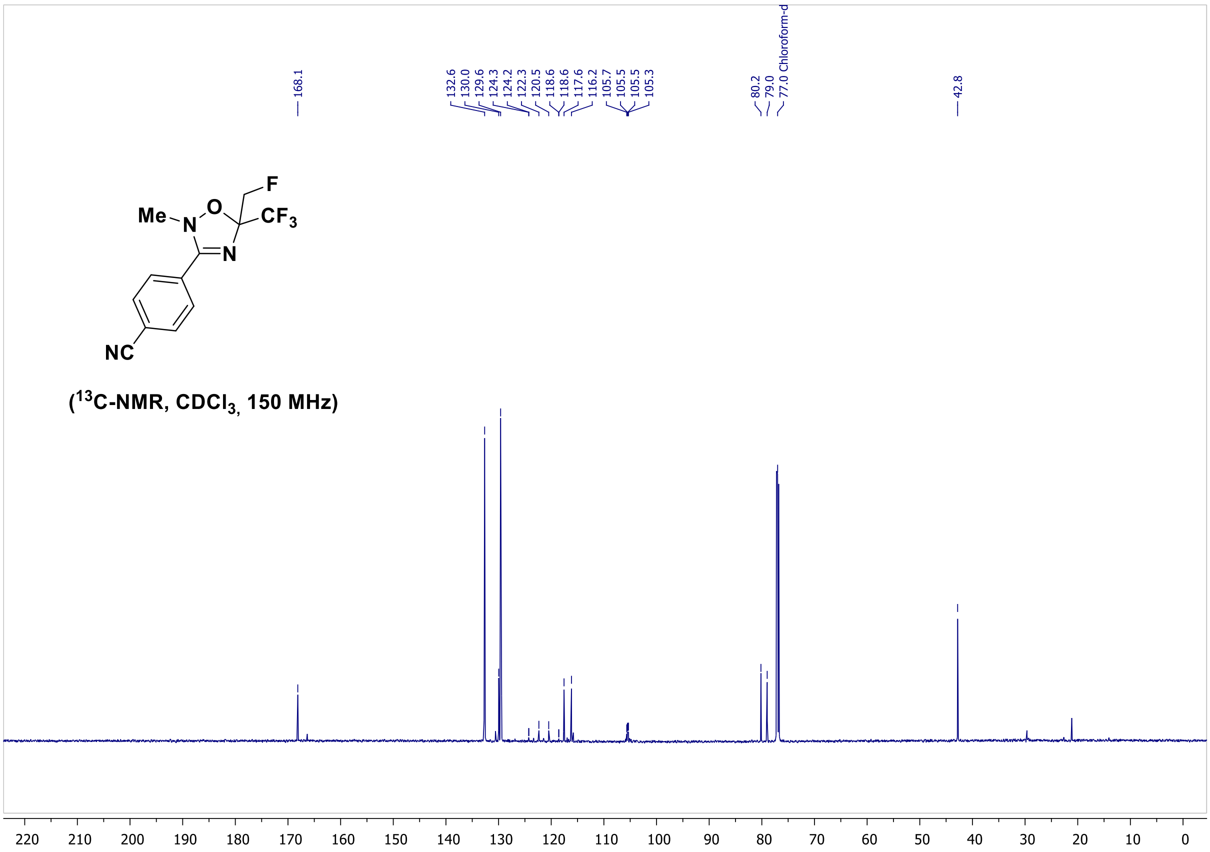
168.1
132.6
130.0
129.6
124.3
124.2
122.3
120.5
118.6
118.6
117.6
116.2
105.7
105.5
105.5
105.3
80.2
79.0
77.0 Chloroform-d
42.8
F
Me
N
O
CF3
N
NC
(13C-NMR, CDCl3, 150 MHz)
220 210 200 190 180 170 160 150 140 130 120 110 100 90 80 70 60 50 40 30 20 10 0

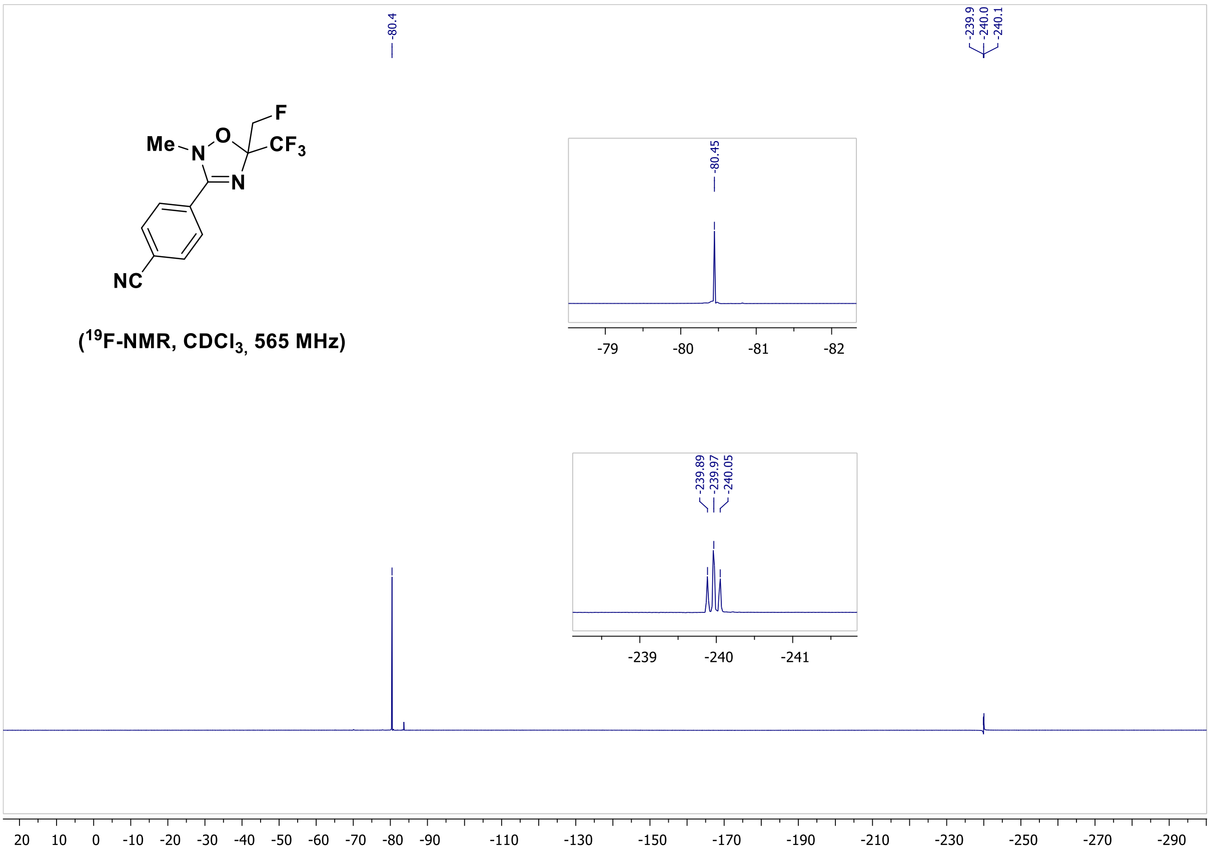
-80.4
-239.9
-240.0
-240.1
F
Me
N
O
CF3
N
NC
(19F-NMR, CDCl3, 565 MHz)
-80.45
-79 -80 -81 -82
-239.89
-239.97
-240.05
-239 -240 -241
20 10 0 -10 -20 -30 -40 -50 -60 -70 -80 -90 -110 -130 -150 -170 -190 -210 -230 -250 -270 -290

5-(fluoromethyl)-2-methyl-3-(4-nitrophenyl)-5-(trifluoromethyl)-2,5-dihydro-1,2,4-oxadiazole (12)

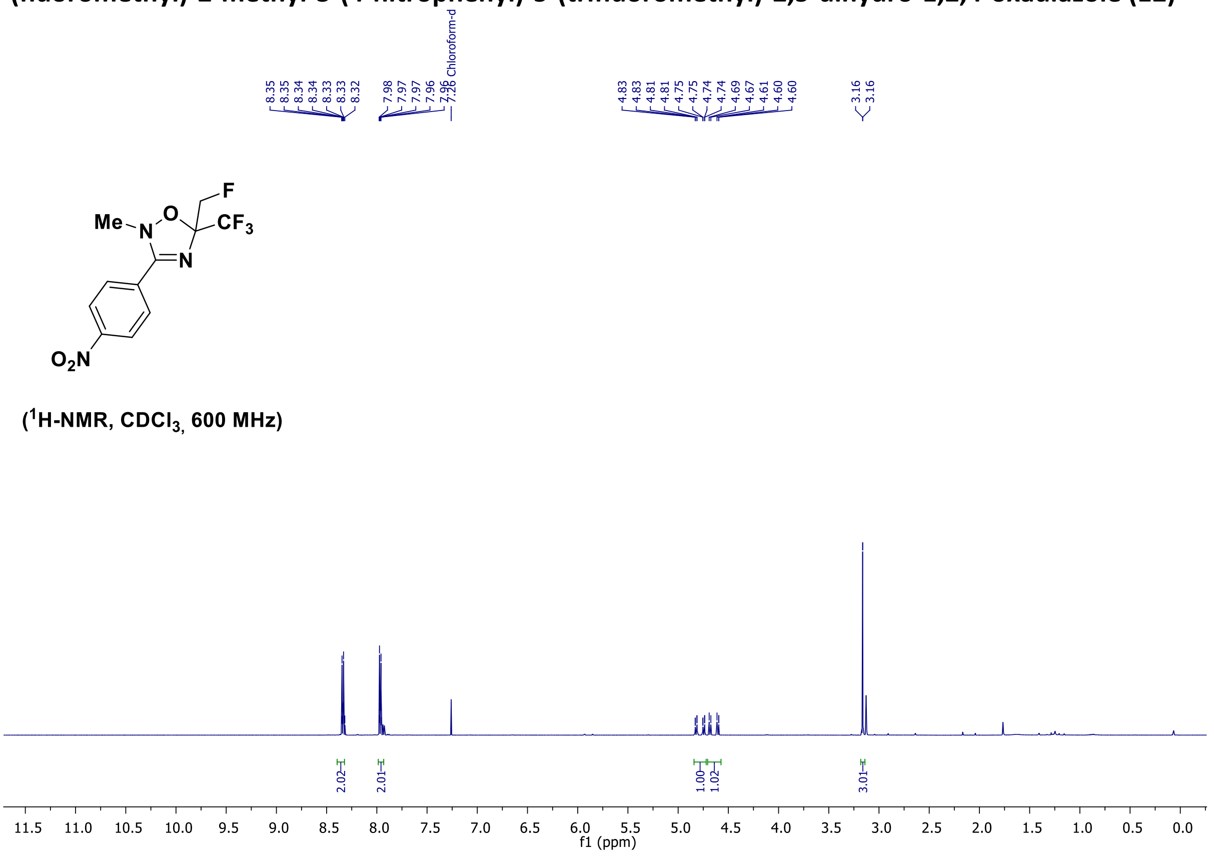

(^{1}H-NMR, $CDCl_3$, 600 MHz)

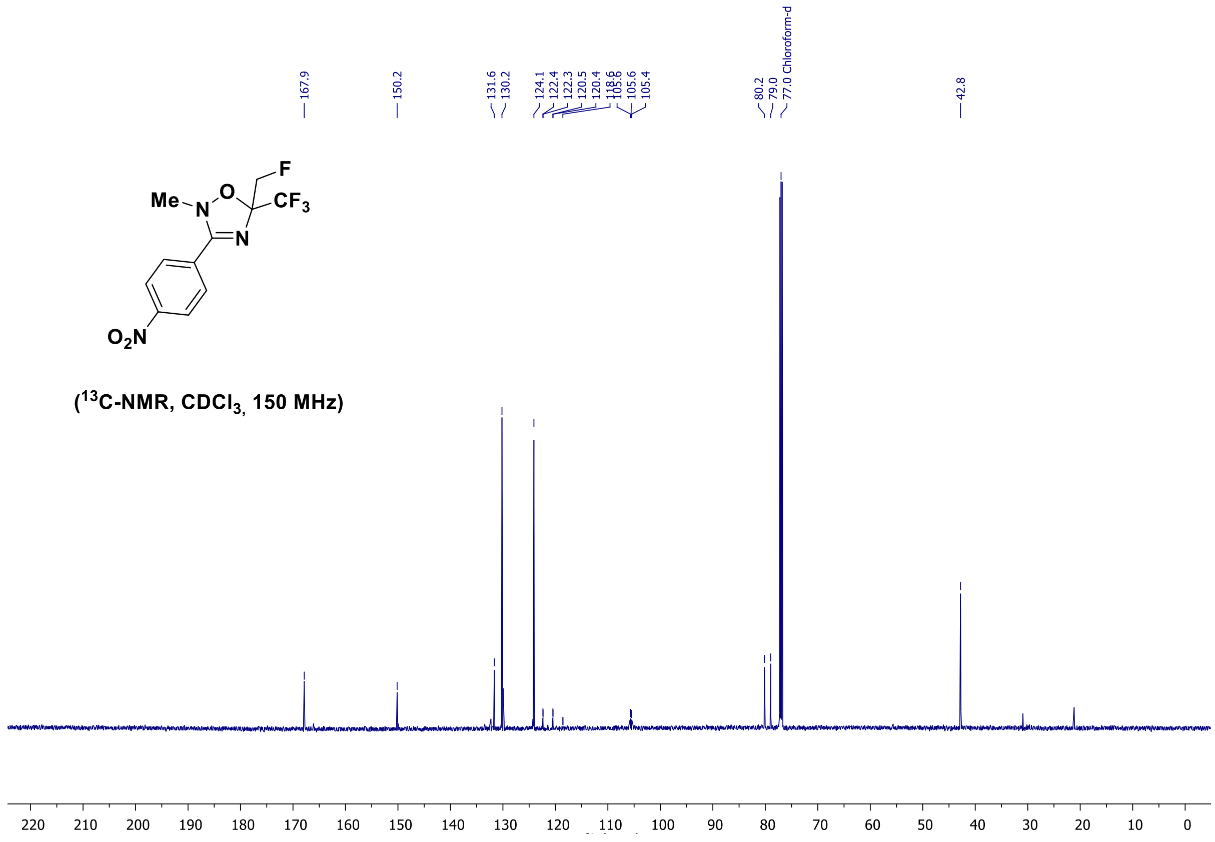

(^{13}C-NMR, $CDCl_3$, 150 MHz)

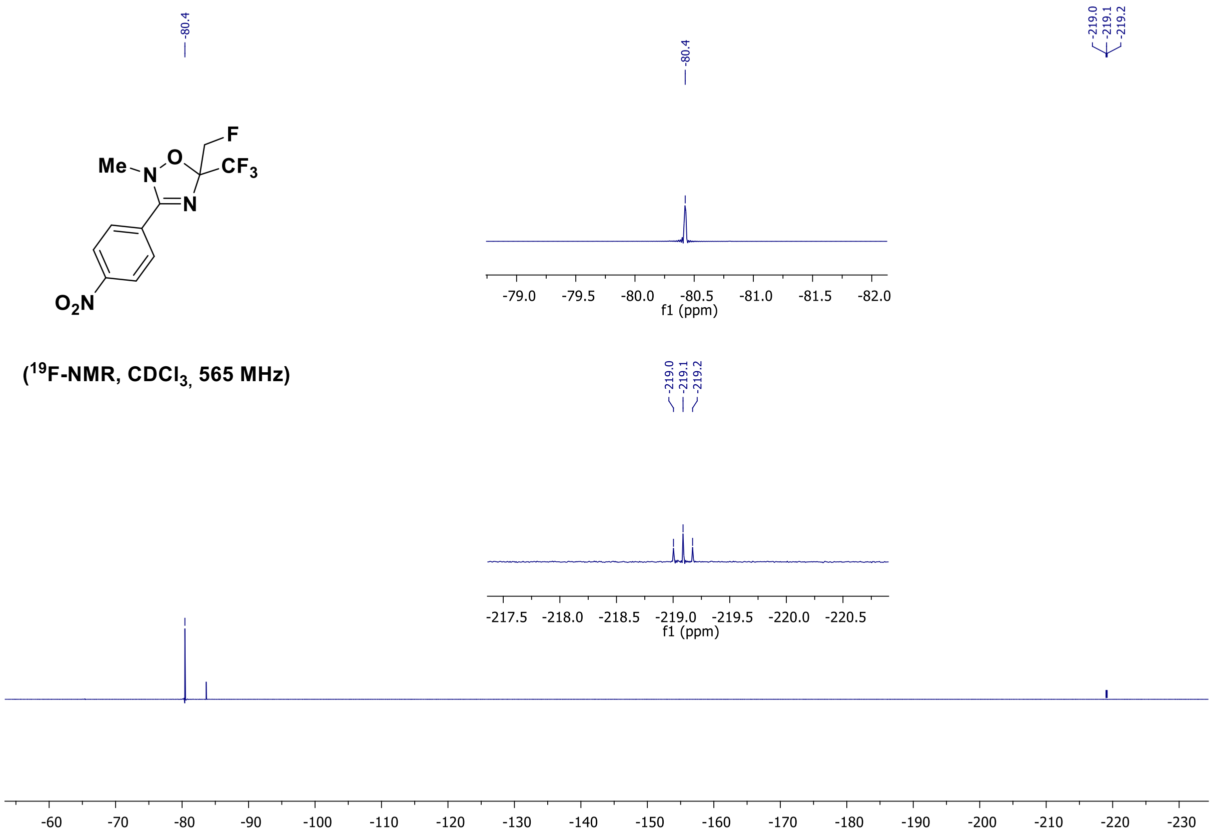

5-(fluoromethyl)-2-methyl-3-(thiophen-2-yl)-5-(trifluoromethyl)-2,5-dihydro-1,2,4-oxadiazole (13)

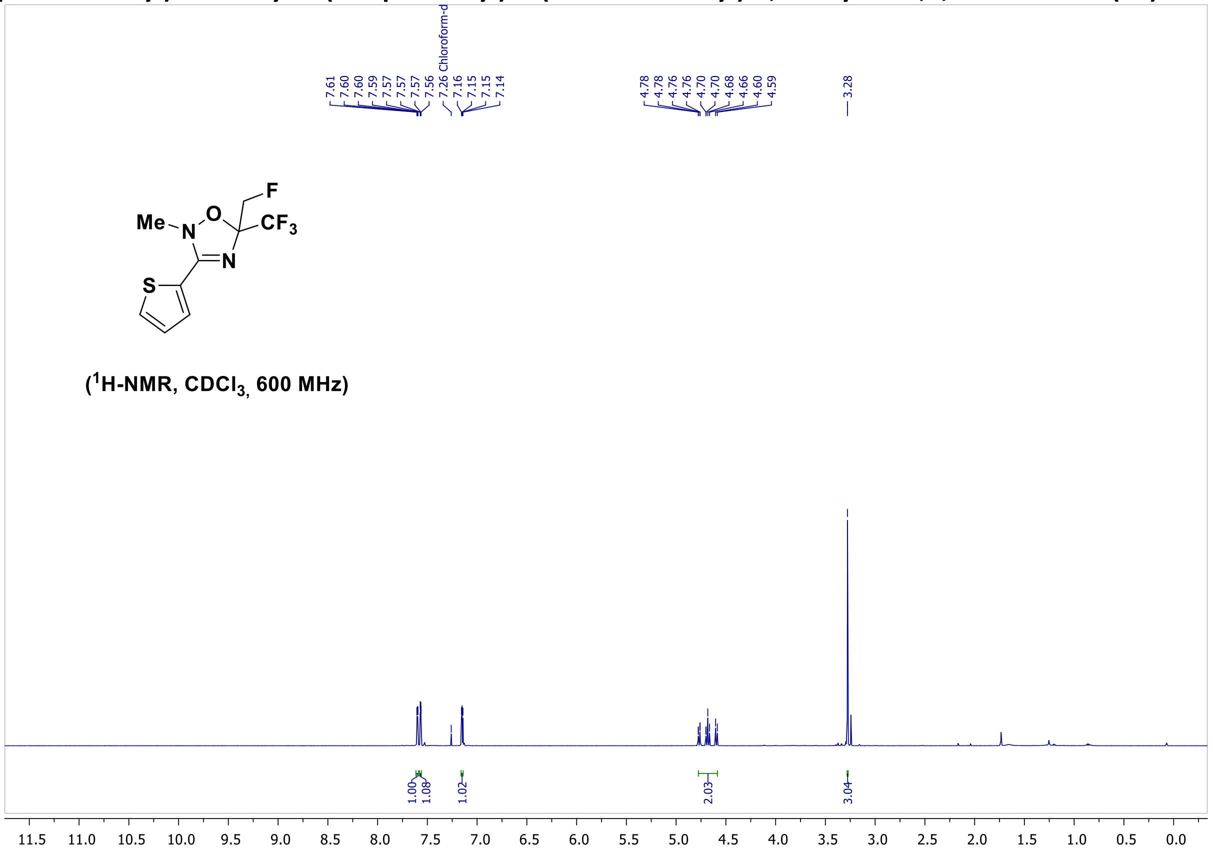

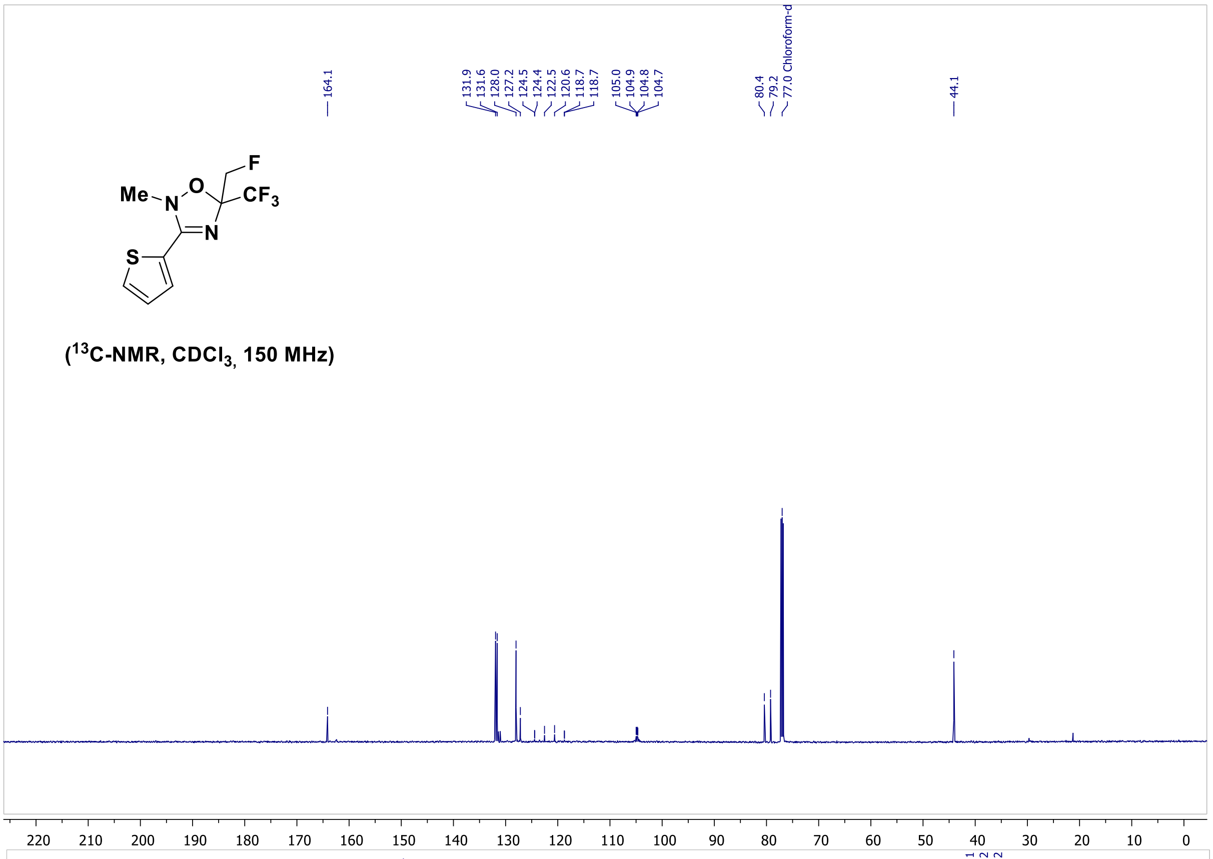

Me
N
O
F
CF3
N
S
(13C-NMR, CDCl3, 150 MHz)
164.1
131.9
131.6
128.0
127.2
124.5
124.4
122.5
120.6
118.7
118.7
105.0
104.9
104.8
104.7
80.4
79.2
77.0 Chloroform-d
44.1
220
210
200
190
180
170
160
150
140
130
120
110
100
90
80
70
60
50
40
30
20
10
0

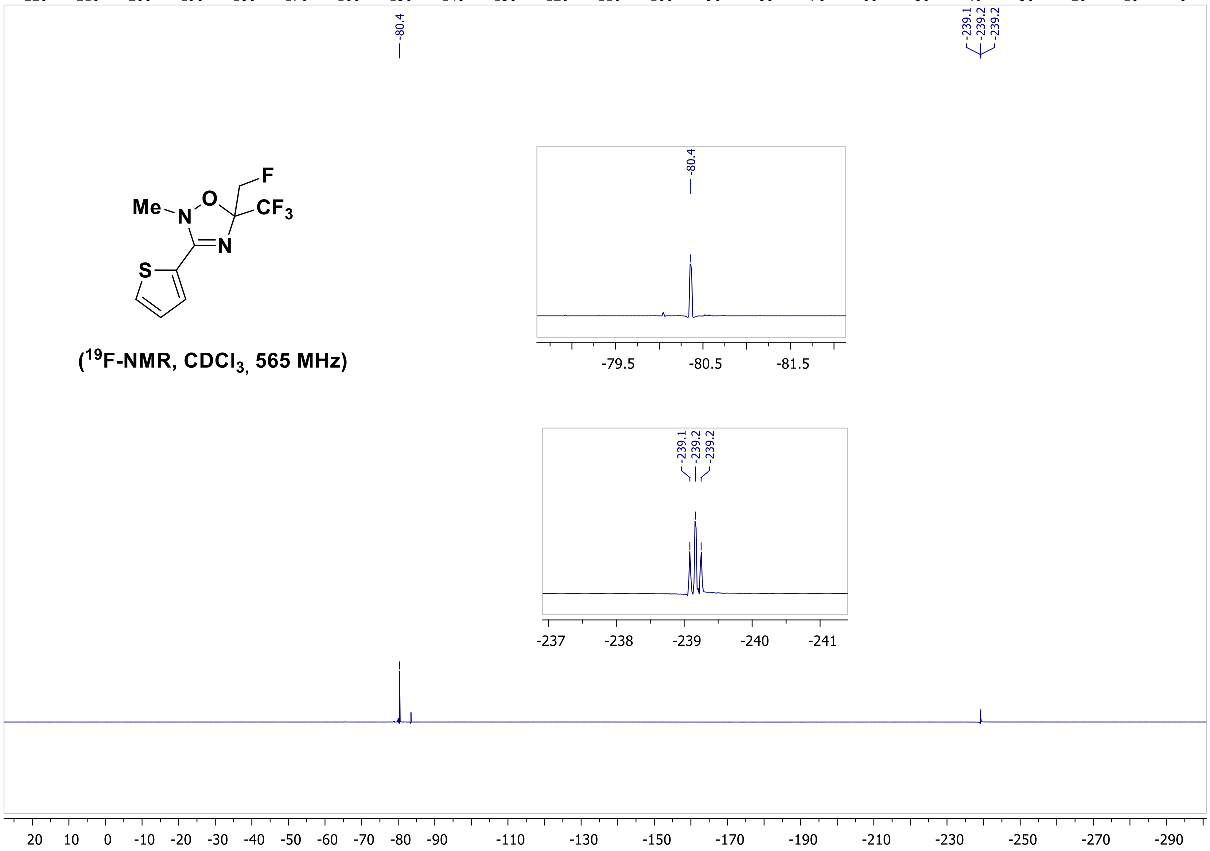

Me
N
O
F
CF3
N
S
(19F-NMR, CDCl3, 565 MHz)
-80.4
-239.1
-239.2
-239.2
-79.5
-80.5
-81.5
-237
-238
-239
-240
-241
20
10
0
-10
-20
-30
-40
-50
-60
-70
-80
-90
-110
-130
-150
-170
-190
-210
-230
-250
-270
-290

3-(4-bromophenyl)-5-(iodomethyl)-4-methyl-5-(trifluoromethyl)-4,5-dihydro-1,2,4-oxadiazole (3)

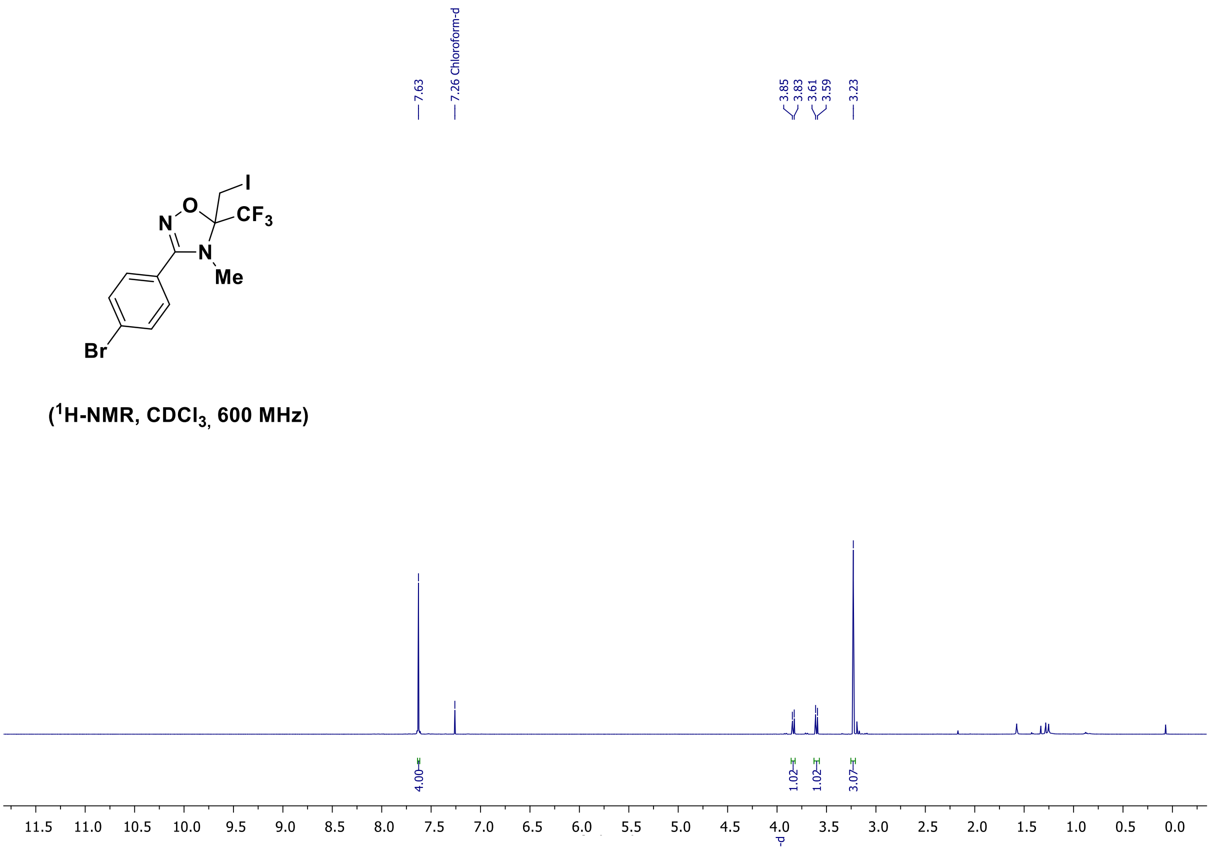

(^{1}H-NMR, $CDCl_3$, 600 MHz)

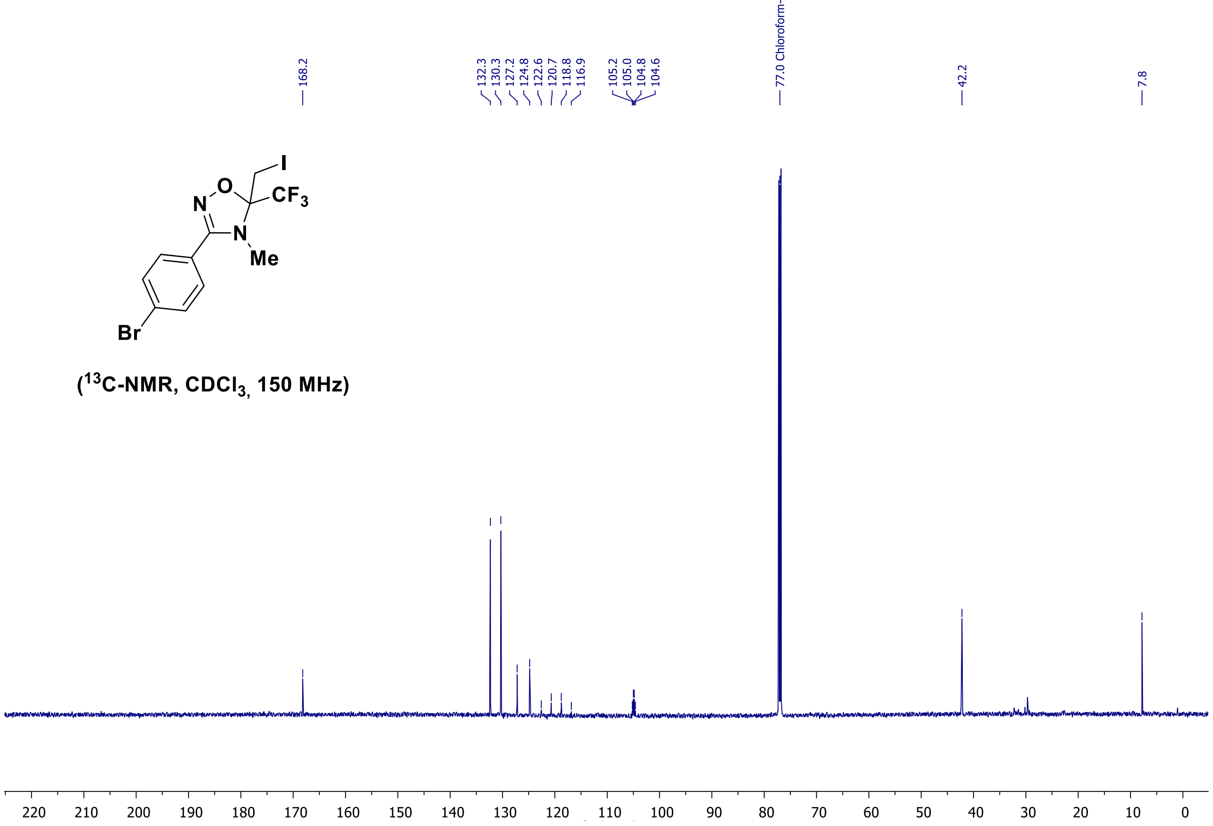

(^{13}C-NMR, $CDCl_3$, 150 MHz)

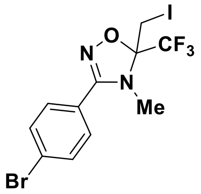

(^{19}F-NMR, $CDCl_3$, 565 MHz)

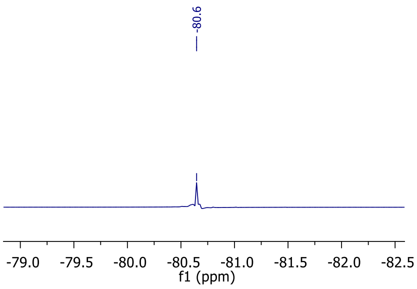

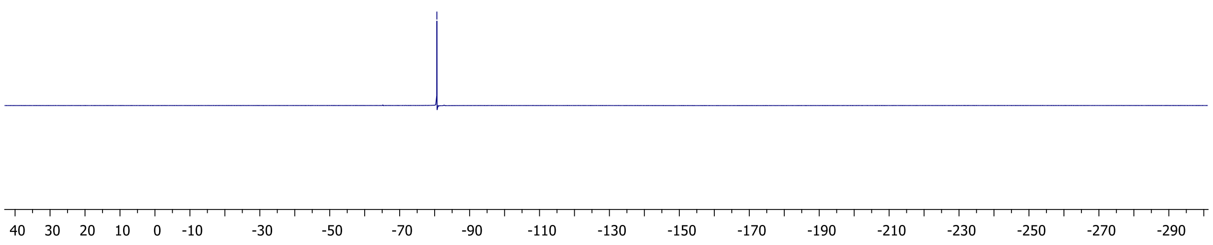

3-(4-fluorophenyl)-5-(iodomethyl)-4-methyl-5-(trifluoromethyl)-4,5-dihydro-1,2,4-oxadiazole (14)

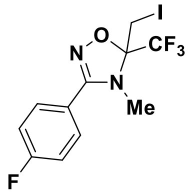

(^{1}H-NMR, $CDCl_3$, 600 MHz)

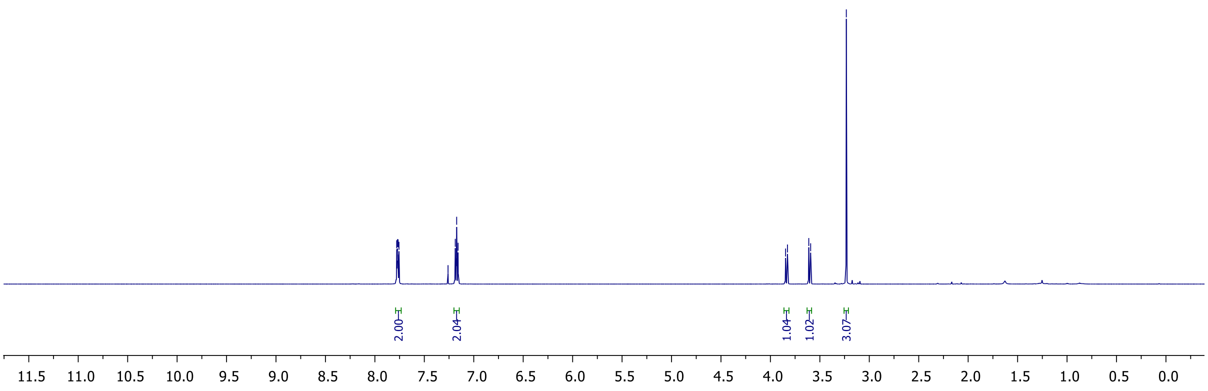

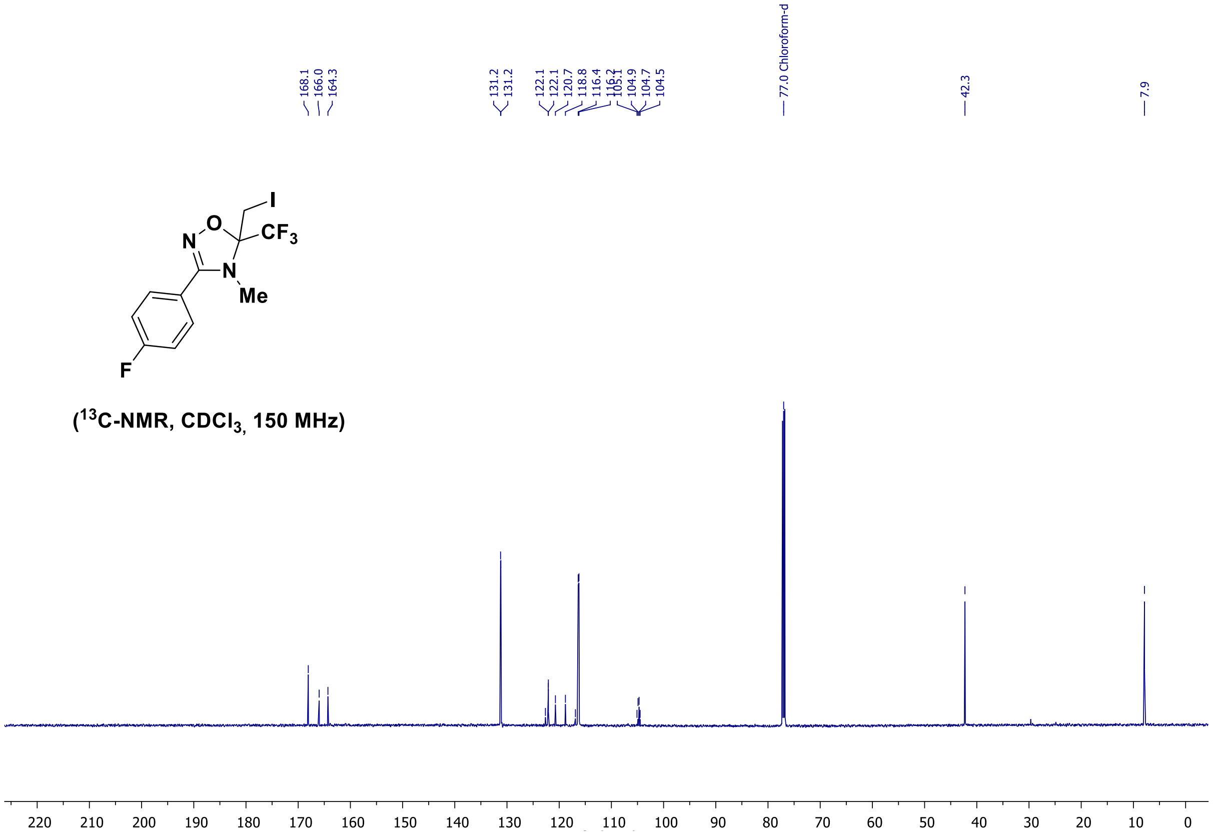

(^{13}C-NMR, $CDCl_3$, 150 MHz)

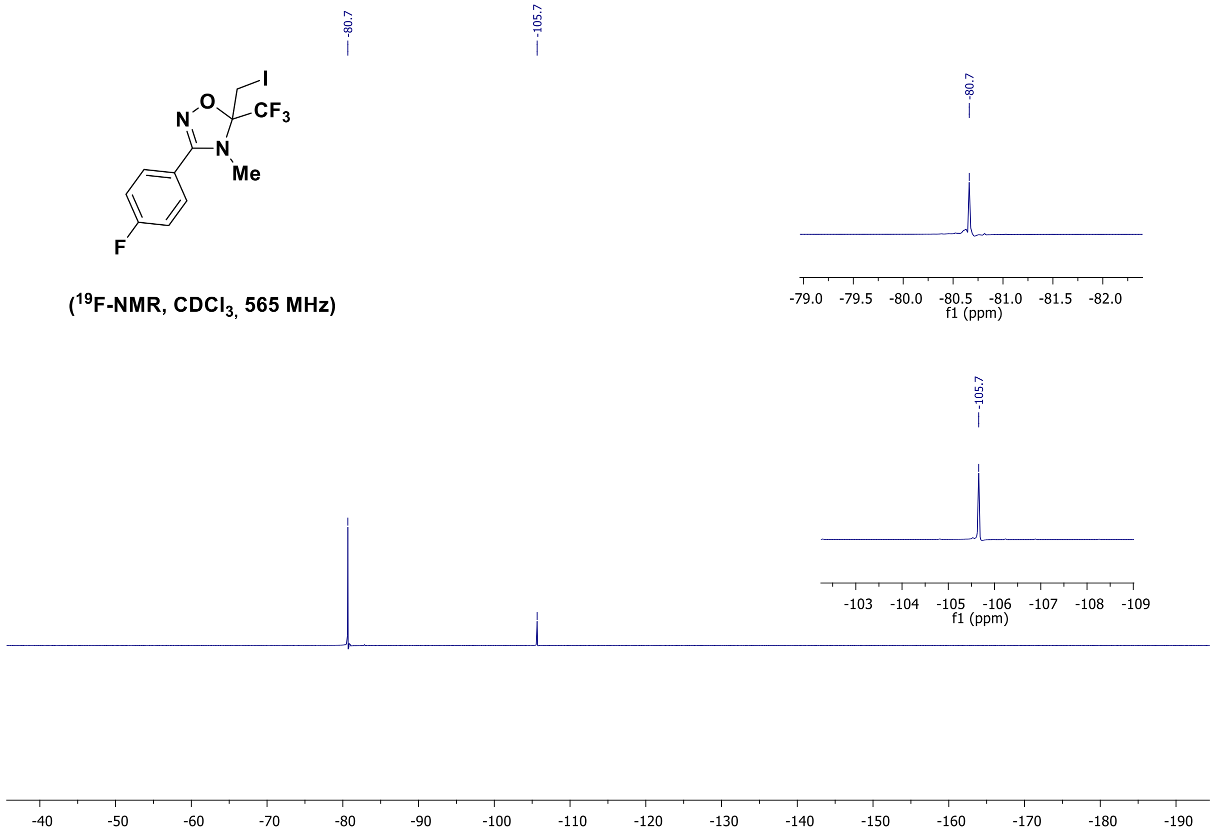

(^{19}F-NMR, $CDCl_3$, 565 MHz)

5-(iodomethyl)-4-methyl-3-phenyl-5-(trifluoromethyl)-4,5-dihydro-1,2,4-oxadiazole (15)

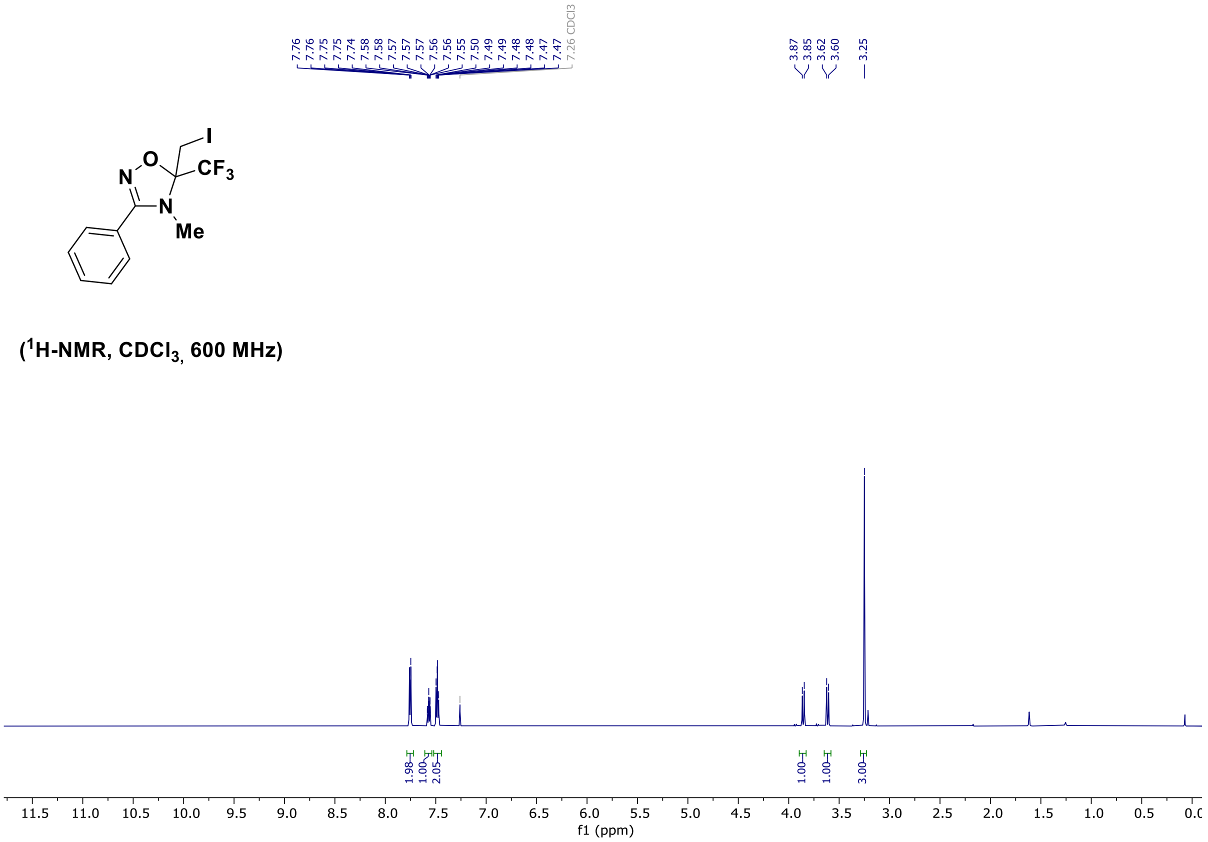

(^{1}H-NMR, $CDCl_3$, 600 MHz)

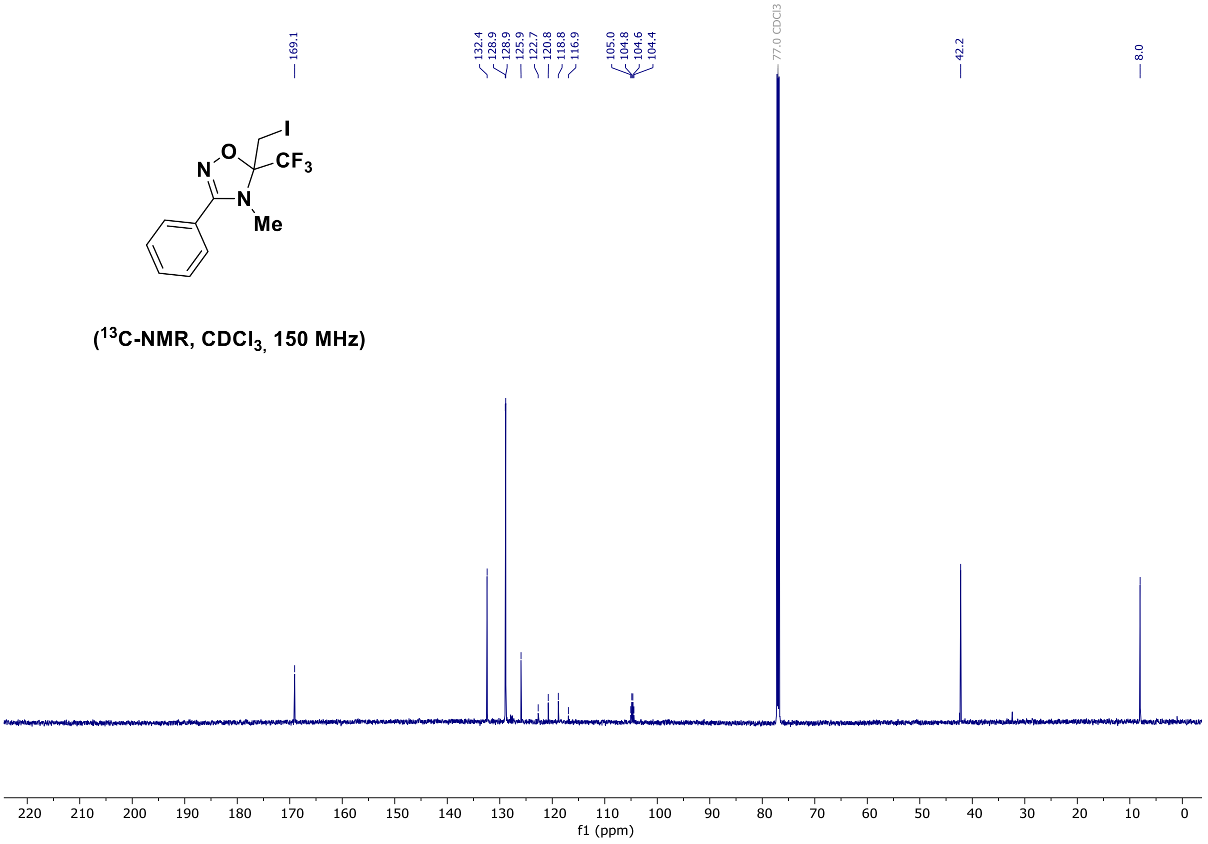

(^{13}C-NMR, $CDCl_3$, 150 MHz)

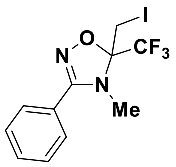

(19F-NMR, $CDCl_3$, 565 MHz)

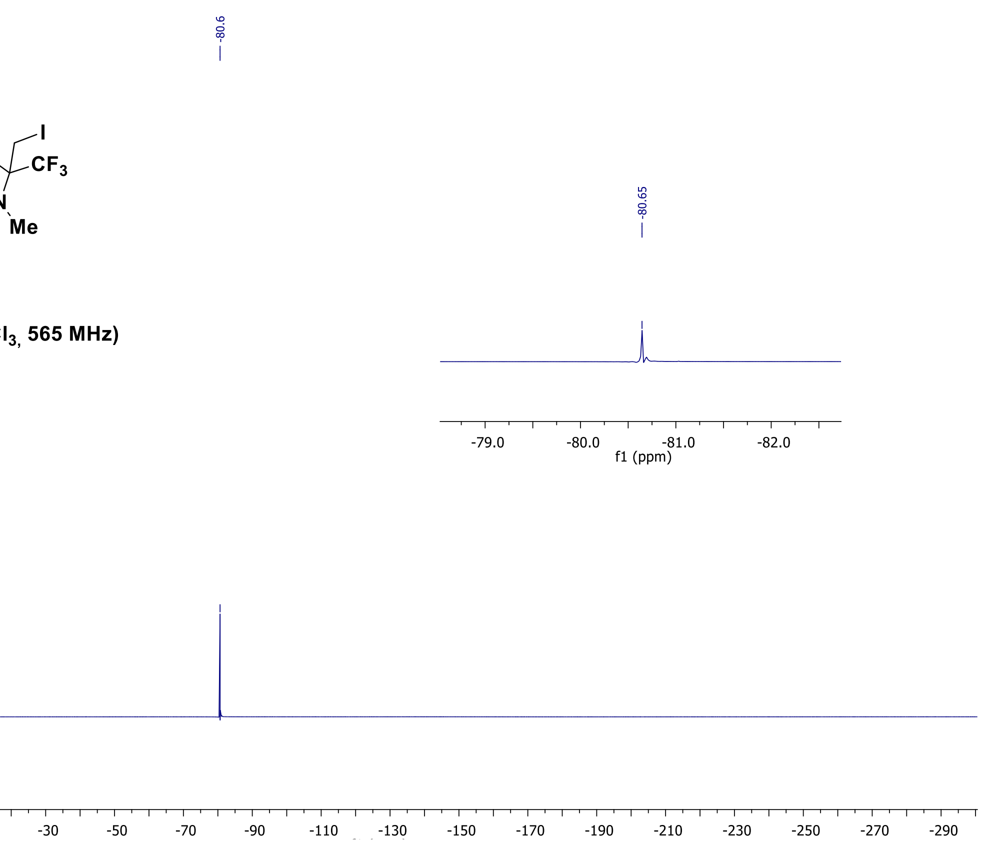

4-(5-(iodomethyl)-4-methyl-5-(trifluoromethyl)-4,5-dihydro-1,2,4-oxadiazol-3-yl)-*N*,*N*-dimethylaniline (16)

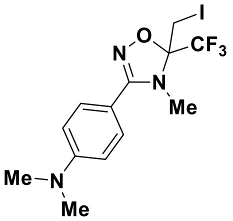

(1H-NMR, $CDCl_3$, 600 MHz)

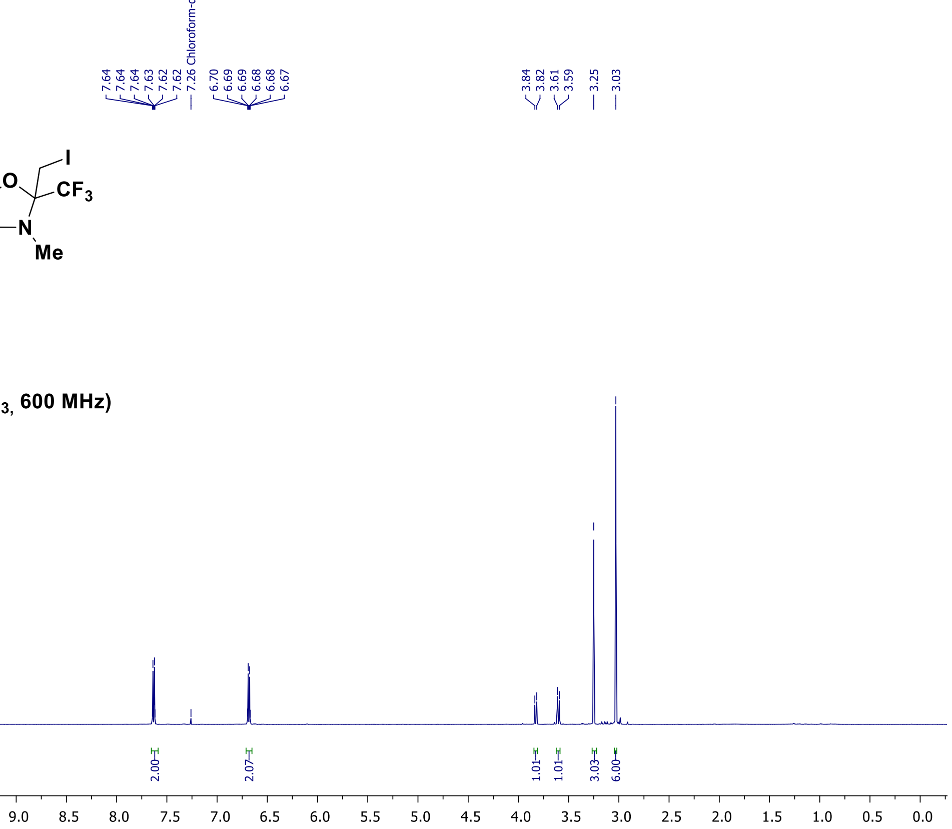

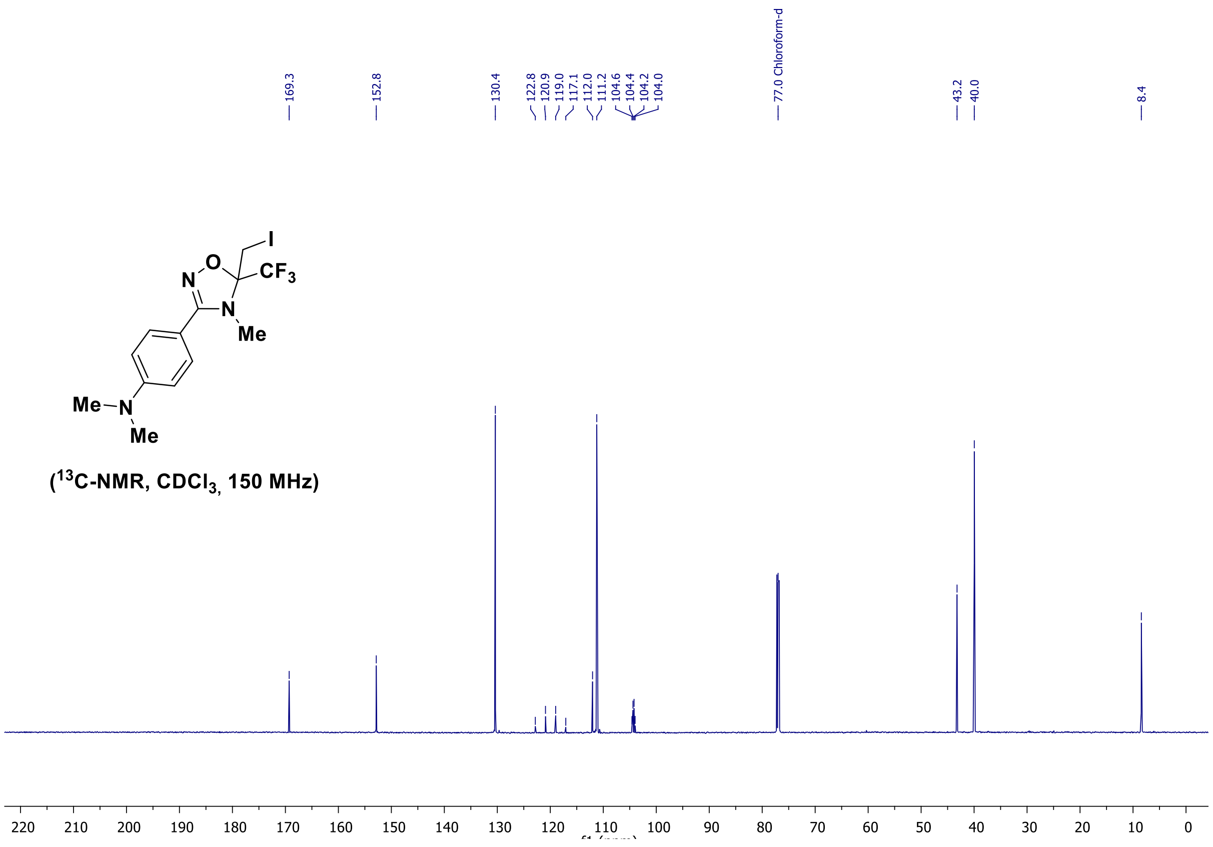
169.3
152.8
130.4
122.8
120.9
119.0
117.1
112.0
111.2
104.6
104.4
104.2
104.0
77.0 Chloroform-d
43.2
40.0
8.4
I
CF3
N
O
N
Me
Me
N
Me
(13C-NMR, CDCl3, 150 MHz)
220 210 200 190 180 170 160 150 140 130 120 110 100 90 80 70 60 50 40 30 20 10 0

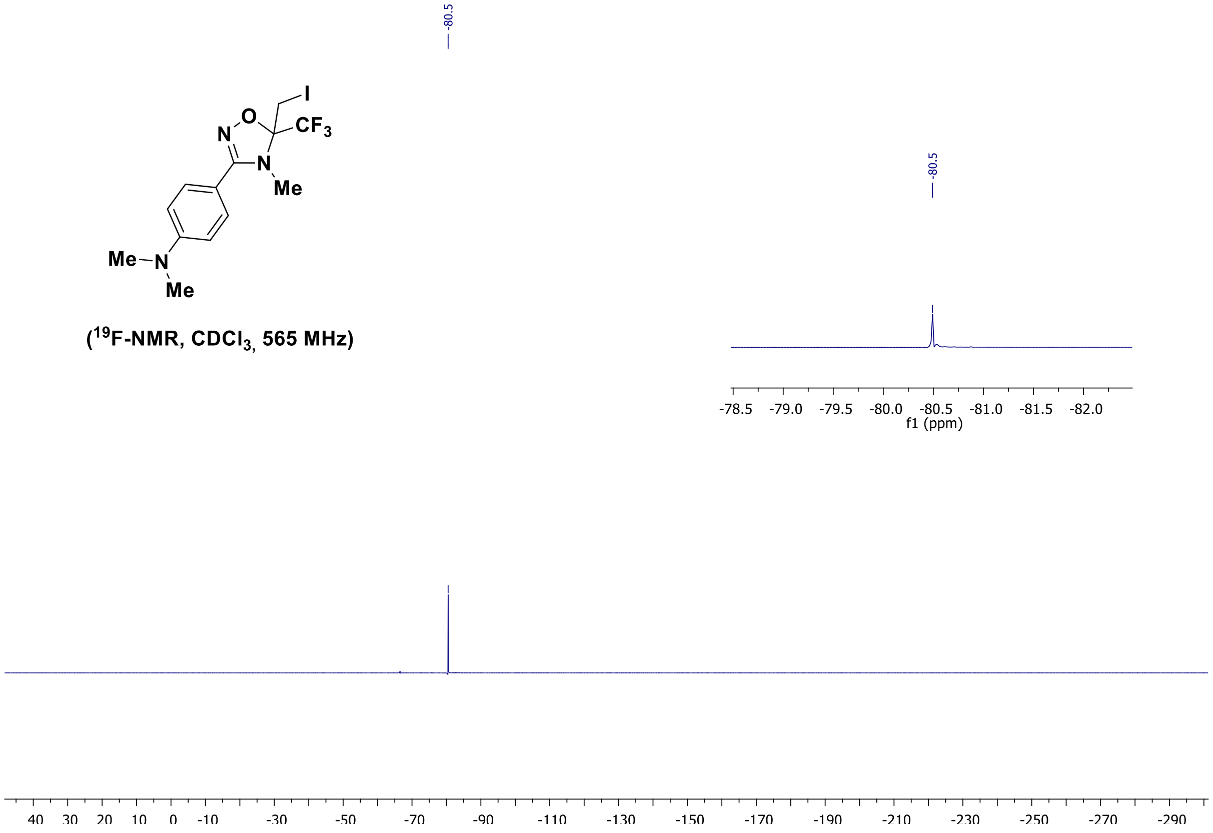
-80.5
I
CF3
N
O
N
Me
Me
N
Me
(19F-NMR, CDCl3, 565 MHz)
-80.5
-78.5 -79.0 -79.5 -80.0 -80.5 -81.0 -81.5 -82.0
f1 (ppm)
40 30 20 10 0 -10 -30 -50 -70 -90 -110 -130 -150 -170 -190 -210 -230 -250 -270 -290

5-(iodomethyl)-3-(4-methoxyphenyl)-4-methyl-5-(trifluoromethyl)-4,5-dihydro-1,2,4-oxadiazole (17)

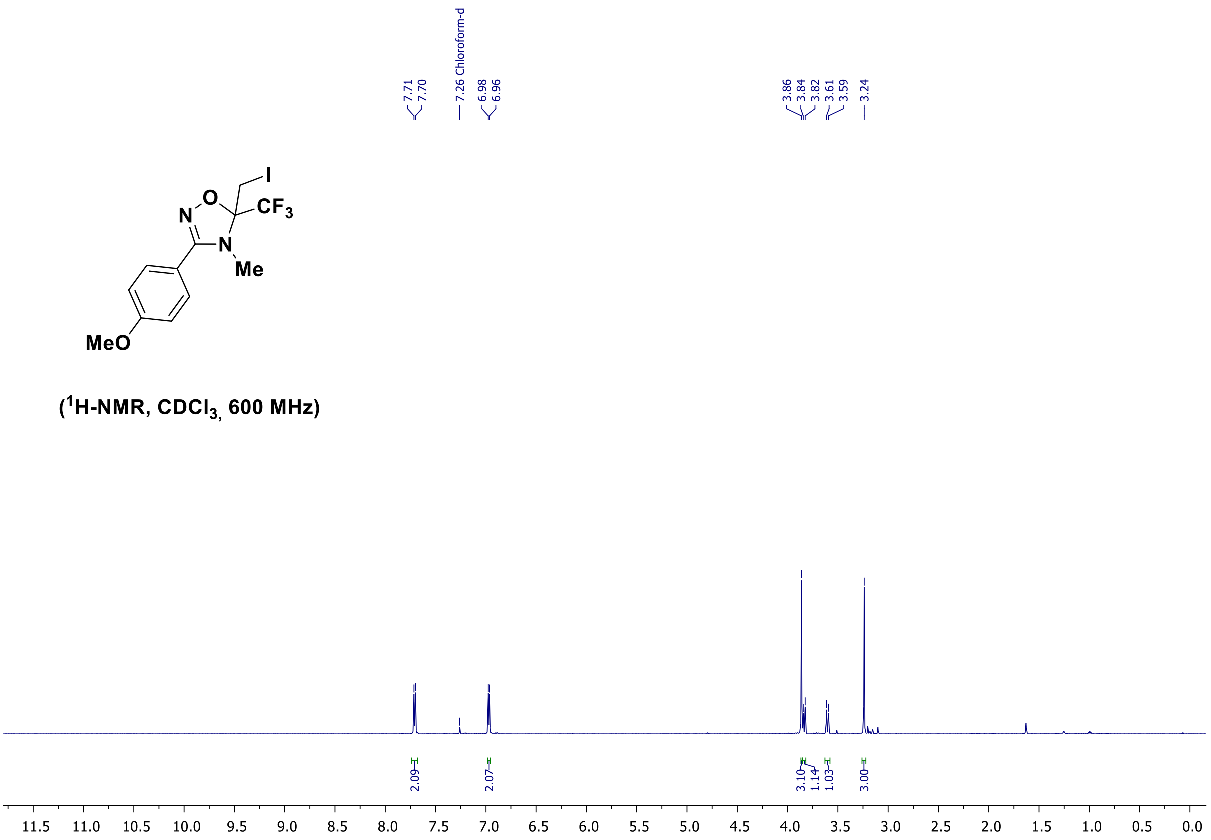

(^{1}H-NMR, $CDCl_3$, 600 MHz)

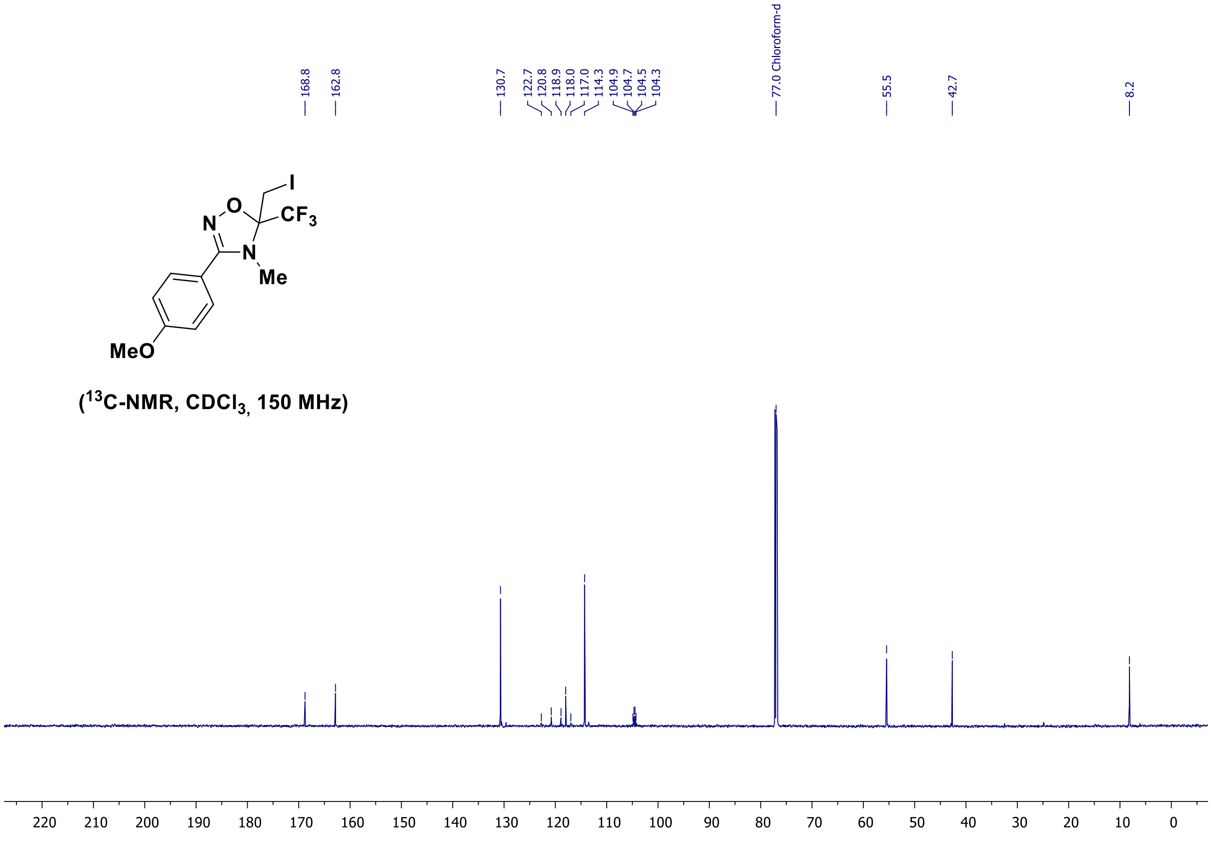

(^{13}C-NMR, $CDCl_3$, 150 MHz)

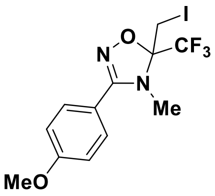

(19F-NMR, $CDCl_3$, 565 MHz)

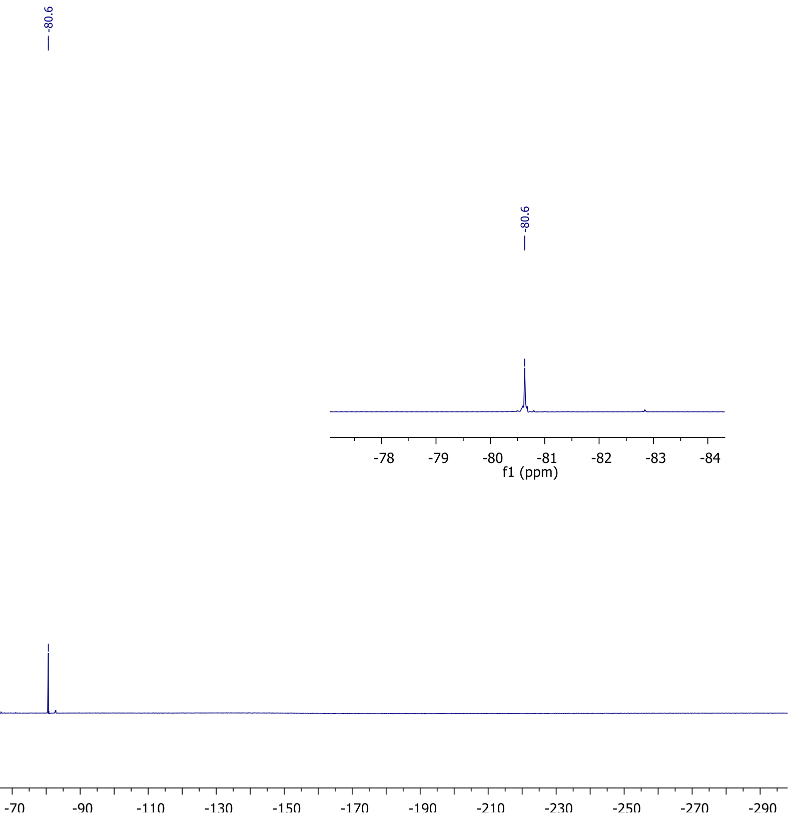

5-(bromomethyl)-2-methyl-3-(3-methylphenyl)-5-(trifluoromethyl)-2,5-dihydro-1,2,4-oxadiazole (18) and 5-(bromomethyl)-4-methyl-3-(3-methylphenyl)-5-(trifluoromethyl)-4,5-dihydro-1,2,4-oxadiazole (18a)

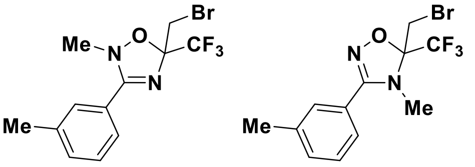

(1H-NMR, $CDCl_3$, 600 MHz)

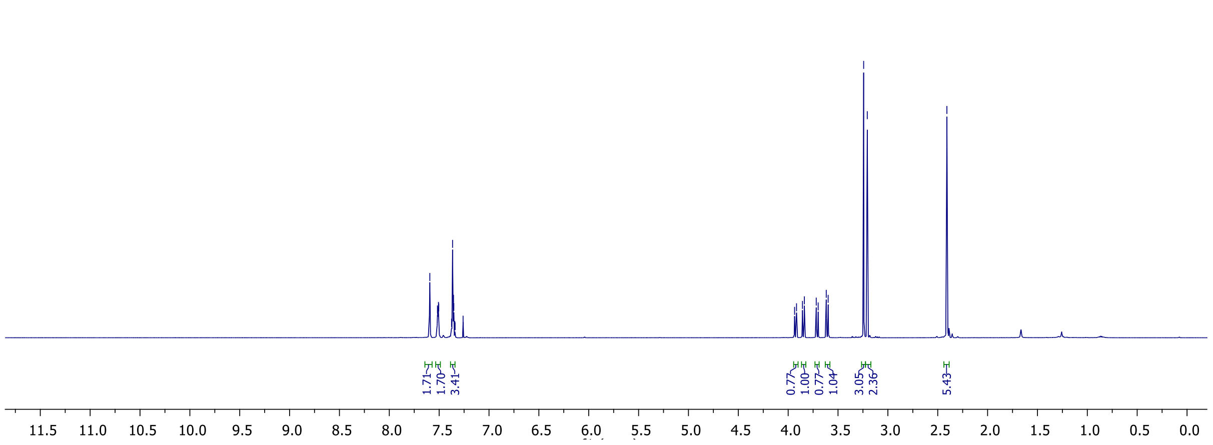

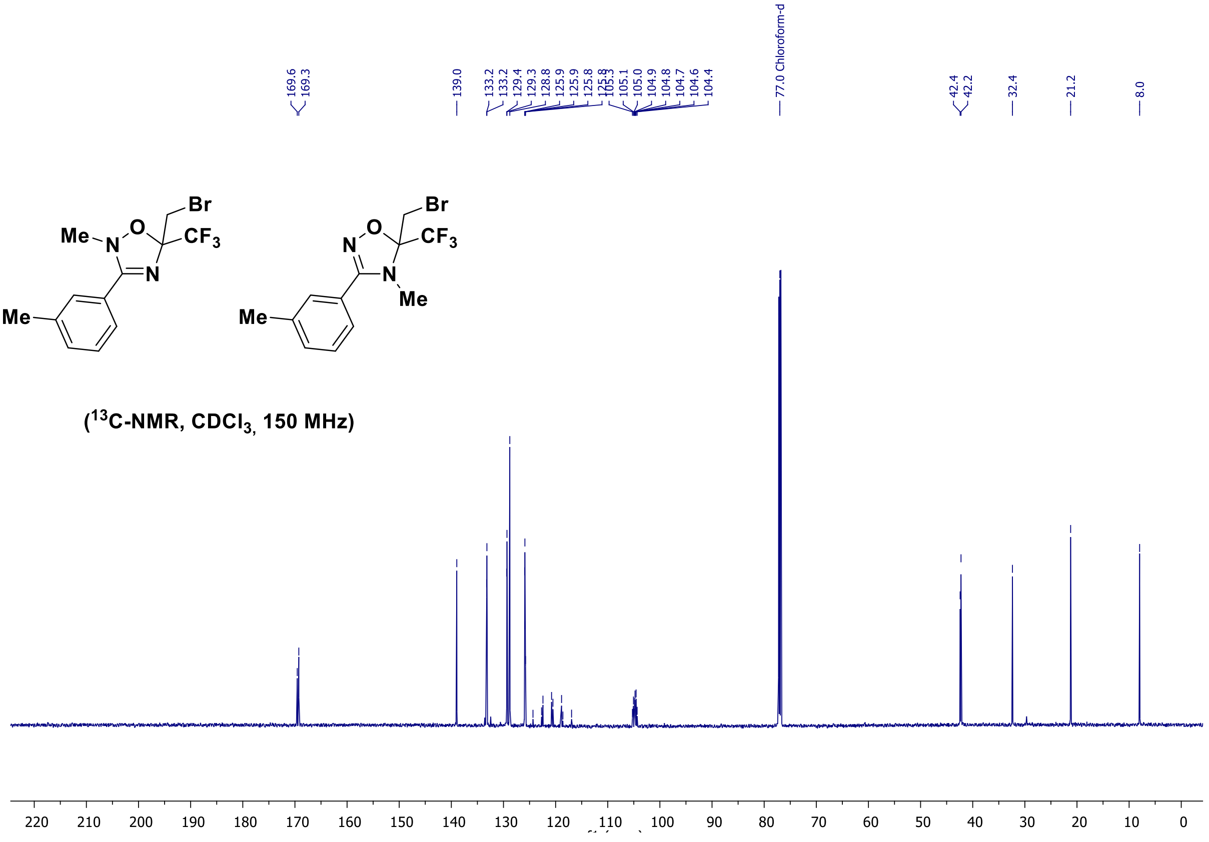

(^{13}C-NMR, $CDCl_3$, 150 MHz)

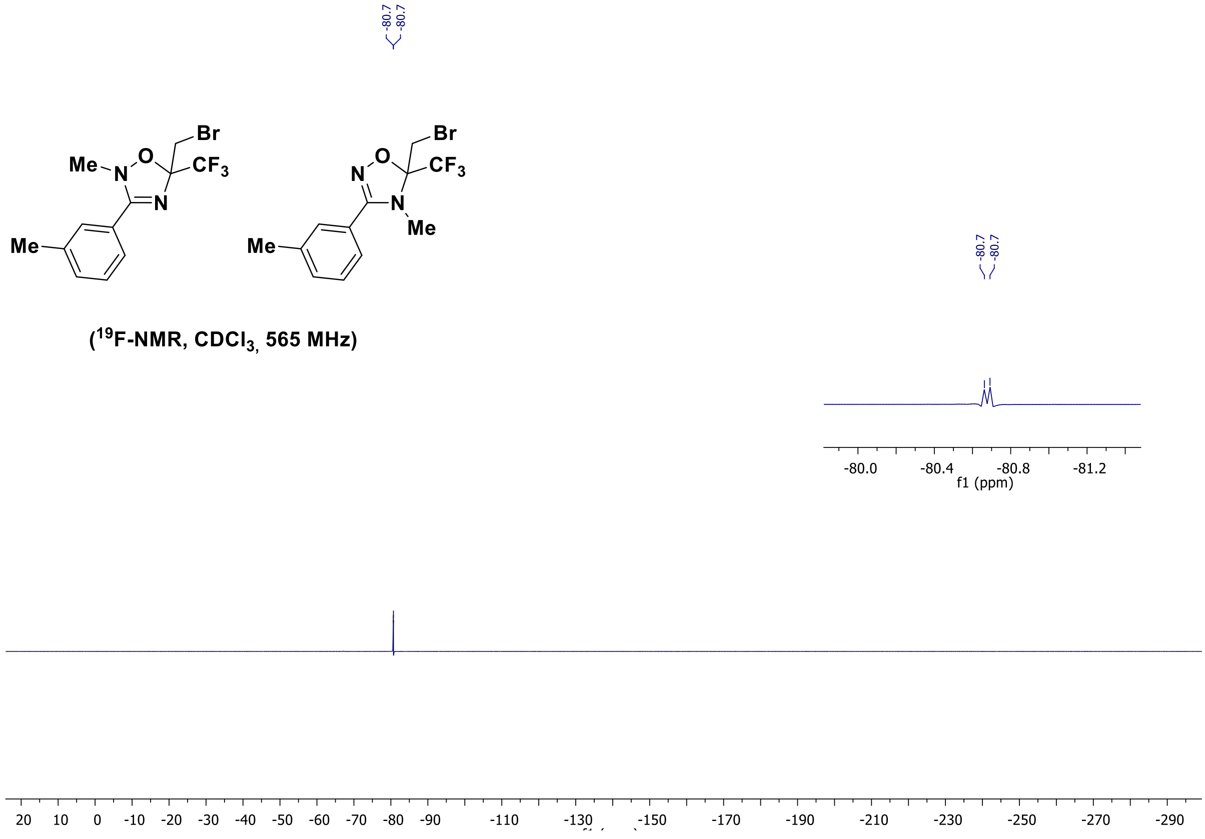

(^{19}F-NMR, $CDCl_3$, 565 MHz)

5-(bromomethyl)-3-(4-chlorophenyl)-2-methyl-5-(trifluoromethyl)-2,5-dihydro-1,2,4-oxadiazole (19) and 5-(bromomethyl)-3-(4-chlorophenyl)-4-methyl-5-(trifluoromethyl)-4,5-dihydro-1,2,4-oxadiazole (19a)

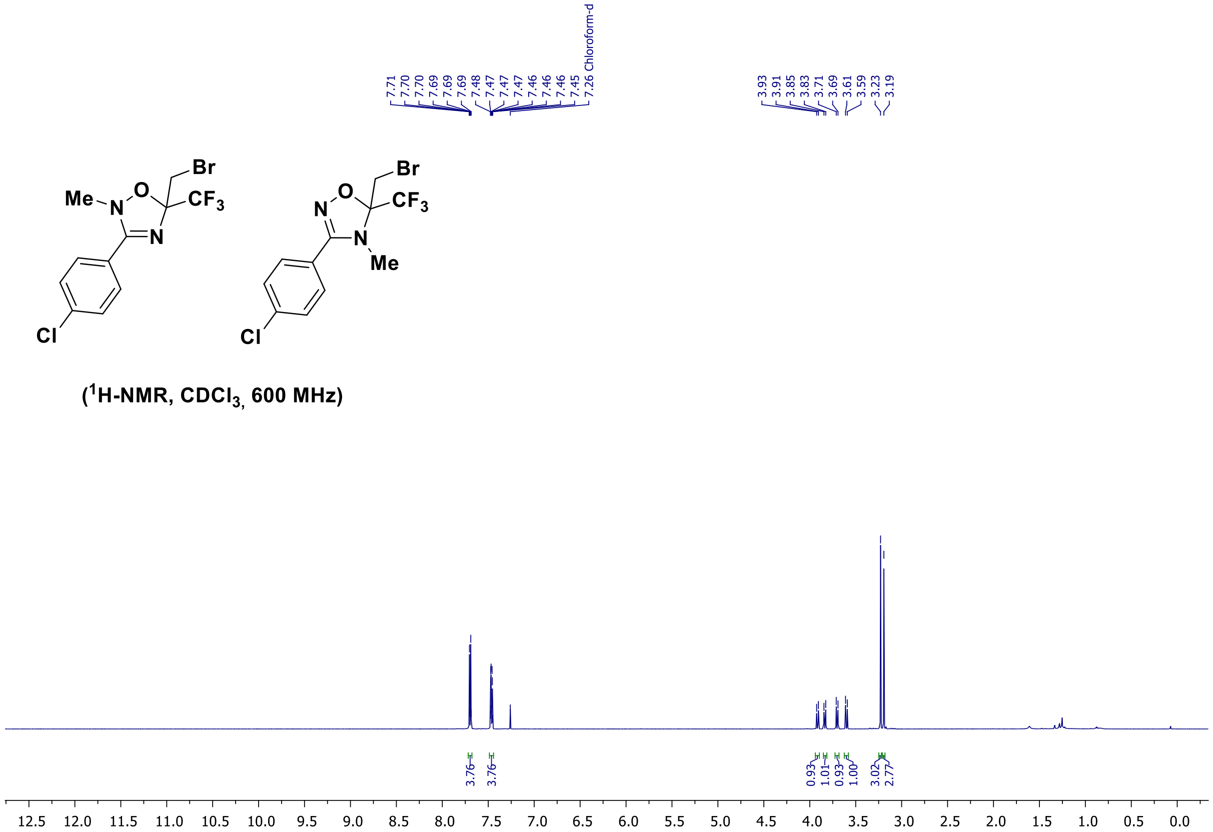

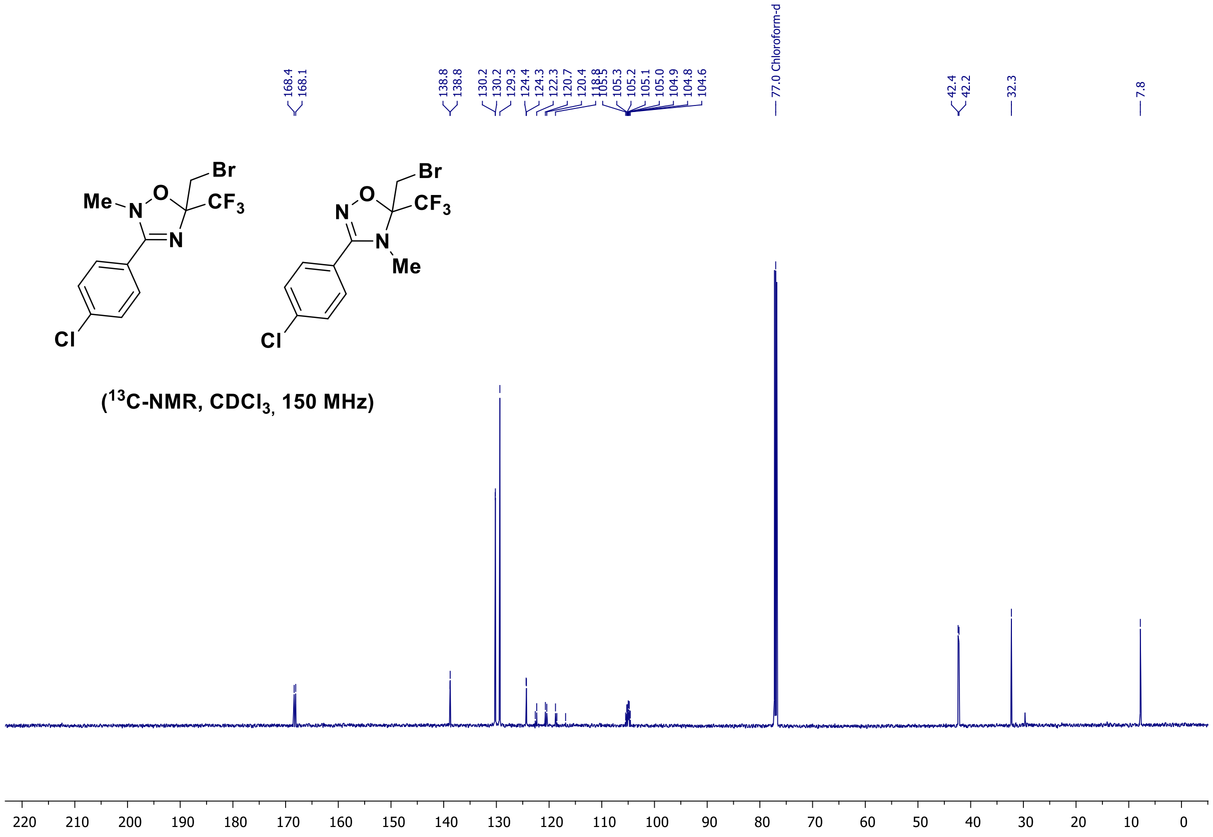

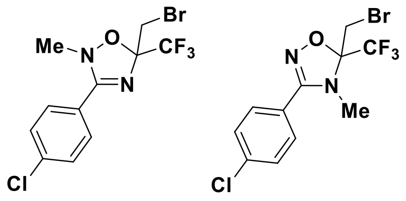

(^{19}F-NMR, $CDCl_3$, 565 MHz)

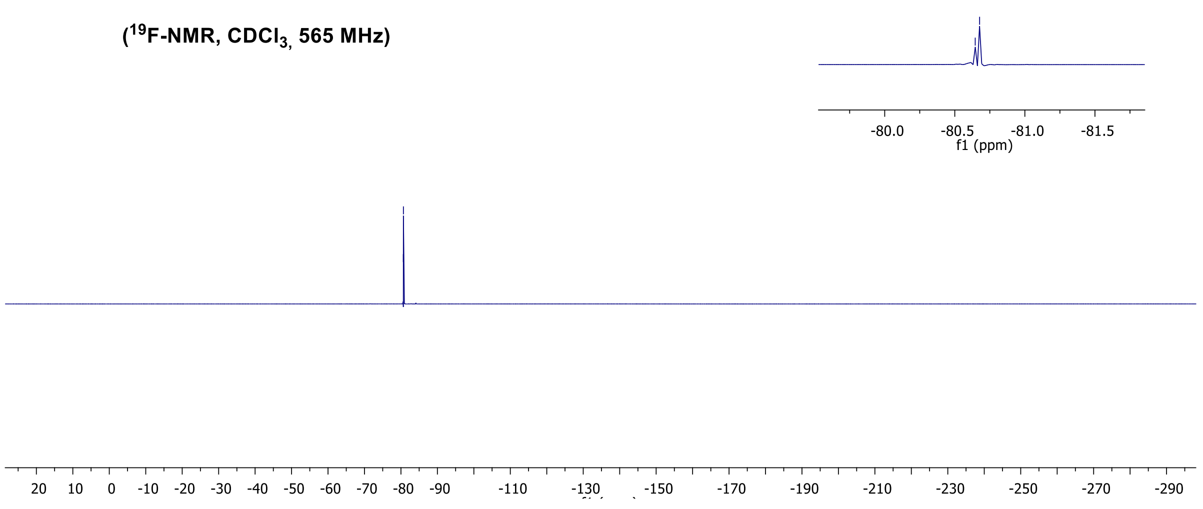

4-(5-(bromomethyl)-2-methyl-5-(trifluoromethyl)-2,5-dihydro-1,2,4-oxadiazol-3-yl)benzonitrile (20) and 4-(5-(bromomethyl)-4-methyl-5-(trifluoromethyl)-4,5-dihydro-1,2,4-oxadiazol-3-yl)benzonitrile (20a)

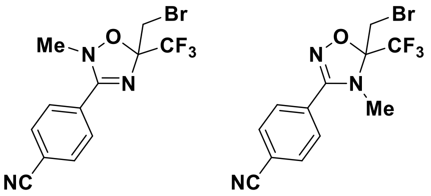

(^{1}H-NMR, $CDCl_3$, 600 MHz)

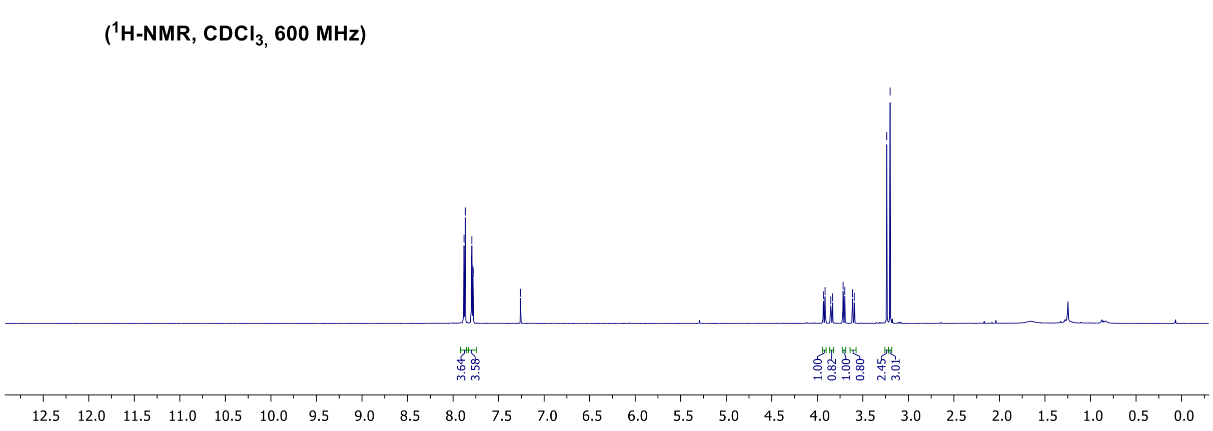

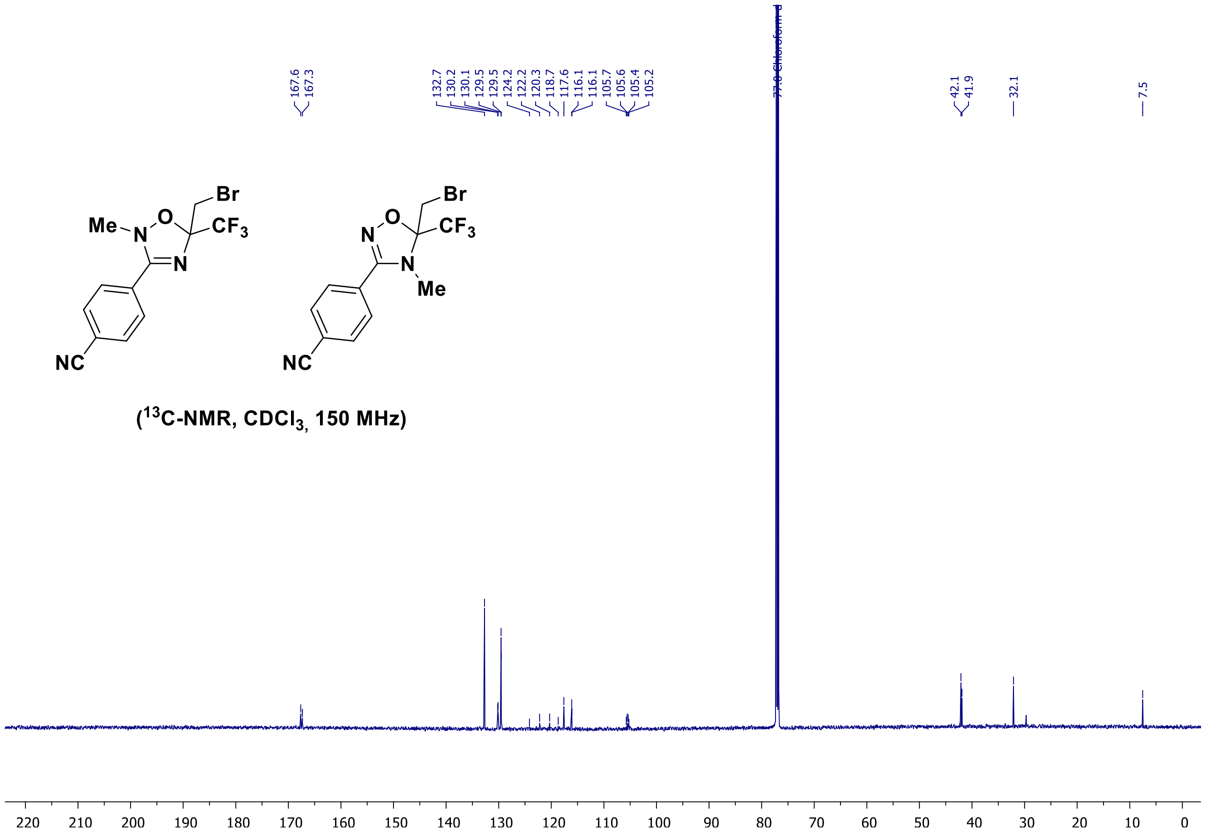
167.6
167.3
132.7
130.2
130.1
129.5
129.5
124.2
122.2
120.3
118.7
117.6
116.1
116.1
105.7
105.6
105.4
105.2
77.0 Chloroform-d
42.1
41.9
32.1
7.5
Br
Me
N
O
CF3
N
NC
Me
(13C-NMR, CDCl3, 150 MHz)
220 210 200 190 180 170 160 150 140 130 120 110 100 90 80 70 60 50 40 30 20 10 0

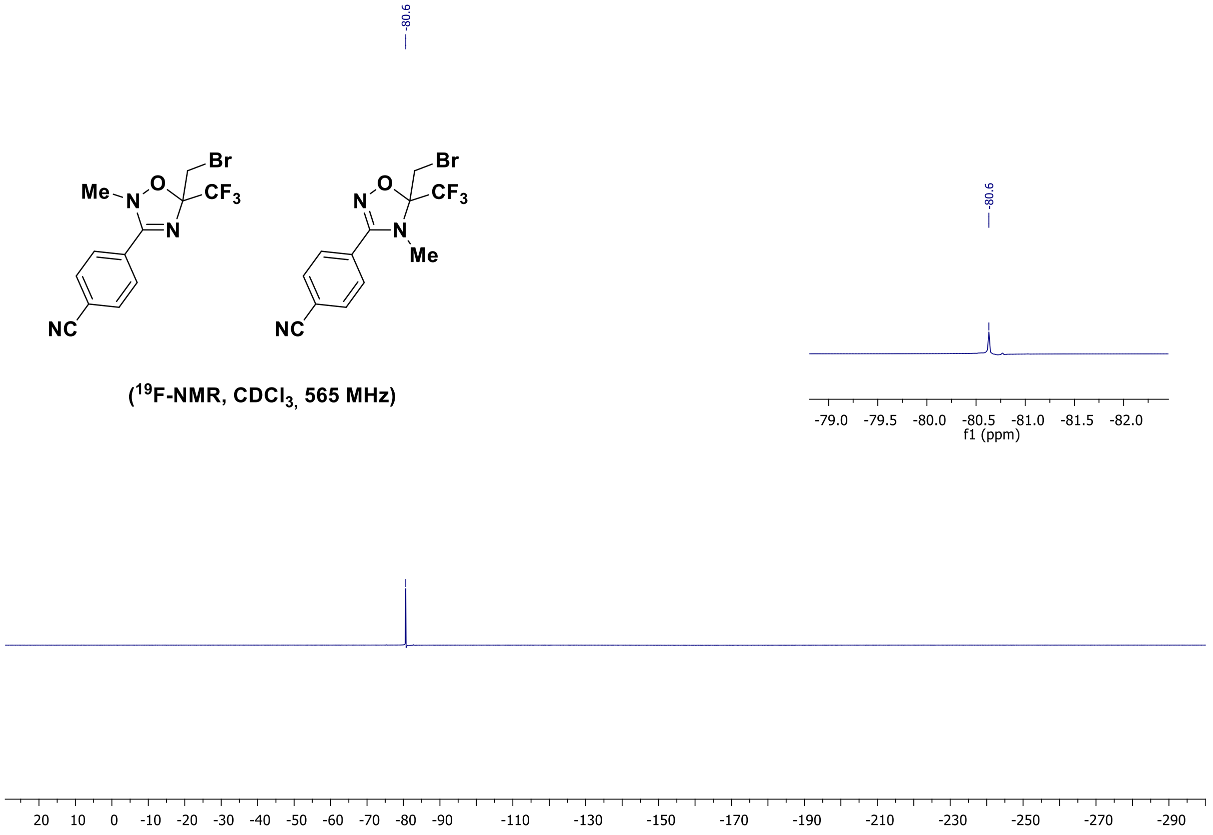
-80.6
Br
Me
N
O
CF3
N
NC
Me
(19F-NMR, CDCl3, 565 MHz)
-80.6
-79.0 -79.5 -80.0 -80.5 -81.0 -81.5 -82.0
f1 (ppm)
20 10 0 -10 -20 -30 -40 -50 -60 -70 -80 -90 -110 -130 -150 -170 -190 -210 -230 -250 -270 -290

5-(bromomethyl)-2-methyl-3-(4-nitrophenyl)-5-(trifluoromethyl)-2,5-dihydro-1,2,4-oxadiazole (20) and 5-(bromomethyl)-4-methyl-3-(4-nitrophenyl)-5-(trifluoromethyl)-4,5-dihydro-1,2,4-oxadiazole (21a)

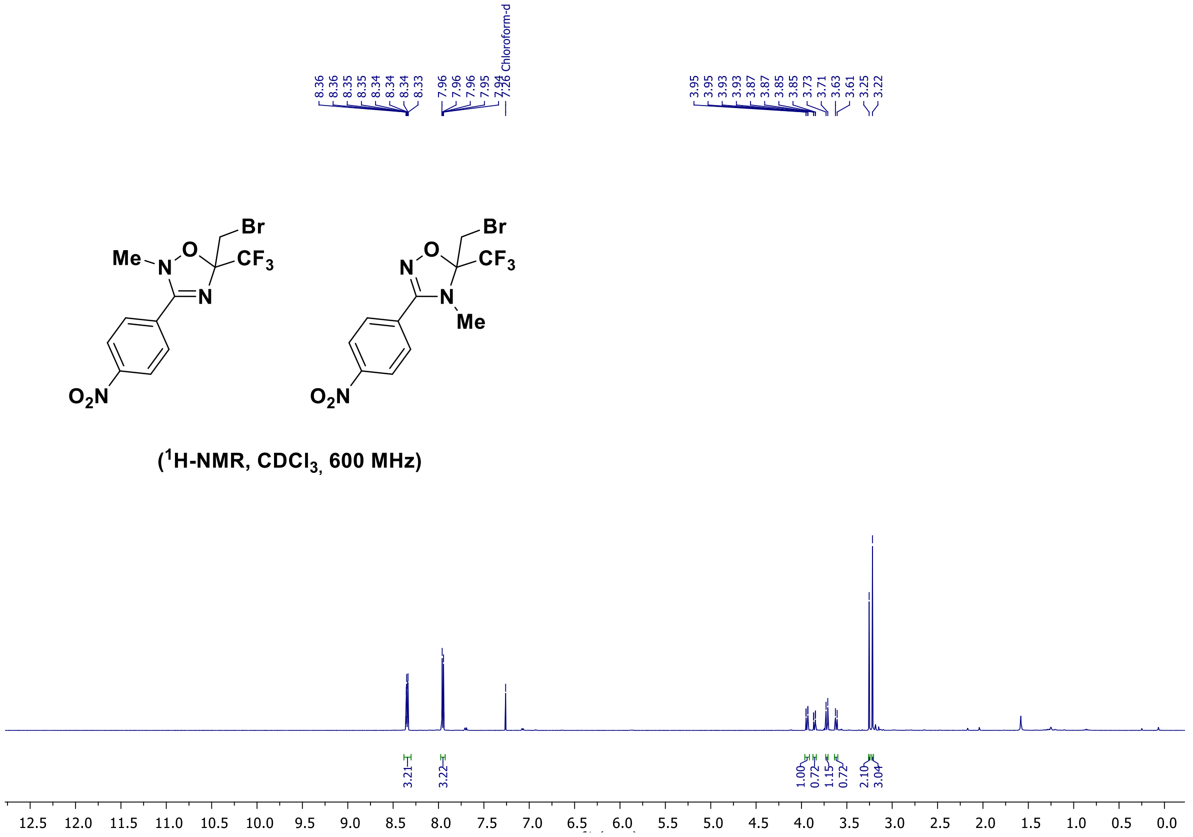

(^{1}H-NMR, $CDCl_3$, 600 MHz)

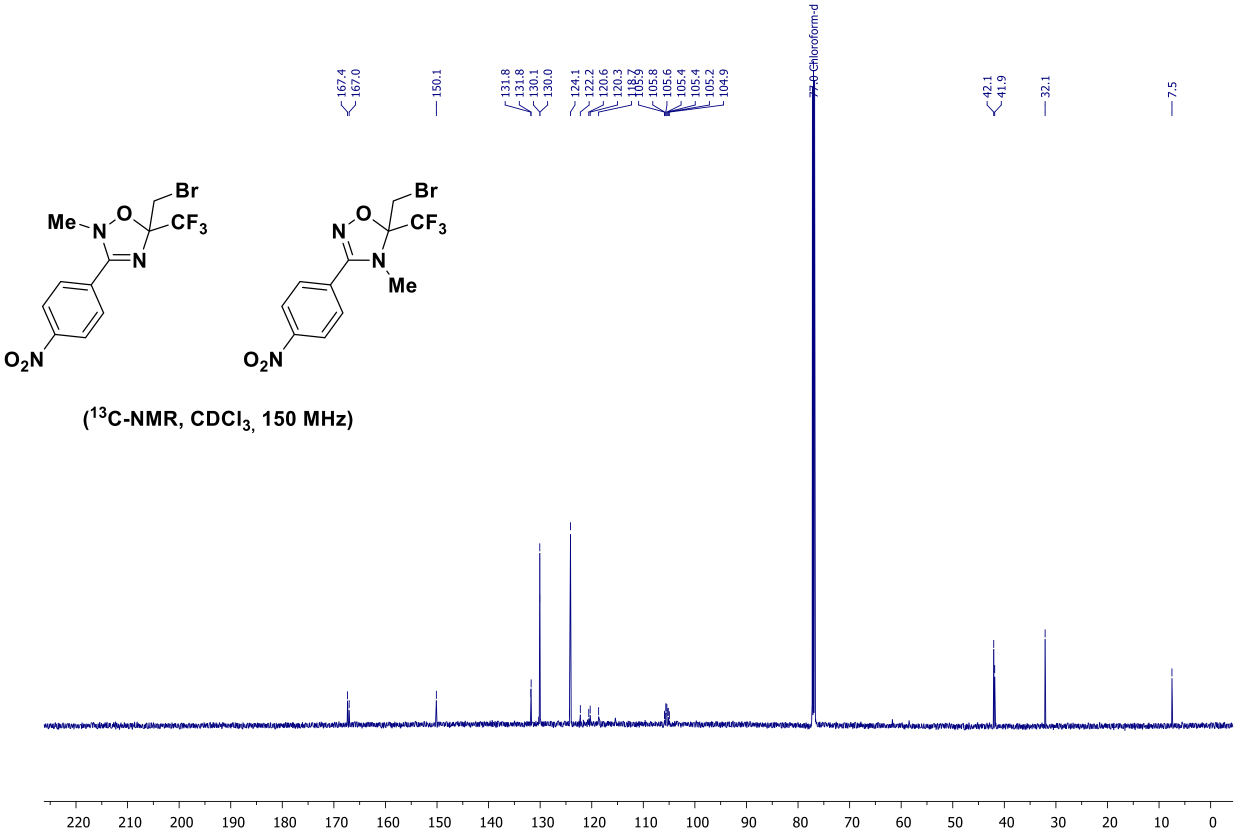

(^{13}C-NMR, $CDCl_3$, 150 MHz)

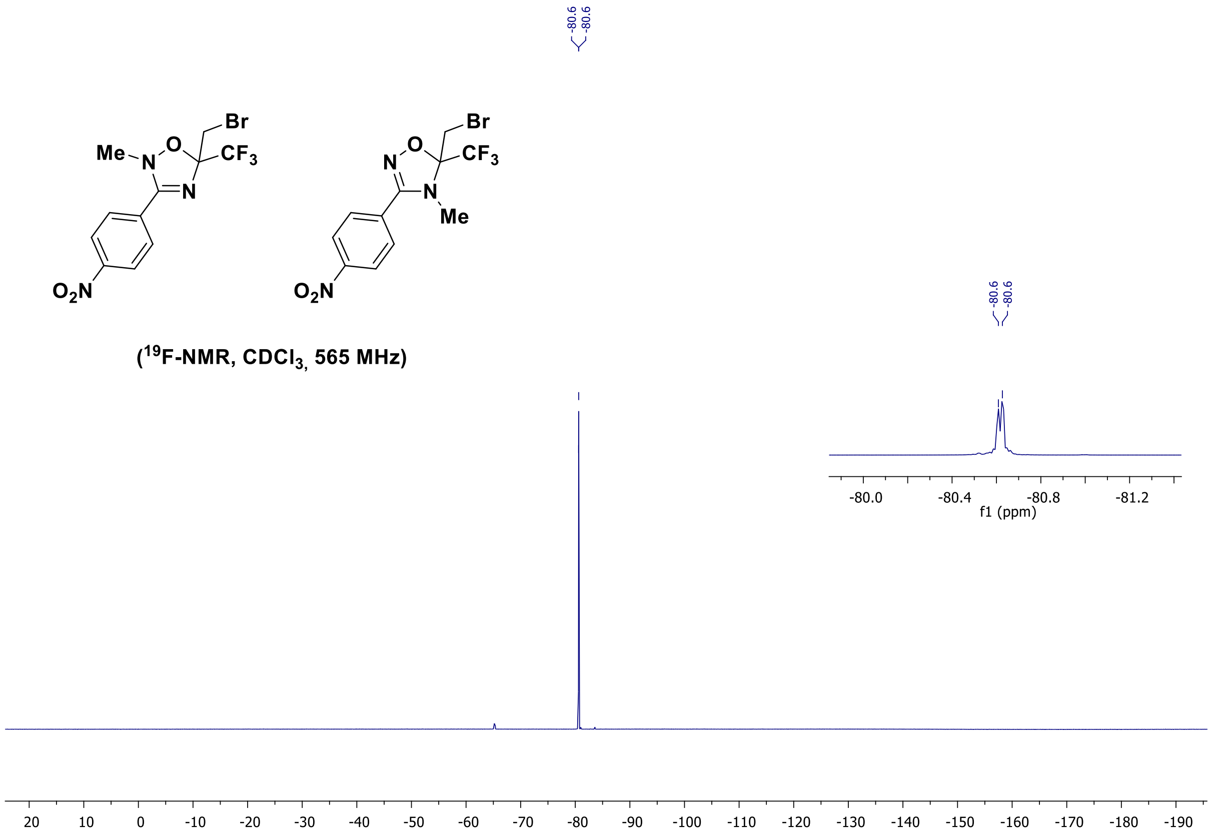

(^{19}F-NMR, $CDCl_3$, 565 MHz)

5-(bromomethyl)-2-methyl-3-(pyridin-2-yl)-5-(trifluoromethyl)-2,5-dihydro-1,2,4-oxadiazole (22) and 5-(bromomethyl)-4-methyl-3-(pyridin-2-yl)-5-(trifluoromethyl)-4,5-dihydro-1,2,4-oxadiazole (22a)

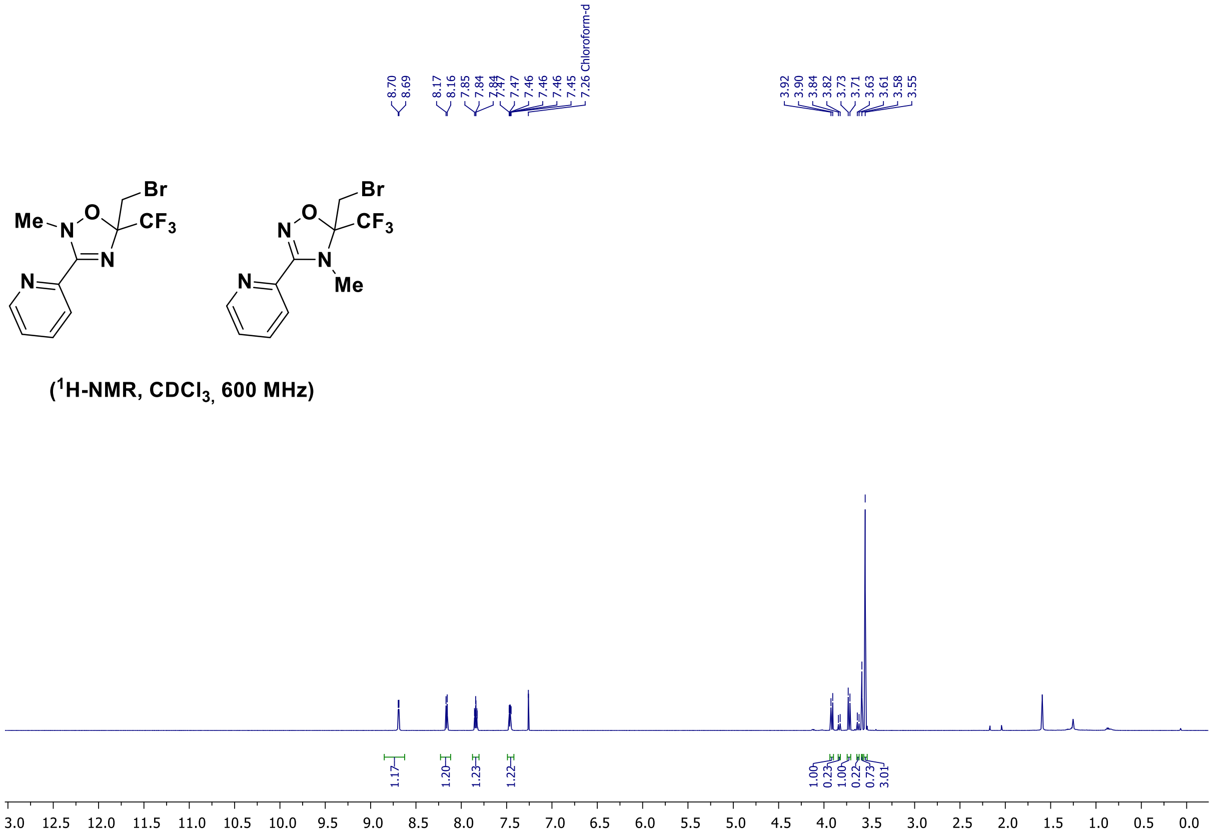

(^{1}H-NMR, $CDCl_3$, 600 MHz)

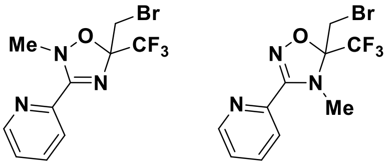

(¹³C-NMR, $CDCl_3$, 150 MHz)

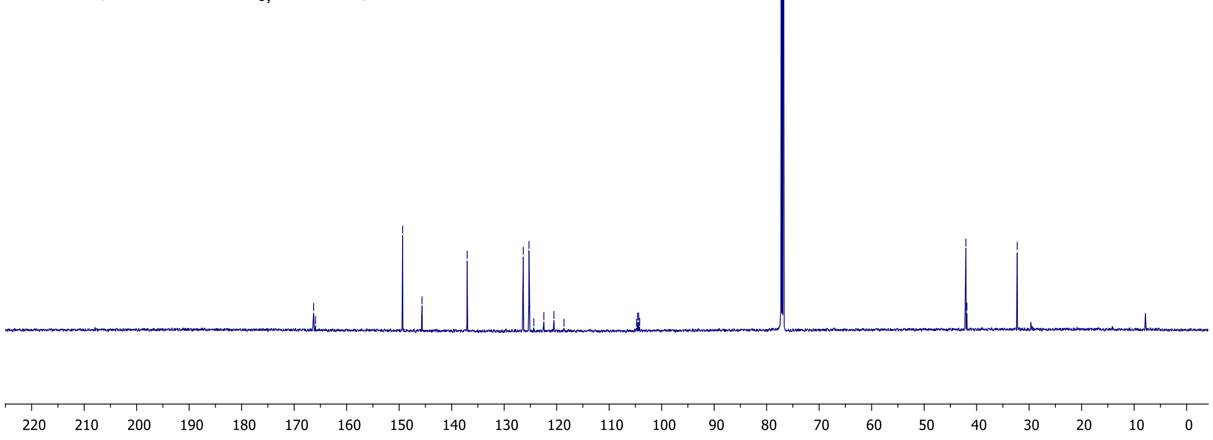

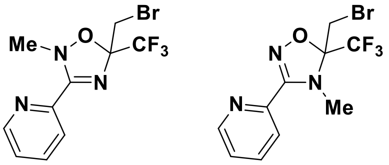

(¹⁹F-NMR, $CDCl_3$, 565 MHz)

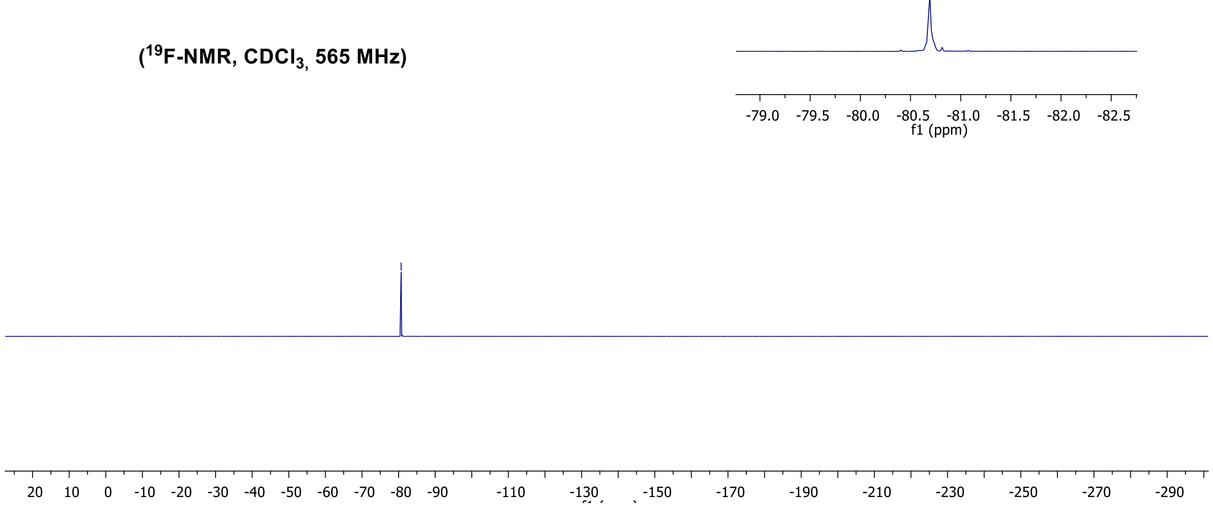

5-(bromomethyl)-2-methyl-3-(thiophen-2-yl)-5-(trifluoromethyl)-2,5-dihydro-1,2,4-oxadiazole (23) and 5-(bromomethyl)-4-methyl-3-(thiophen-2-yl)-5-(trifluoromethyl)-4,5-dihydro-1,2,4-oxadiazole (23a)

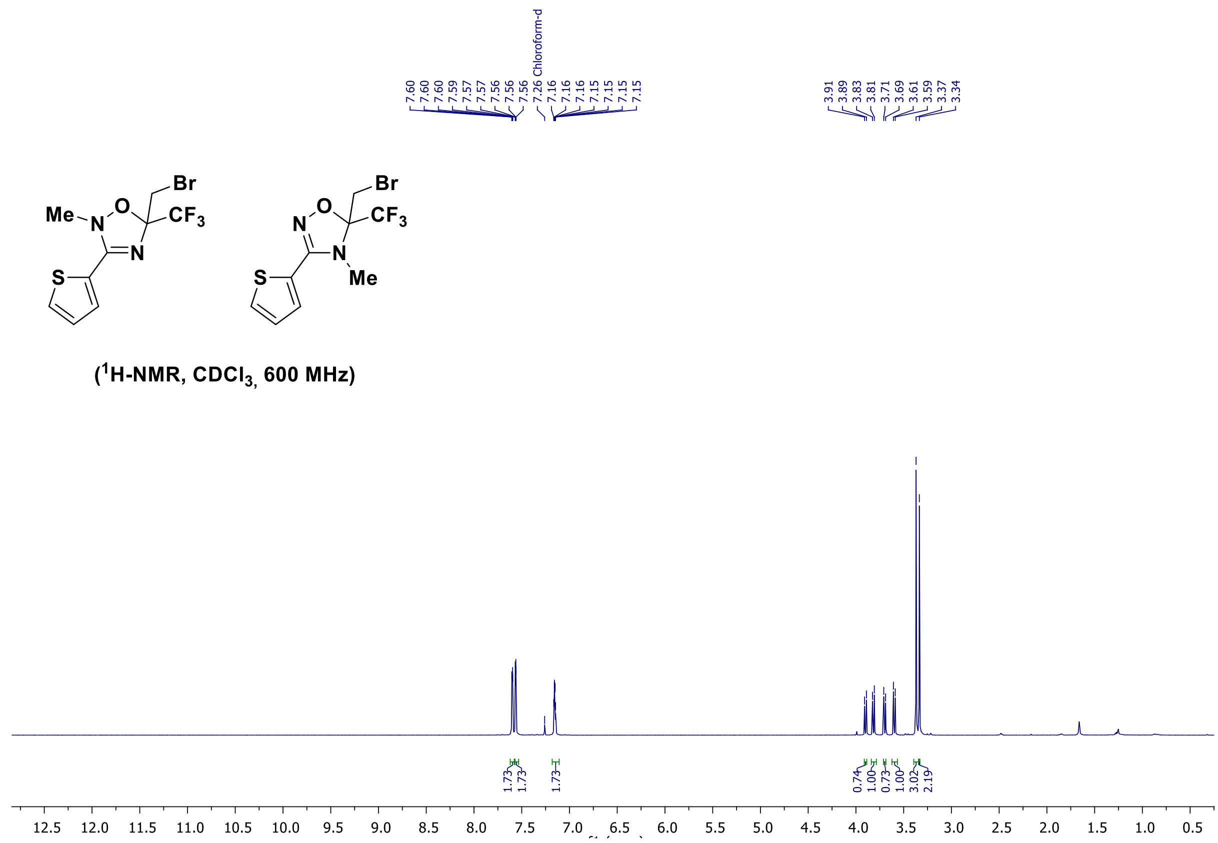

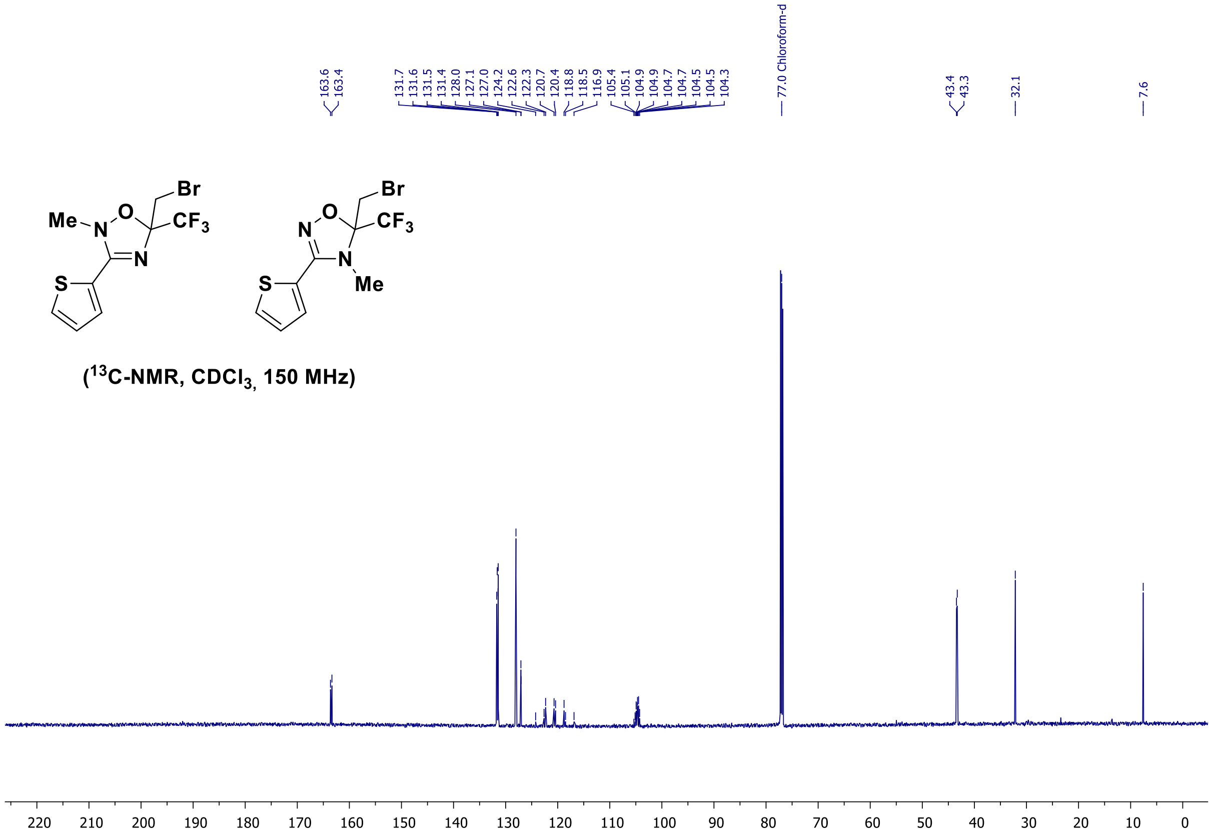

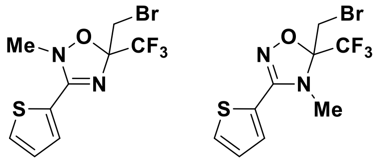

(^19^F-NMR, CDCl$_3$, 565 MHz)

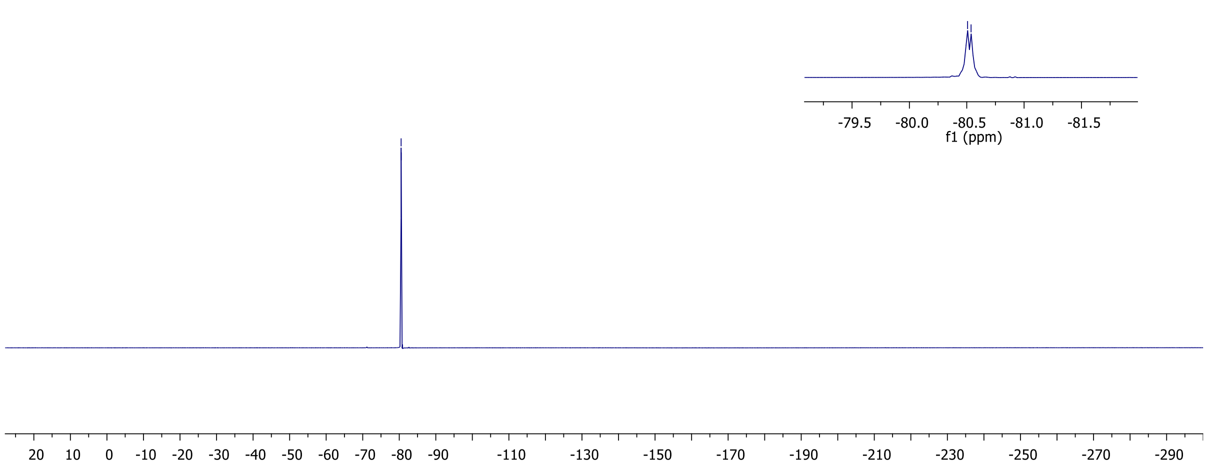

3-(4-bromophenyl)-5-(iodomethyl)-4-(methyl-*d*$_3$)-5-(trifluoromethyl)-4,5-dihydro-1,2,4-oxadiazole (24)

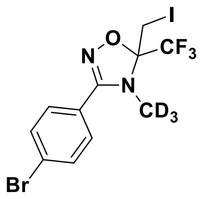

(^{1}H-NMR, CDCl$_3$, 600 MHz)

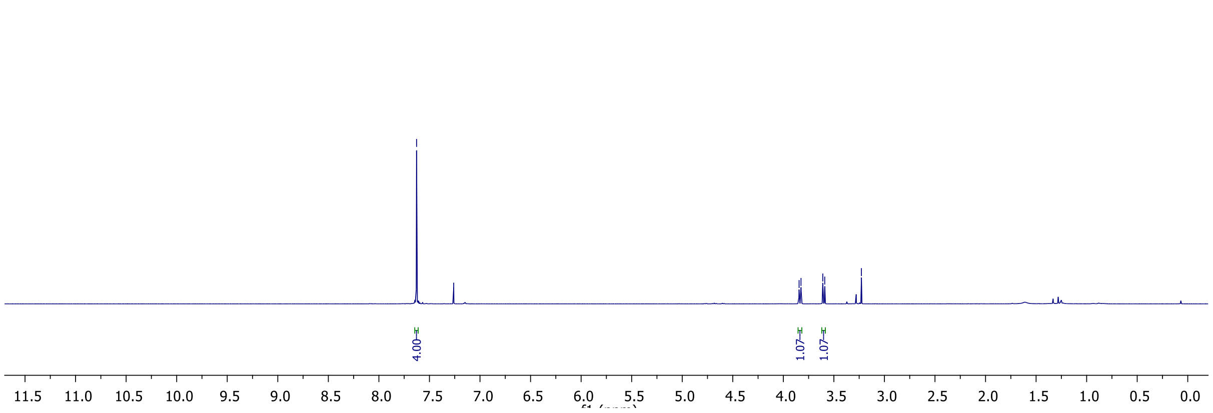

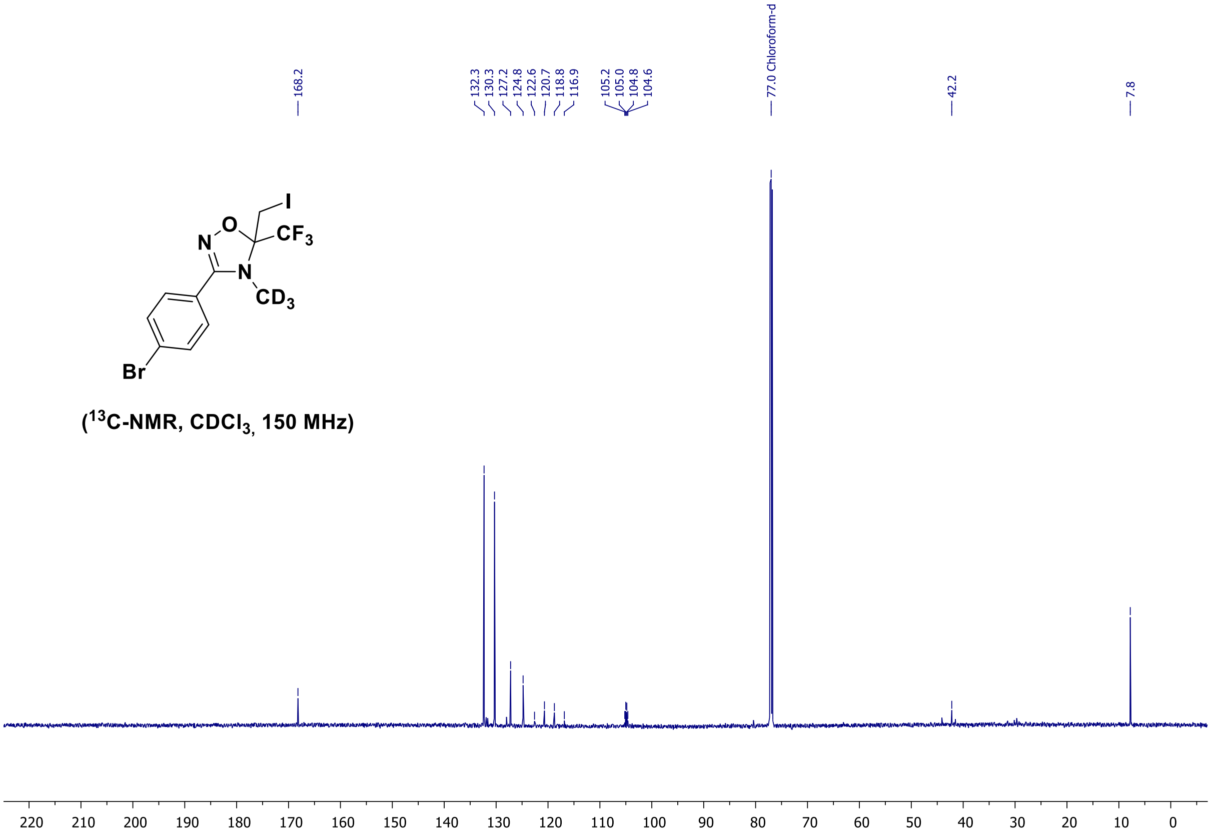

168.2
132.3
130.3
127.2
124.8
122.6
120.7
118.8
116.9
105.2
105.0
104.8
104.6
77.0 Chloroform-d
42.2
7.8
N
O
I
CF3
N
CD3
Br
(13C-NMR, CDCl3, 150 MHz)
220 210 200 190 180 170 160 150 140 130 120 110 100 90 80 70 60 50 40 30 20 10 0

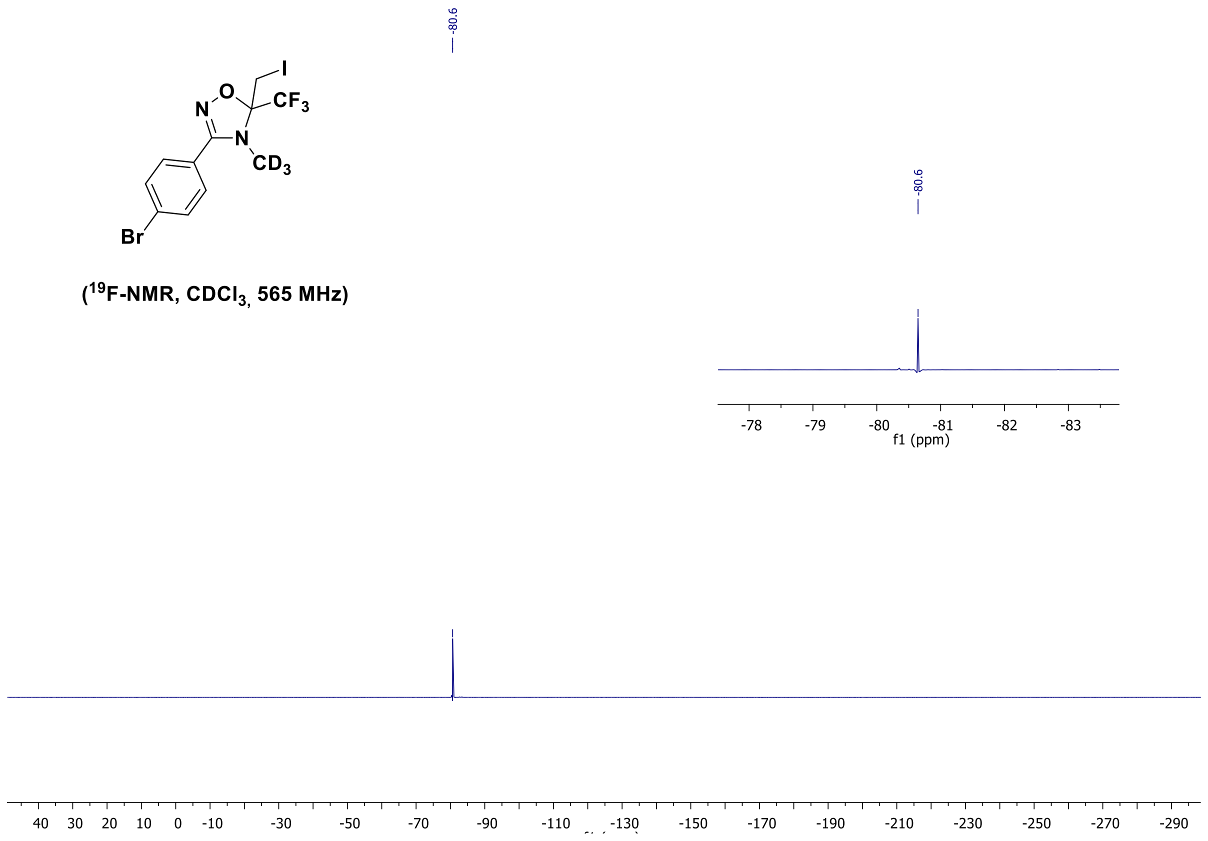

-80.6
N
O
I
CF3
N
CD3
Br
(19F-NMR, CDCl3, 565 MHz)
-80.6
-78 -79 -80 -81 -82 -83
f1 (ppm)
40 30 20 10 0 -10 -30 -50 -70 -90 -110 -130 -150 -170 -190 -210 -230 -250 -270 -290

3-(pyridin-4-yl)-5-(trifluoromethyl)-5-((trimethylsilyl)methyl)-2,5-dihydro-1,2,4-oxadiazole (25)

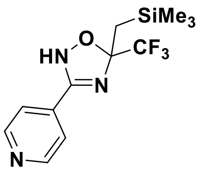

(^{1}H-NMR, $CDCl_3$, 600 MHz)

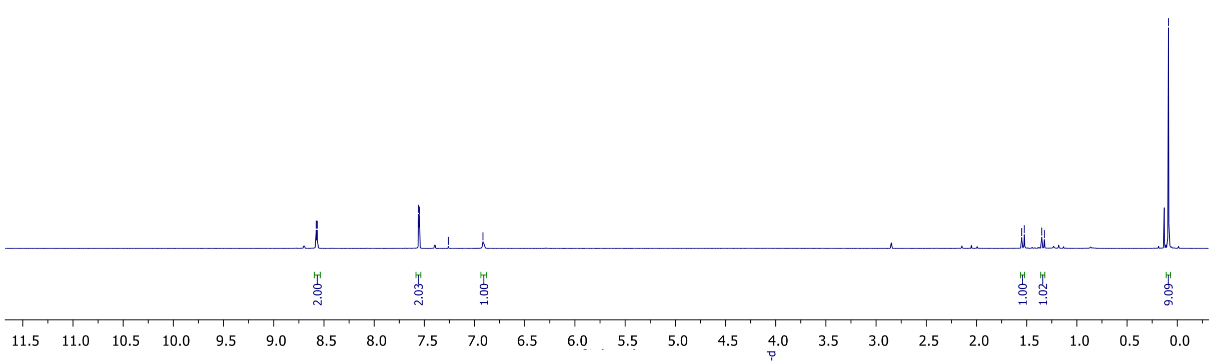

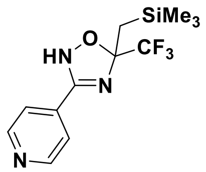

(^{13}C-NMR, $CDCl_3$, 150 MHz)

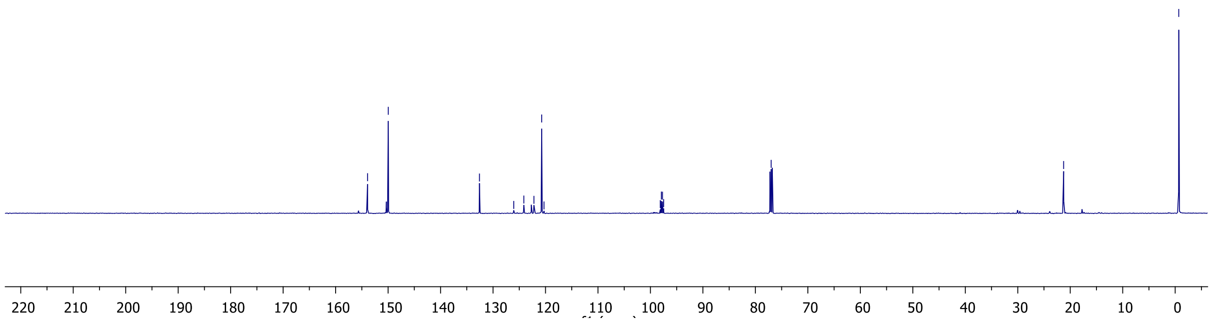

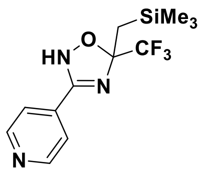

(^{19}F-NMR, $CDCl_3$, 565 MHz)

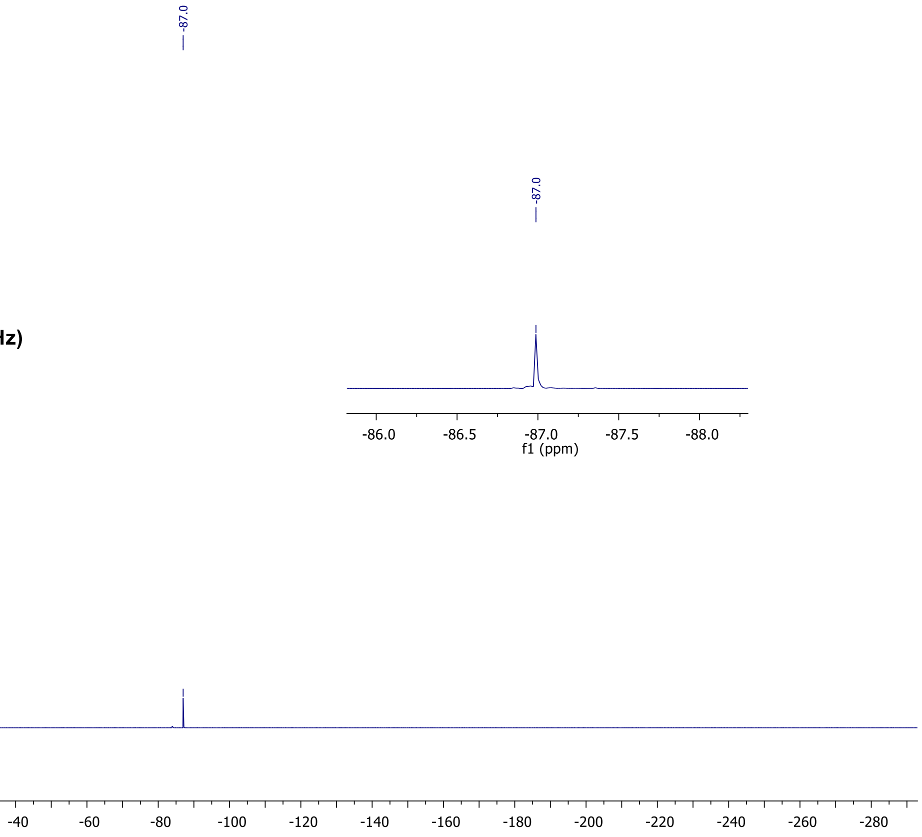

3-(4-methoxyphenyl)-5-methyl-5-(trifluoromethyl)-2,5-dihydro-1,2,4-oxadiazole (26)

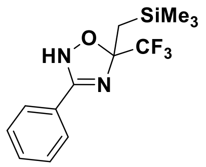

(^{1}H-NMR, $CDCl_3$, 600 MHz)

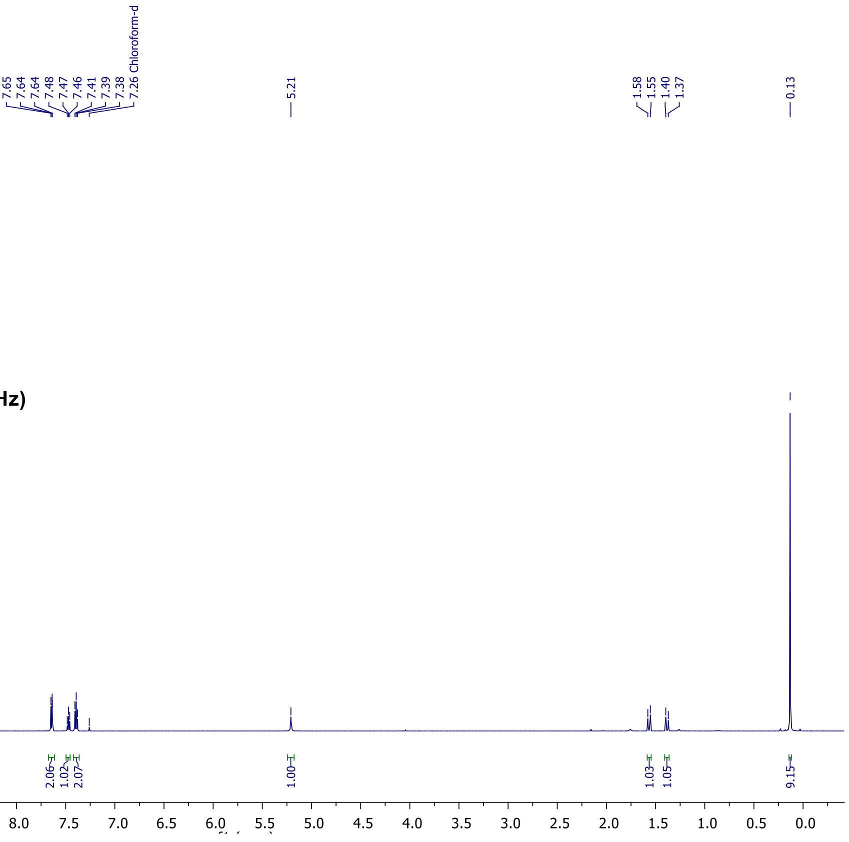

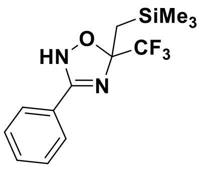

(^{13}C-NMR, $CDCl_3$, 150 MHz)

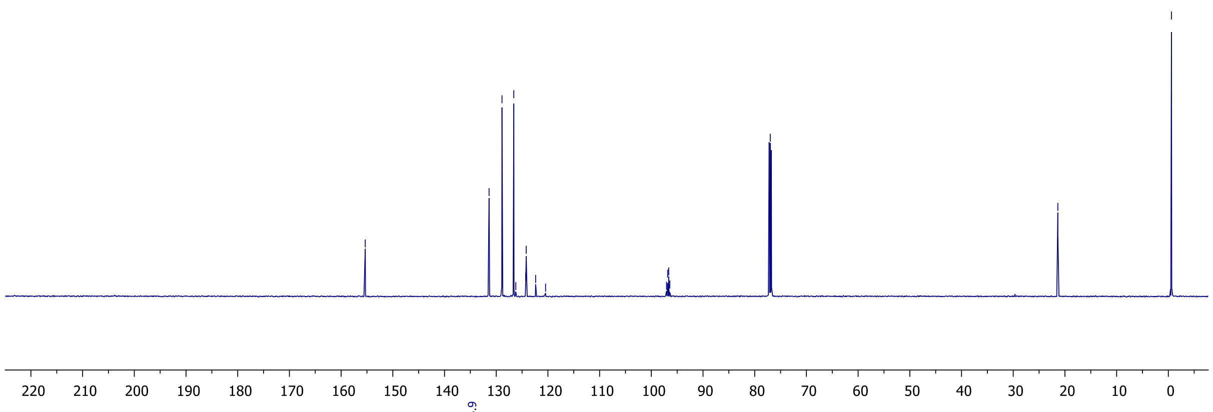

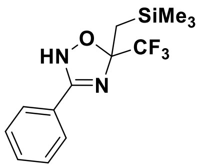

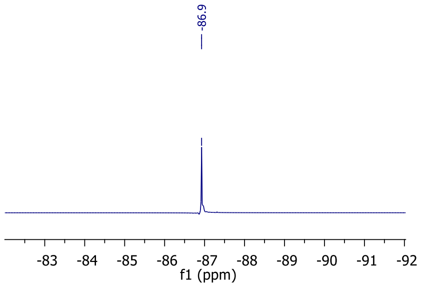

(^{19}F-NMR, $CDCl_3$, 565 MHz)

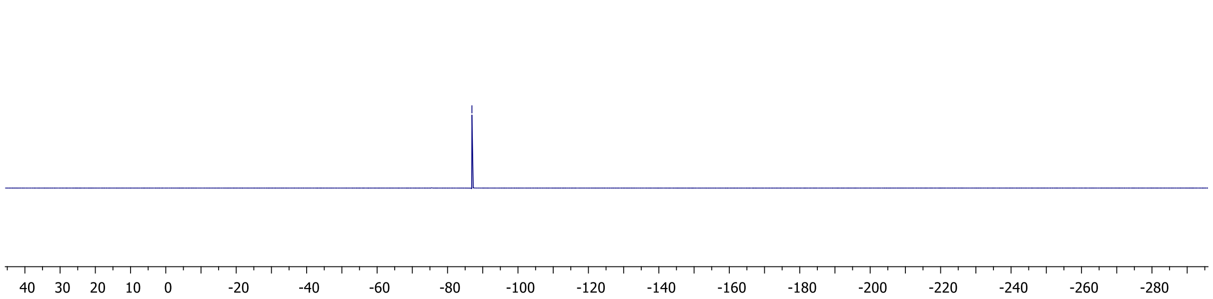

2-(3-phenyl-5-(trifluoromethyl)-2,5-dihydro-1,2,4-oxadiazol-5-yl)acetonitrile (27)

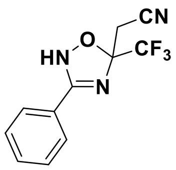

(¹H-NMR, Acetone-d_6, 400 MHz)

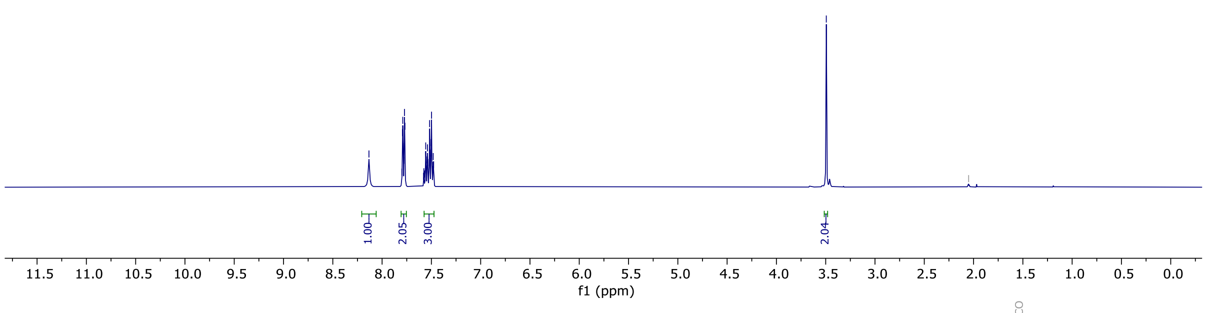

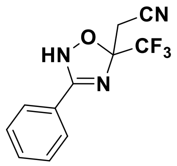

(¹³C-NMR, Acetone-d_6, 100 MHz)

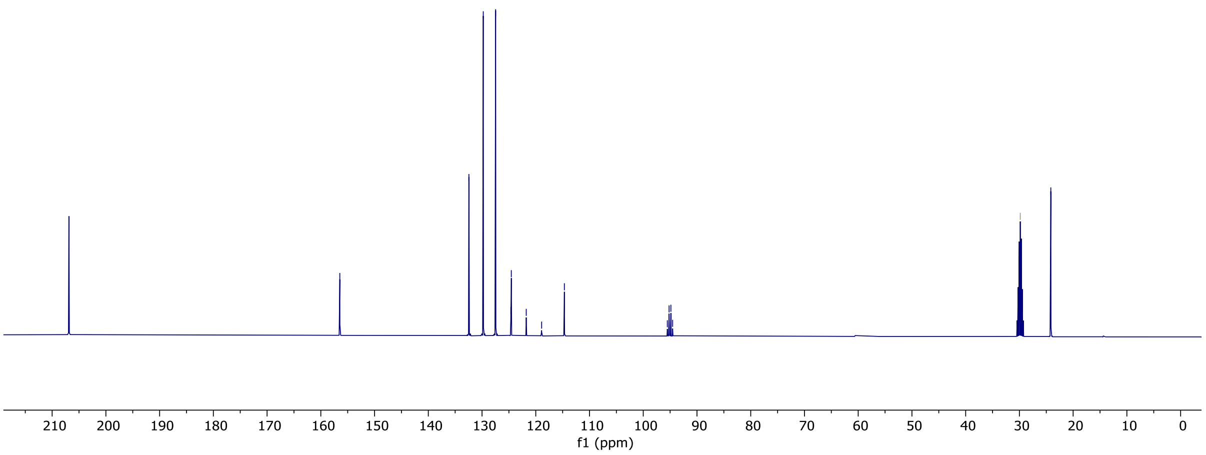

(^19^F-NMR, Acetone-d_6, 377 MHz)

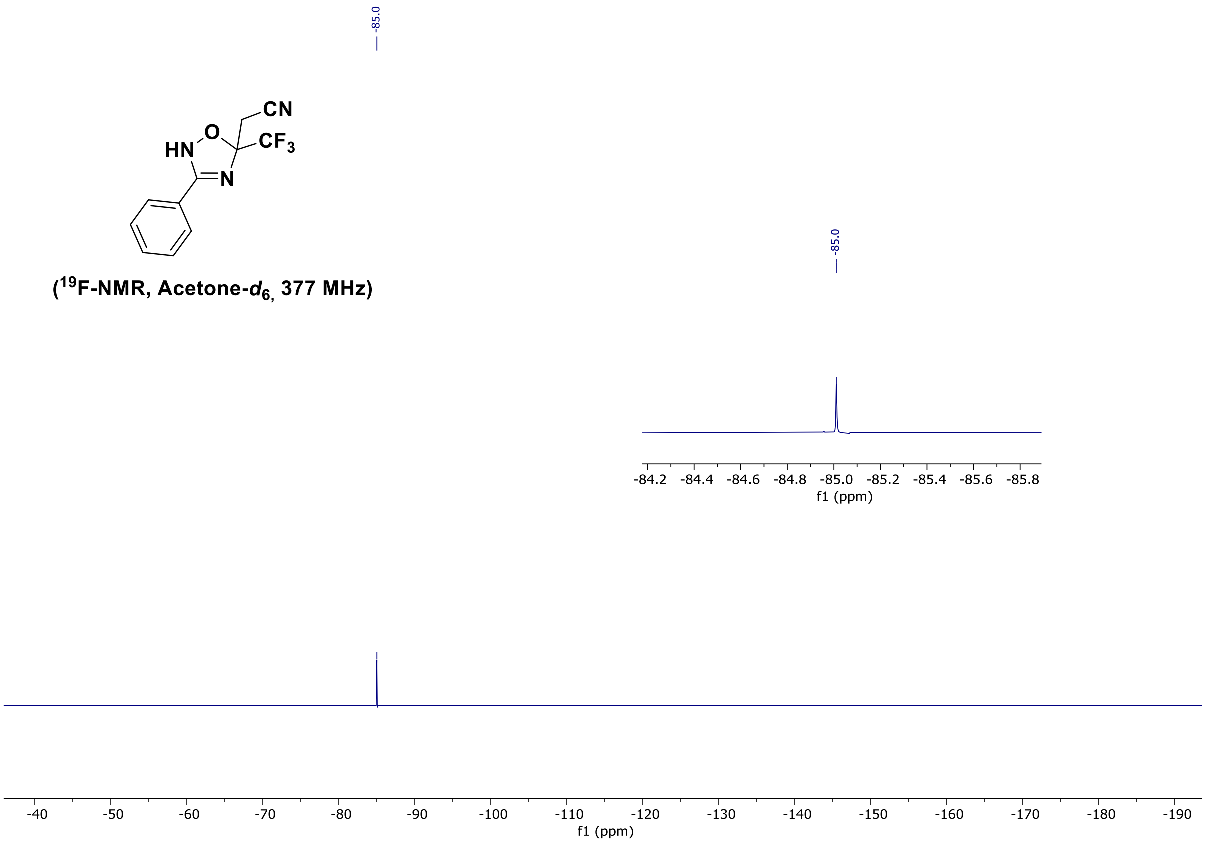

5-(methoxymethyl)-3-phenyl-5-(trifluoromethyl)-2,5-dihydro-1,2,4-oxadiazole (28)

(^1^H-NMR, Acetone-d_6, 400 MHz)

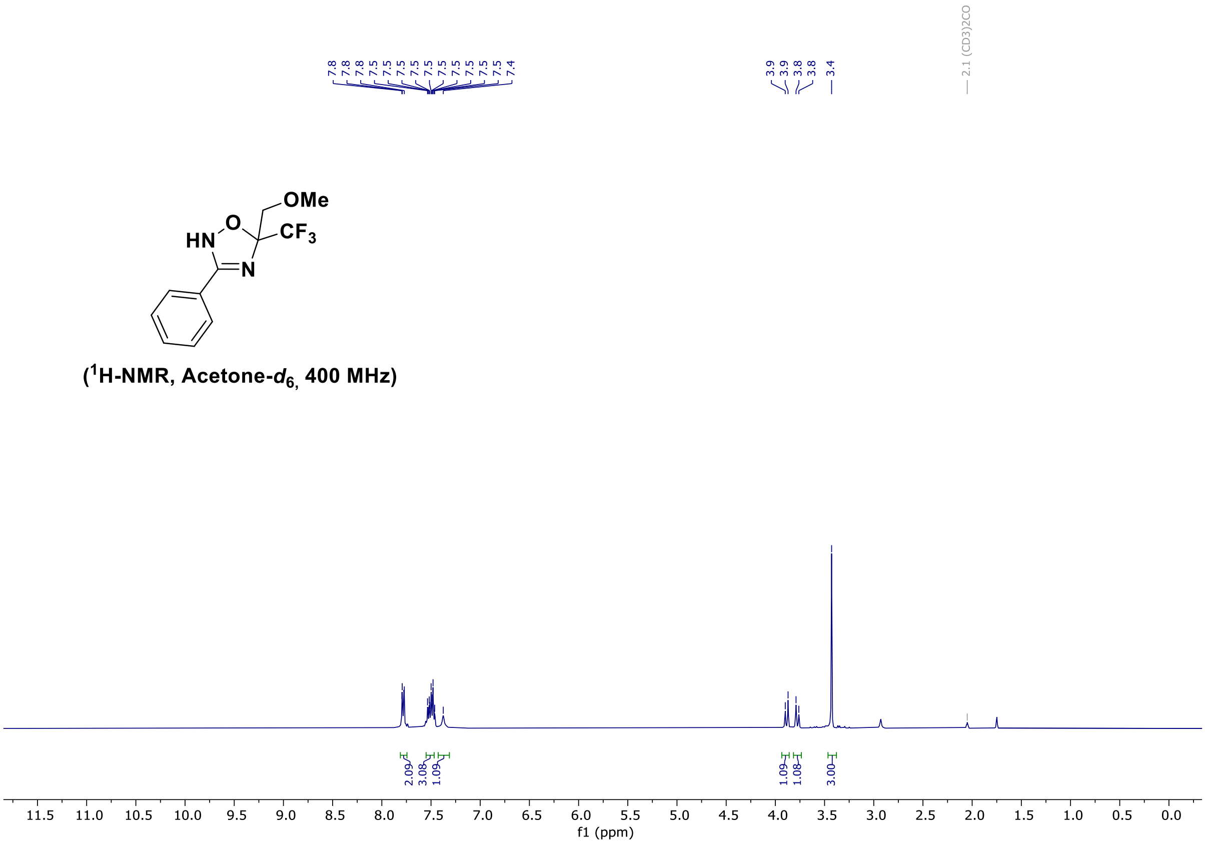

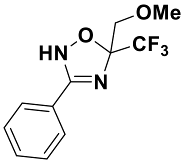

(^{13}C-NMR, Acetone-d_6, 100 MHz)

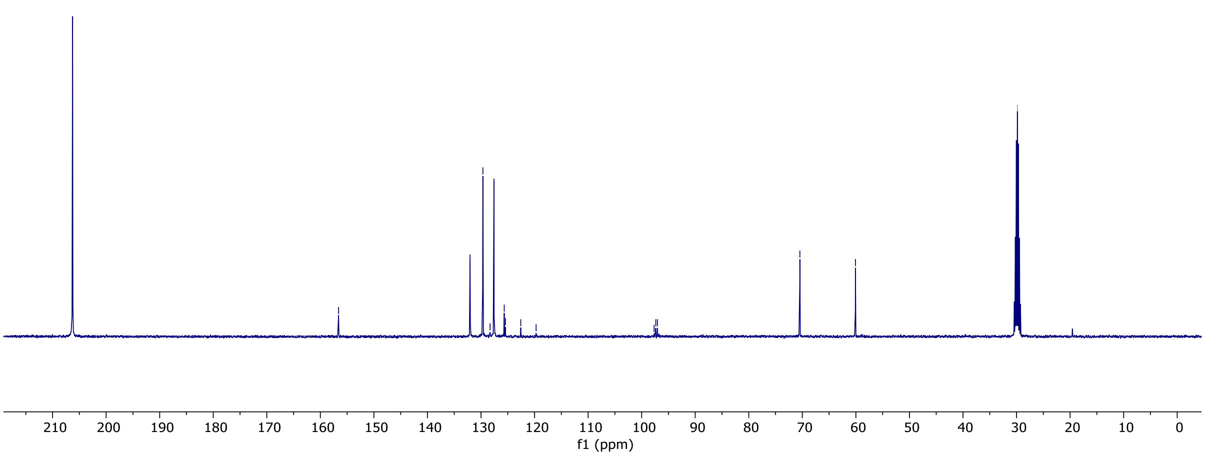

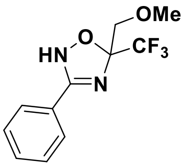

(^{19}F-NMR, Acetone-d_6, 377 MHz)

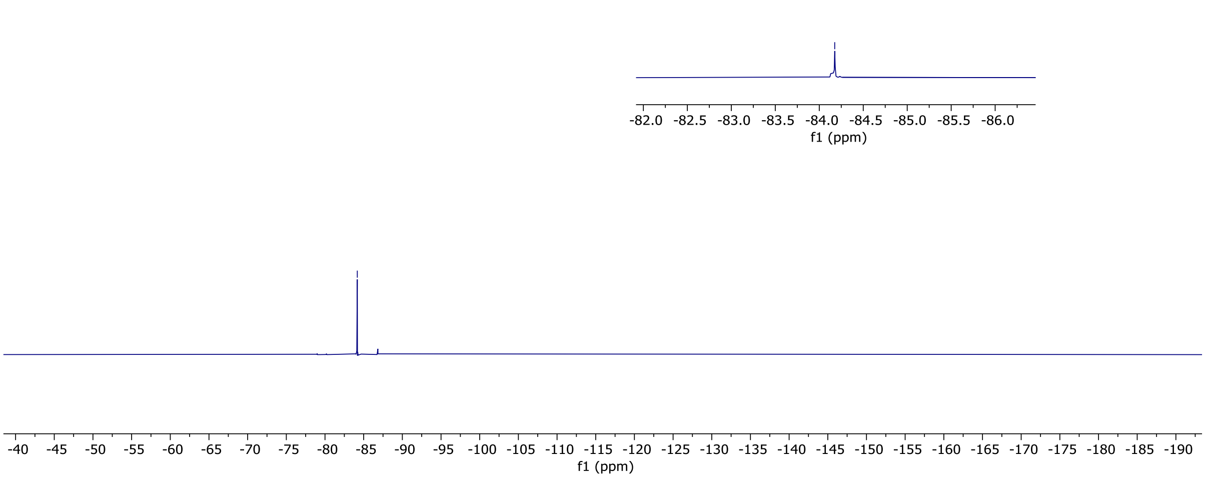

5-((methylthio)methyl)-3-phenyl-5-(trifluoromethyl)-2,5-dihydro-1,2,4-oxadiazole (29)

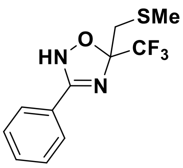

(¹H-NMR, Acetone-d_6, 400 MHz)

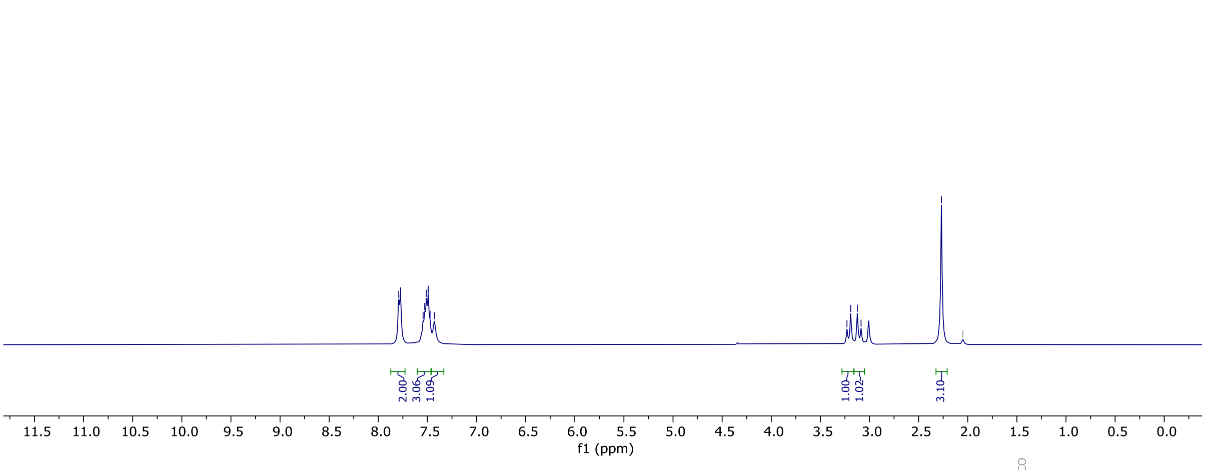

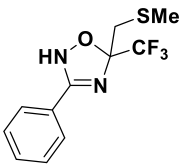

(¹³C-NMR, Acetone-d_6, 100 MHz)

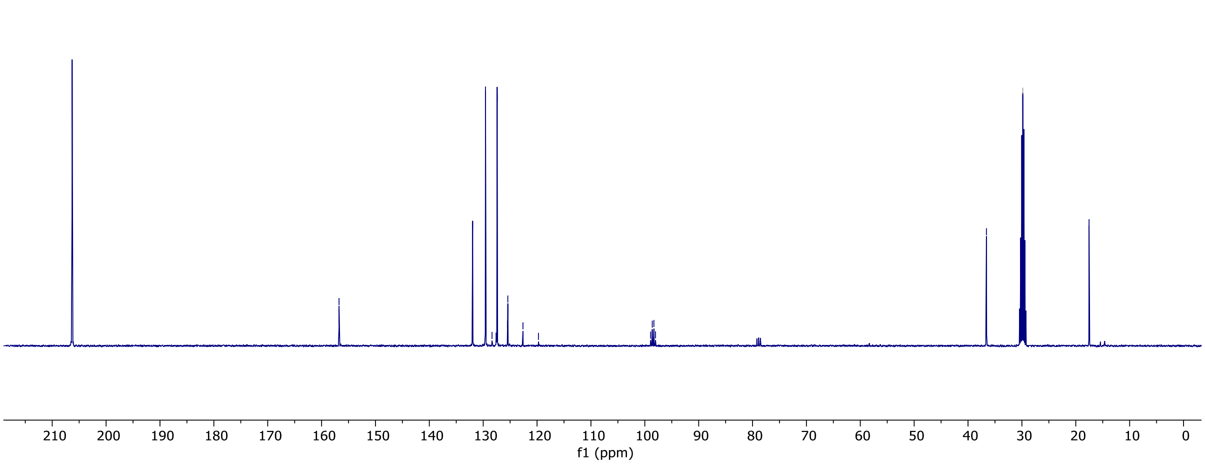

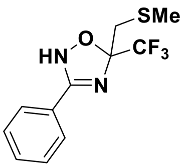

(^{19}F-NMR, Acetone-d_6, 377 MHz)

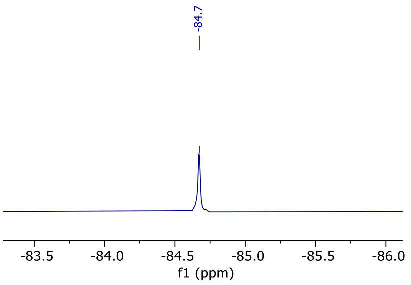

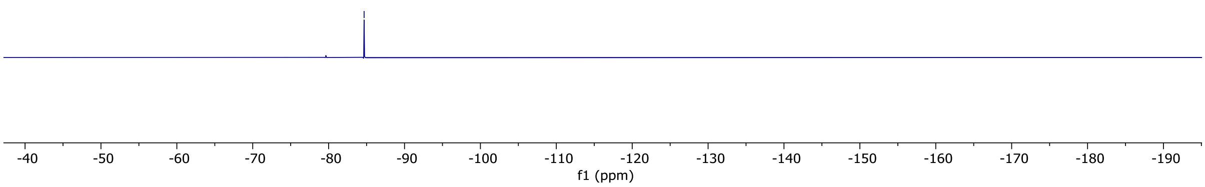

3-phenyl-5-((phenylselanyl)methyl)-5-(trifluoromethyl)-2,5-dihydro-1,2,4-oxadiazole (30)

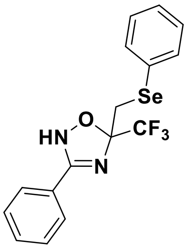

(^{1}H-NMR, Acetone-d_6, 400 MHz)

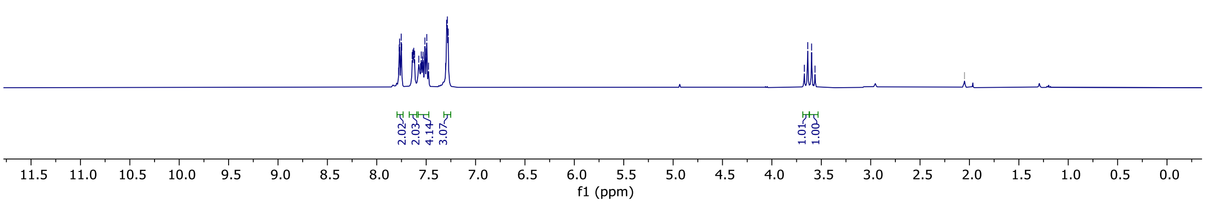

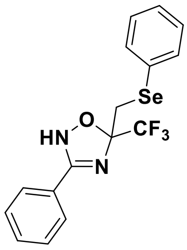

(^{13}C-NMR, Acetone-d_6, 100 MHz)

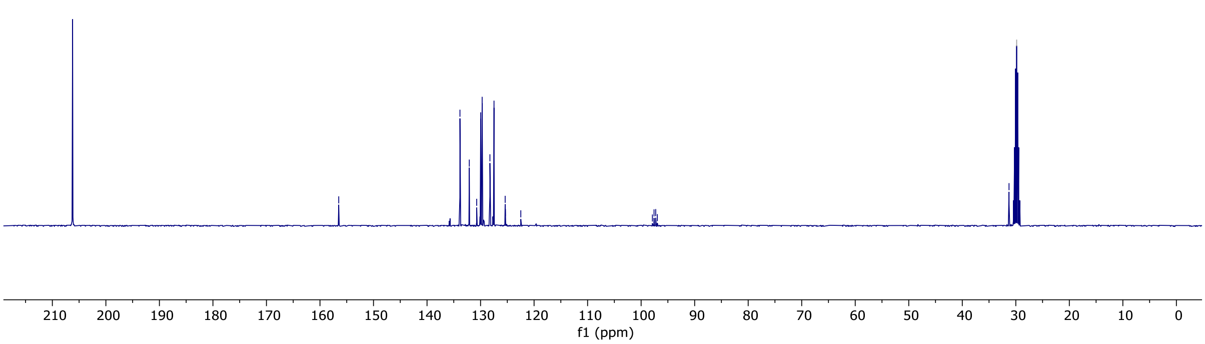

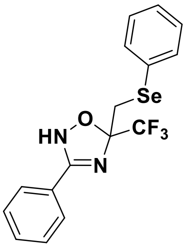

(^{19}F-NMR, Acetone-d_6, 377 MHz)

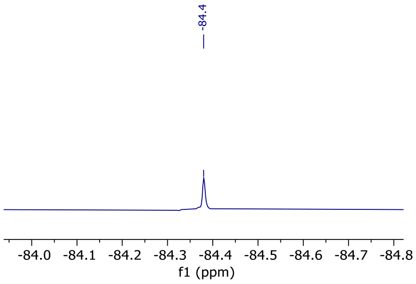

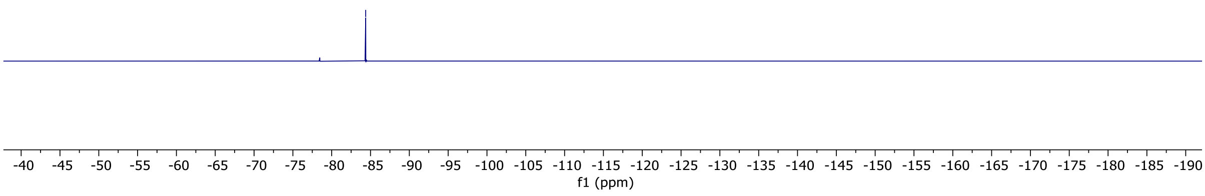

3-phenyl-5,5-bis(trifluoromethyl)-2,5-dihydro-1,2,4-oxadiazole (31)

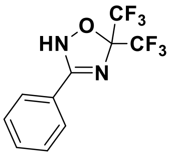

(^{1}H-NMR, Acetone-d_6, 400 MHz)

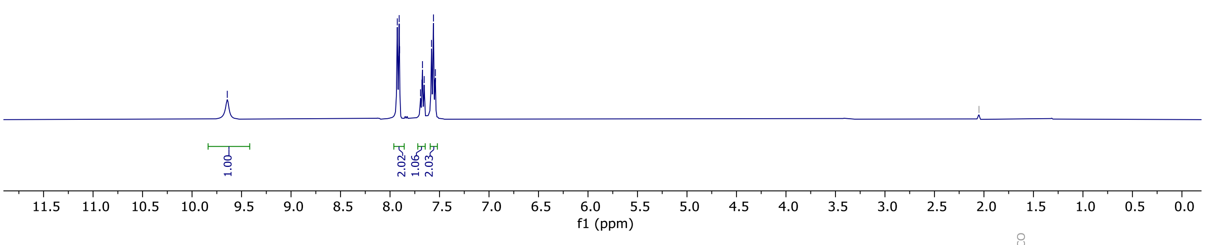

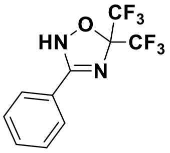

(^{13}C-NMR, Acetone-d_6, 100 MHz)

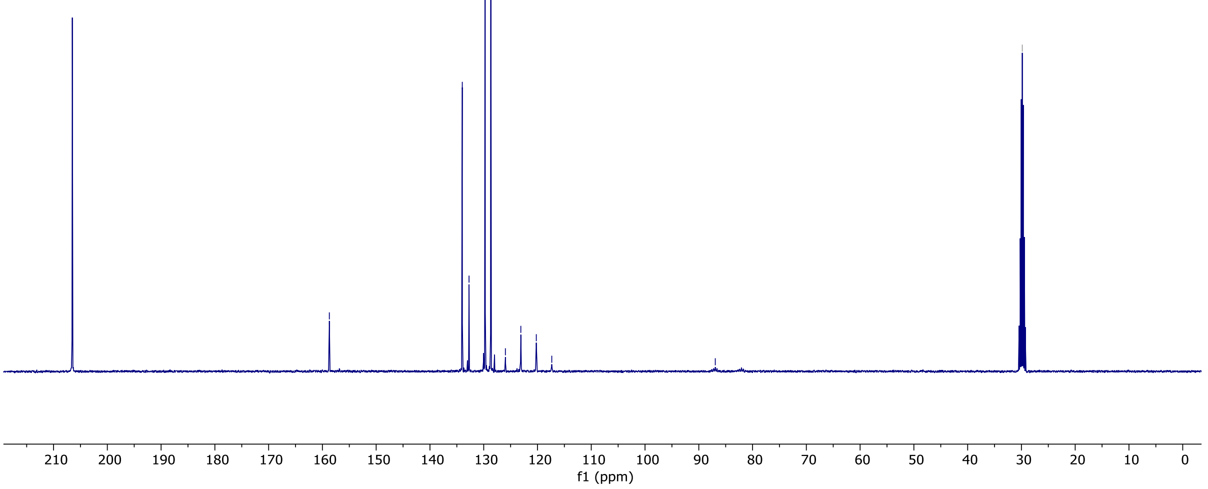

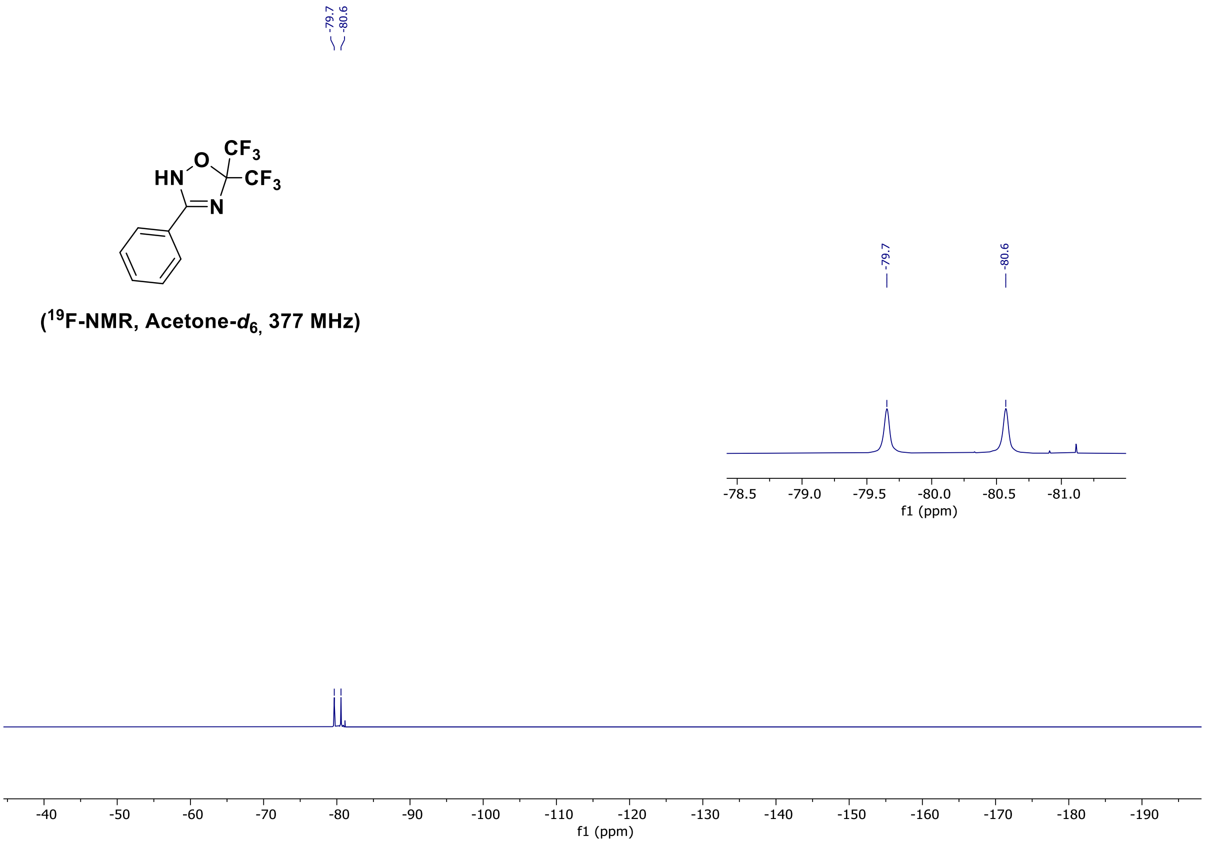

(^{19}F-NMR, Acetone-d_6, 377 MHz)

5-(difluoromethyl)-3-phenyl-5-(trifluoromethyl)-2,5-dihydro-1,2,4-oxadiazole (32)

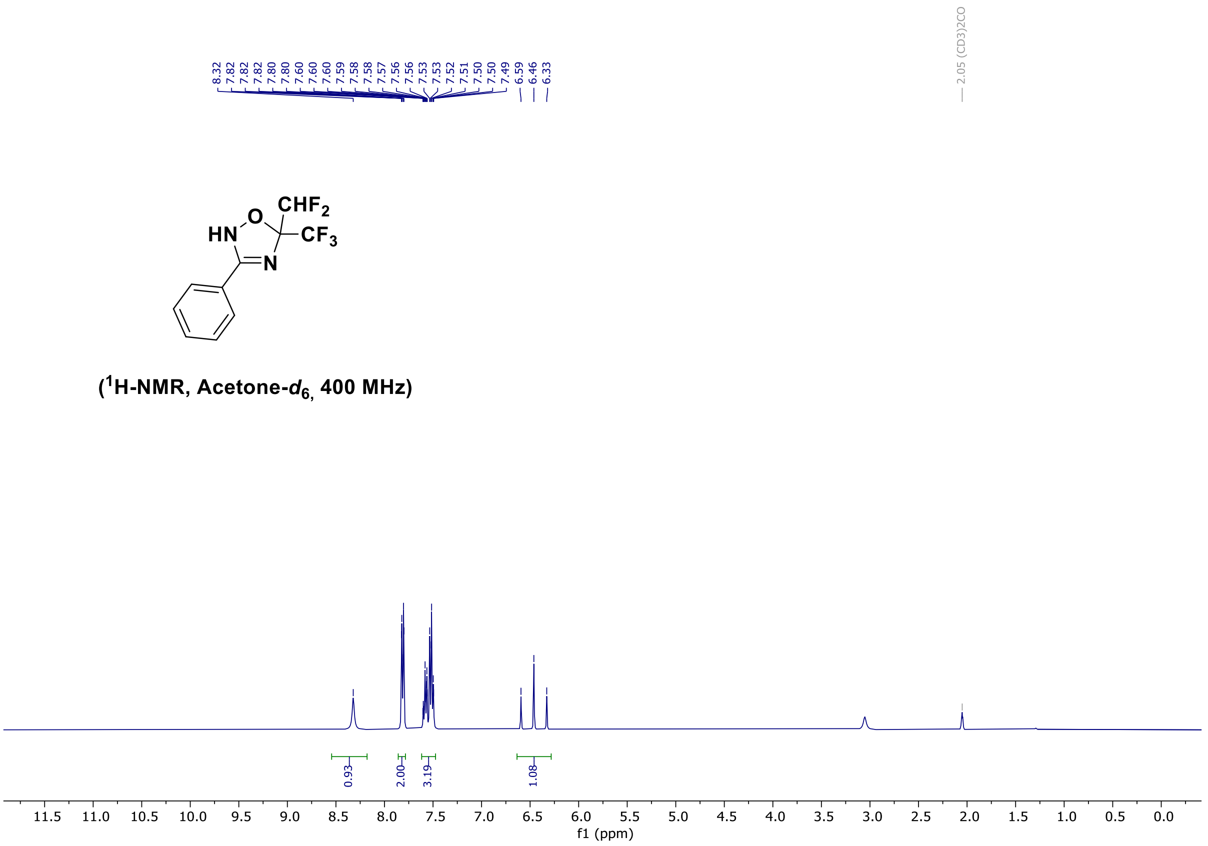

(^{1}H-NMR, Acetone-d_6, 400 MHz)

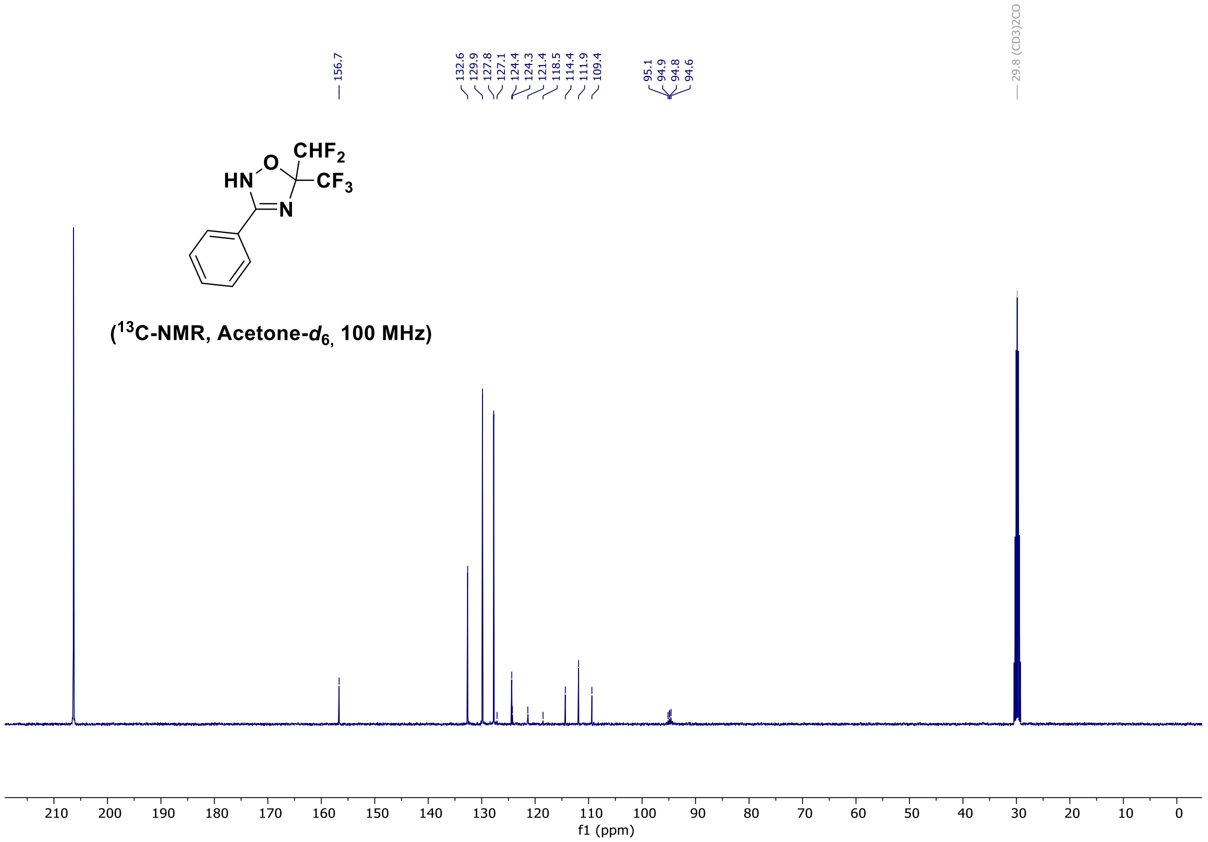
156.7
132.6
129.9
127.8
127.1
124.4
124.3
121.4
118.5
114.4
111.9
109.4
95.1
94.9
94.8
94.6
29.8 (CD3)2CO
CHF2
HN
O
CF3
N
(13C-NMR, Acetone-d6, 100 MHz)
210 200 190 180 170 160 150 140 130 120 110 100 90 80 70 60 50 40 30 20 10 0
f1 (ppm)

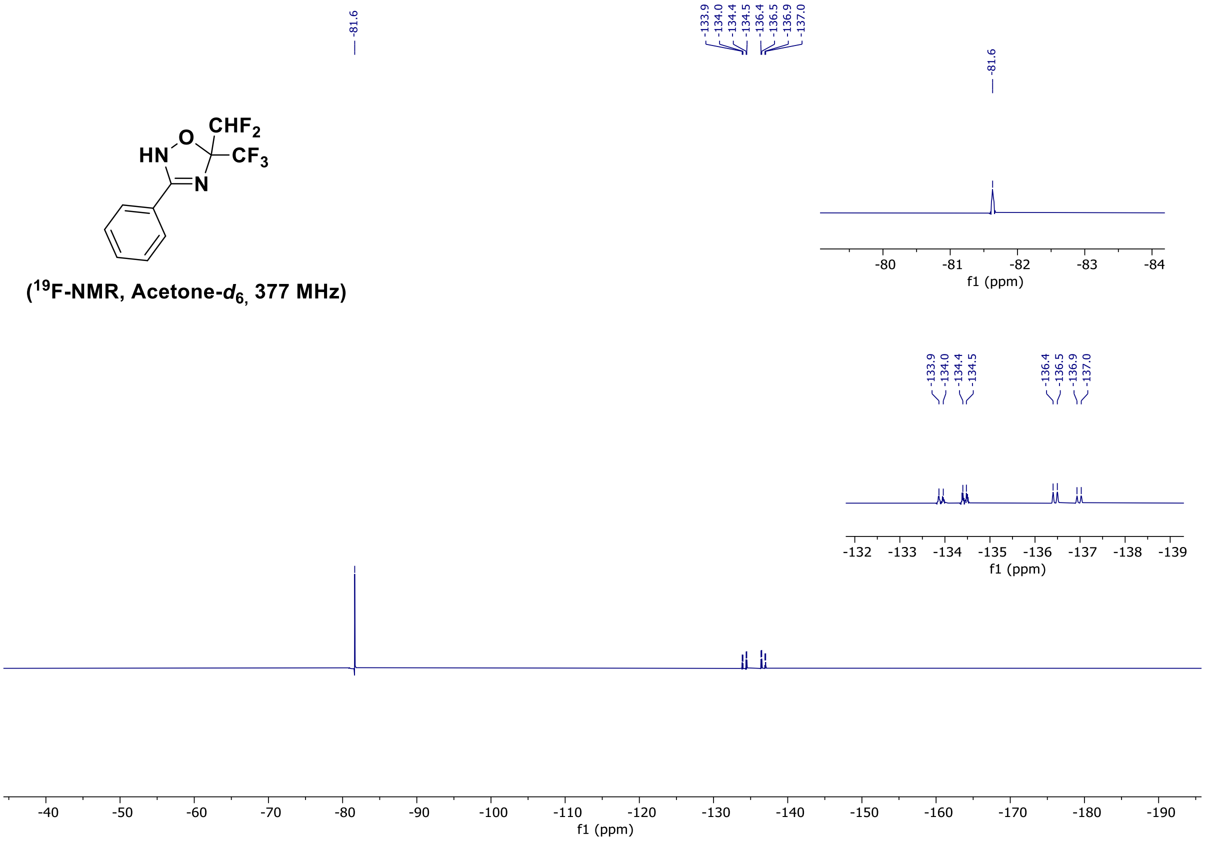
-81.6
-133.9
-134.0
-134.4
-134.5
-136.4
-136.5
-136.9
-137.0
CHF2
HN
O
CF3
N
(19F-NMR, Acetone-d6, 377 MHz)
-80 -81 -82 -83 -84
f1 (ppm)
-132 -133 -134 -135 -136 -137 -138 -139
f1 (ppm)
-40 -50 -60 -70 -80 -90 -100 -110 -120 -130 -140 -150 -160 -170 -180 -190
f1 (ppm)

3-(4-methoxyphenyl)-5-methyl-5-(trifluoromethyl)-2,5-dihydro-1,2,4-oxadiazole (33)

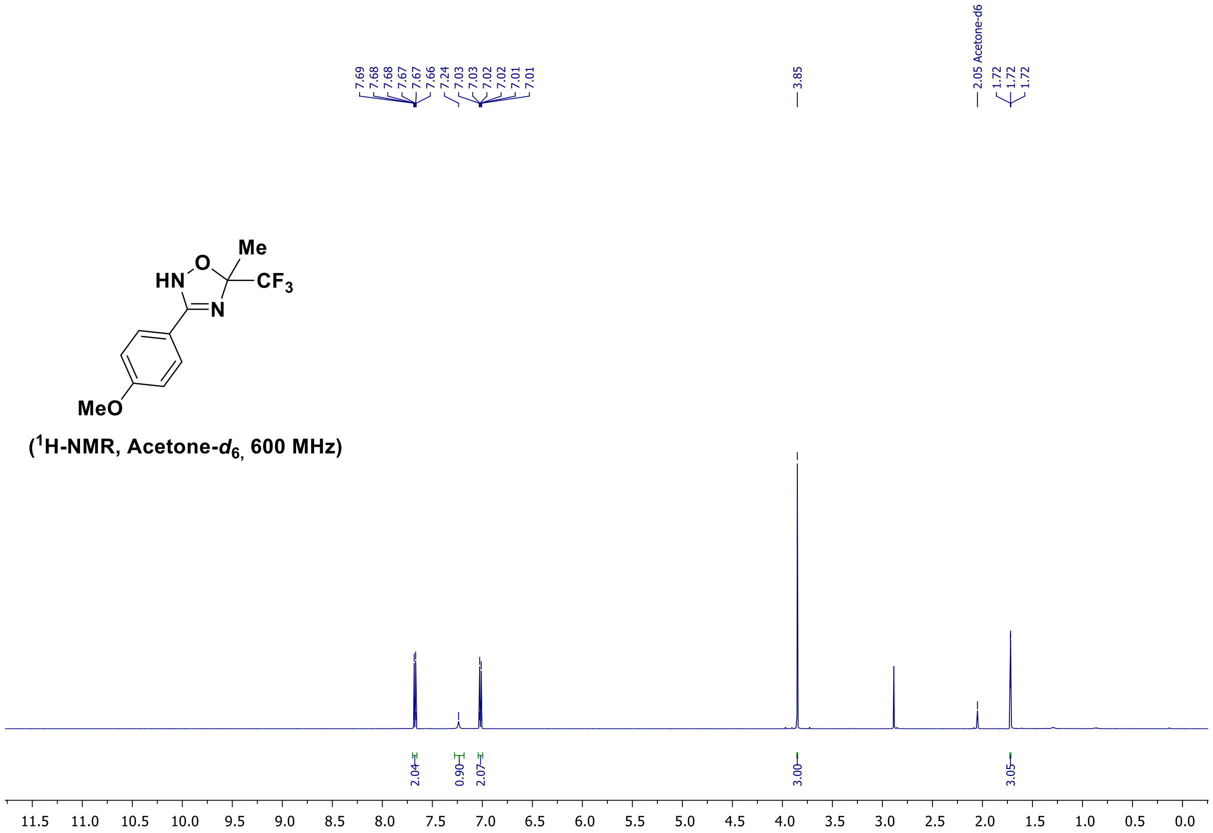

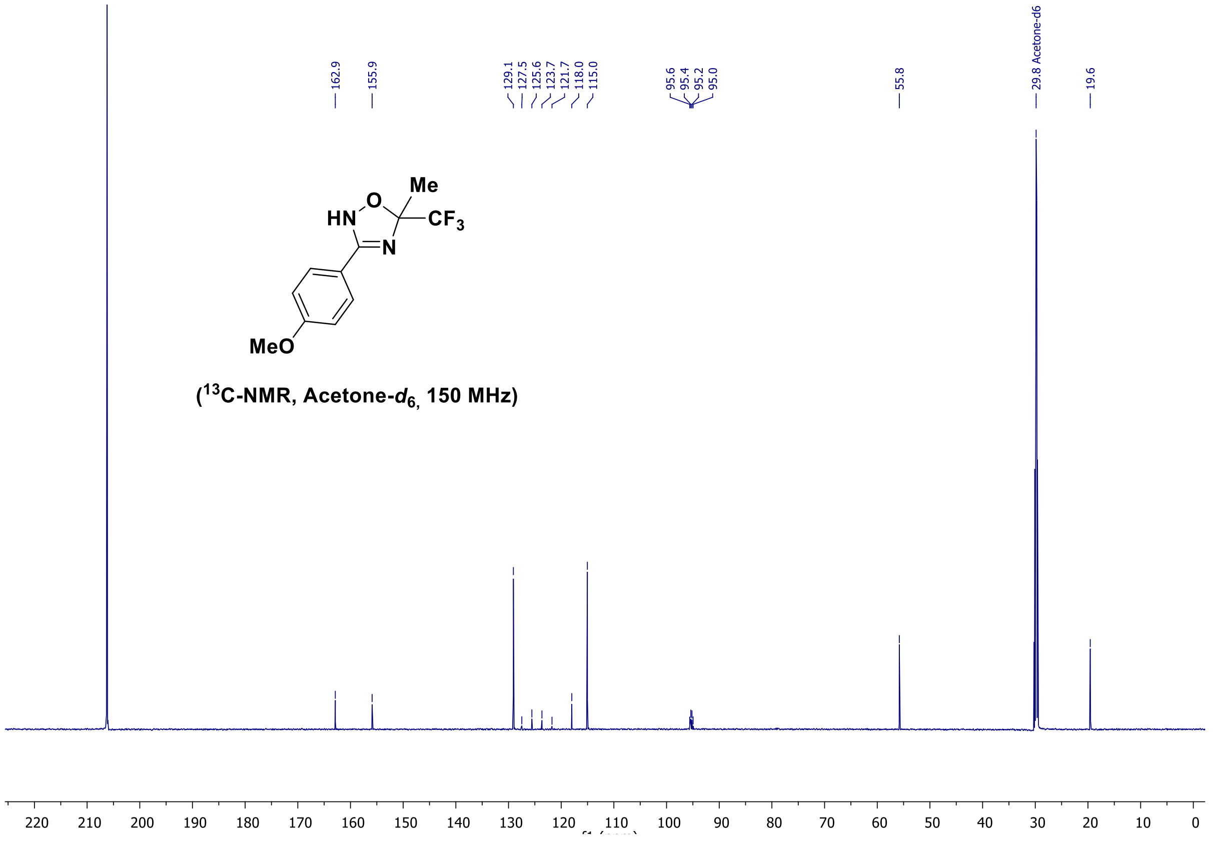

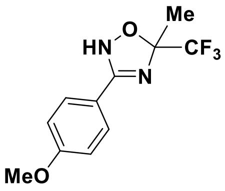

(^{19}F-NMR, Acetone-d_6, 565 MHz)

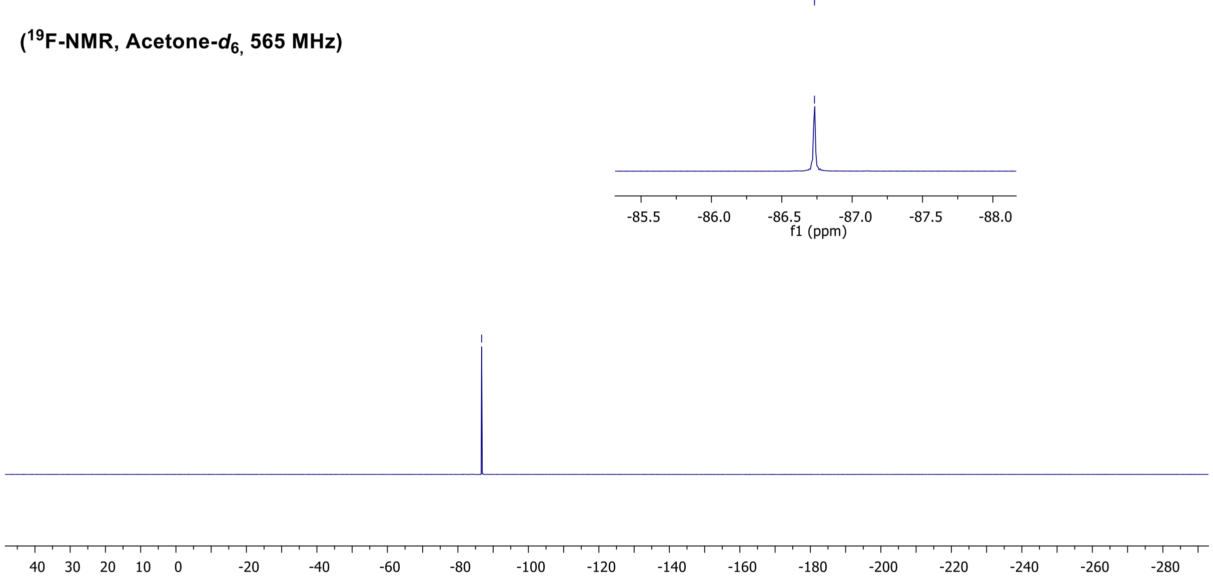

5-butyl-3-phenyl-5-(trifluoromethyl)-2,5-dihydro-1,2,4-oxadiazole (34)

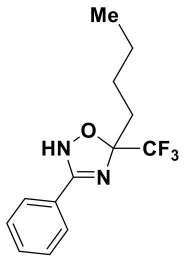

(^{1}H-NMR, Acetone-d_6, 600 MHz)

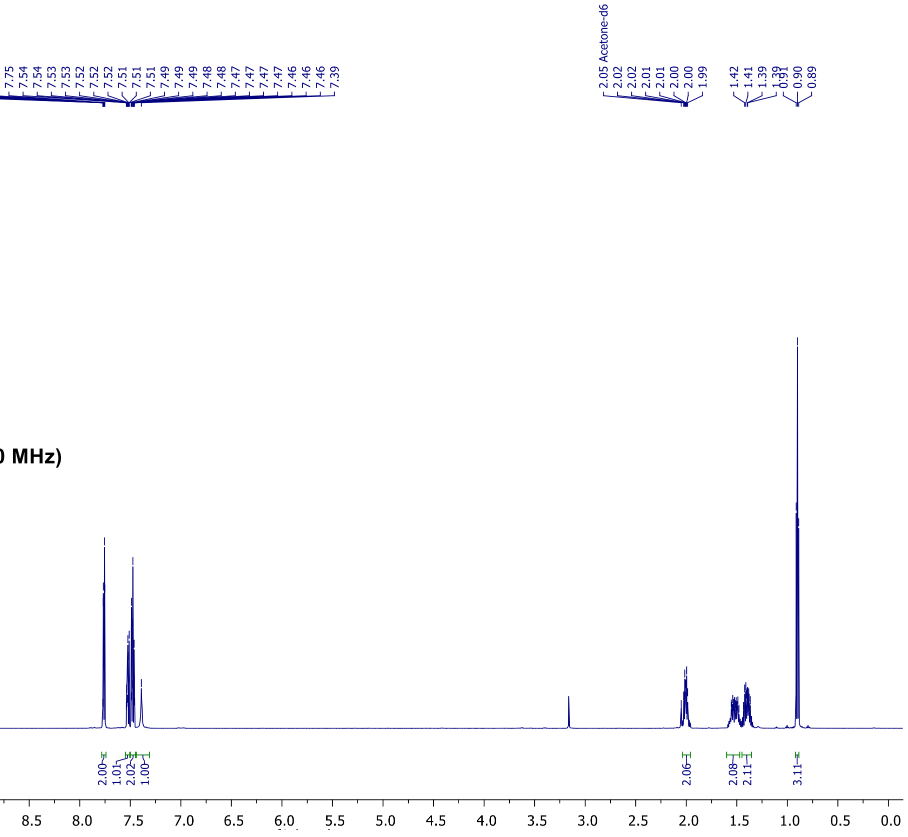

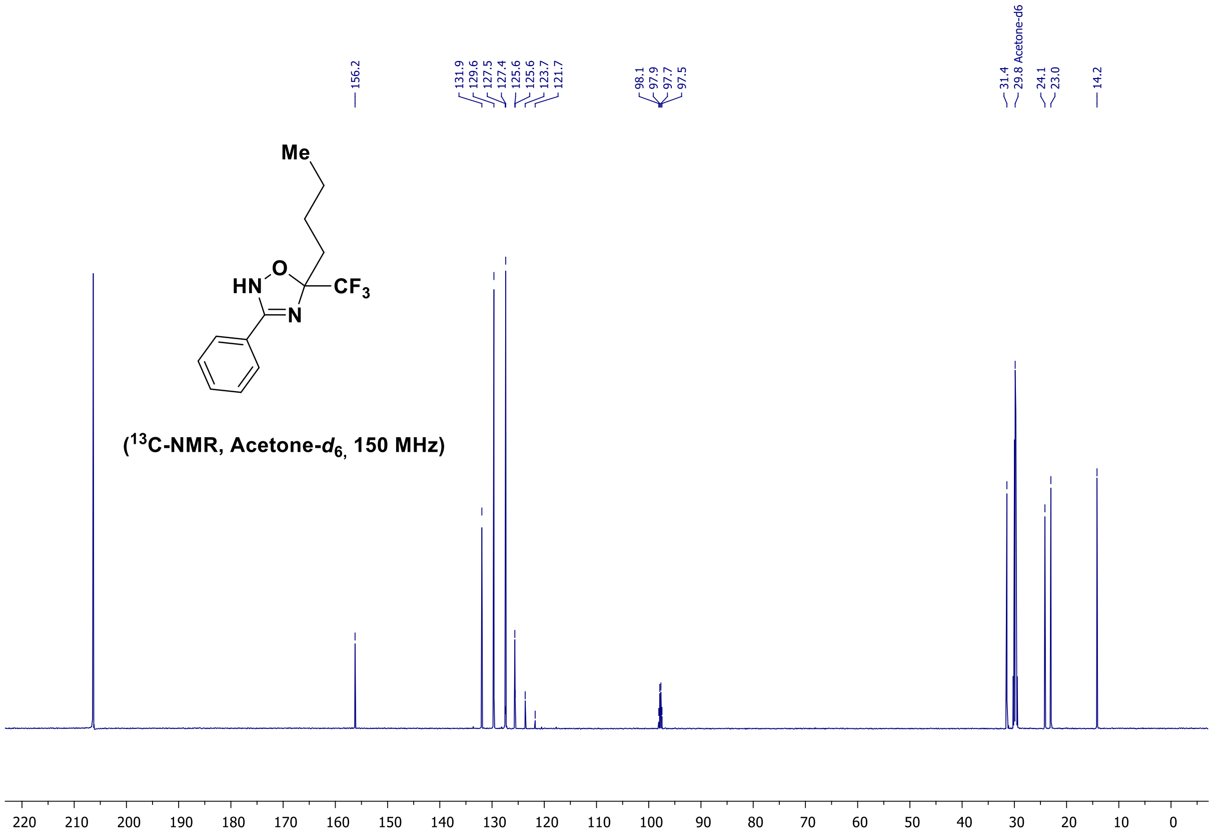

(^{13}C-NMR, Acetone-d_6, 150 MHz)

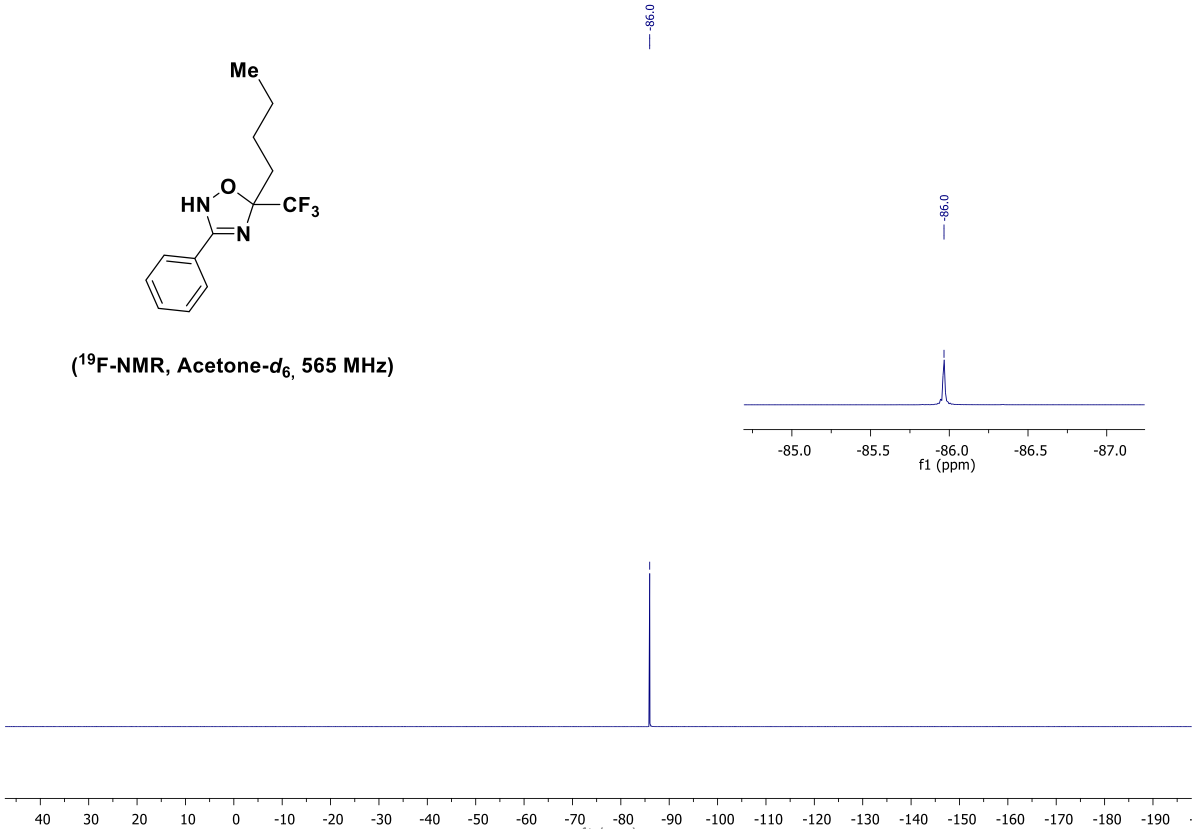

(^{19}F-NMR, Acetone-d_6, 565 MHz)

5-(*sec*-butyl)-3-phenyl-5-(trifluoromethyl)-2,5-dihydro-1,2,4-oxadiazole (35)

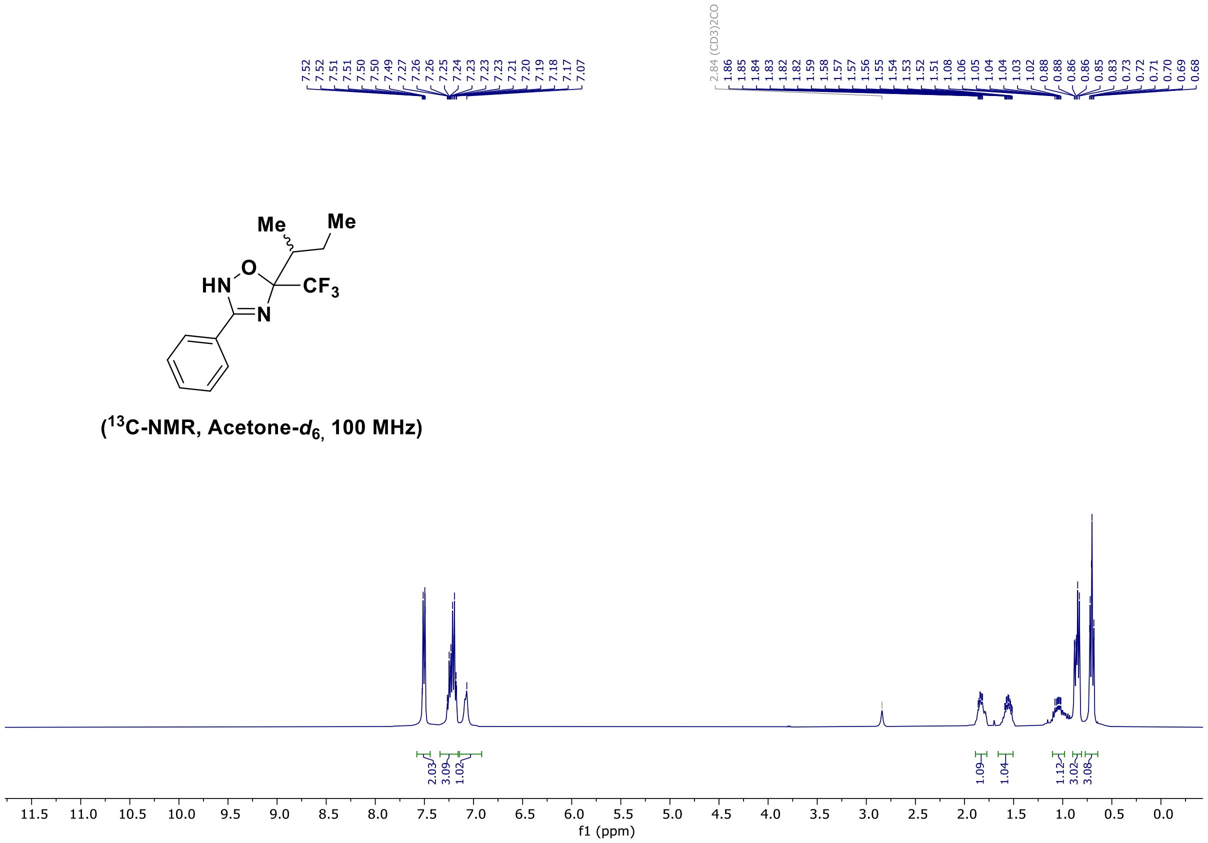

(^{13}C-NMR, Acetone-d_6, 100 MHz)

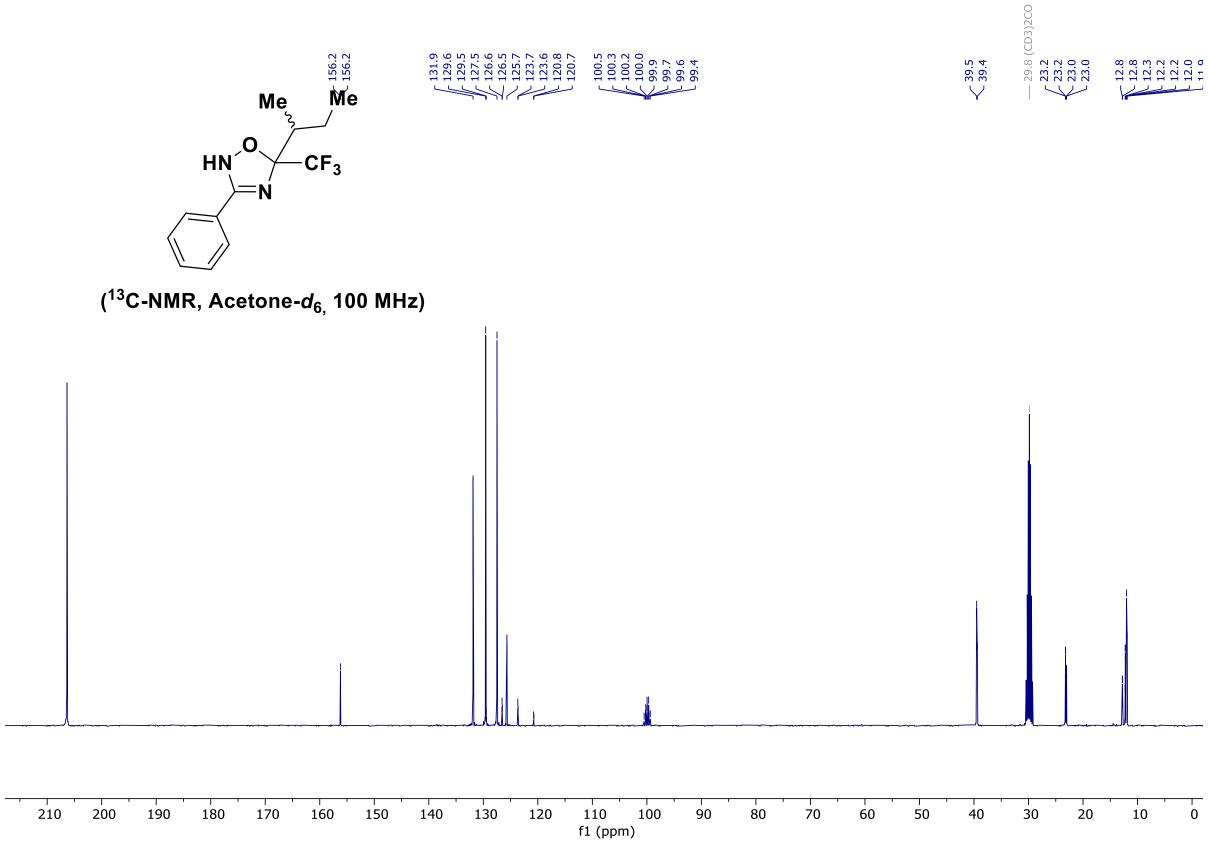

(^{13}C-NMR, Acetone-d_6, 100 MHz)

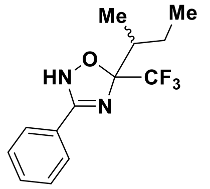

(^{19}F-NMR, Acetone-d_6, 377 MHz)

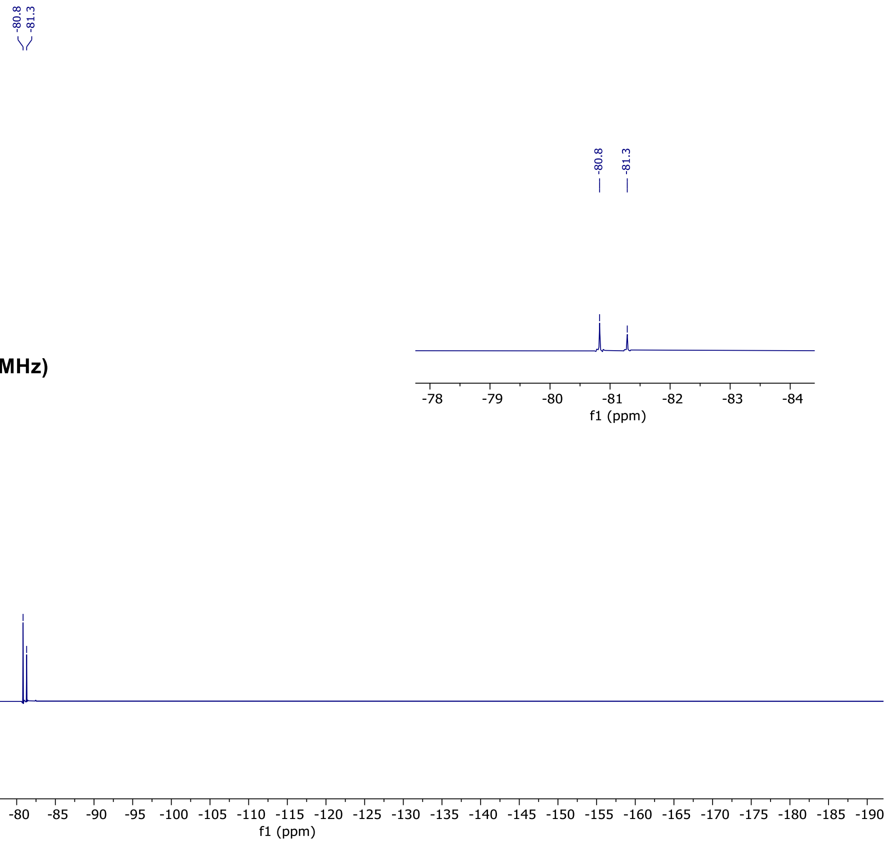

4-(5-hexyl-5-(trifluoromethyl)-2,5-dihydro-1,2,4-oxadiazol-3-yl)-*N*,*N*-dimethylaniline (36)

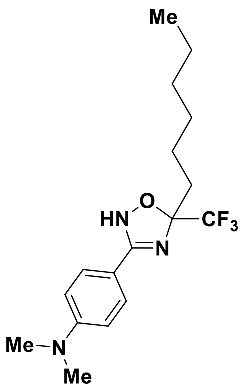

(^{1}H-NMR, Acetone-d_6, 600 MHz)

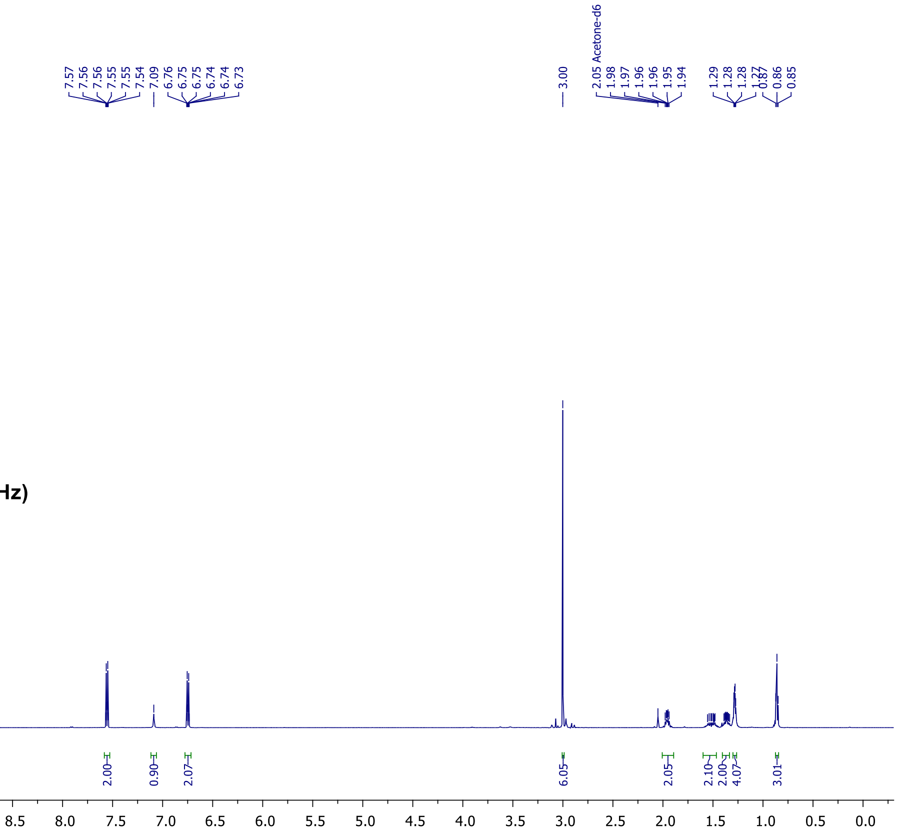

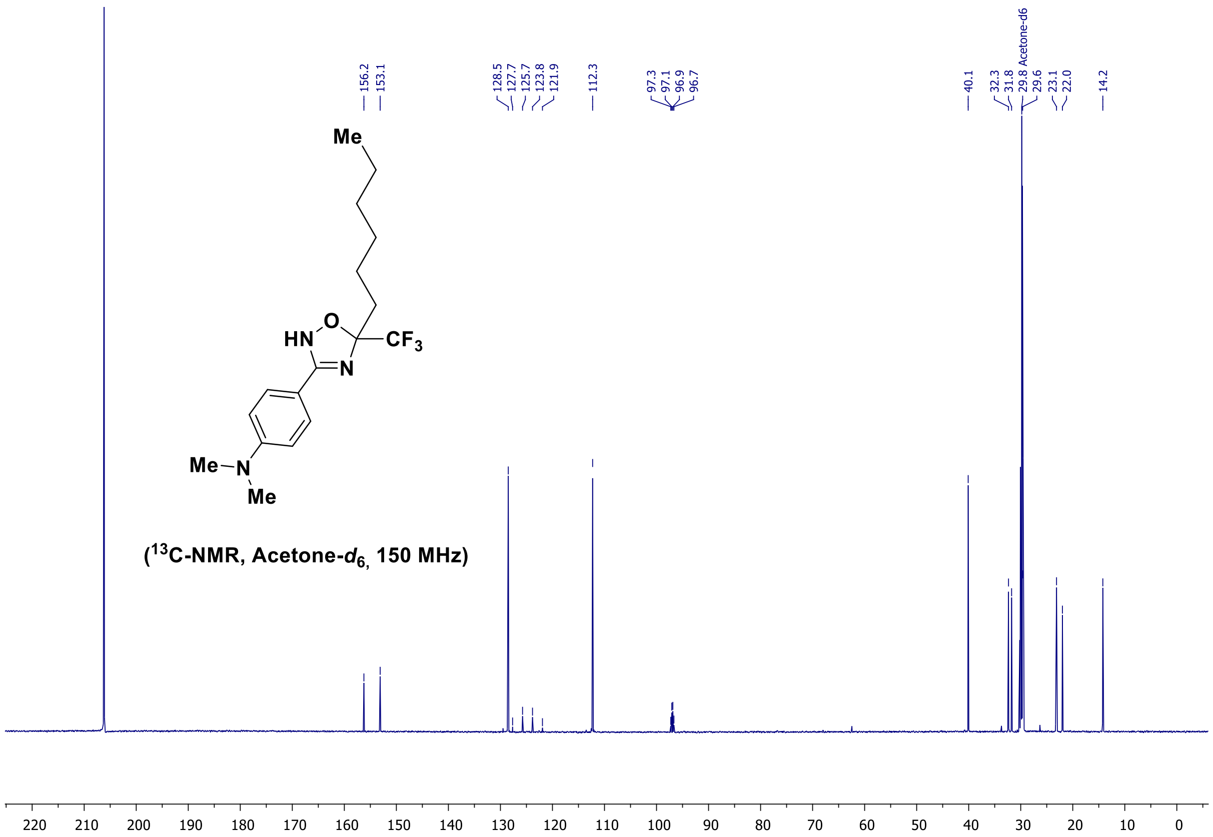

(^{13}C-NMR, Acetone-d_6, 150 MHz)

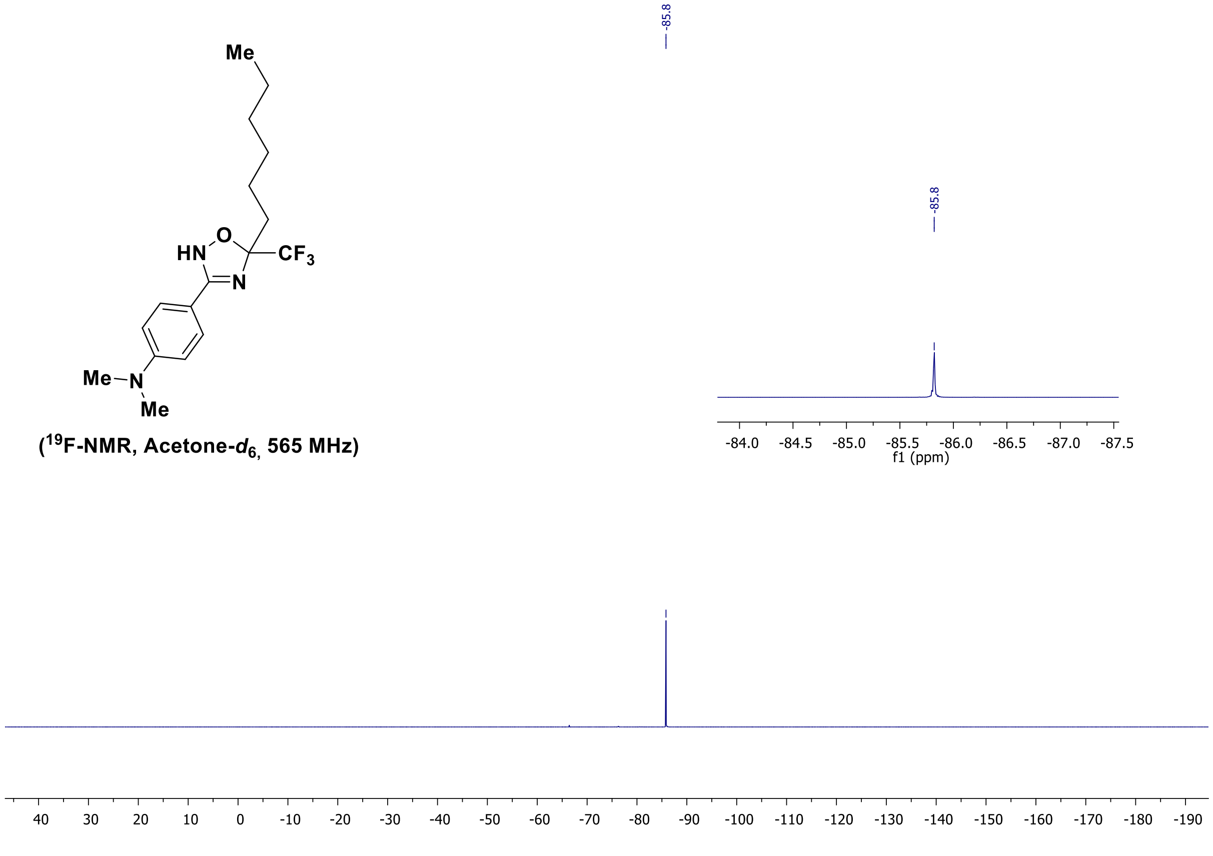

(^{19}F-NMR, Acetone-d_6, 565 MHz)

3,5-diphenyl-5-(trifluoromethyl)-2,5-dihydro-1,2,4-oxadiazole (37)

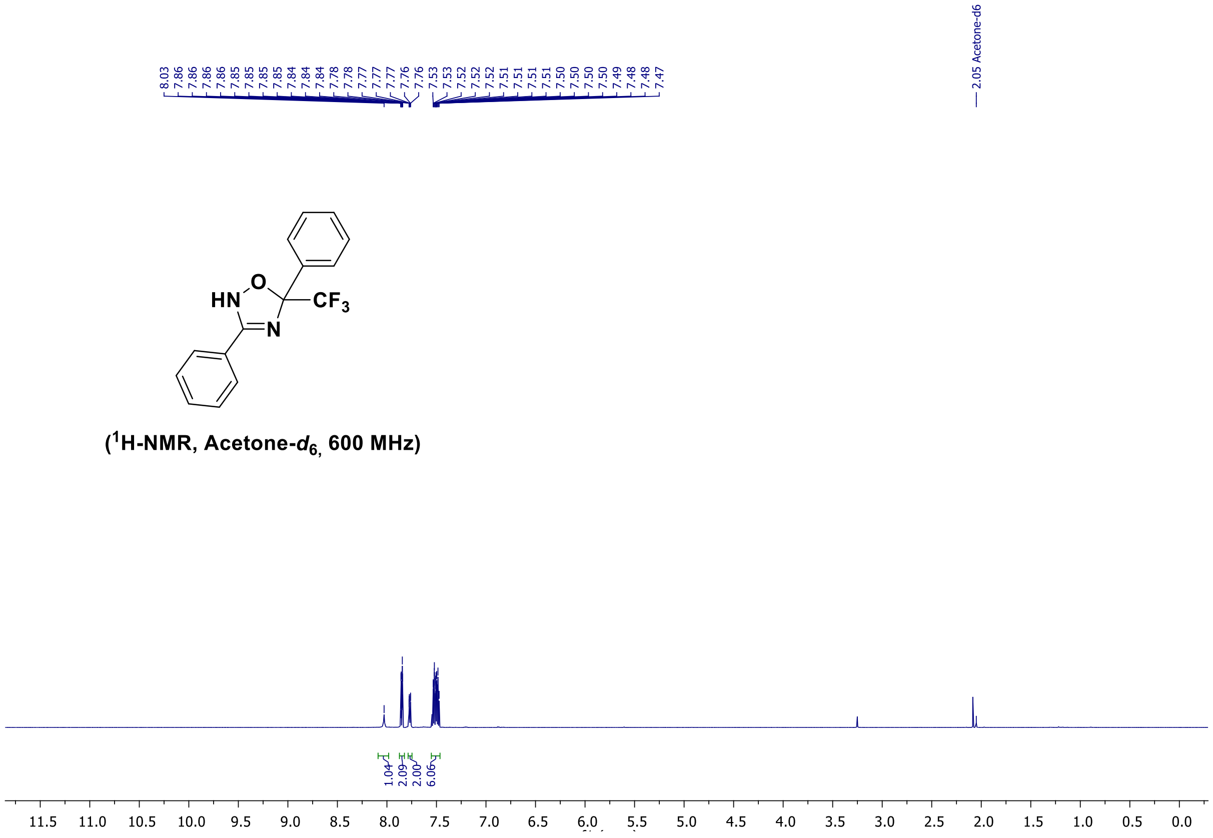

(^{1}H-NMR, Acetone-d_6, 600 MHz)

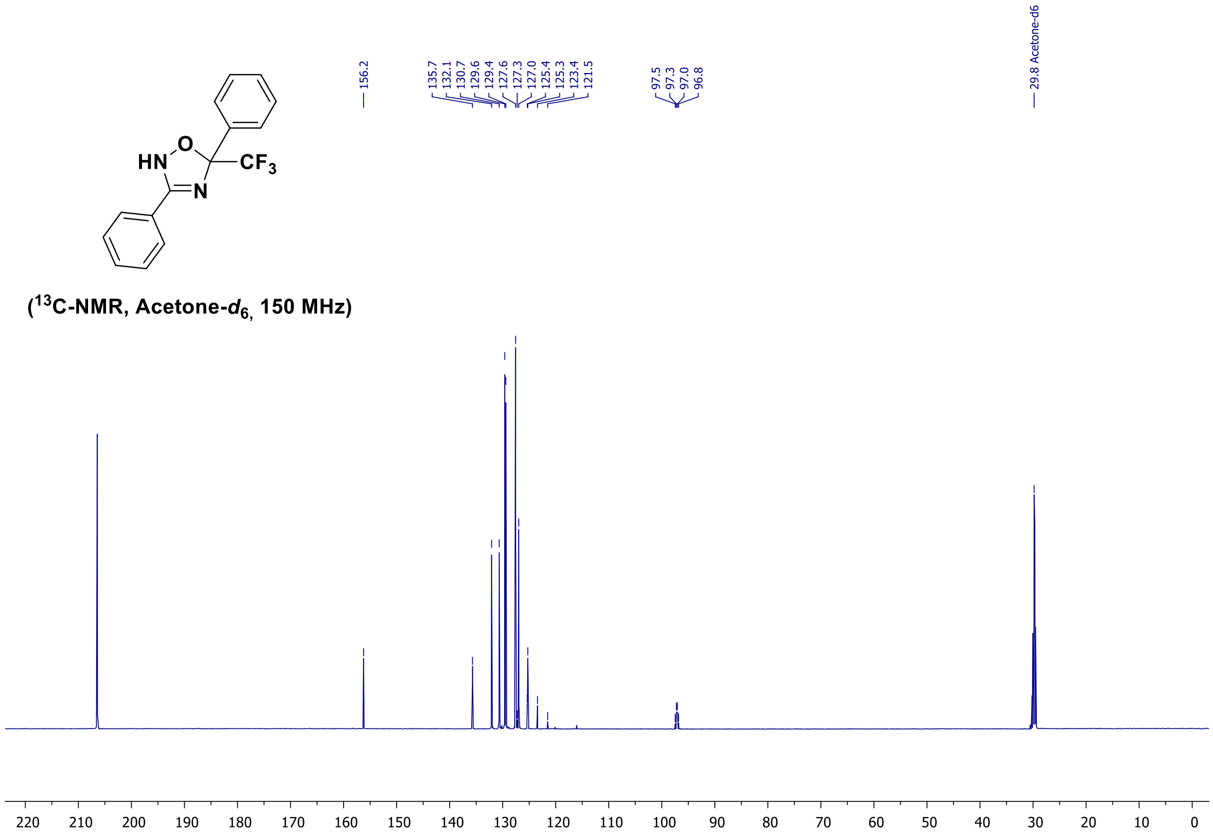

(^{13}C-NMR, Acetone-d_6, 150 MHz)

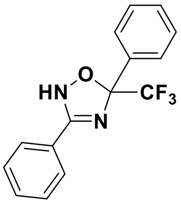

(^{19}F-NMR, Acetone-d_6, 565 MHz)

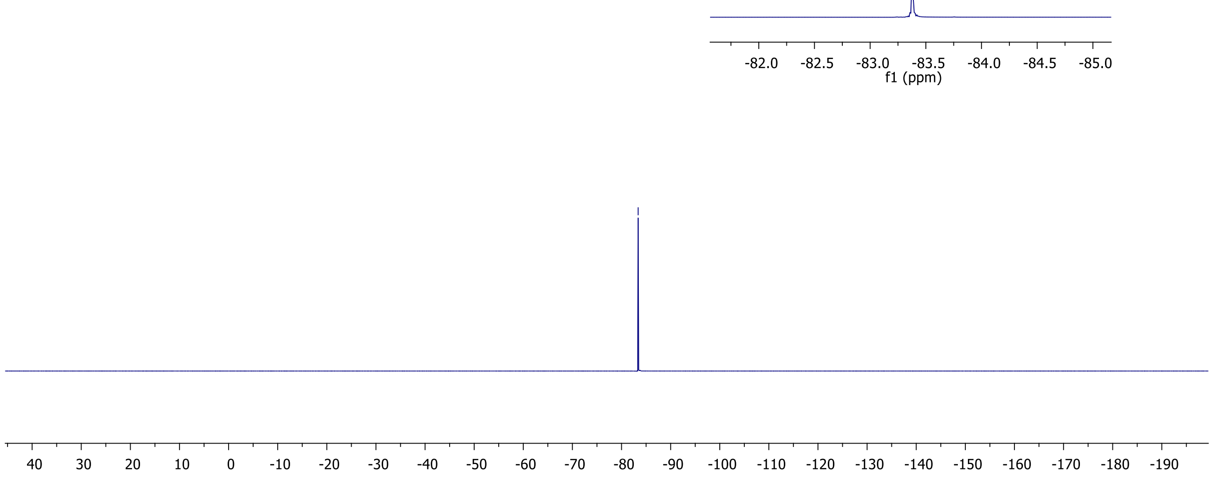

3-phenyl-5-(thiophen-2-yl)-5-(trifluoromethyl)-2,5-dihydro-1,2,4-oxadiazole (38)

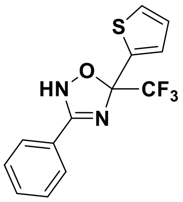

(^{1}H-NMR, Acetone-d_6, 400 MHz)

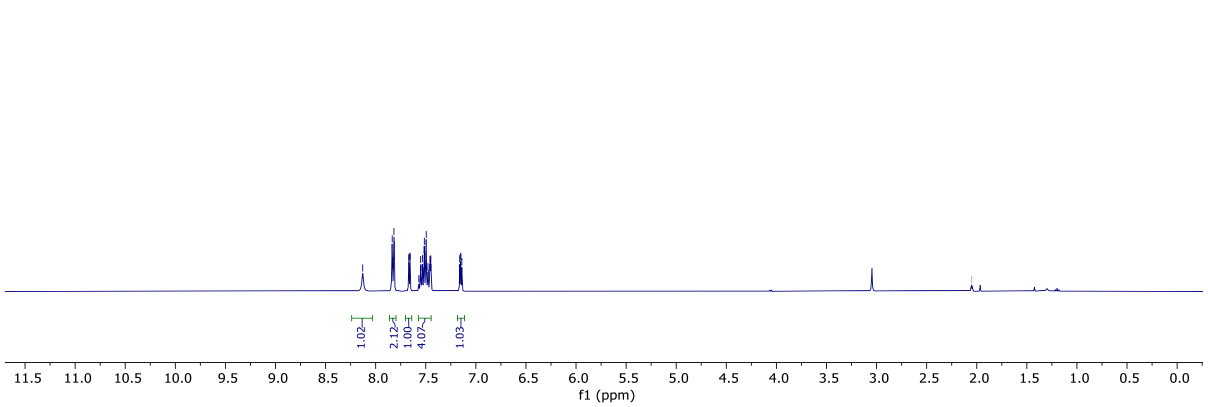

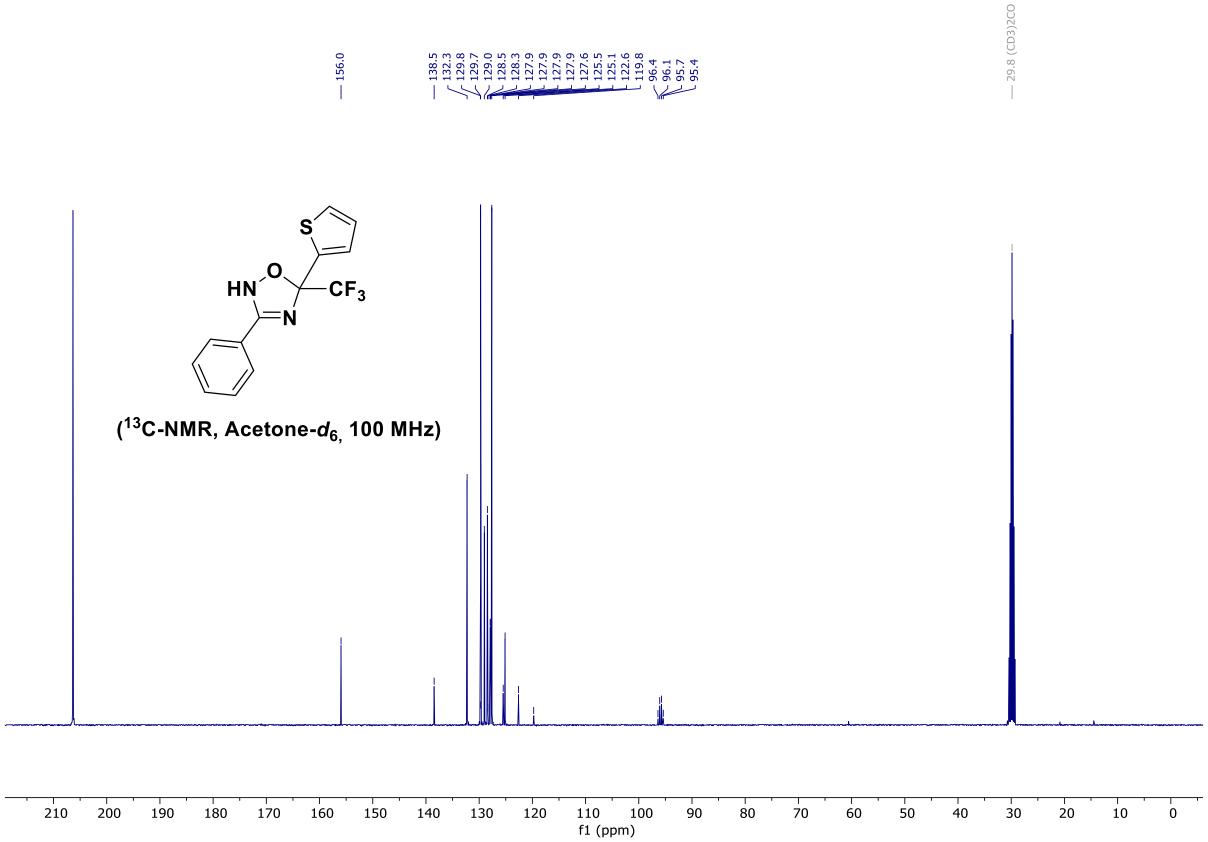
156.0
138.5
132.3
129.8
129.7
129.0
128.5
128.3
127.9
127.9
127.9
127.9
127.6
125.5
125.1
122.6
119.8
96.4
96.1
95.7
95.4
29.8 (CD3)2CO
S
HN
O
CF$_3$
N
(^{13}C-NMR, Acetone-d_6, 100 MHz)
210 200 190 180 170 160 150 140 130 120 110 100 90 80 70 60 50 40 30 20 10 0
f1 (ppm)

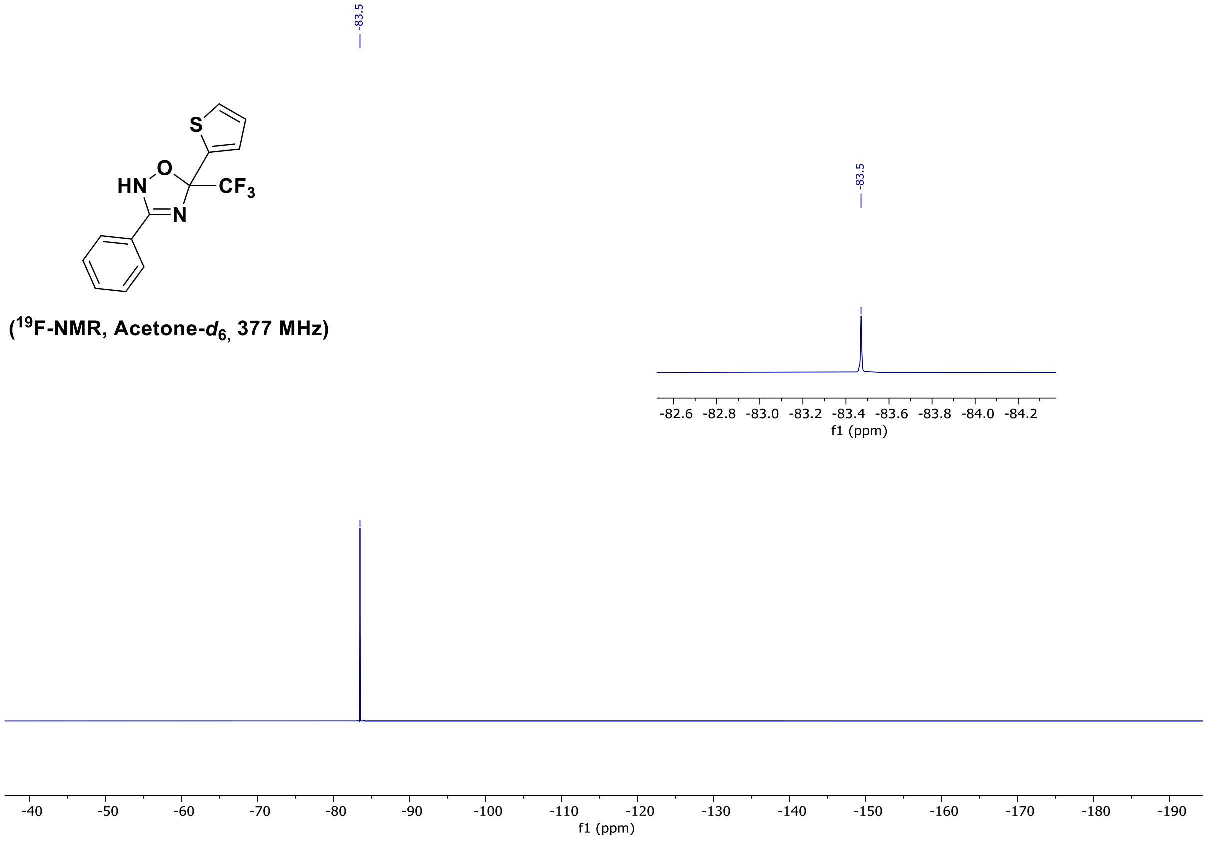
-83.5
S
HN
O
CF$_3$
N
(^{19}F-NMR, Acetone-d_6, 377 MHz)
-83.5
-82.6 -82.8 -83.0 -83.2 -83.4 -83.6 -83.8 -84.0 -84.2
f1 (ppm)
-40 -50 -60 -70 -80 -90 -100 -110 -120 -130 -140 -150 -160 -170 -180 -190
f1 (ppm)

N,*N*-dimethyl-4-(5-methyl-5-(trifluoromethyl)-2,5-dihydro-1,2,4-oxadiazol-3-yl)aniline (39)

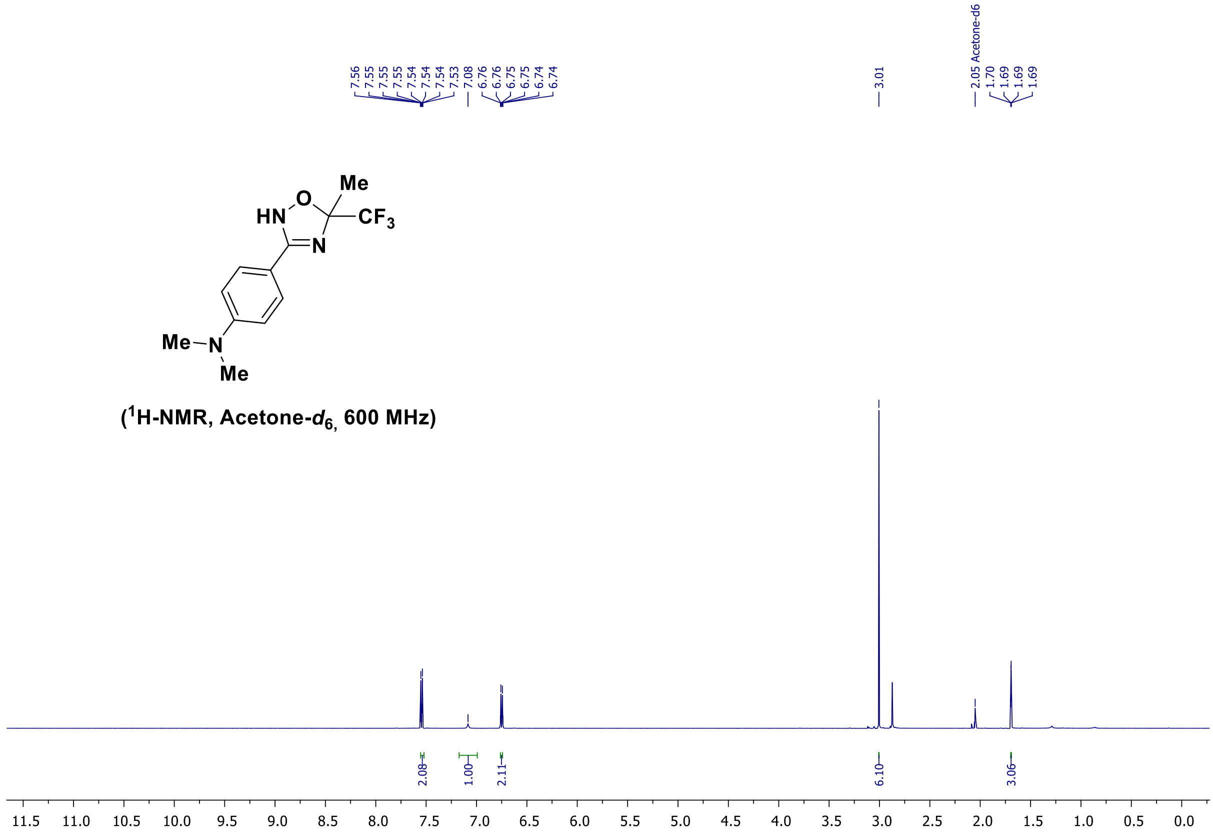

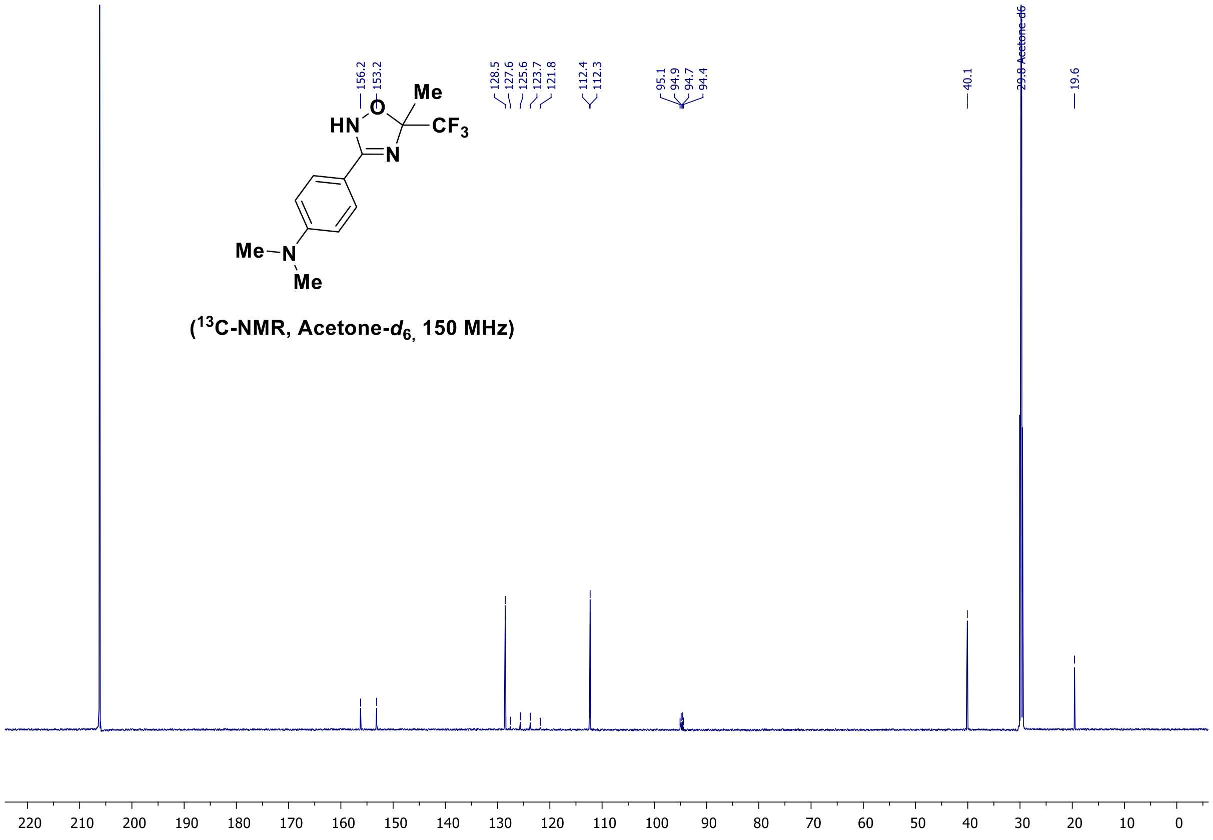

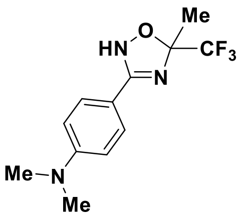

(^{19}F-NMR, Acetone-d_6, 565 MHz)

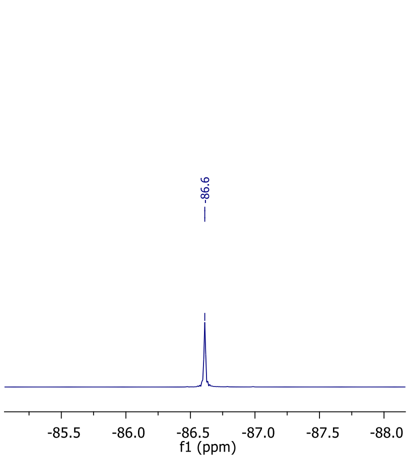
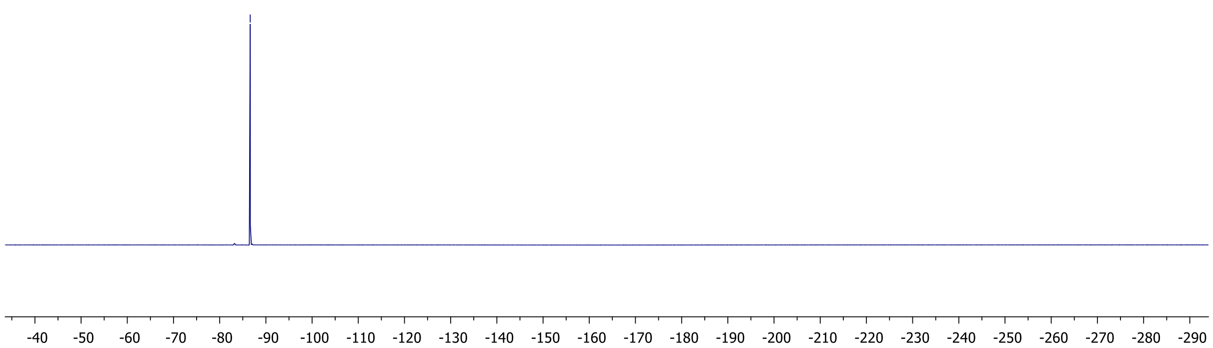

3-(4-methoxyphenyl)-5-ethyl-5-(trifluoromethyl)-2,5-dihydro-1,2,4-oxadiazole (40)

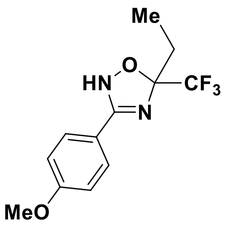

(^{1}H-NMR, Acetone-d_6, 600 MHz)

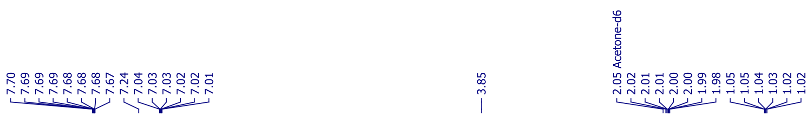
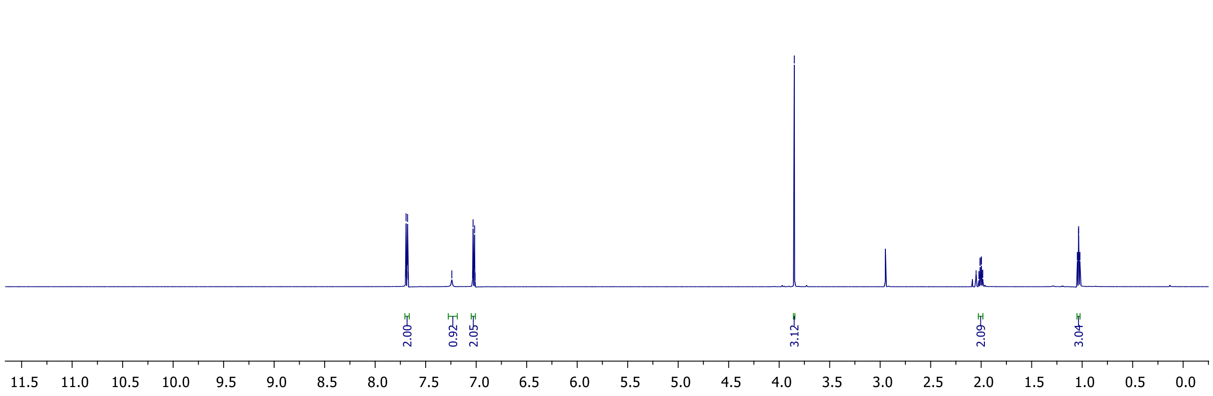

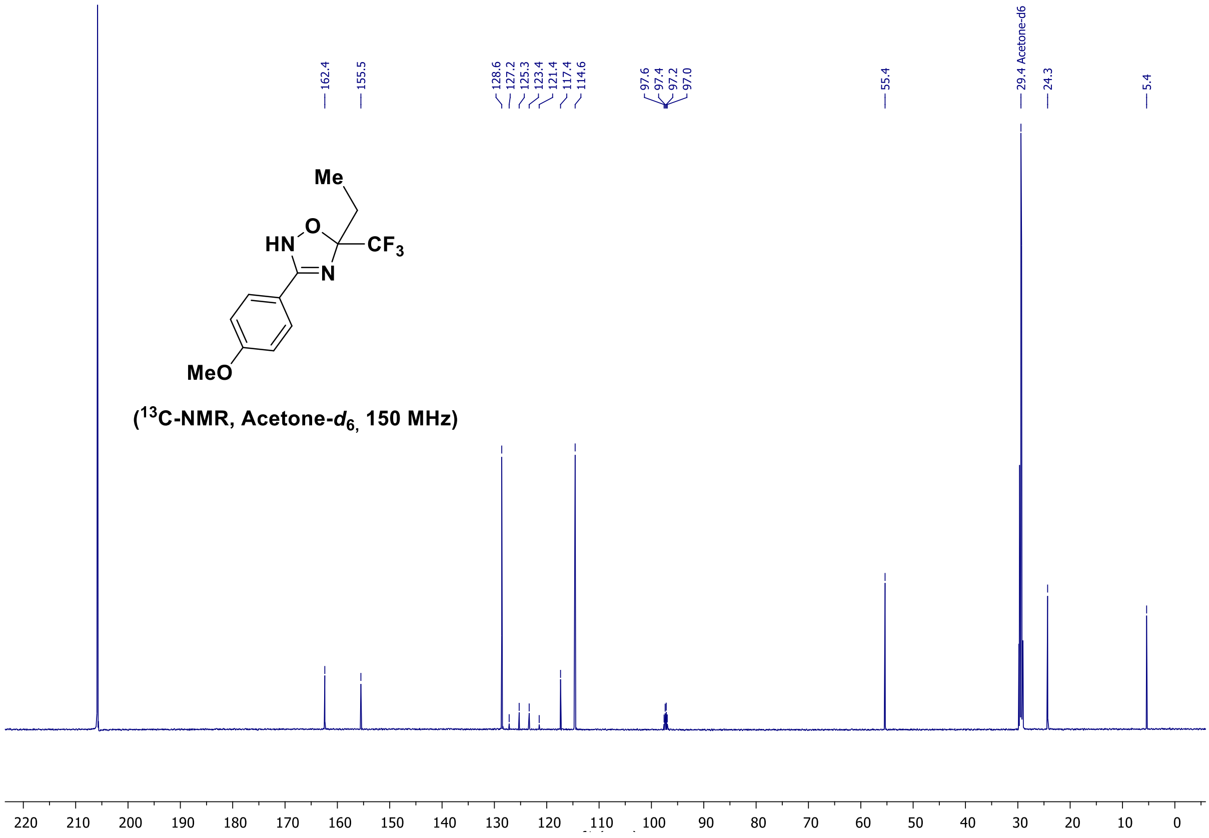

(^{13}C-NMR, Acetone-d_6, 150 MHz)

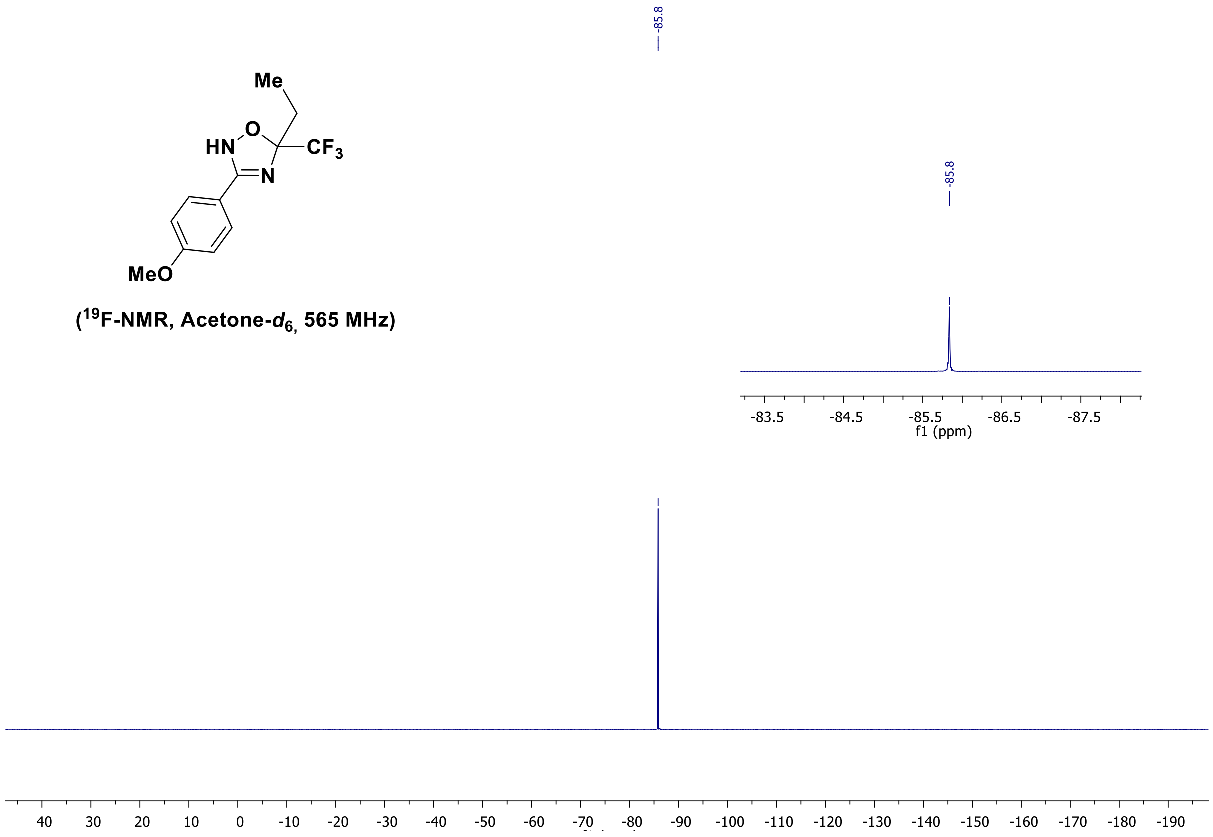

(^{19}F-NMR, Acetone-d_6, 565 MHz)

5-benzyl-3-(4-methoxyphenyl)-5-(trifluoromethyl)-2,5-dihydro-1,2,4-oxadiazole (41)

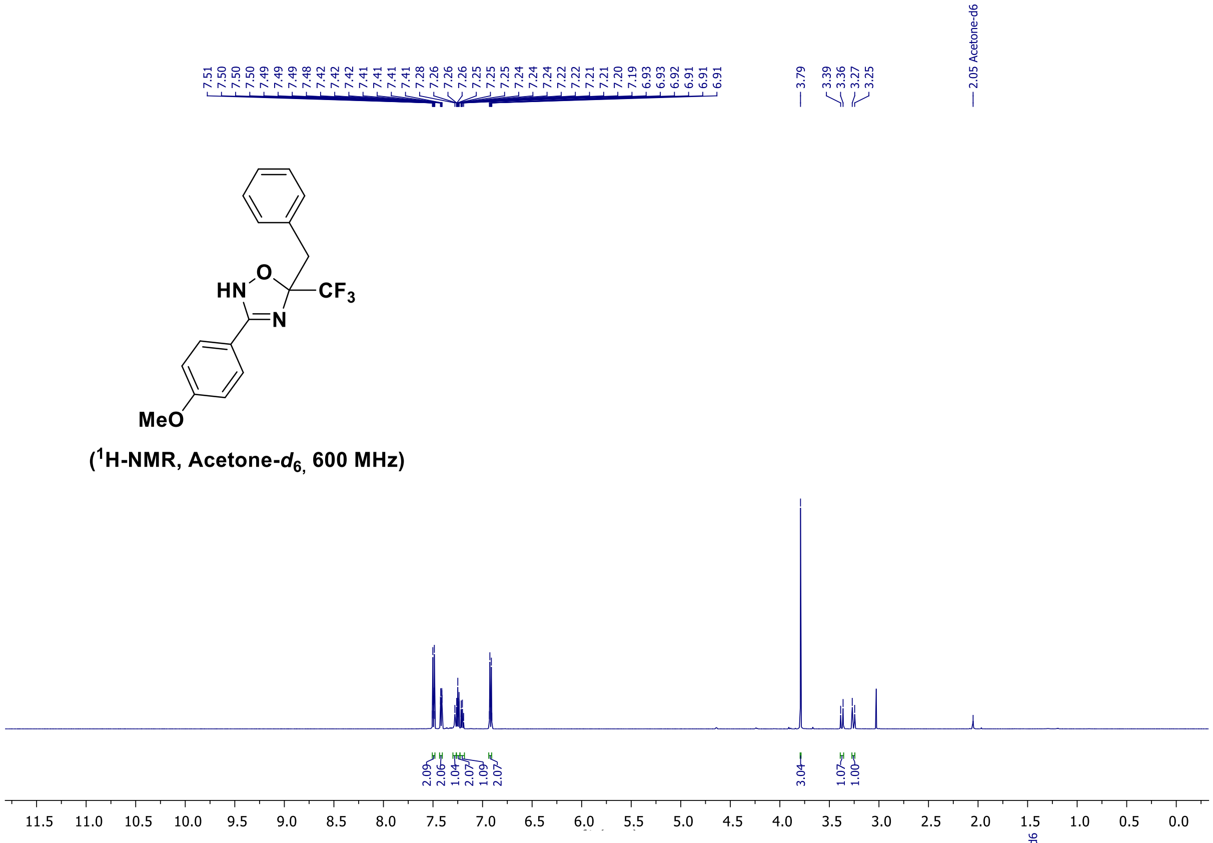

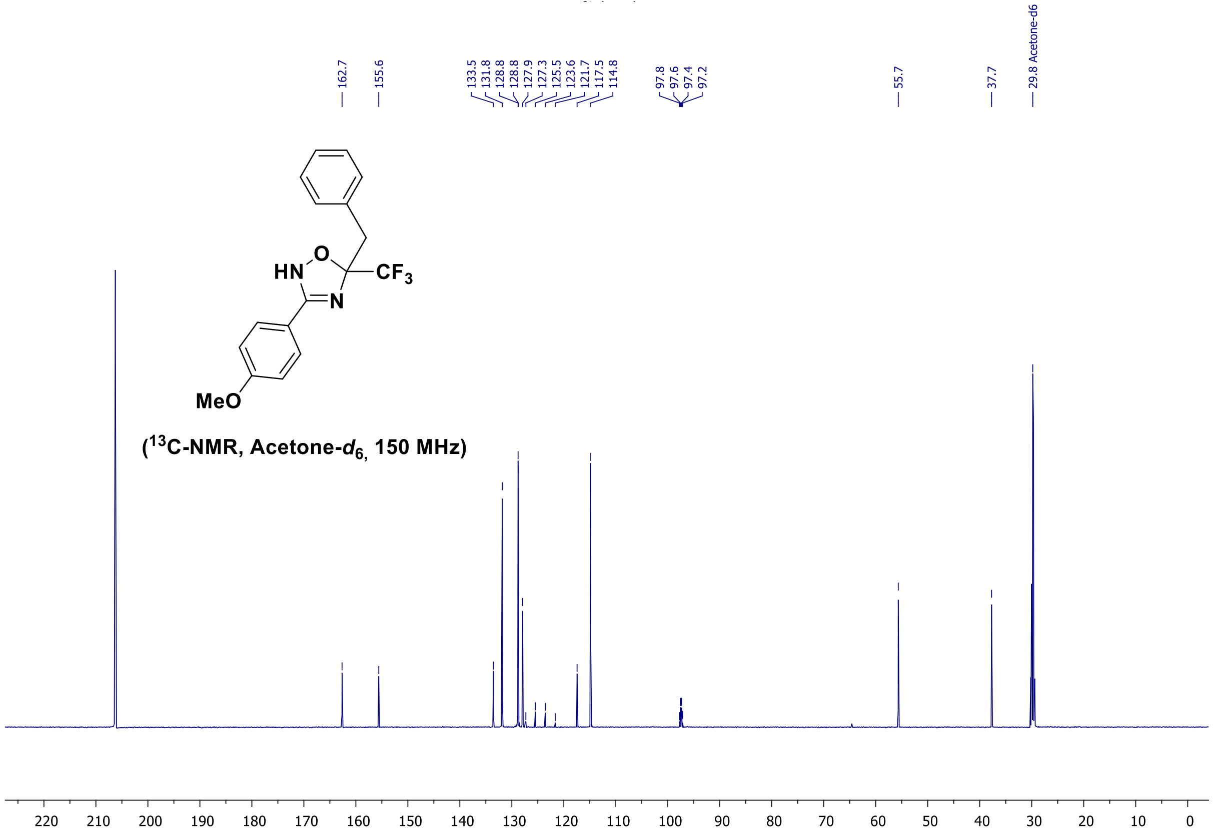

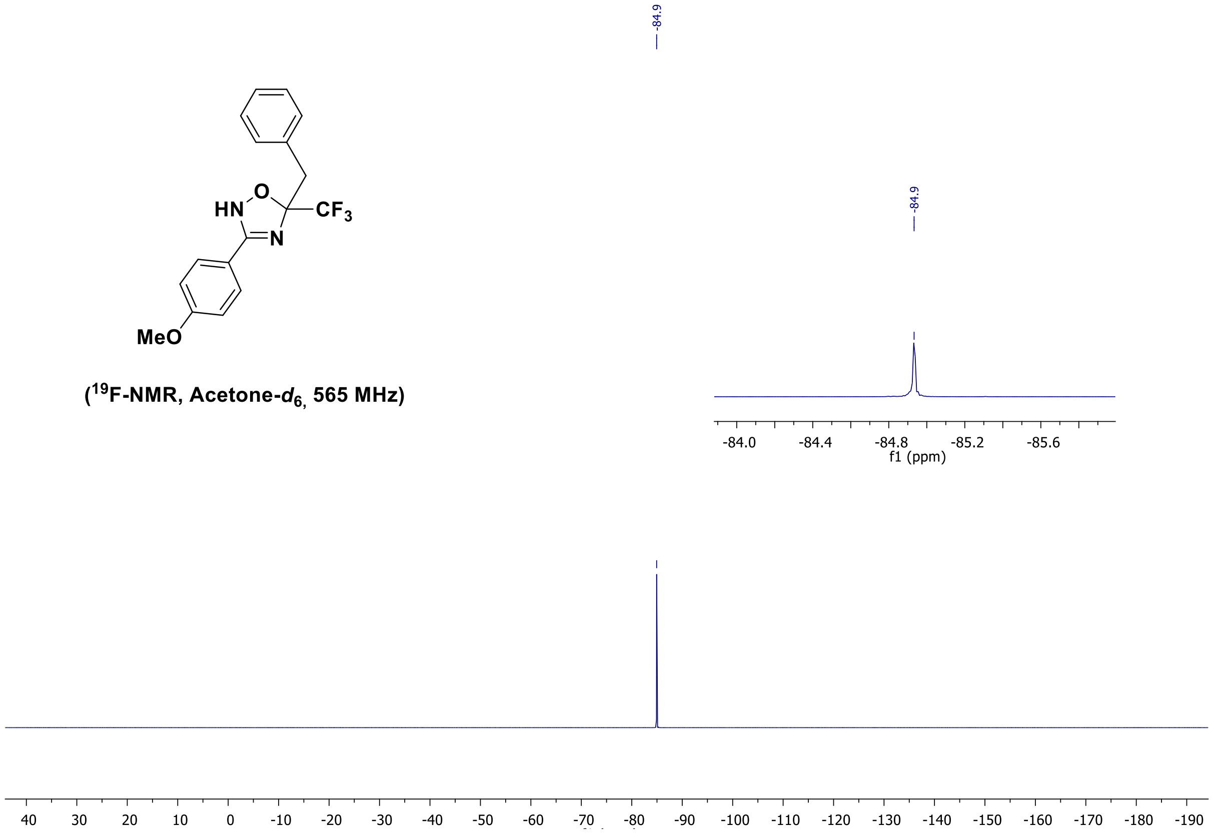

(^{19}F-NMR, Acetone-d_6, 565 MHz)

4-methyl-3-phenyl-5-(trifluoromethyl)-5-((trimethylsilyl)methyl)-4,5-dihydro-1,2,4-oxadiazole (42)

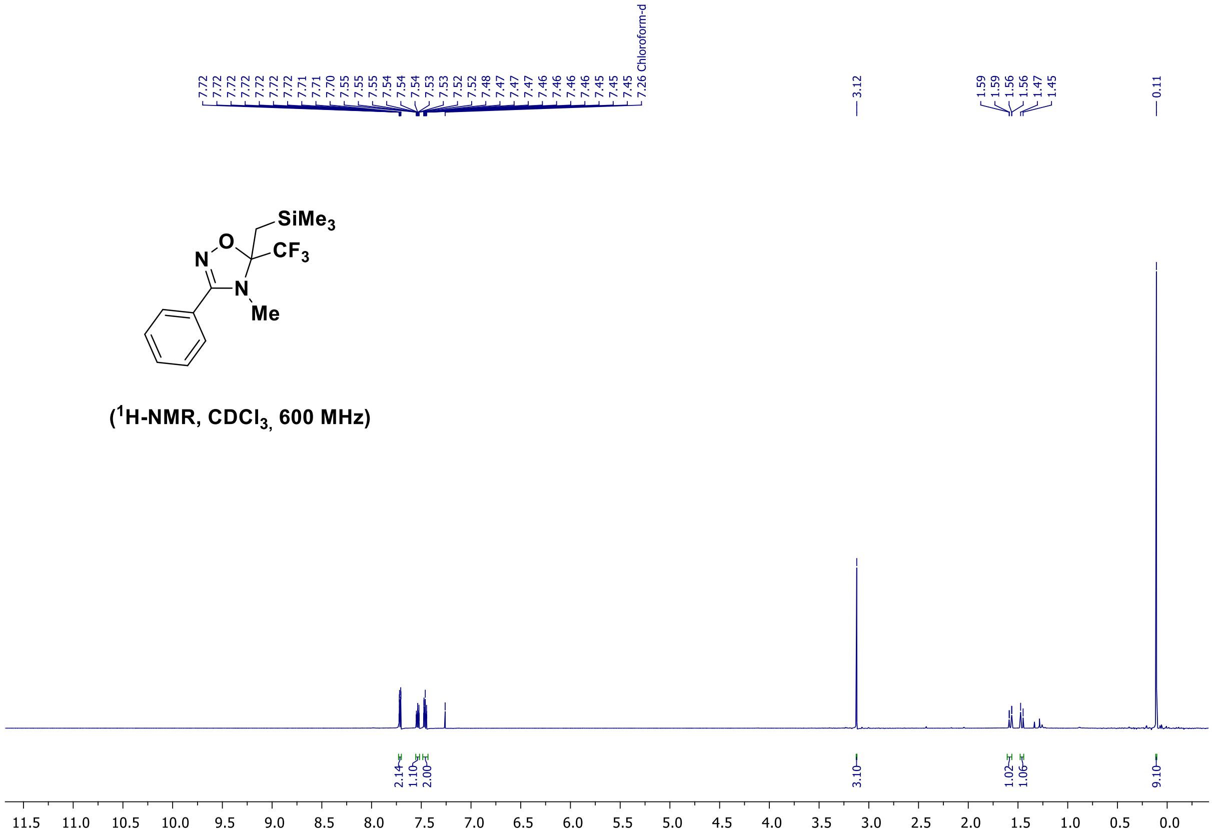

(^{1}H-NMR, $CDCl_3$, 600 MHz)

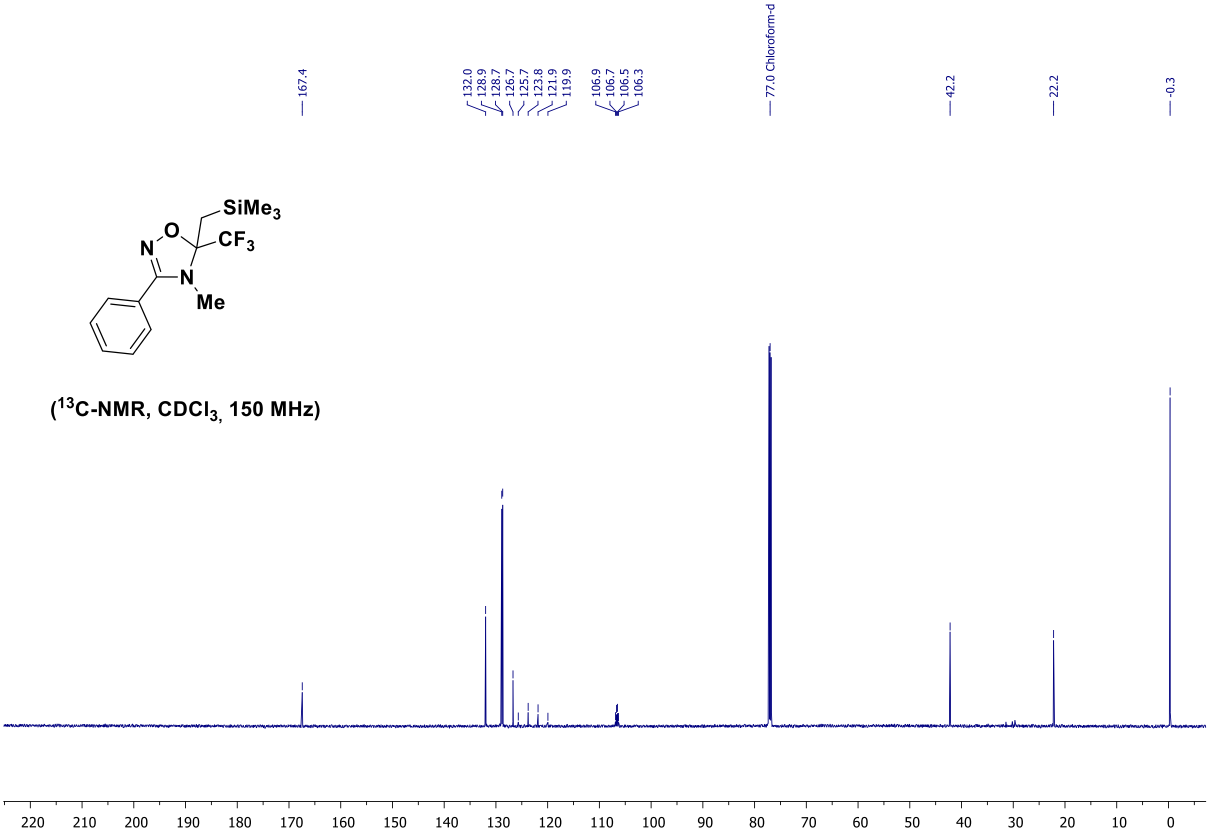

(^13C-NMR, CDCl$_3$, 150 MHz)

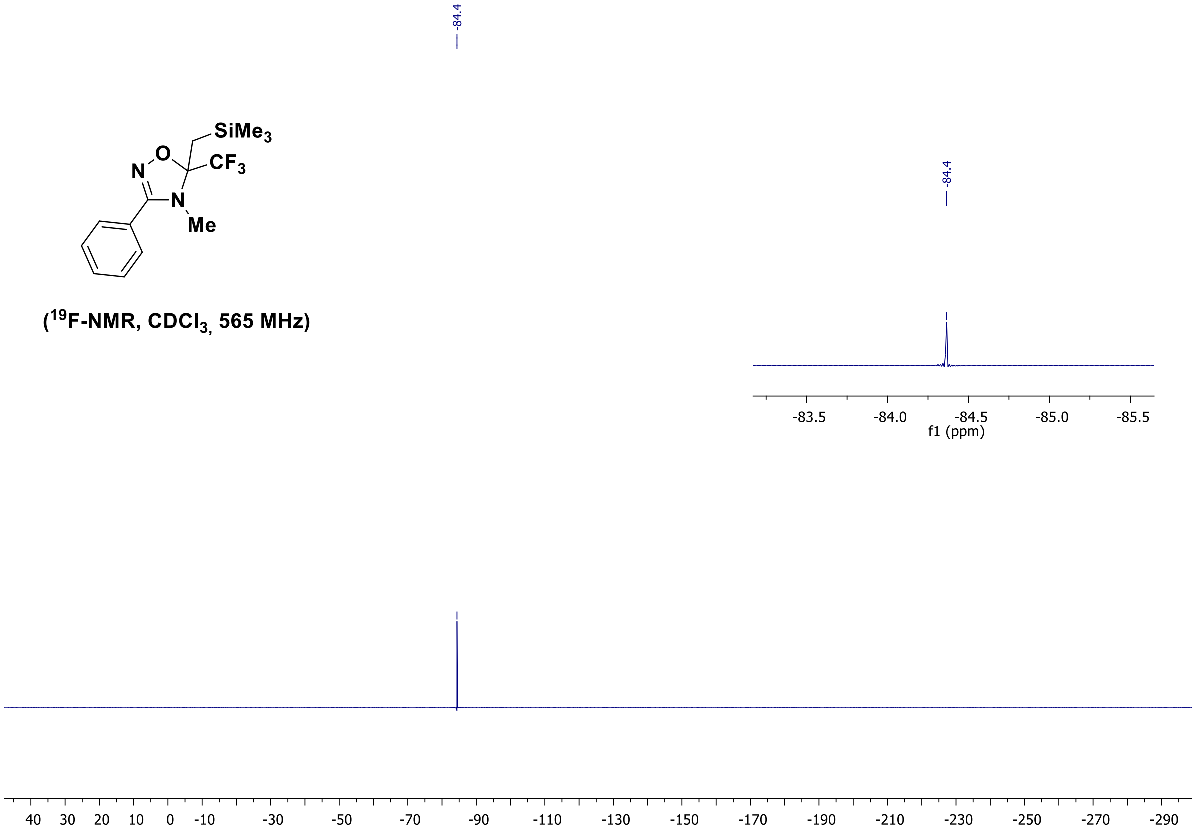

(^19F-NMR, CDCl$_3$, 565 MHz)

1-(5-butyl-3-phenyl-5-(trifluoromethyl)-1,2,4-oxadiazol-2(5H)-yl)ethan-1-one (43)

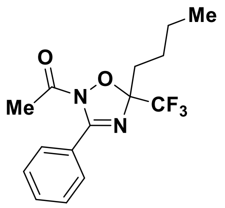

(^{1}H-NMR, $CDCl_3$, 400 MHz)

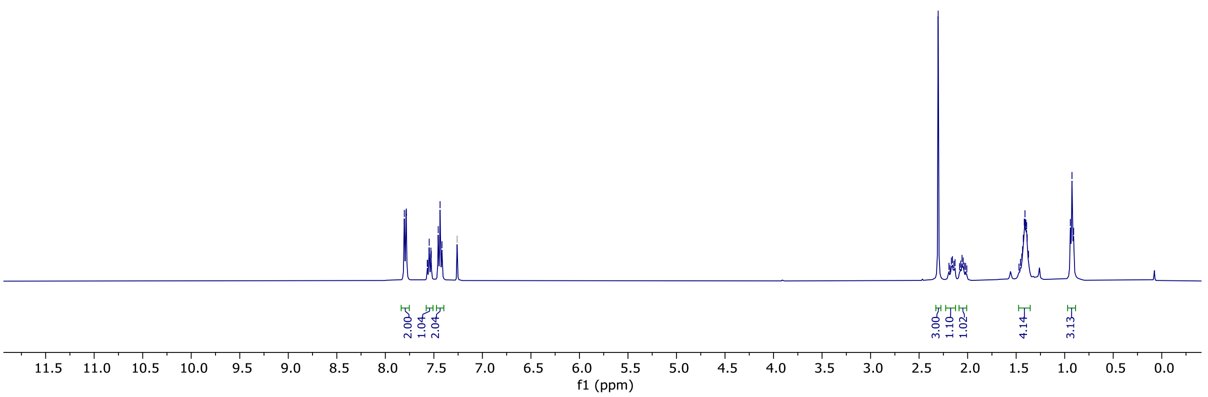

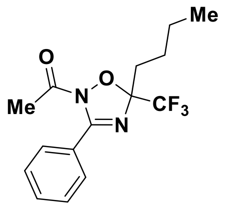

(^{13}C-NMR, $CDCl_3$, 100 MHz)

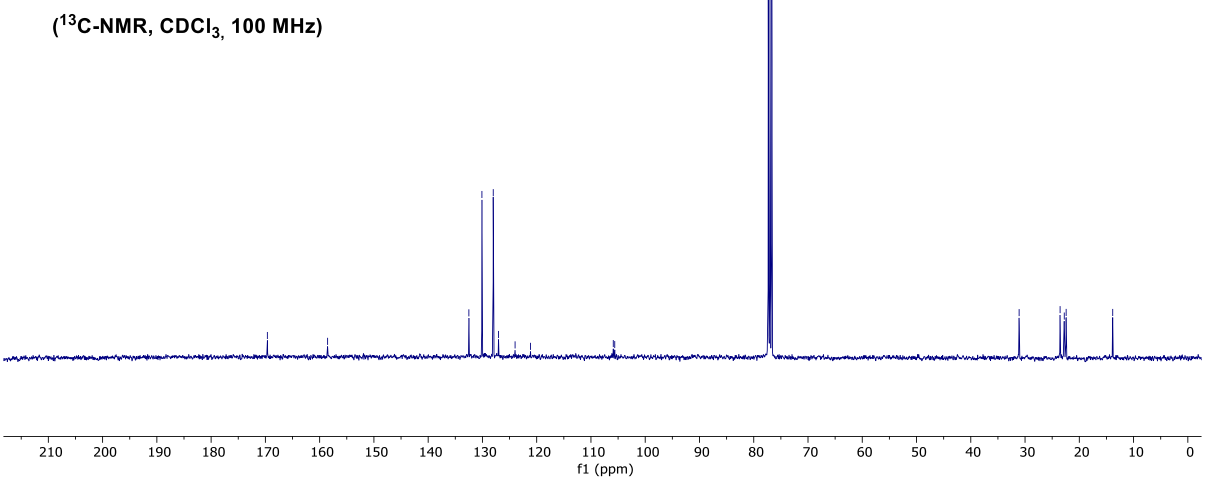

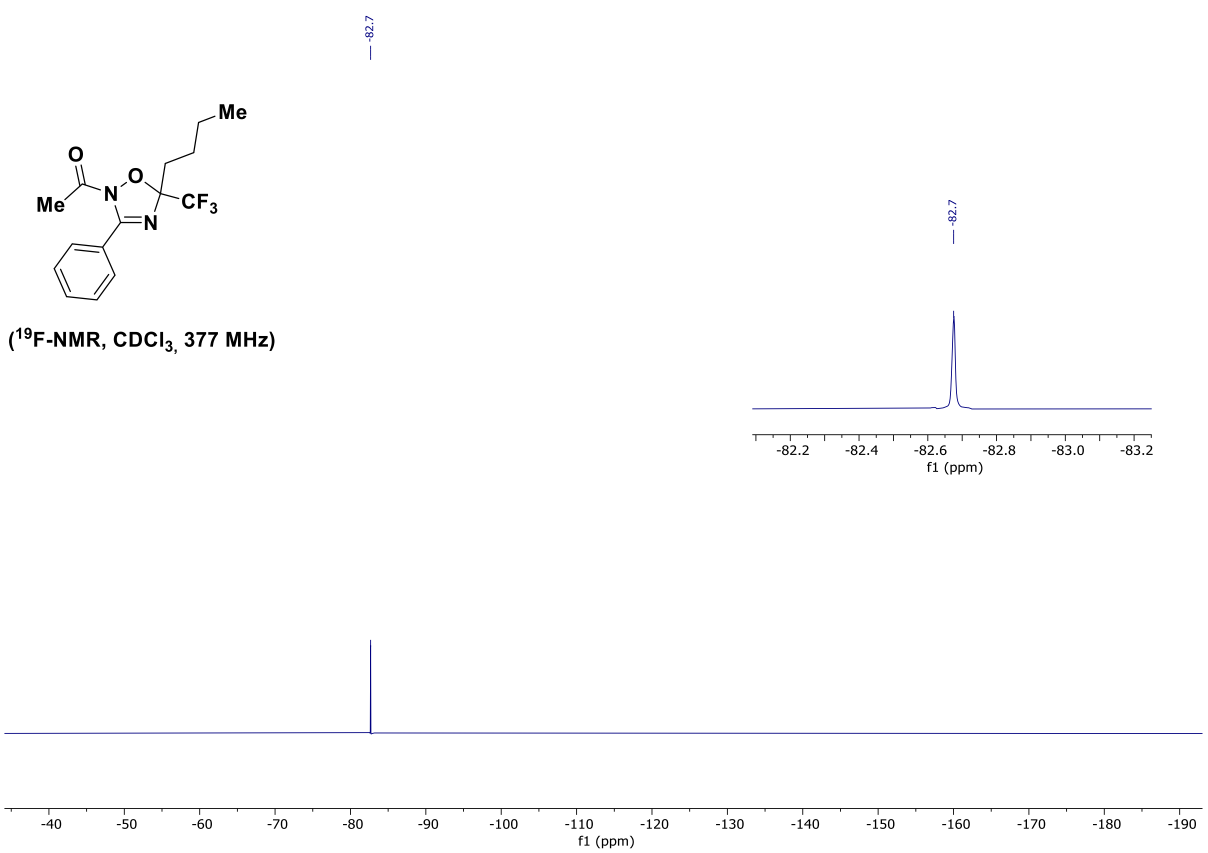

(^{19}F-NMR, $CDCl_3$, 377 MHz)

5-(chloromethyl)-3-phenyl-5-(trifluoromethyl)-2,5-dihydro-1,2,4-oxadiazole (44)

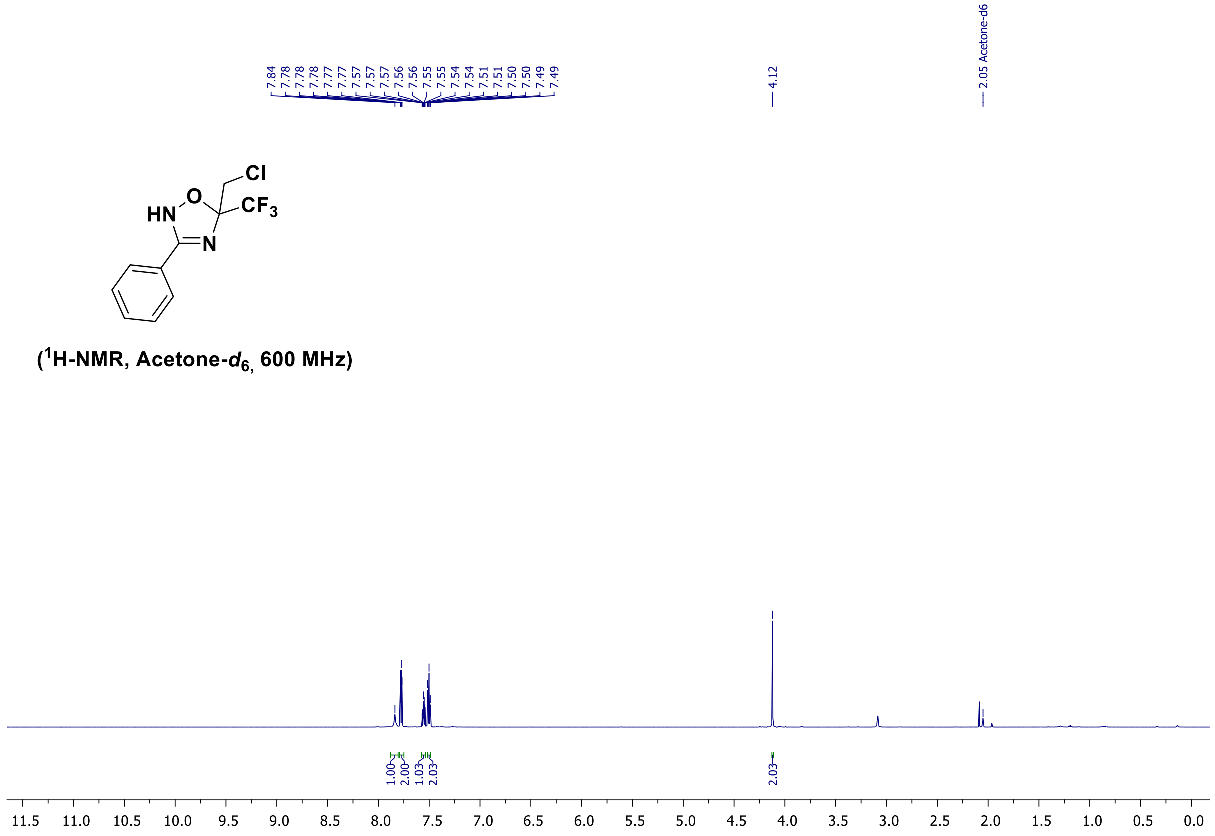

(^{1}H-NMR, Acetone-d_6, 600 MHz)

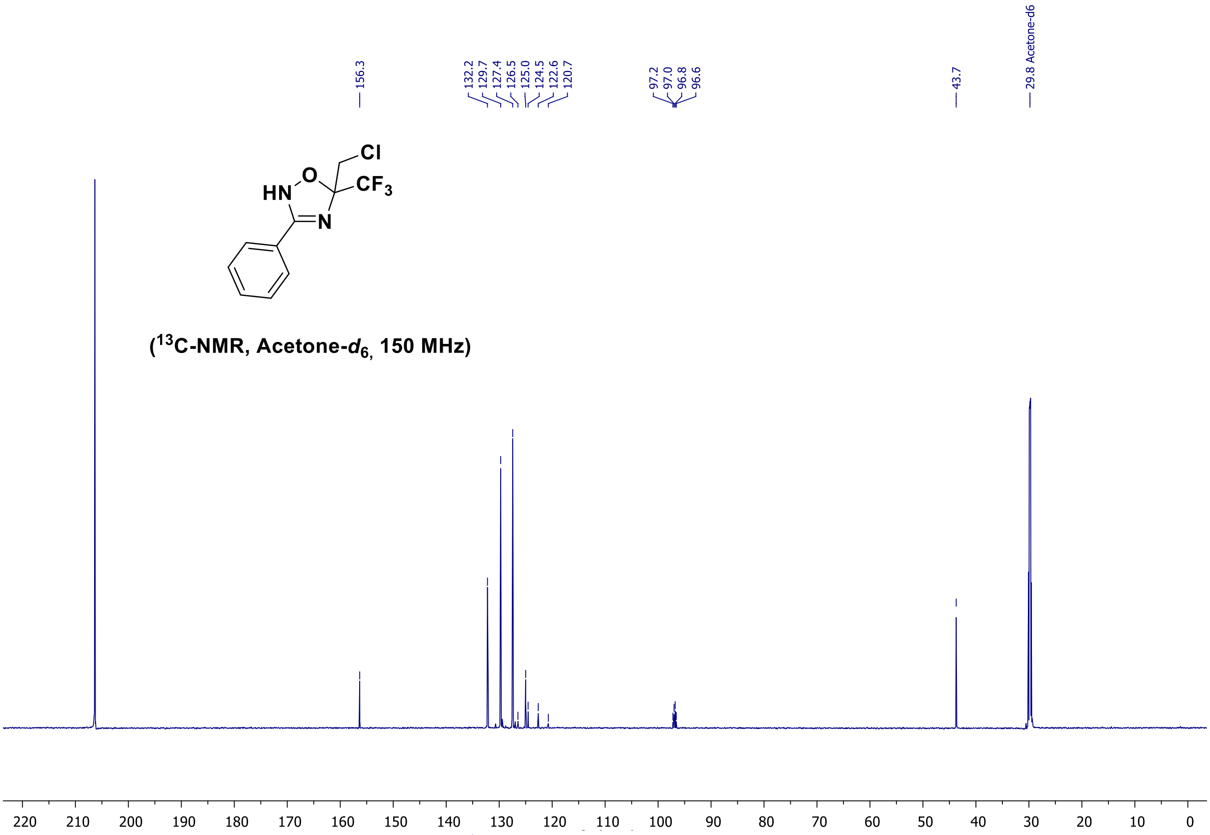

156.3
132.2
129.7
127.4
126.5
125.0
124.5
122.6
120.7
97.2
97.0
96.8
96.6
43.7
29.8 Acetone-d6
Cl
HN
O
CF3
N
(13C-NMR, Acetone-d6, 150 MHz)
220 210 200 190 180 170 160 150 140 130 120 110 100 90 80 70 60 50 40 30 20 10 0

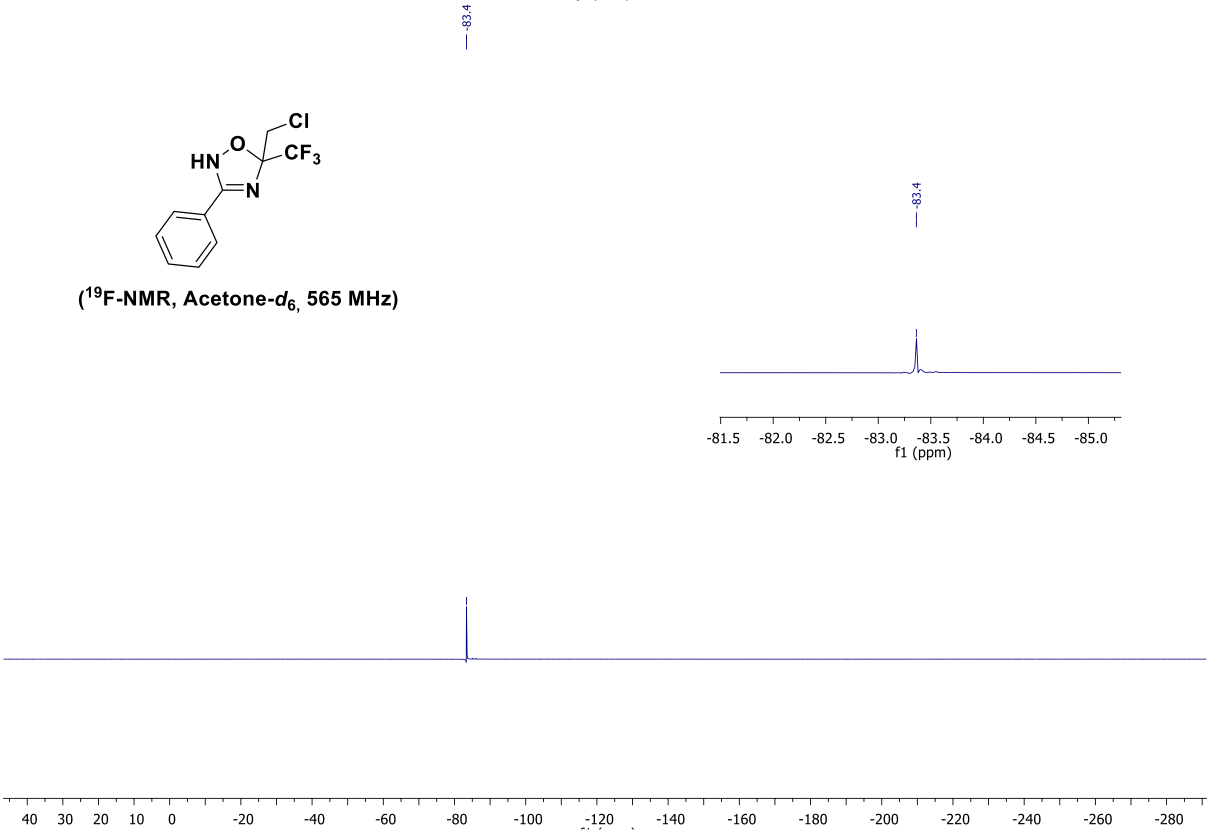

-83.4
Cl
HN
O
CF3
N
(19F-NMR, Acetone-d6, 565 MHz)
-83.4
-81.5 -82.0 -82.5 -83.0 -83.5 -84.0 -84.5 -85.0
f1 (ppm)
40 30 20 10 0 -20 -40 -60 -80 -100 -120 -140 -160 -180 -200 -220 -240 -260 -280

5-(chloromethyl)-3-(thiophen-2-yl)-5-(trifluoromethyl)-2,5-dihydro-1,2,4-oxadiazole (45)

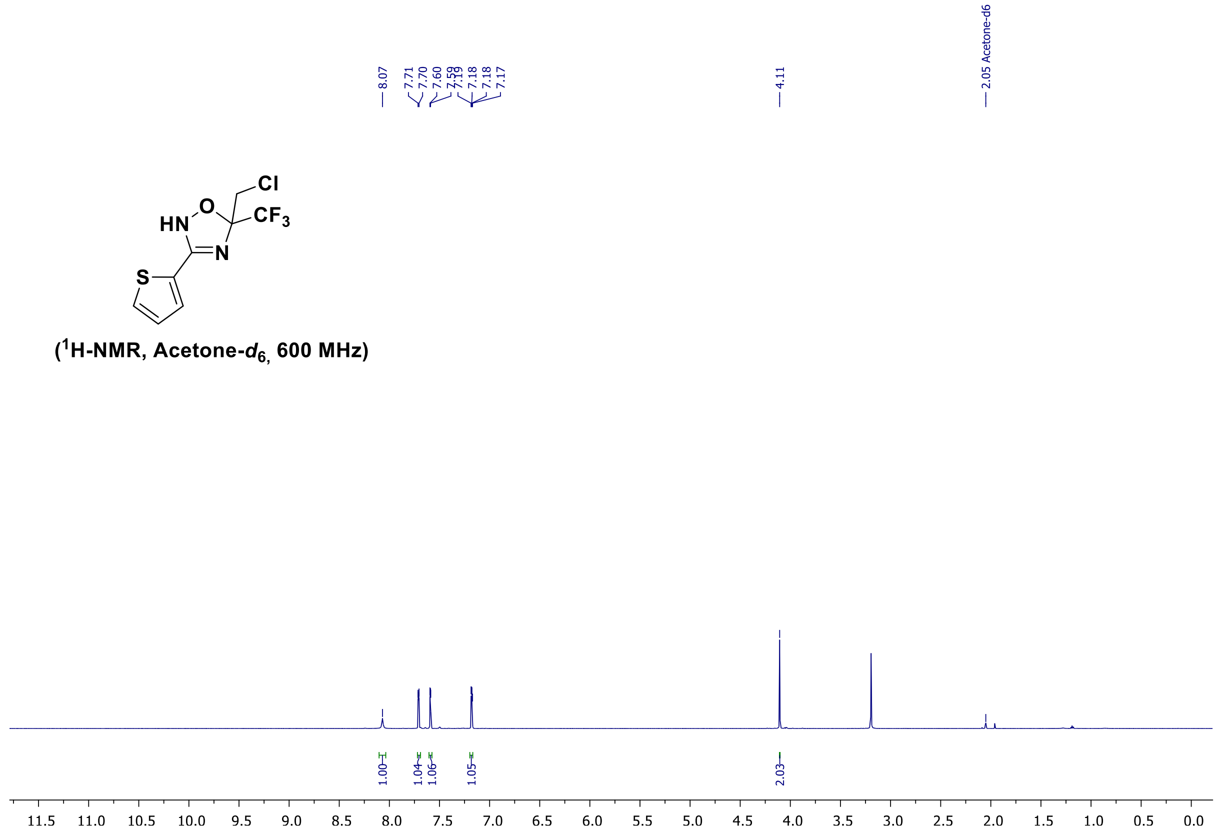

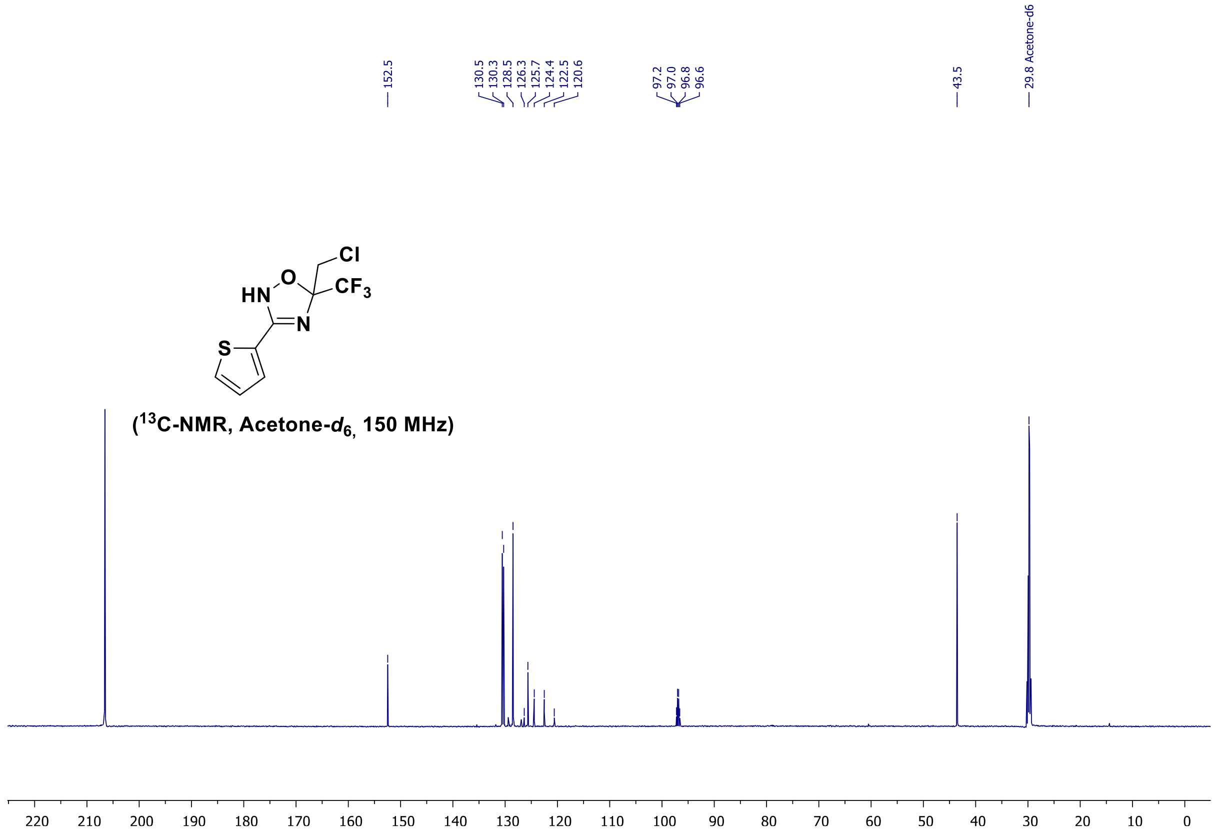

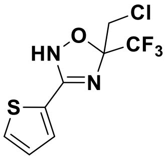

(^{19}F-NMR, Acetone-d_6, 565 MHz)

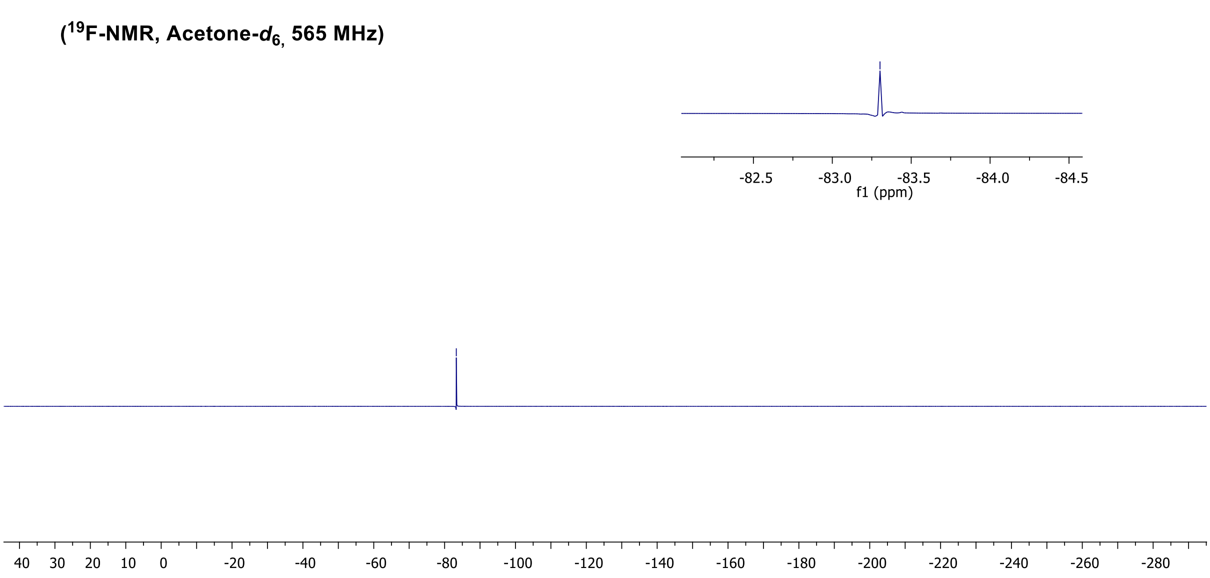

2-benzyl-5-(chloromethyl)-3-(4-chlorophenyl)-5-(trifluoromethyl)-2,5-dihydro-1,2,4-oxadiazole (46)

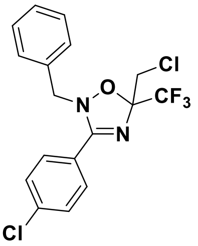

(^{1}H-NMR, CDCl$_3$, 600 MHz)

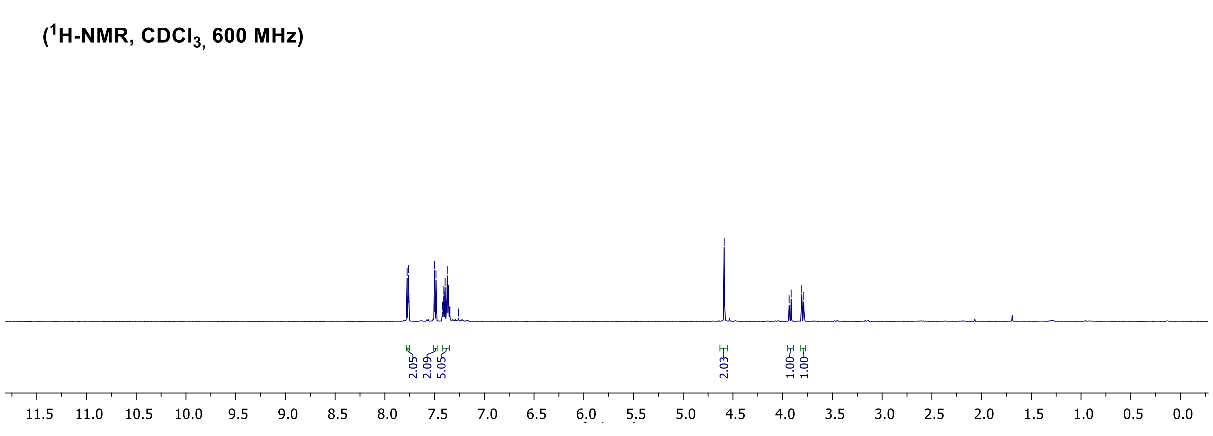

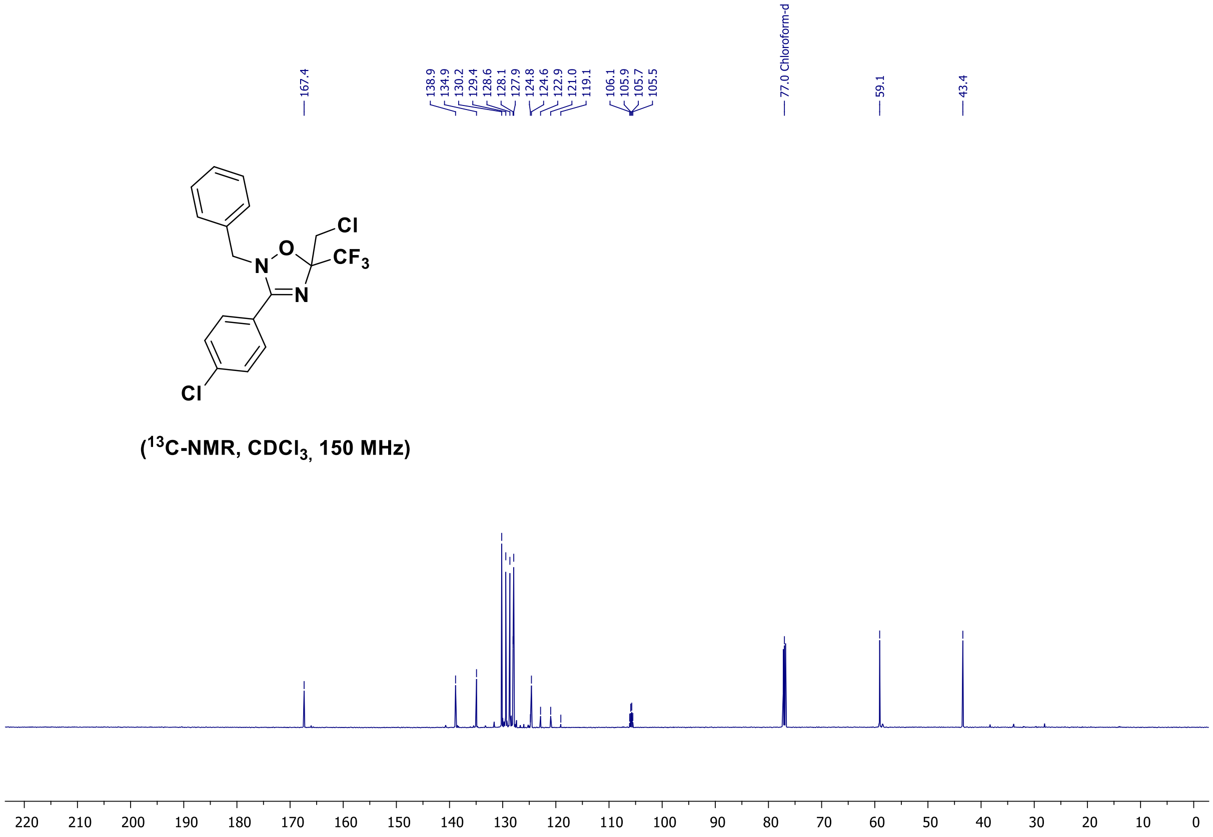
167.4
138.9
134.9
130.2
129.4
128.6
128.1
127.9
124.8
124.6
122.9
121.0
119.1
106.1
105.9
105.7
105.5
77.0 Chloroform-d
59.1
43.4
Cl
O
N
CF3
N
Cl
(13C-NMR, CDCl3, 150 MHz)
220 210 200 190 180 170 160 150 140 130 120 110 100 90 80 70 60 50 40 30 20 10 0

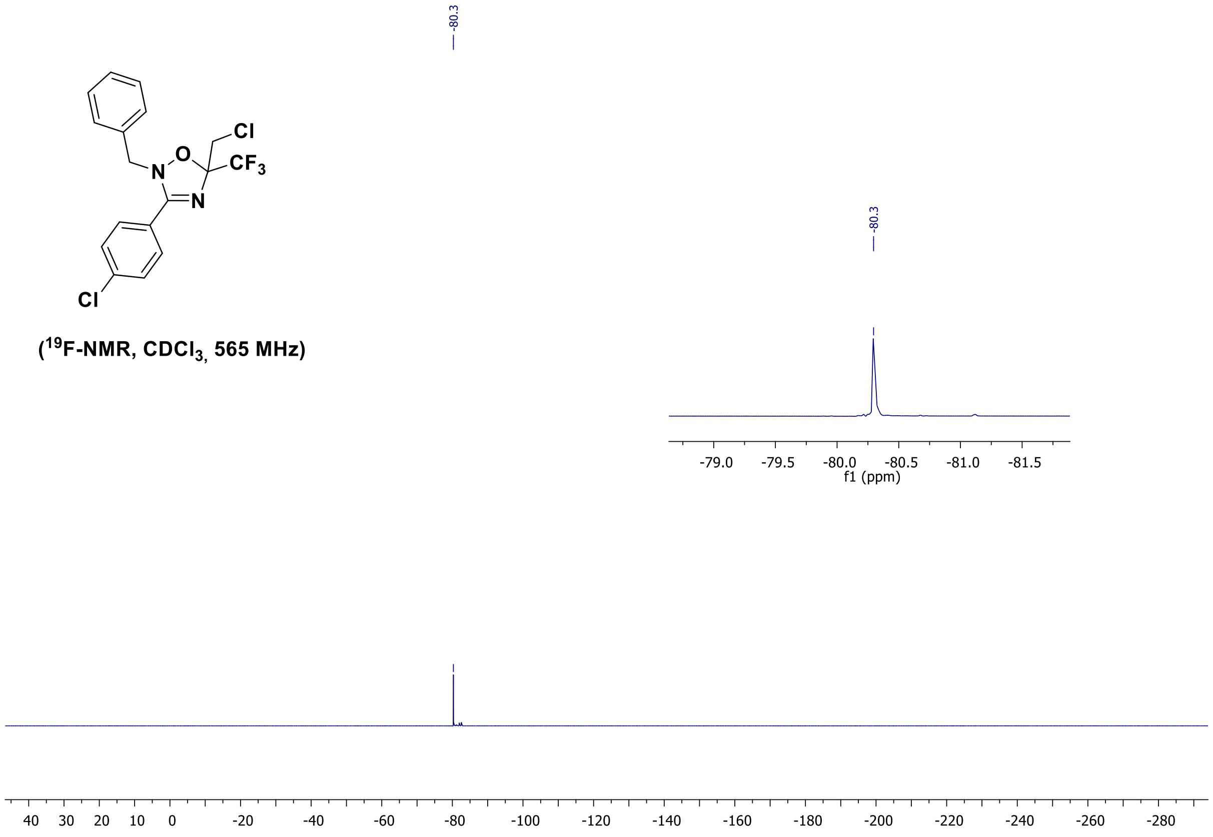
-80.3
Cl
O
N
CF3
N
Cl
(19F-NMR, CDCl3, 565 MHz)
-80.3
-79.0 -79.5 -80.0 -80.5 -81.0 -81.5
f1 (ppm)
40 30 20 10 0 -20 -40 -60 -80 -100 -120 -140 -160 -180 -200 -220 -240 -260 -280

2-allyl-5-(chloromethyl)-3-phenyl-5-(trifluoromethyl)-2,5-dihydro-1,2,4-oxadiazole (47)

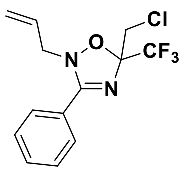

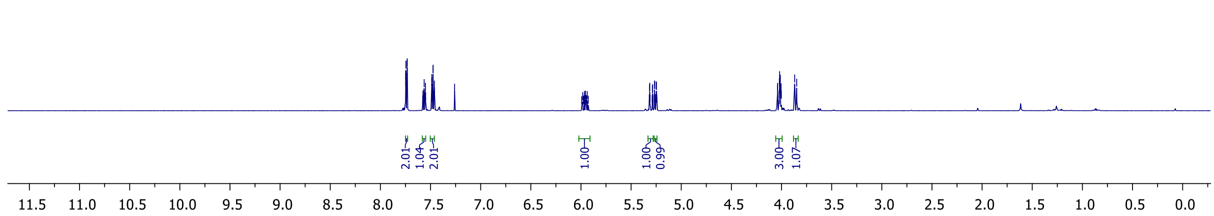

(¹H-NMR, $CDCl_3$, 600 MHz)

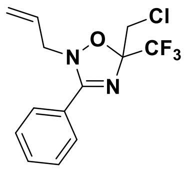

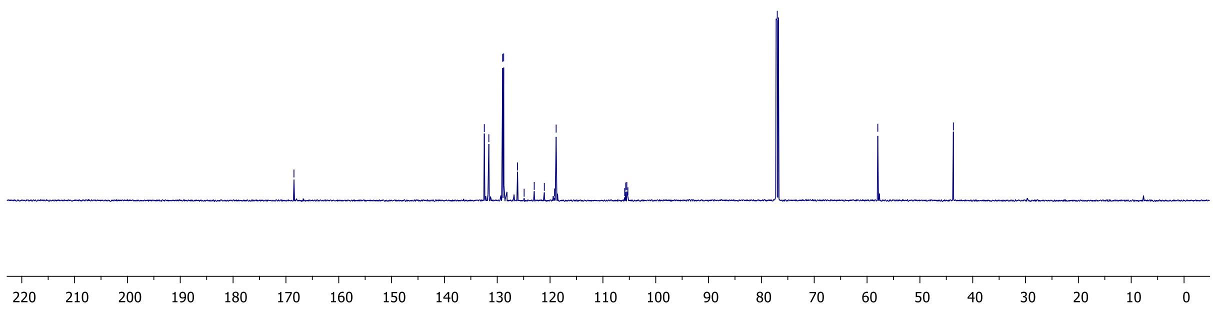

(¹³C-NMR, $CDCl_3$, 150 MHz)

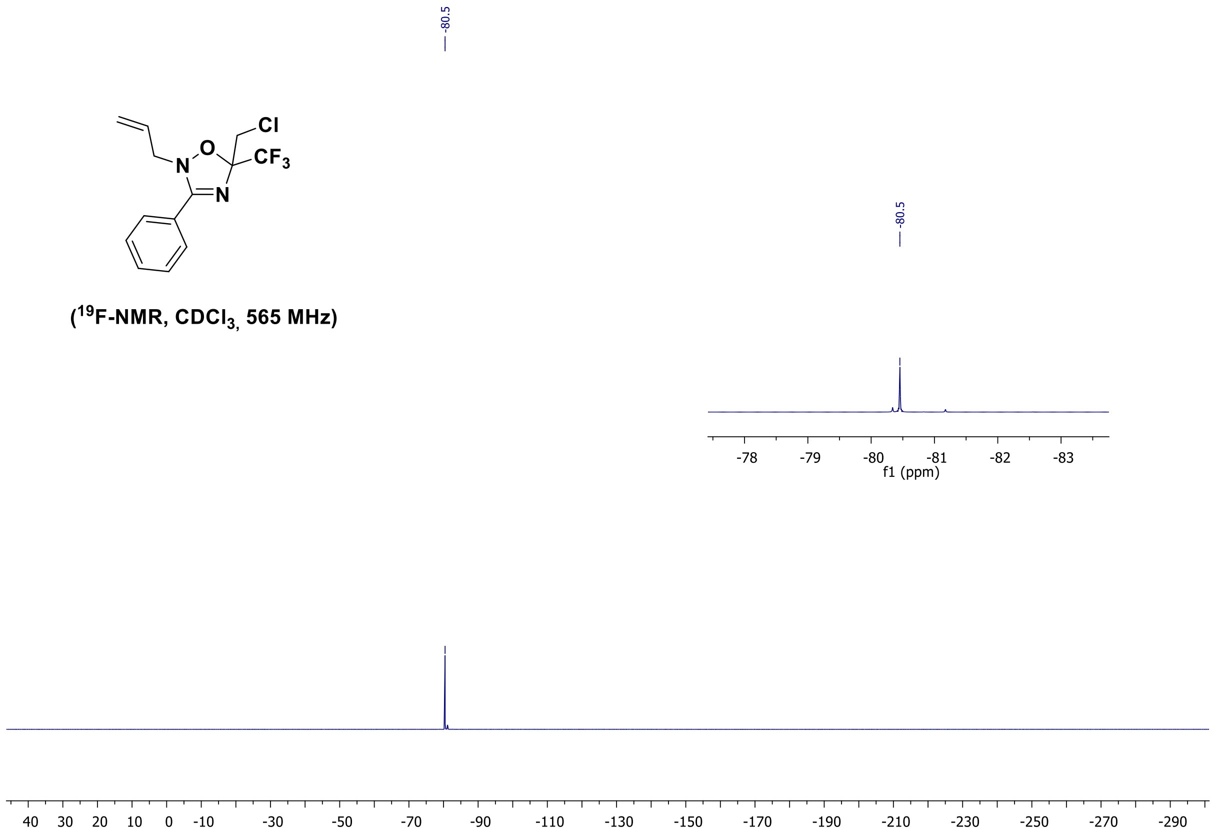

(^{19}F-NMR, $CDCl_3$, 565 MHz)

3-(4-methoxyphenyl)-2,5-dimethyl-5-(trifluoromethyl)-2,5-dihydro-1,2,4-oxadiazole (48)

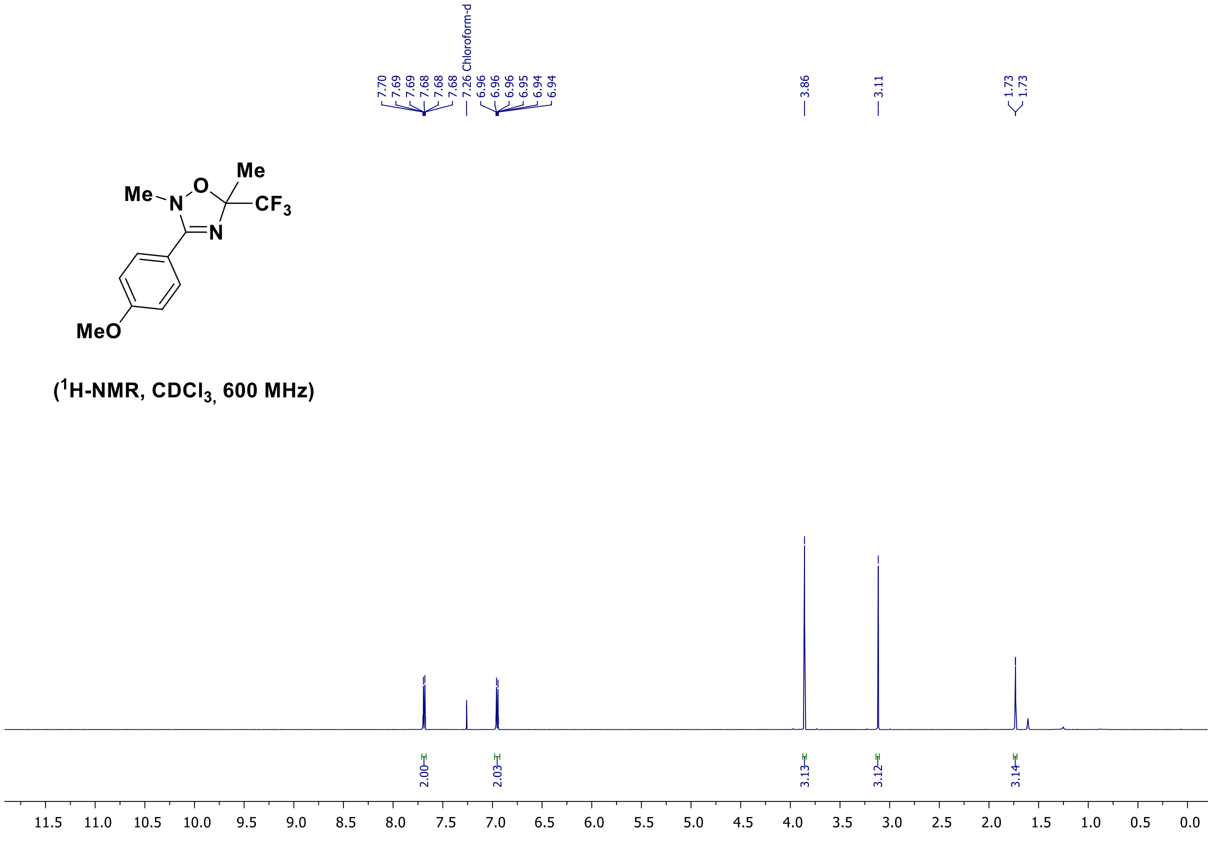

(^{1}H-NMR, $CDCl_3$, 600 MHz)

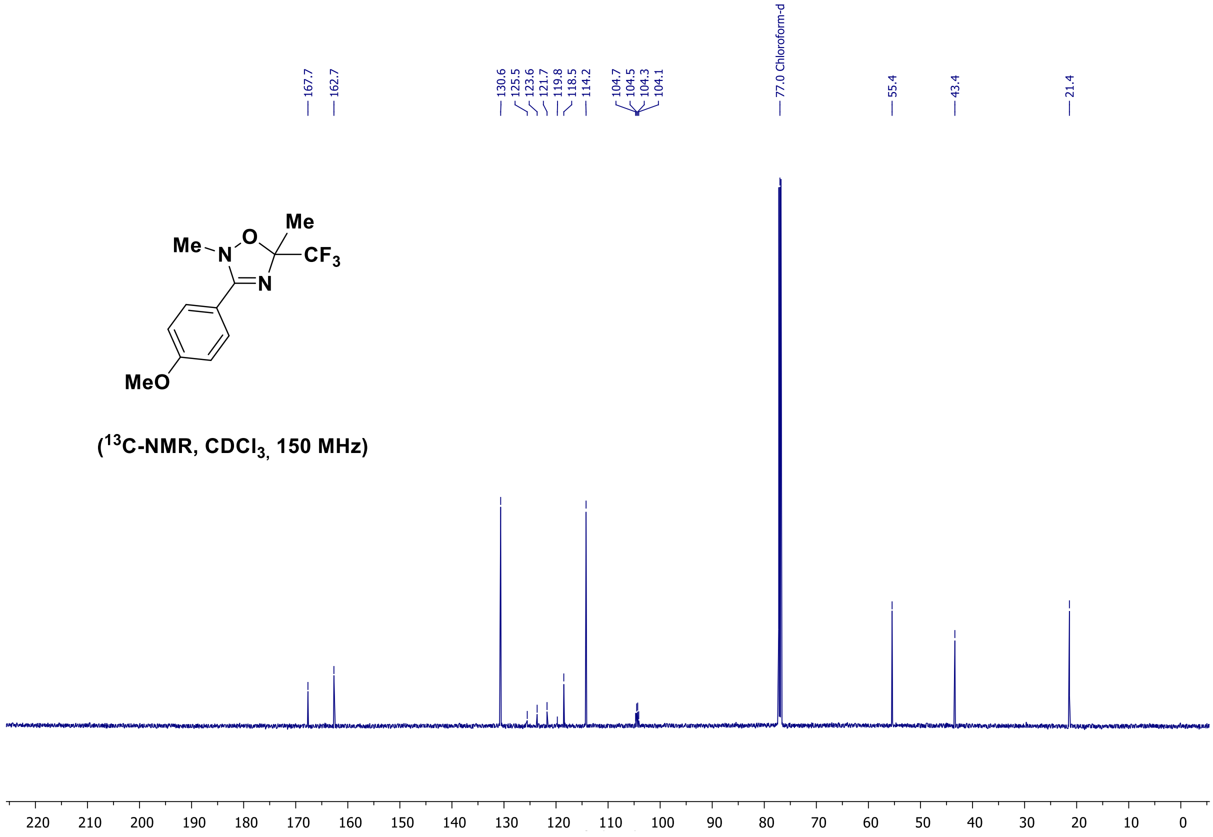

(^{13}C-NMR, $CDCl_3$, 150 MHz)

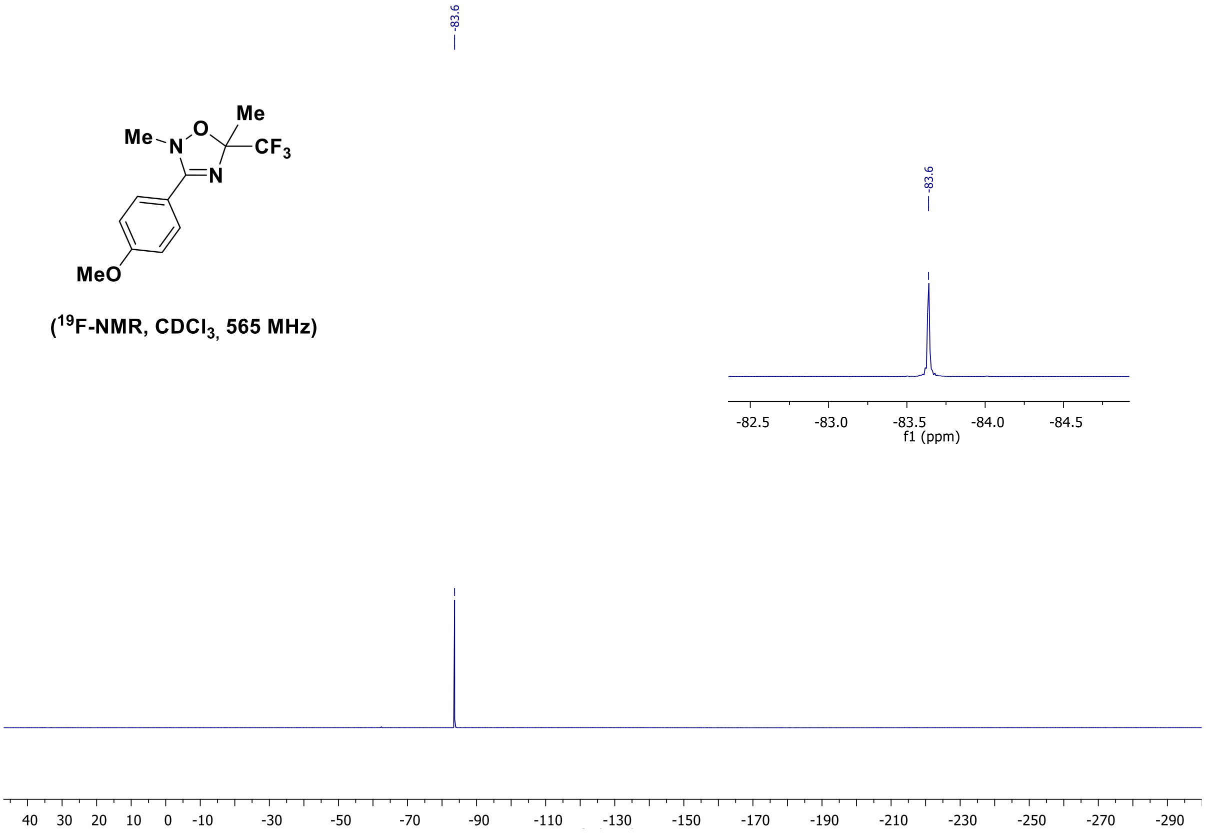

(^{19}F-NMR, $CDCl_3$, 565 MHz)

2,5-dimethyl-5-(trifluoromethyl)-3-(4-(trifluoromethyl)phenyl)-2,5-dihydro-1,2,4-oxadiazole (49)

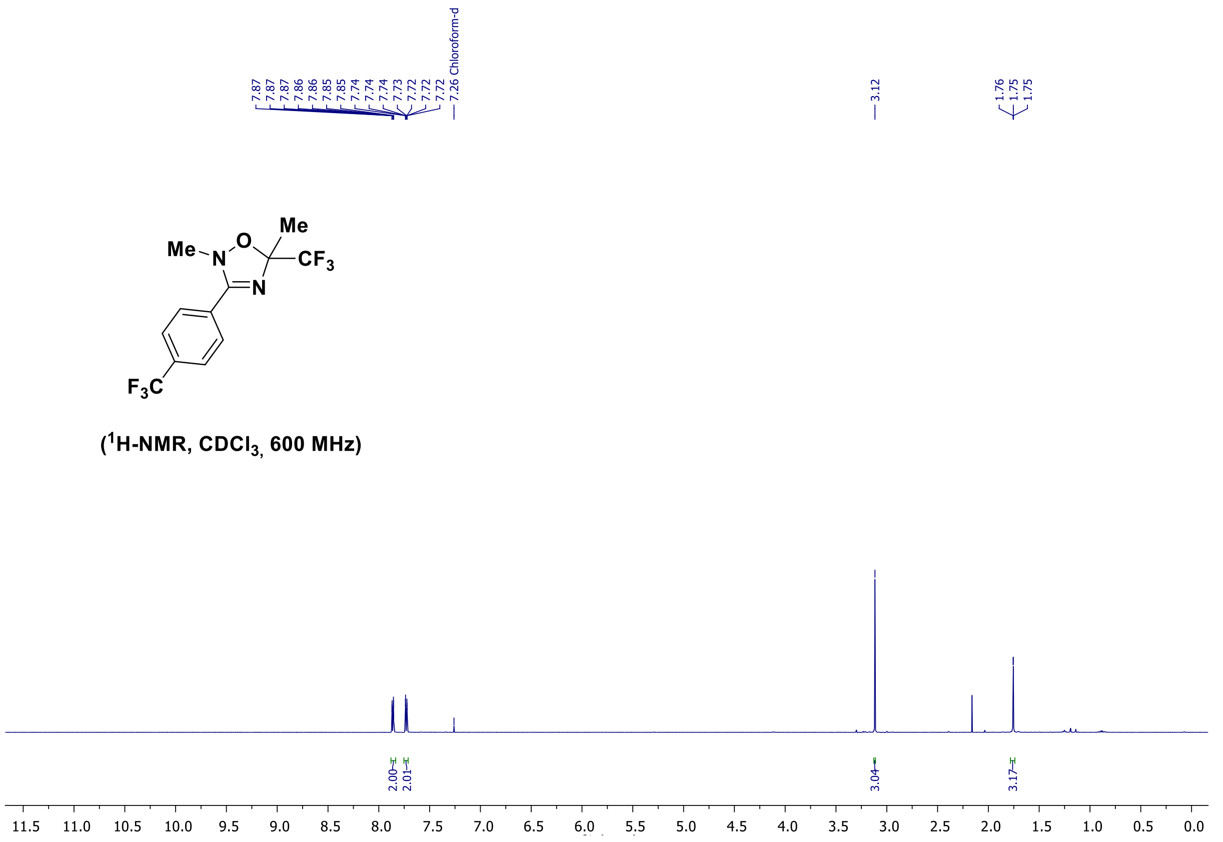

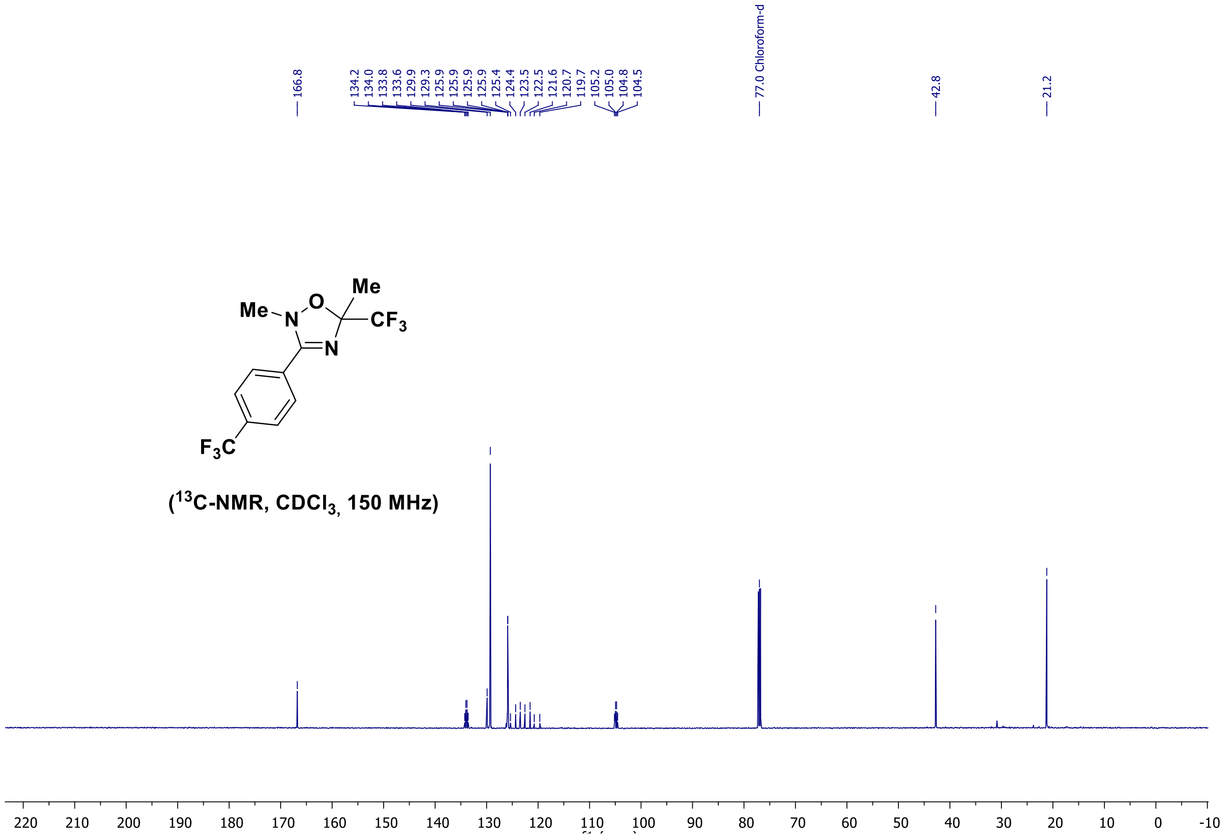

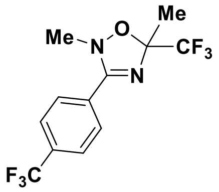

(^{19}F-NMR, $CDCl_3$, 565 MHz)

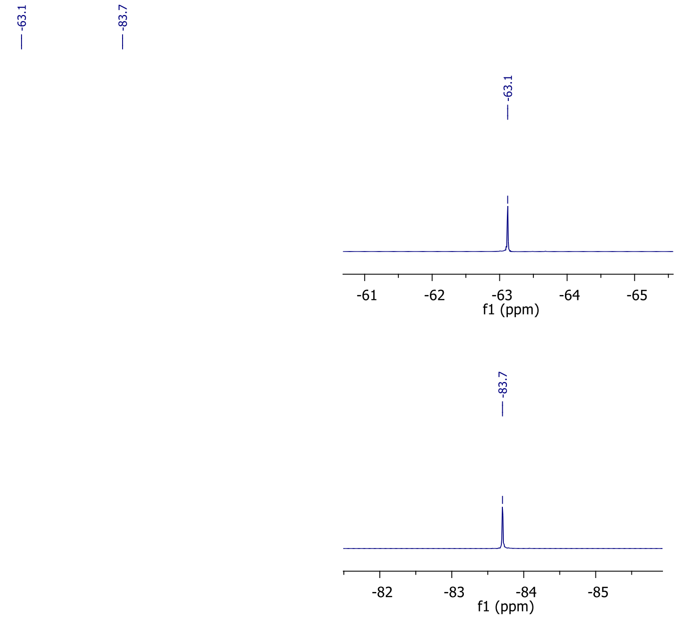

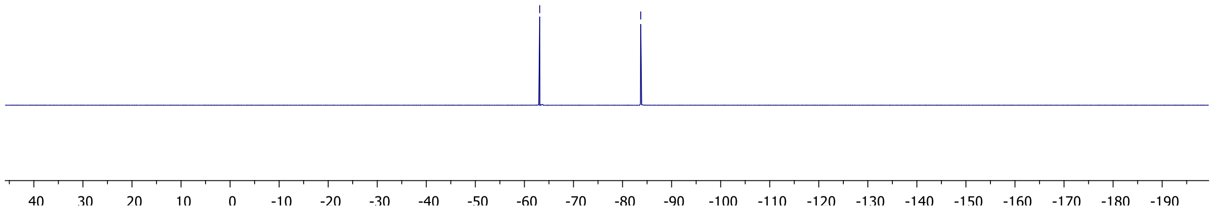

X-Ray Analysis for compounds 2, 13, 18, 23, 24 and 46

X-ray intensity data measured on STOE STADIVARI diffractometer equipped with multilayer monochromator, Mo K/α and Cu K/α micro focus sealed tube and Oxford cooling system. Structures solved by *Direct Methods*. Non-hydrogen atoms were refined with *anisotropic displacement parameters*. Hydrogen atoms inserted at calculated positions and refined with riding model. Used Software: X-Area Recipe[i], *X-Area Pilatus3_SV*[ii], *OLEX2*[iii] for cell refinement, data collection, structure solution, refinement, molecular diagrams and graphical user-interface, *Shelxl*[iv] for refinement and graphical user-interface *SHELXT-2015*[v] for structure solution, *SHELXL-2015*[vi] for refinement, *Platon*[vii] for symmetry check. Experimental data and CCDC-Codes Experimental data (Available online: http://www.ccdc.cam.ac.uk/conts/retrieving.html) can be found in Table 1. Tables 2 to 7 show data related to sample, crystal data, data collection and structure refinement Figure 1,3,5,7,9, and 11 show molecular structures. Figures 2,4,5,6,8,10 and 12 are related to quality aspects.

Table 2. Experimental parameter and CCDC-Code.

Sample	Machine	Source	Temp.	Detector Distance	Time/ Frame	#Frames	CCDC
			[K]	[mm]	[s]		
**Compound 23** (DC707)	Stoe Stadivari	Mo	100	50	30	3381	2493076
**Compound 2** (DC716)	Stoe Stadivari	Mo	100	50	10	6911	2493077
**Compound 46** (DC721)	Stoe Stadivari	Cu	100	40	2	11276	2493078
**Compound 13** (DC723)	Stoe Stadivari	Mo	100	50	10	4569	2493079
**Compound 24** (DC747)	Stoe Stadivari	Mo	100	50	2	4981	2493080
**Compound 18** (DC757)	Stoe Stadivari	Mo	100	50	1	5761	2493081

Compound 2

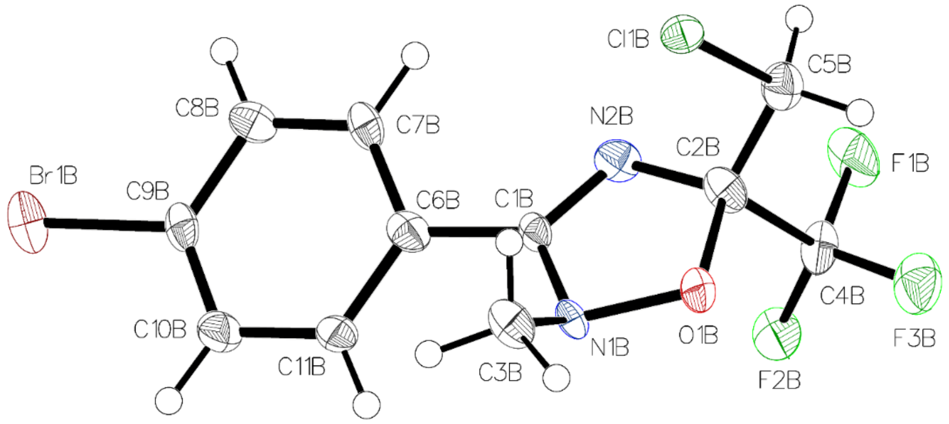

Figure 1 One of two molecules in the asymmetric drawn with 50% displacement ellipsoid. The bond precision for C-C bonds is 0.0170Å. The spacegroup of *P212121* is chiral but the second molecule in the asymmetric unit is in S and not in R as in the one visualized at the position C2B. The resulting crystal is built of both species and characterizes the racemic compound. 2 B-Alerts had to be discussed in the Cif-file. Both seem to be related to a twinning which could not be explained with the work during the model built. Still the structure proof is reliable.

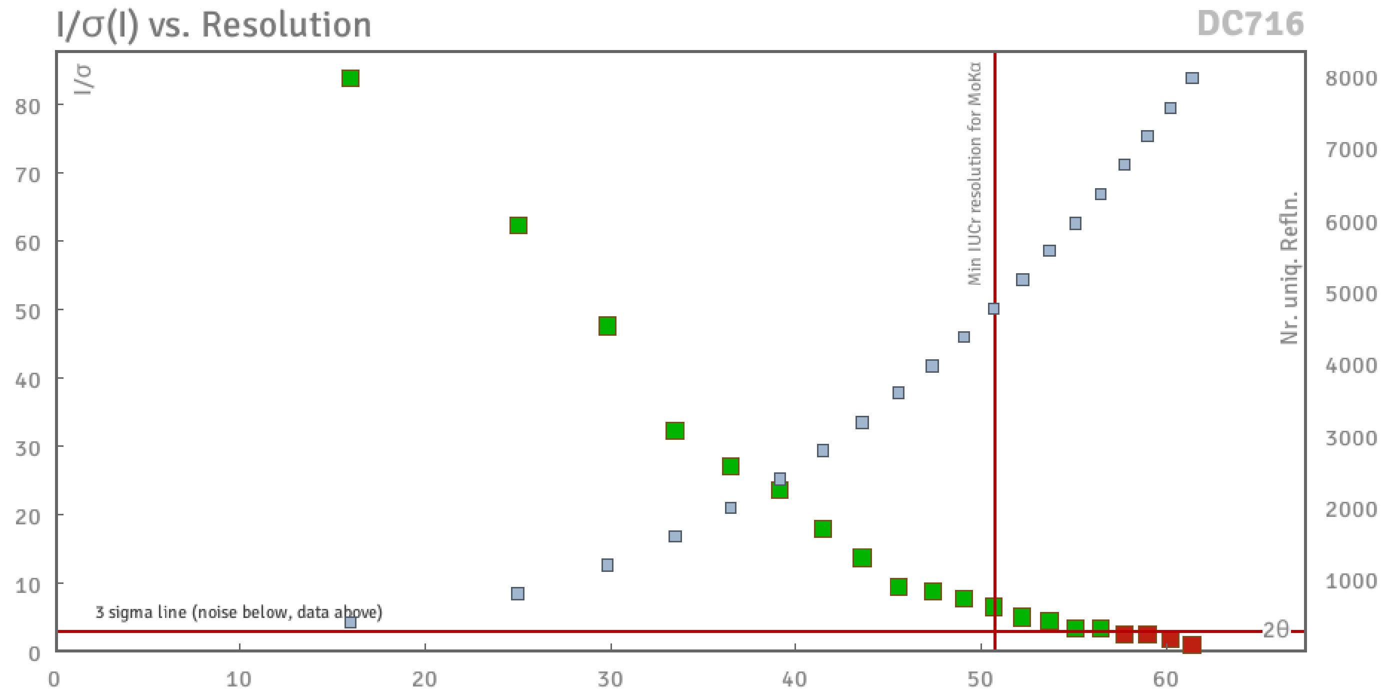

Figure 2. Data quality I 3 sigma line: All data are above the “noise level” line along the min IUCR definition.

Table 1. Sample and crystal data, Data collection and structure refinement

Identification code	DC716
Empirical formula	$C_{11}H_9BrClF_3N_2O$
Formula weight	357.56
Temperature/K	100
Crystal system	orthorhombic
Space group	$P2_12_12_1$
a/Å	27.4602(8)
b/Å	12.7464(3)
c/Å	7.4938(2)
α/°	90
β/°	90
γ/°	90
Volume/Å^3	2622.97(12)
Z	8
ρ_{calc}g/cm^3	1.811
μ/mm^{-1}	3.366
F(000)	1408.0
Crystal size/mm^3	0.4 × 0.25 × 0.05
Radiation	Mo Kα (λ = 0.71073)
2Θ range for data collection/°	5.48 to 51.354
Index ranges	-33 ≤ h ≤ 32, -15 ≤ k ≤ 15, -8 ≤ l ≤ 9
Reflections collected	38494
Independent reflections	4954 [R_{int} = 0.0540, R_{sigma} = 0.0405]
Data/restraints/parameters	4954/0/347
Goodness-of-fit on F^2	1.079

Final R indexes [I>=2σ (I)]	R_1 = 0.0728, wR_2 = 0.1870
Final R indexes [all data]	R_1 = 0.0837, wR_2 = 0.1937
Largest diff. peak/hole / e Å^{-3}	2.40/-0.80
Flack parameter	0.5

Compound 13

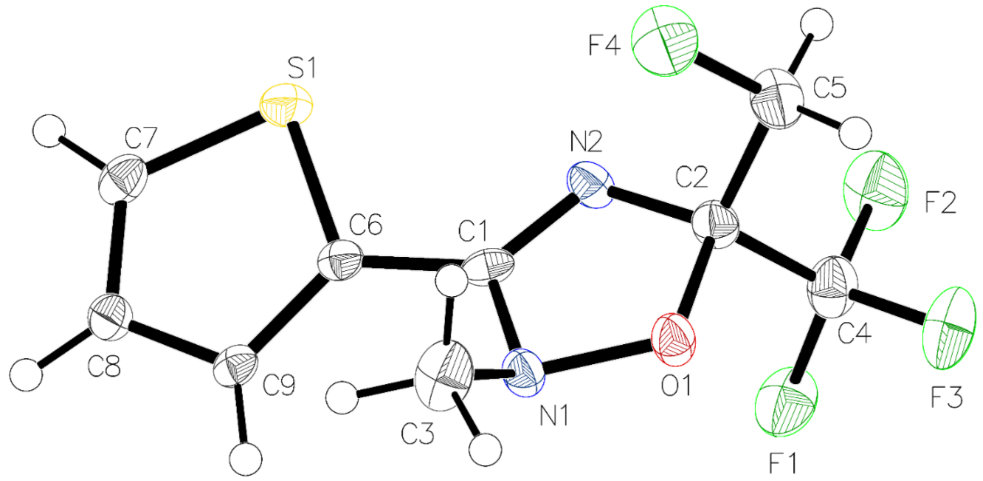

Figure 3. Asymmetric drawn with 50% displacement ellipsoid. The bond precision for C-C bonds is 0.0042Å.

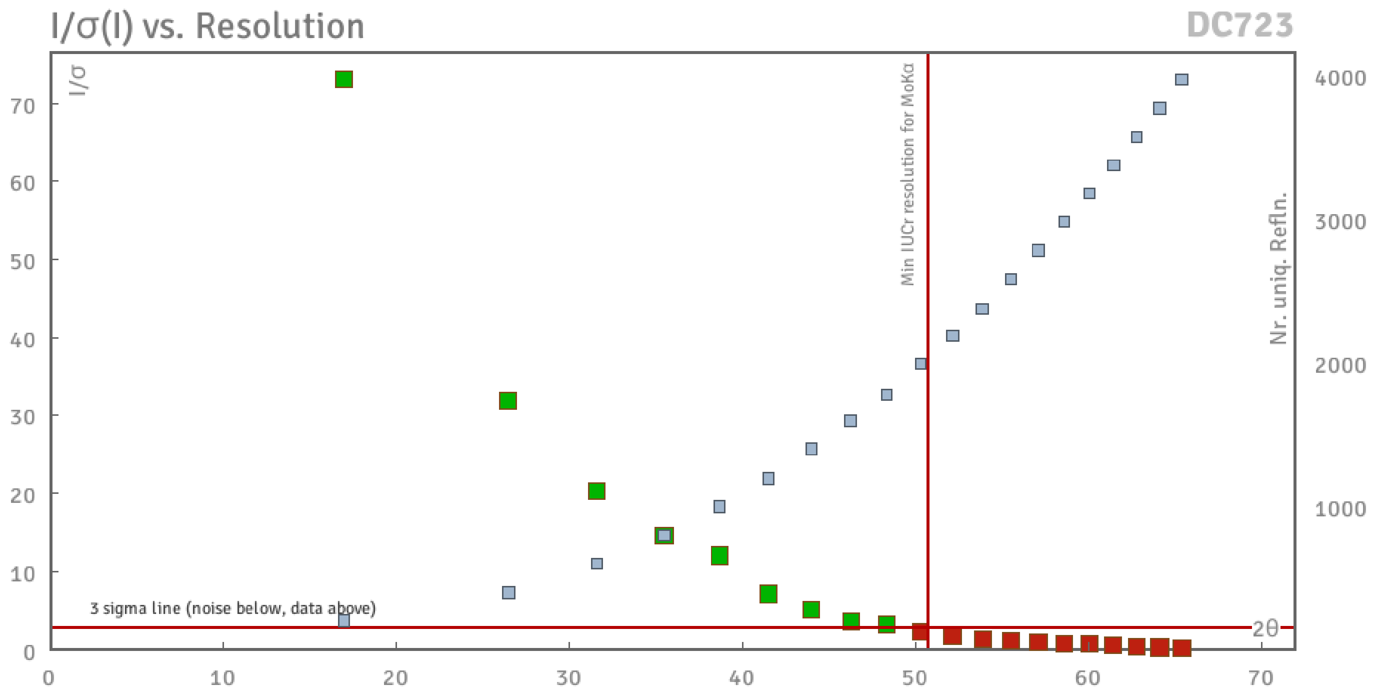

Figure 4. Data quality 3 sigma line: Almost all data are above the “noise level” line along the min IUCR definition.

Table 4. Sample and crystal data, Data collection and structure refinement

Identification code	DC723
Empirical formula	$C_9H_8F_4N_2OS$
Formula weight	268.23
Temperature/K	100
Crystal system	orthorhombic
Space group	Pbca
a/Å	7.6657(5)
b/Å	11.8373(8)
c/Å	24.323(2)
α/°	90
β/°	90
γ/°	90
Volume/Å^3	2207.1(3)
Z	8
ρ_{calc}g/cm^3	1.614
μ/mm^{-1}	0.333
F(000)	1088.0
Crystal size/mm^3	0.3 × 0.187 × 0.01
Radiation	Mo Kα (λ = 0.71073)
2Θ range for data collection/°	6.282 to 50.694
Index ranges	-9 ≤ h ≤ 9, -14 ≤ k ≤ 14, -28 ≤ l ≤ 29
Reflections collected	31974
Independent reflections	2020 [R_{int} = 0.1131, R_{sigma} = 0.0545]
Data/restraints/parameters	2020/0/155
Goodness-of-fit on F^2	0.999

Final R indexes [I>=2σ (I)]	R_1 = 0.0421, wR_2 = 0.1107
Final R indexes [all data]	R_1 = 0.0728, wR_2 = 0.1207
Largest diff. peak/hole / e Å^{-3}	0.55/-0.37

Compound 18

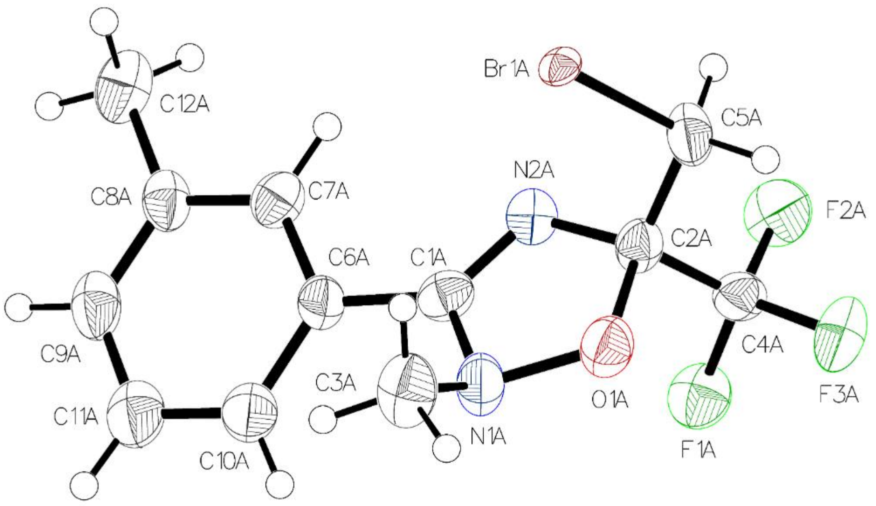

Figure 5. One of two molecules in the asymmetric drawn with 50% displacement ellipsoid. The bond precision for C-C bonds is 0.0107Å

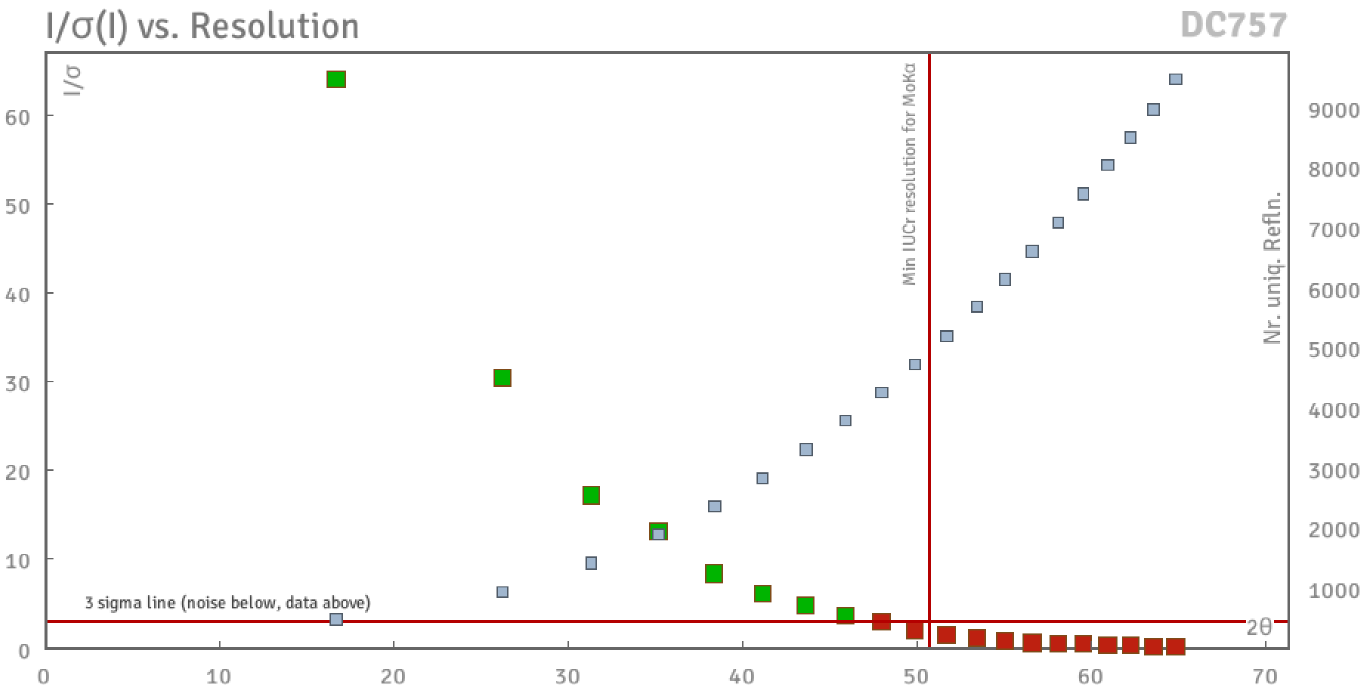

Figure 6. Data quality 3 sigma line: Almost all data are above the “noise level” line along the min IUCR definition.

Table 5. Sample and crystal data, Data collection and structure refinement

Identification code	DC757
Empirical formula	$C_{12}H_{12}BrF_3N_2O$
Formula weight	337.15
Temperature/K	100
Crystal system	monoclinic
Space group	$P2_1/c$
a/Å	9.5398(6)
b/Å	14.6986(7)
c/Å	19.7485(14)
α/°	90
β/°	103.711(5)
γ/°	90
Volume/Å^3	2690.3(3)
Z	8
ρ_{calc}g/cm^3	1.665
μ/mm^{-1}	3.084
F(000)	1344.0
Crystal size/mm^3	0.3 × 0.233 × 0.1
Radiation	Mo Kα (λ = 0.71073)
2Θ range for data collection/°	5.544 to 50.694
Index ranges	-11 ≤ h ≤ 11, -17 ≤ k ≤ 17, -23 ≤ l ≤ 23
Reflections collected	52144
Independent reflections	4922 [R_{int} = 0.0793, R_{sigma} = 0.0971]
Data/restraints/parameters	4922/10/347
Goodness-of-fit on F^2	1.007

Final R indexes [I>=2σ (I)]	R_1 = 0.0490, wR_2 = 0.1402
Final R indexes [all data]	R_1 = 0.0842, wR_2 = 0.1471
Largest diff. peak/hole / e Å^{-3}	0.86/-0.71

Compound 23

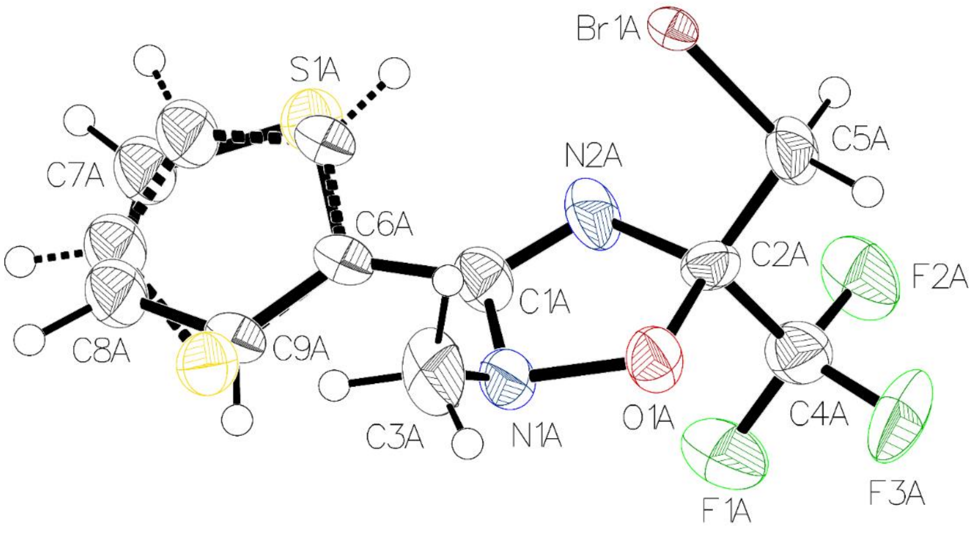

Figure 7. One of two molecules in the asymmetric drawn with 50% displacement ellipsoid. The bond precision for C-C bonds is 0.0137Å. The degree of disorder is 24%.

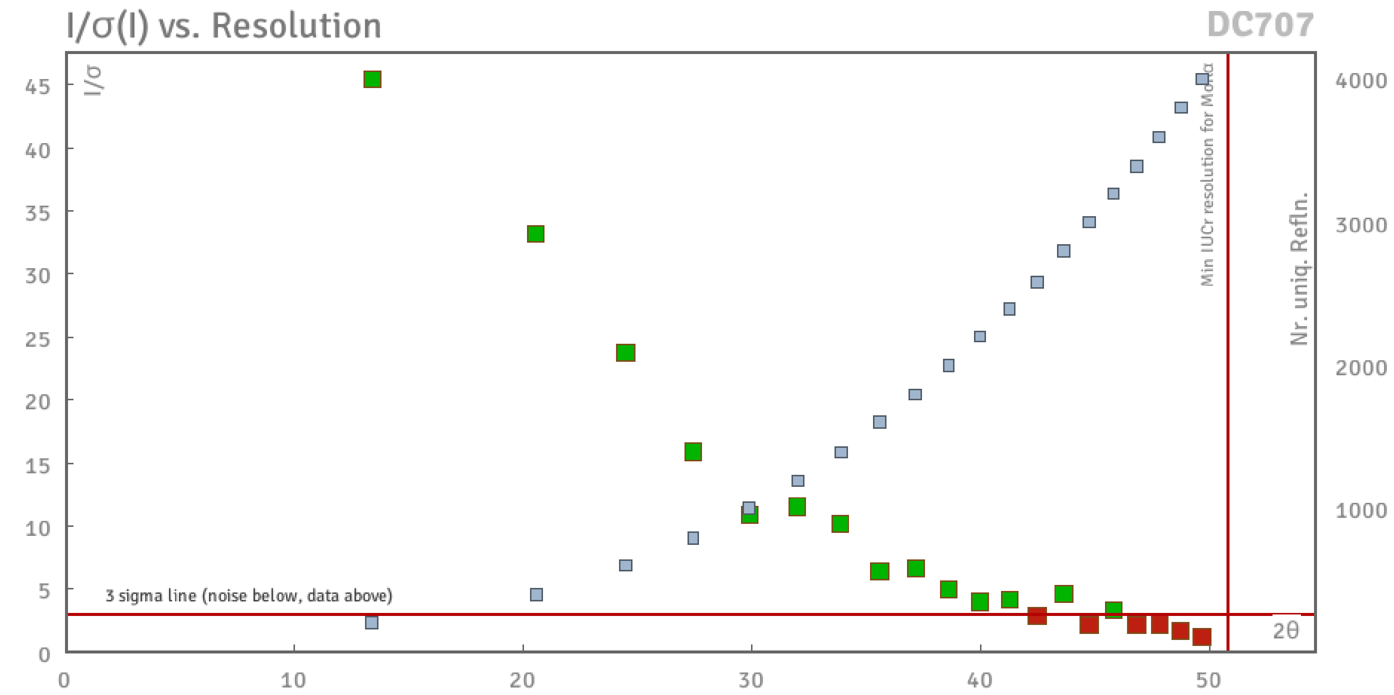

Figure 8. Data quality I 3 sigma line: Data higher 40 degree start getting close and below the "noise level" line along the min IUCR definition. The result still allows the proof of the structure.

Table 6. Sample and crystal data, Data collection and structure refinement

Identification code	DC707
Empirical formula	$C_9H_8BrF_3N_2OS$
Formula weight	329.14
Temperature/K	100
Crystal system	monoclinic
Space group	$P2_1/c$
a/Å	8.7339(10)
b/Å	14.4009(8)
c/Å	19.2869(13)
α/°	90
β/°	102.089(7)
γ/°	90

Volume/Å^3	2372.0(3)
Z	8
ρ_{calc}g/cm^3	1.843
μ/mm^{-1}	3.665
F(000)	1296.0
Crystal size/mm^3	0.14 × 0.08 × 0.02
Radiation	Mo Kα (λ = 0.71073)
2Θ range for data collection/°	5.726 to 50.098
Index ranges	-8 ≤ h ≤ 10, -16 ≤ k ≤ 17, -22 ≤ l ≤ 16
Reflections collected	19213
Independent reflections	4114 [R_{int} = 0.1399, R_{sigma} = 0.3041]
Data/restraints/parameters	4114/156/310
Goodness-of-fit on F^2	0.963
Final R indexes [I>=2σ (I)]	R_1 = 0.0600, wR_2 = 0.1563
Final R indexes [all data]	R_1 = 0.1709, wR_2 = 0.1730
Largest diff. peak/hole / e Å^{-3}	1.21/-1.12

Compound 24

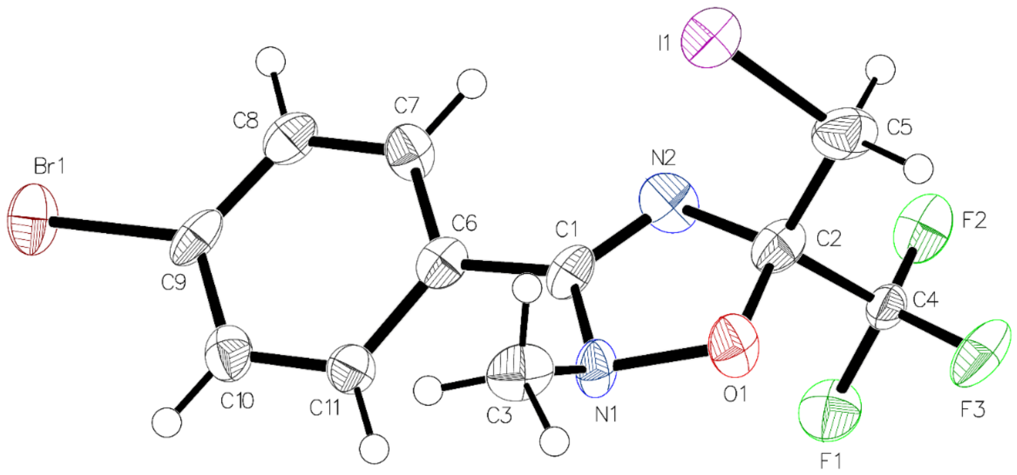

Figure 9. Asymmetric drawn with 50% displacement ellipsoid. The bond precision for C-C bonds is 0.0169Å.

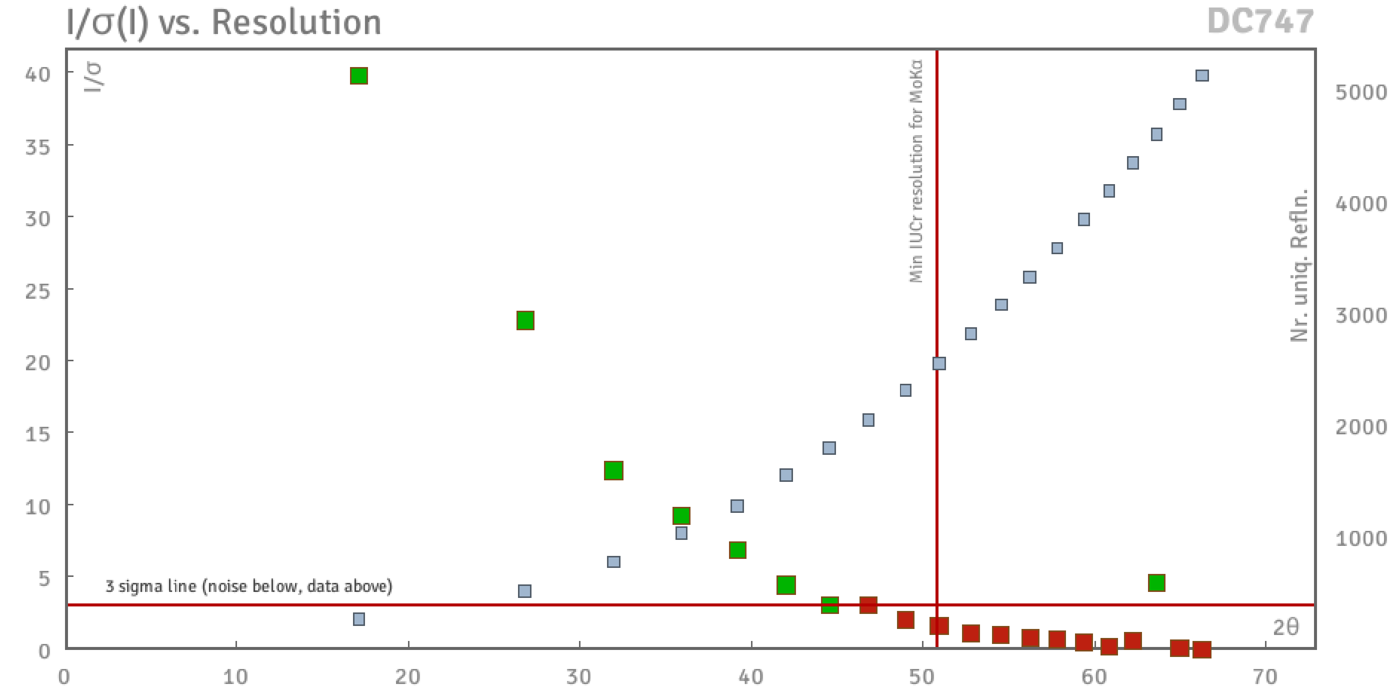

Figure 10. Data quality 3 sigma line: Almost all data are above the "noise level" line along the min IUCR definition.

Table 7. Sample and crystal data, Data collection and structure refinement

Identification code	DC747
Empirical formula	$C_{11}H_9BrF_3IN_2O$
Formula weight	449.01
Temperature/K	100
Crystal system	monoclinic
Space group	$P2_1/c$
a/Å	13.9854(13)
b/Å	12.3430(14)
c/Å	8.1470(7)
α/°	90
β/°	102.027(7)

γ/°	90
Volume/Å^3	1375.5(2)
Z	4
ρ_{calc}g/cm^3	2.168
μ/mm^{-1}	5.260
F(000)	848.0
Crystal size/mm^3	0.4 × 0.217 × 0.1
Radiation	Mo Kα (λ = 0.71073)
2Θ range for data collection/°	6.29 to 50.696
Index ranges	-16 ≤ h ≤ 16, -14 ≤ k ≤ 14, -9 ≤ l ≤ 9
Reflections collected	17829
Independent reflections	2504 [R_{int} = 0.1449, R_{sigma} = 0.1026]
Data/restraints/parameters	2504/46/173
Goodness-of-fit on F^2	1.006
Final R indexes [I>=2σ (I)]	R_1 = 0.0685, wR_2 = 0.1725
Final R indexes [all data]	R_1 = 0.1168, wR_2 = 0.2059
Largest diff. peak/hole / e Å^{-3}	2.06/-2.12

Compound 46

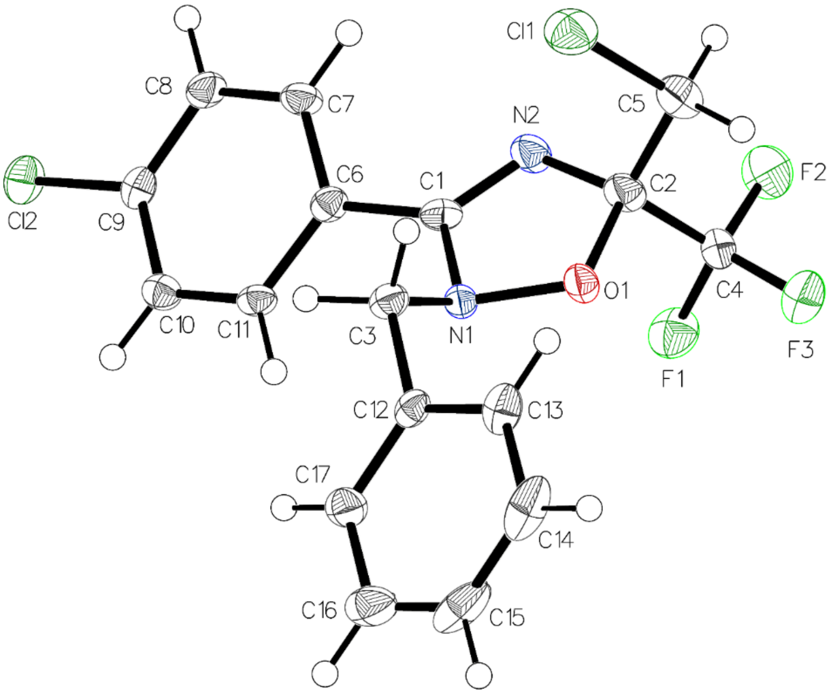

Figure 11. Asymmetric drawn with 50% displacement ellipsoid. The bond precision for C-C bonds is 0.0052Å.

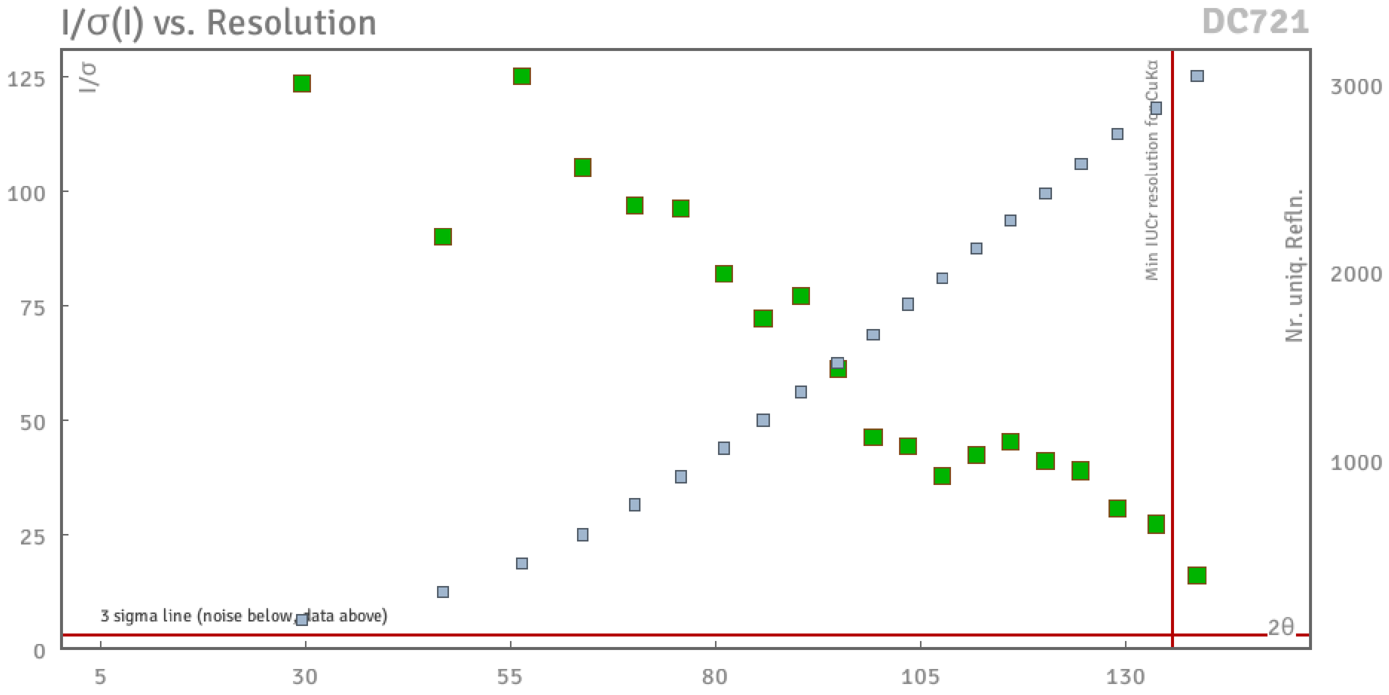

Figure 12. Data quality 3 sigma line: All data are above the "noise level" line along the min IUCR definition.

Table 8. Sample and crystal data, Data collection and structure refinement

Identification code	DC721
Empirical formula	$C_{17}H_{13}Cl_2F_3N_2O$
Formula weight	389.19
Temperature/K	100
Crystal system	monoclinic
Space group	$P2_1/n$
a/Å	10.8042(4)
b/Å	13.9249(4)
c/Å	11.1764(4)
α/°	90
β/°	99.627(3)
γ/°	90
Volume/Å^3	1657.78(10)
Z	4
ρ_{calc}g/cm^3	1.559
μ/mm^{-1}	3.899
F(000)	792.0
Crystal size/mm^3	0.34 × 0.19 × 0.06
Radiation	Cu Kα (λ = 1.54186)
2Θ range for data collection/°	10.238 to 141.27
Index ranges	-12 ≤ h ≤ 11, -15 ≤ k ≤ 16, -13 ≤ l ≤ 7
Reflections collected	23115
Independent reflections	2913 [R_{int} = 0.0218, R_{sigma} = 0.0116]
Data/restraints/parameters	2913/0/226
Goodness-of-fit on F^2	1.072

Final R indexes [I>=2σ (I)]	R_1 = 0.0566, wR_2 = 0.1573
Final R indexes [all data]	R_1 = 0.0589, wR_2 = 0.1593
Largest diff. peak/hole / e $Å^{-3}$	0.94/-0.63

Computational Details

Methods

Geometry calculations and thermochemistry were performed using the Gaussian 16 program.[2] The stationary points on the energy hypersurface were studied using the DFT M06-2X functional.[3] The basis set chosen for performing geometry optimizations and subsequent vibrational analysis was def2-TZVP.[4-5] Vibrational analysis was conducted to examine the nature of the critical points. The molecules were considered solutes in a polarized continuum (THF) within the Solvation Model based on density (SMD).[6] The relative free energies reported in this section were calculated at 195 K and expressed in kcal mol^{-1}. Single-point calculations using the coupled cluster method, CCSD(T), were performed to refine the energy. Single-point CCSD(T) calculations were performed using geometries optimized at the M06-2X/def2-TZVP level with the same basis set using the DLPNO approximation[7] available in ORCA software[8], version 6.0. To compute the free energies at the CCSD(T)/def2-TZVP level, the potential energies at the CCSD(T)/def2-TZVP level were combined with the thermochemistry calculated at the M06-2x/def-TZVP level. Calculations related to conformational sampling were conducted using CREST,[9] then the most stable conformers were optimized at DFT level. Quantitative evaluation of the steric hindrance and calculation of the Fukui function were performed using the package Multiwfn.[10] Images were generated using the graphical software Jmol.[11]

Earlier calculations related to the bromide compound were conducted with explicit solvent only (PCM) (Fig. 13a). However, subsequent studies have considered also explicit solvent simulation (explicit solvation + PCM) (Fig. 13b), due to the peculiar behaviour observed in the case of brominated compounds. Since the same reaction performed with bromine gave the two products in comparable yield, explicit solvent molecules were introduced in the calculation to better and more realistically account for all possible interactions and steric effects.

This involves the introduction of coordinated molecules to the Li atom. Experimentally, the reaction between 1,2,4-oxodiazole and methyl iodide was conducted in tetrahydrofuran; thus, three molecules of THF were added to the calculations.

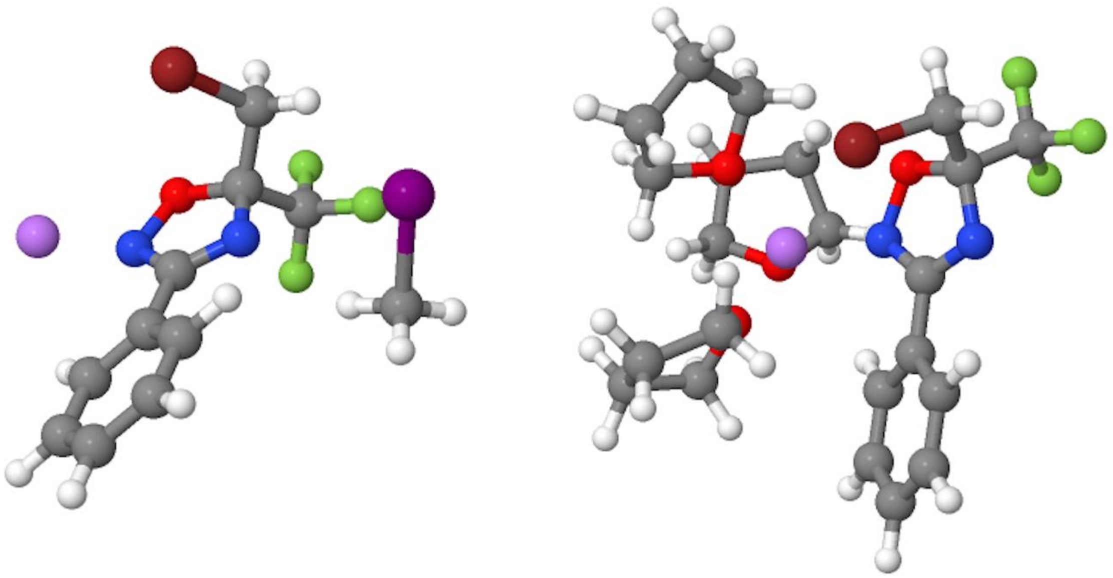

a) **Int-Br** calculation using PCM only

b) **Int-Br** with three THF molecules and PCM

Figure 13. Solvent simulation with and without explicit solvent molecules.

The nucleophilicity of the two nitrogen atoms was evaluated in each reactant with the three different halogen atoms for each structure. This evaluation was conducted using the Fukui function,[12] a significant principal in conceptual density functional theory (CDFT). This function calculates the frontier orbitals and describes the electronic density distribution after the addition or removal of electrons. This enables the prediction of the most electrophilic and nucleophilic sites within a molecule.
Fukui functions are also defined as derivatives of the electron density with respect to the number of electrons at a constant potential. Therefore, by adding or removing an electron while maintaining a constant potential, we can calculate the density changes at each point. These electrons are then added/removed from the frontier orbitals.
The Fukui function for electrophilic attack f- is:

$$f^{-}(r) = \rho_N(\boldsymbol{r}) - \rho_{N-1}(\boldsymbol{r}) \approx \rho^{HOMO}(\boldsymbol{r})$$

The Fukui function for nucleophilic attack f+ is:

$$f^{+}(r) = \rho_{N+1}(\boldsymbol{r}) - \rho_N(\boldsymbol{r}) \approx \rho^{LUMO}(\boldsymbol{r})$$

where N denotes the number of electrons in the system.
Finally, to determine the condensed local nucleophilicity index:

$$N_{Nu}^{A} = N_{Nu} f_A^{-}$$

Condensed nucleophilicity indices were calculated for the most stable conformations with explicit solvation, considering the different orientations of the halogen atom forming dihedral angles with the oxygen atom of approximately 60, 180 and -60 degrees, with fluoride, bromine and iodine (Table 9), respectively 1 for 60 degrees, 2 for 180 degrees, and 3 for -60 degrees. (Example for clarity: Int-F1 refers to the Int-F compound with the fluorine atom at 60 degrees relative to the oxygen in the ring).

Table 9. Condensed nucleophilicity indices, N_{Nu}^{A}, for nitrogen at the 2 and 4-position. Expressed as *e*eV*, where *e* is the elementary charge. The figure on the right shows the **Int-F** molecule, in which the nitrogen atom near the oxygen in the isoxazole ring is referred to as N2, while the nitrogen at position 4 is referred to as N4.

Compound	*N2*	*N4*
Int-F1	0.69005	0.52398
Int-F2	0.62602	0.51844
Int-F3	0.62575	0.5673
Int-I1	0.6361	0.53307
Int-I2	0.60085	0.50644
Int-I3	0.57774	0.46737
Int-Br	0.57182	0.49387

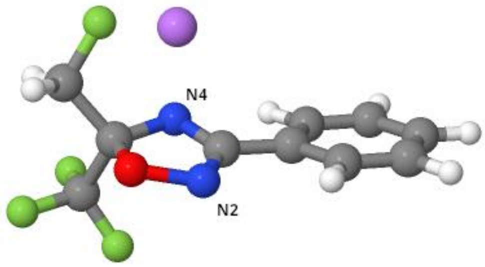

According to the calculations of nucleophilicity, the reactivity could be explained by a steric effect.

A conformational search was performed to determine the best conformation:

- coordination sites for lithium atoms,
- for the three solvent molecules surrounding the cation,
- orientation of the halogen atom attached to the oxadiazole ring.

It was found to be the most stable compound for the cases with fluoride, bromine, and iodide, as summarized in Figure 14.

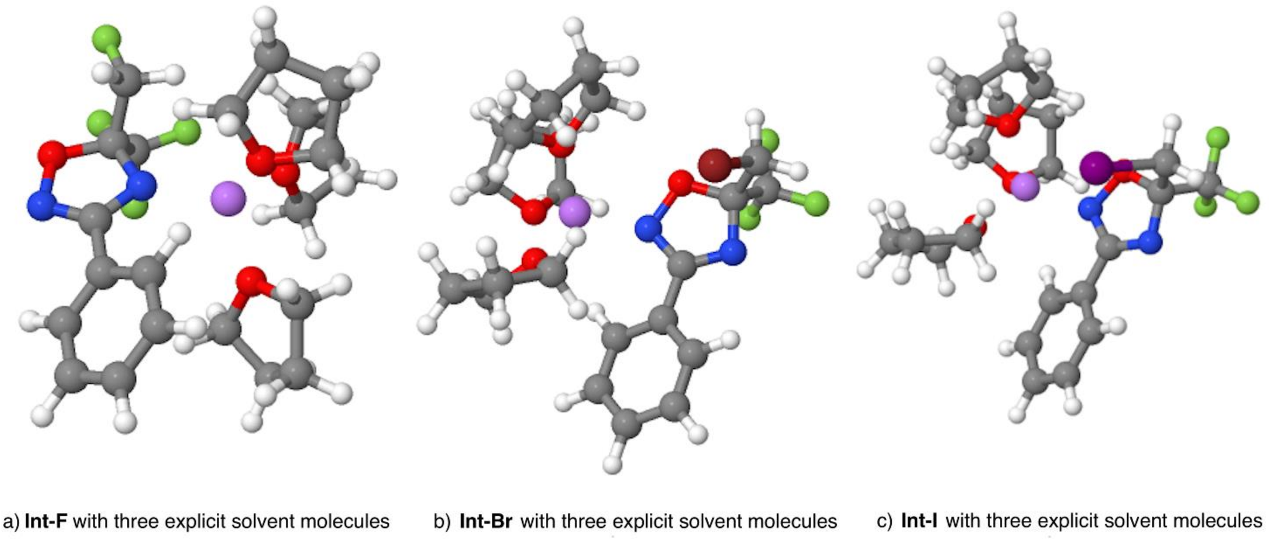

Figure 14. Results of the conformational search for the compound with halogen's dihedral angle respect to oxygen atom of oxadiazole ring with fluoride (left, in green), bromine (center, in dark red), and iodine (right, in purple).

In the case of the compound with fluorine, the most stable conformation was with the halogen atom on the opposite side of the lithium coordination at the nitrogen in the 4-position, forming a dihedral angle of approximately 60° with oxygen (Figure 14a). Conversely, for the compound with iodine and bromine, the halogen prefers to be above the ring, while coordination between lithium and nitrogen occurs at the 2-position (Figure 14b and 14c).

Energy decomposition analysis (EDA) is an important class of methods for exploring the nature of the interactions between fragments in a chemical system. In the current study, Liu's energy decomposition[13] (EDA-SBL) was used. In this method, the total molecular energy is expressed as:

$$E = E_{steric} + E_{electrostatic} + E_{quantum}$$

The steric term corresponds to the energy derived from the Weizsäcker kinetic functional,[14] representing the exact kinetic energy under the assumption that electrons in the current system behave as noninteracting bosons. The electrostatic term is the sum of all the classical Coulomb interactions of the particles in the system, and the quantum term is the energy caused by the quantum effect.

Cartesian Coordinates and energies:

Int-F

Atom	X	Y	Z (Angstrom)
8	1.289789	-0.006651	-1.579827
6	1.594440	0.285624	-0.225156
7	0.344432	0.488525	0.442197
6	-0.553036	0.030306	-0.466412
7	-0.109361	-0.304804	-1.636180
6	2.339727	-0.940453	0.348967
9	3.533997	-1.105098	-0.243538
6	2.542420	1.487113	-0.129117
9	1.764627	2.653914	0.000924
6	-1.996491	-0.043830	-0.156637

6	-2.427130	0.059074	1.163656
6	-3.781967	0.003897	1.464860
6	-4.713685	-0.160152	0.449468
6	-4.286978	-0.269277	-0.870183
6	-2.936583	-0.208951	-1.174155
9	1.649398	-2.064624	0.174281
9	2.568314	-0.810239	1.662519
1	-1.694596	0.163605	1.954149
1	-2.600020	-0.285372	-2.199938
1	-4.108224	0.082092	2.494341
1	-5.011152	-0.398209	-1.664685
1	-5.769932	-0.205229	0.683471
1	3.180808	1.445230	0.752186
1	3.136513	1.593527	-1.033477
3	0.190489	2.281948	1.077009

Energy -976.972697 (Hartree)

TS Int-F→2F

Atom	X	Y	Z (Angstrom)
8	2.794132	-0.983191	1.084905
6	1.650056	-1.436919	0.355648
7	0.904165	-0.259968	0.024319
6	1.673911	0.755137	0.380945
7	2.821596	0.476122	0.970915
6	2.188683	-2.088428	-0.937950
9	2.774180	-3.264686	-0.715831
6	0.897164	-2.469078	1.181633
9	0.397287	-1.836172	2.300481
6	1.322105	2.164196	0.117782
6	0.551317	2.487193	-0.997143
6	0.233986	3.811811	-1.258331
6	0.684093	4.815407	-0.409323
6	1.455459	4.494520	0.701155
6	1.777129	3.171810	0.965966
9	3.142936	-1.279221	-1.469823
9	1.254971	-2.255990	-1.860051
1	0.214059	1.700742	-1.660926
1	2.368497	2.913498	1.834957
1	-0.363113	4.060619	-2.126138
1	1.803106	5.275705	1.364875
1	0.432774	5.848686	-0.612855
1	0.066155	-2.887403	0.610390
1	1.567064	-3.263236	1.509347
6	-1.203876	-0.302374	0.000162
1	-1.156212	-0.844331	-0.926612
1	-1.189583	-0.822400	0.940656
1	-1.214540	0.772744	-0.007576
53	-3.764505	-0.380827	-0.031879
3	4.350881	-0.304455	0.073772

Energy -1314.479476 (Hartree)

4F

Atom	X	Y	Z (Angstrom)
8	2.817500	-1.475748	0.896410
6	1.537853	-1.710725	0.349079
7	1.055819	-0.392635	-0.000517
6	2.077236	0.471013	0.313549
7	3.126585	-0.093150	0.783895
6	1.722701	-2.549452	-0.932592
9	2.214712	-3.761117	-0.659580
6	0.673639	-2.464829	1.351451
9	0.555289	-1.675704	2.476991
6	2.029550	1.922296	0.068578

6	1.471538	2.417911	-1.108349
6	1.474904	3.782932	-1.351574
6	2.031082	4.654843	-0.423489
6	2.589118	4.160348	0.748668
6	2.590465	2.795789	0.996993
9	2.565452	-1.951854	-1.771904
9	0.561275	-2.716456	-1.572465
1	1.049916	1.734995	-1.835762
1	3.017300	2.401251	1.909995
1	1.044441	4.165584	-2.267906
1	3.020587	4.838491	1.473635
1	2.028275	5.720611	-0.614003
1	-0.322492	-2.663332	0.953890
1	1.160990	-3.397959	1.632996
6	-0.367826	-0.071106	0.031634
1	-0.902664	-0.721150	-0.657905
1	-0.784650	-0.183432	1.033848
1	-0.510439	0.955502	-0.291629
53	-4.163589	0.242414	-0.009682

Energy -1307.096728 (Hartree)

TS Int-F→2F

Atom	X	Y	Z (Angstrom)
8	-1.164481	-1.317420	-0.720228
6	-2.126338	-1.139926	0.334860
7	-2.025401	0.219709	0.755997
6	-1.170132	0.775922	-0.042730
6	-3.501772	-1.384052	-0.316882
9	-3.689028	-2.663434	-0.638256
6	-1.871681	-2.148062	1.444866
9	-0.645466	-1.853454	2.003015
6	-0.792621	2.199891	0.032887
6	-1.180923	2.953056	1.139044
6	-0.830064	4.291057	1.222569
6	-0.094237	4.883753	0.202857
6	0.291078	4.135965	-0.902550
6	-0.055406	2.795666	-0.989270
9	-3.565037	-0.683238	-1.479187
9	-4.512815	-0.991746	0.438561
1	-1.751040	2.480707	1.928244
1	0.239785	2.213926	-1.853581
1	-1.128498	4.872611	2.085317
1	0.861757	4.596493	-1.698582
1	0.179132	5.929222	0.270346
1	-2.639047	-2.061435	2.215410
1	-1.834802	-3.163999	1.052468
7	-0.582317	-0.010450	-0.985920
6	1.435427	-0.371912	-0.542555
1	1.459610	-1.030238	-1.392359
1	1.678349	0.669013	-0.666495
1	1.116274	-0.746800	0.415554
53	3.874392	-0.922664	0.002523
3	-1.621101	-0.668345	-2.511514

Energy -1314.480082 (Hartree)

Int-I

Atom	X	Y	Z (Angstrom)
6	-0.935236	0.906598	0.028695
6	1.121198	0.303308	-0.307452
7	0.266835	0.487584	0.669819
7	0.708263	0.474861	-1.563682
6	2.527719	-0.091390	-0.065256
6	3.033512	-0.072244	1.232083

6	3.349507	-0.481114	-1.121320
6	4.350394	-0.436976	1.470704
1	2.387887	0.230607	2.045962
6	4.666177	-0.844251	-0.879631
1	2.951900	-0.507002	-2.127758
6	5.168715	-0.822545	0.415956
1	4.738748	-0.420297	2.481140
1	5.300005	-1.148660	-1.702819
1	6.196294	-1.107985	0.603370
8	-0.710923	0.812300	-1.386177
6	-1.157335	2.415498	0.298532
9	-0.167744	3.122098	-0.316201
9	-1.123761	2.732455	1.583243
9	-2.303075	2.872621	-0.211130
6	-2.191665	0.152152	0.434184
1	-2.367029	0.256929	1.501875
1	-3.060015	0.483252	-0.127535
53	-1.989952	-1.933070	0.053112
3	0.311555	2.237194	-2.228307

Energy -1174.756440 (Hartree)

TS Int-F→4I

Atom	X	Y	Z (Angstrom)
6	-1.860165	-0.289934	0.839063
6	-0.992791	1.624748	0.203309
7	-0.649892	0.472851	0.756586
7	-2.263434	1.805416	-0.104136
6	-0.023608	2.705228	-0.072835
6	1.097657	2.859036	0.739140
6	-0.236123	3.581545	-1.135684
6	2.002491	3.879498	0.485130
1	1.250470	2.189620	1.575731
6	0.672543	4.597891	-1.387685
1	-1.107055	3.459255	-1.766776
6	1.792668	4.747494	-0.578836
1	2.870718	3.997371	1.120543
1	0.508046	5.272772	-2.217816
1	2.501528	5.541407	-0.777208
8	-2.884391	0.540496	0.282548
6	-2.226021	-0.505863	2.324400
9	-2.337450	0.655858	2.964288
9	-1.285798	-1.225152	2.943278
9	-3.388769	-1.151702	2.463297
6	-1.856707	-1.645347	0.140262
1	-1.090542	-2.309368	0.529424
1	-2.830872	-2.121004	0.207021
53	-1.471499	-1.389346	-1.945334
6	1.262362	-0.400136	0.535885
1	1.463428	0.304956	-0.250405
1	0.805996	-1.343523	0.298020
1	1.474837	-0.152908	1.559211
53	3.590290	-1.428150	0.244597
3	-3.119927	0.926950	-1.618943

Energy -1512.268672 (Hartree)

4I

Atom	X	Y	Z (Angstrom)
6	-2.341904	0.410888	-0.028478
6	-0.424454	1.446334	-0.579331
7	-1.058850	0.846863	0.469696
7	-1.083210	1.430454	-1.678846
6	0.888030	2.114286	-0.510637
6	1.279687	2.823875	0.627111

6	1.731949	2.065917	-1.624353
6	2.507443	3.479864	0.648342
1	0.630399	2.872780	1.490841
6	2.954770	2.726904	-1.599426
1	1.433606	1.496077	-2.493890
6	3.345377	3.434874	-0.463477
1	2.808560	4.021619	1.534619
1	3.608901	2.675400	-2.459170
1	4.302991	3.937263	-0.440711
8	-2.278066	0.719446	-1.404409
6	-3.458209	1.291318	0.590115
9	-3.239015	2.580320	0.335151
9	-3.501393	1.144472	1.918909
9	-4.660104	0.980873	0.104315
6	-2.697795	-1.053553	0.181642
1	-2.864126	-1.288901	1.229106
1	-3.587401	-1.293458	-0.393563
53	-1.148170	-2.333000	-0.520076
6	-0.517105	0.470797	1.764278
1	0.559760	0.331216	1.687617
1	-0.946210	-0.483320	2.067740
1	-0.742090	1.217648	2.526913
53	3.071975	-1.370706	0.502457
3	3.016934	1.164440	0.207861

Energy -1512.346490 (Hartree)

TS Int-F→2I

Atom	X	Y	Z (Angstrom)
8	0.685224	-1.091731	-0.550357
6	1.421625	-1.002289	0.679985
7	1.253440	0.330350	1.162600
6	0.481154	0.922421	0.308151
7	0.043545	0.196390	-0.757377
6	0.748645	-1.993863	1.654188
9	0.803090	-3.247477	1.192969
6	2.871521	-1.419820	0.473407
6	0.040569	2.323639	0.442348
6	0.351968	3.031888	1.601478
6	-0.061405	4.347070	1.741141
6	-0.787202	4.960880	0.726606
6	-1.098530	4.257341	-0.429955
6	-0.686082	2.940874	-0.574504
9	-0.536701	-1.688926	1.826057
9	1.338552	-1.975414	2.850053
1	0.913801	2.542572	2.386296
1	-0.922433	2.395793	-1.479433
1	0.180607	4.894382	2.643105
1	-1.662358	4.734238	-1.221224
1	-1.110518	5.988185	0.838004
1	3.449492	-1.260139	1.378751
1	2.958001	-2.447656	0.133318
6	-2.017501	-0.217718	-0.636025
1	-1.740410	-1.253468	-0.726658
1	-2.083515	0.239107	0.336195
1	-2.189633	0.374262	-1.516912
53	-4.497995	-0.861197	-0.530989
53	3.779803	-0.214597	-1.039152
3	1.225799	-0.279908	-2.243432

Energy -1512.270339 (Hartree)

2I

Atom	X	Y	Z (Angstrom)
8	0.787827	-1.206217	-0.388918

6	1.730814	-1.062364	0.668874
7	1.659554	0.300309	1.119389
6	0.742111	0.860270	0.427308
7	0.159526	0.061149	-0.547088
6	1.273686	-1.989769	1.817677
9	1.244289	-3.267634	1.429867
6	3.117413	-1.491323	0.214600
6	0.342005	2.269352	0.566406
6	0.485350	2.893428	1.804117
6	0.137370	4.226849	1.947966
6	-0.345561	4.941691	0.857827
6	-0.480673	4.322403	-0.378231
6	-0.140249	2.986246	-0.527257
9	0.045177	-1.670270	2.226515
9	2.086627	-1.902545	2.871815
1	0.862759	2.325910	2.645002
1	-0.230252	2.504445	-1.492759
1	0.242478	4.709705	2.910897
1	-0.848163	4.880949	-1.229261
1	-0.614960	5.984155	0.971717
1	3.832852	-1.388673	1.025189
1	3.107839	-2.511023	-0.159427
6	-1.291242	-0.120258	-0.562916
1	-1.550901	-0.719911	-1.431899
1	-1.636933	-0.612334	0.348884
1	-1.758392	0.856823	-0.656535
53	-5.078058	-0.644661	-0.649268
53	3.826550	-0.272095	-1.381831
3	-5.974536	-1.925848	1.349892

Energy -1512.329889 (Hartree)

Methyl Iodide

Atom	X	Y	Z (Angstrom)
6	1.807446	0.000000	0.000004
1	2.130162	0.173891	1.020203
1	2.129833	0.796650	-0.660780
1	2.129788	-0.970580	-0.359585
53	-0.325178	0.000001	0.000003

Energy -337.532450 (Hartree)

Int-Br

Atom	X	Y	Z (Angstrom)
6	0.136288	-1.670735	-0.513929
6	1.783810	-0.286142	-0.305669
7	0.476615	-0.290184	-0.400870
7	2.413256	-1.452082	-0.340051
6	2.549929	0.970194	-0.143504
6	1.892347	2.115022	0.302648
6	3.911930	1.029814	-0.431912
6	2.592050	3.301503	0.467920
1	0.832704	2.061975	0.522760
6	4.610047	2.217499	-0.264974
1	4.422664	0.153885	-0.813436
6	3.952021	3.354709	0.186914
1	2.075296	4.186293	0.817798
1	5.667134	2.257347	-0.495603
1	4.496964	4.281518	0.315219
8	1.353855	-2.411466	-0.505067
6	-0.522230	-1.923894	-1.888284
9	0.276653	-1.542804	-2.885762
9	-1.671700	-1.248487	-2.015880
9	-0.801935	-3.220905	-2.083079
6	-0.826835	-2.176220	0.561290

1	-1.742013	-1.589601	0.557179
1	-1.048991	-3.232599	0.436171
3	3.920760	-2.310855	0.363932
35	-0.042489	-1.979319	2.326129
6	-1.581979	2.191515	-1.486529
1	-2.240001	2.026995	-2.332765
1	-1.278671	3.230357	-1.415131
1	-0.734092	1.513738	-1.487867
53	-2.698066	1.739181	0.275000

Energy -3788.857295 (Hartree)

TS IntBr→2Br

Atom	X	Y	Z (Angstrom)
6	1.801573	-1.024569	0.456278
6	0.827702	0.896578	0.174079
7	1.655566	0.297178	0.968871
7	0.317591	0.178124	-0.864967
6	0.396792	2.295428	0.350897
6	0.753686	2.979600	1.511440
6	-0.364384	2.933912	-0.626700
6	0.350319	4.292822	1.691631
1	1.341903	2.473185	2.265508
6	-0.765098	4.248947	-0.442108
1	-0.634870	2.407273	-1.533034
6	-0.409107	4.928553	0.715909
1	0.626339	4.821573	2.594896
1	-1.354529	4.743507	-1.203275
1	-0.723742	5.954660	0.858720
8	0.960169	-1.117556	-0.701945
6	1.256632	-2.049091	1.473667
9	-0.009430	-1.776901	1.779492
9	1.967169	-2.034953	2.601325
9	1.293470	-3.291570	0.982986
6	3.237472	-1.392477	0.095127
1	3.904417	-1.217707	0.934455
1	3.323421	-2.414152	-0.264698
3	1.363808	-0.338859	-2.430679
35	3.850630	-0.239024	-1.355763
6	-1.728095	-0.220672	-0.614899
1	-1.480332	-1.253496	-0.787677
1	-1.958668	0.425180	-1.443120
1	-1.716419	0.175148	0.386041
53	-4.209250	-0.840053	-0.361547

Energy -3788.833286 (Hartree)

2Br

Atom	X	Y	Z (Angstrom)
6	2.134659	-0.678056	0.033032
6	0.890278	1.116721	0.084003
7	1.871499	0.605396	-0.540788
7	0.352407	0.303541	1.115577
6	0.273295	2.413706	-0.207929
6	1.045481	3.409238	-0.805300
6	-1.075330	2.634784	0.069334
6	0.468595	4.629616	-1.112775
1	2.090521	3.220944	-1.015165
6	-1.646769	3.856528	-0.251339
1	-1.679974	1.849558	0.508714
6	-0.876083	4.852986	-0.836505
1	1.066455	5.408130	-1.568290
1	-2.695620	4.027596	-0.047510
1	-1.324975	5.807423	-1.080958
8	1.183645	-0.867751	1.090173

6	3.538978	-0.684272	0.673544
9	3.648668	0.295611	1.565984
9	4.483744	-0.516052	-0.247550
9	3.769527	-1.835991	1.302479
6	2.031079	-1.816749	-0.973485
1	2.690236	-1.638534	-1.818210
1	2.244427	-2.777182	-0.512374
3	-0.912433	-1.223695	0.635585
35	0.215652	-1.912607	-1.670121
6	0.361494	0.864687	2.475034
53	-3.457867	-0.995994	0.423809
1	1.370333	1.160522	2.764903
1	-0.304799	1.723760	2.481926
1	-0.028189	0.109961	3.153742

Energy -3788.917330 (Hartree)

TS Int-Br→4Br

Atom	X	Y	Z (Angstrom)
6	-1.587613	-1.067844	0.479293
6	-1.182495	1.077049	0.577148
7	-0.665627	-0.039313	0.090206
7	-2.319555	0.973433	1.224736
6	-0.574528	2.408768	0.376685
6	-0.006401	2.728327	-0.854800
6	-0.589764	3.355152	1.399121
6	0.540845	3.986044	-1.060461
1	-0.006887	1.991271	-1.648765
6	-0.039543	4.612086	1.189352
1	-1.007623	3.097999	2.365177
6	0.525292	4.928705	-0.039395
1	0.979226	4.230957	-2.019403
1	-0.043949	5.341064	1.989659
1	0.956974	5.908389	-0.200621
8	-2.628879	-0.420826	1.203351
6	-0.936545	-2.052849	1.479768
9	-0.384603	-1.400461	2.503464
9	0.021199	-2.790717	0.907229
9	-1.838879	-2.898578	1.988207
6	-2.143290	-1.882049	-0.688225
1	-1.332517	-2.339008	-1.249522
1	-2.842300	-2.641708	-0.348157
3	-3.717239	2.121914	1.771961
35	-3.110871	-0.737219	-1.919089
6	1.424058	-0.374597	-0.120692
53	3.930108	-0.768379	-0.387302
1	1.518310	0.633023	-0.480928
1	1.486378	-0.571888	0.934166
1	1.196627	-1.166298	-0.810654

Energy -3788.828728 (Hartree)

4Br

Atom	X	Y	Z (Angstrom)
6	-2.432936	-0.044552	-0.306181
6	-0.409668	0.937320	-0.176153
7	-1.626063	0.917460	0.408065
7	-0.319685	0.161720	-1.203073
6	0.759620	1.693780	0.299055
6	1.180579	1.568136	1.621462
6	1.499831	2.445353	-0.609285
6	2.346310	2.196482	2.029792
1	0.616921	0.960445	2.318995
6	2.661165	3.077642	-0.189934
1	1.164500	2.533654	-1.635060

6	3.085159	2.951050	1.126007
1	2.683495	2.088604	3.052367
1	3.237250	3.663968	-0.893775
1	3.996208	3.438422	1.449006
8	-1.591718	-0.446521	-1.365522
6	-3.663845	0.629030	-0.966883
9	-3.306275	1.736781	-1.608849
9	-4.594837	0.956539	-0.066956
9	-4.226141	-0.194830	-1.849067
6	-2.904934	-1.231124	0.529328
1	-3.549329	-0.907947	1.342981
1	-3.432170	-1.944376	-0.099188
3	1.285274	-0.664589	-1.959045
35	-1.389381	-2.152888	1.309148
6	-2.093061	1.678365	1.552367
53	3.433906	-1.132232	-0.608544
1	-1.314305	2.376605	1.844703
1	-2.984346	2.248181	1.292210
1	-2.312766	1.018173	2.393203

Energy -3788.923419 (Hartree)

Int-Br-EXPSOLV

Atom	X	Y	Z (Angstrom)
6	-1.242589	-1.344877	-0.698794
6	-0.387199	0.625155	-0.916922
7	-1.556885	0.044584	-0.736432
7	0.683449	-0.137356	-1.024705
6	-0.248939	2.098221	-0.971737
6	-1.223844	2.897363	-0.377329
6	0.840744	2.698232	-1.600407
6	-1.104735	4.279958	-0.399952
1	-2.068769	2.422863	0.107307
6	0.954073	4.081259	-1.627837
1	1.593025	2.080004	-2.075466
6	-0.015092	4.874714	-1.024398
1	-1.863658	4.893641	0.068998
1	1.797503	4.542158	-2.126793
1	0.076044	5.953538	-1.046902
8	0.159447	-1.472568	-0.920893
6	-1.941610	-2.065883	-1.872593
9	-1.594552	-1.535949	-3.046319
9	-3.276180	-1.985668	-1.777940
9	-1.629734	-3.370505	-1.925896
6	-1.659680	-2.059321	0.589834
1	-2.730691	-1.964360	0.750810
1	-1.364096	-3.105413	0.582668
3	2.548743	0.043397	-0.464397
6	4.022827	2.682146	-0.015832
8	3.074861	1.677671	0.331349
6	2.449224	2.115405	1.538607
6	3.547499	2.839208	2.335016
6	4.686101	3.031884	1.310370
1	4.694807	2.271121	-0.767082
1	3.494036	3.545180	-0.435028
1	1.628232	2.793622	1.283463
1	2.041764	1.235595	2.037295
1	3.179248	3.790514	2.714787
1	3.878917	2.243822	3.184567
1	5.089171	4.042913	1.311234
1	5.502553	2.336986	1.508861
6	2.592850	-2.547953	1.119004
8	3.132190	-1.244941	0.853240
6	4.090294	-0.977896	1.875587

6	3.422216	-1.493404	3.138046
6	2.711959	-2.762148	2.638376
1	1.570217	-2.552738	0.748800
1	3.182097	-3.290004	0.571874
1	5.014534	-1.530063	1.663005
1	4.301228	0.090361	1.874781
1	4.134683	-1.690544	3.937259
1	2.697275	-0.757828	3.490459
1	3.307659	-3.649306	2.849752
1	1.735817	-2.889502	3.101858
6	3.363411	-1.023985	-2.955542
8	3.777427	-0.084543	-1.959999
6	5.064720	-0.527058	-1.515951
6	5.036235	-2.063358	-1.590210
6	3.735635	-2.372410	-2.357813
1	2.297334	-0.883253	-3.124262
1	3.914235	-0.829398	-3.882016
1	5.826728	-0.110947	-2.180885
1	5.223019	-0.137846	-0.509815
1	5.912156	-2.435352	-2.119474
1	5.025768	-2.508144	-0.596087
1	3.862055	-3.141876	-3.117355
1	2.949516	-2.690328	-1.671151
35	-0.794002	-1.250621	2.130224
6	-4.754458	1.051284	-1.271898
1	-5.401085	0.482543	-1.931142
1	-4.994141	2.108752	-1.295856
1	-3.702148	0.861120	-1.461510
53	-5.128910	0.369209	0.716083

Energy -4486.247909 (Hartree)

TS Int-Br→2Br-EXPSOLV

Atom	X	Y	Z (Angstrom)
6	-0.211396	2.442086	-0.982119
6	0.032293	1.465059	0.933449
7	-0.217549	2.637912	0.432816
7	0.221061	0.442053	0.067616
6	0.126704	1.223238	2.389197
6	-0.252624	2.233708	3.272205
6	0.587377	0.009307	2.895172
6	-0.164687	2.034011	4.641377
1	-0.614705	3.172572	2.874103
6	0.685416	-0.184216	4.266332
1	0.869261	-0.790760	2.224281
6	0.310540	0.826755	5.141489
1	-0.463154	2.823050	5.319817
1	1.053361	-1.127624	4.649786
1	0.385815	0.674253	6.210840
8	0.027746	1.057767	-1.213566
6	0.973487	3.216577	-1.604192
9	2.130766	2.845520	-1.056593
9	0.848844	4.535630	-1.429855
9	1.070004	2.994216	-2.921292
6	-1.486028	2.894017	-1.690960
1	-1.653708	3.956185	-1.535717
1	-1.445734	2.668641	-2.753546
3	-0.774964	-1.324214	-0.003547
6	-1.678544	-2.548577	2.666297
8	-1.806948	-1.622227	1.573592
6	-2.772690	-0.605939	1.888247
6	-2.889883	-0.624850	3.400691
6	-2.701314	-2.110609	3.710171
1	-1.860558	-3.557482	2.293997

1	-0.656034	-2.490322	3.044123
1	-2.413178	0.339873	1.483373
1	-3.724962	-0.856767	1.408773
1	-2.089459	-0.031520	3.845456
1	-3.847926	-0.236760	3.742197
1	-2.348824	-2.295753	4.723519
1	-3.640957	-2.646107	3.566236
6	-2.109593	-1.087989	-2.718330
8	-2.107147	-1.594599	-1.376178
6	-3.308358	-2.347505	-1.207599
6	-4.358792	-1.516033	-1.921342
6	-3.586461	-0.985805	-3.139475
1	-1.588503	-0.133165	-2.702063
1	-1.562609	-1.785321	-3.359921
1	-3.195744	-3.329442	-1.682980
1	-3.476527	-2.482830	-0.140890
1	-5.235894	-2.099597	-2.195676
1	-4.673605	-0.695137	-1.275878
1	-3.772966	-1.605597	-4.015627
1	-3.864985	0.038040	-3.381291
6	1.155689	-2.602992	-1.613870
8	0.434105	-2.783527	-0.375660
6	-0.302031	-4.020902	-0.416486
6	-0.331381	-4.430708	-1.878110
6	1.018474	-3.915314	-2.370630
1	0.701478	-1.770377	-2.161424
1	2.190654	-2.353326	-1.381862
1	0.221652	-4.758178	0.197384
1	-1.292986	-3.846232	0.005416
1	-0.457250	-5.504898	-2.001962
1	-1.142875	-3.917155	-2.396746
1	1.815334	-4.601674	-2.079786
1	1.059766	-3.769245	-3.448490
35	-3.033180	1.966153	-0.979115
6	2.312190	-0.031050	0.028403
1	2.216439	0.142623	-1.028801
1	2.105551	-1.002964	0.438276
1	2.546307	0.786011	0.689459
53	4.777363	-0.625409	-0.240247

Energy -4486.225321 (Hartree)

2Br-EXPSOLV

Atom	X	Y	Z (Angstrom)
6	-2.742003	1.776503	-0.595379
6	-0.813183	1.907939	0.414015
7	-2.000700	2.354477	0.483620
7	-0.580976	0.991298	-0.652117
6	0.263780	2.249067	1.350447
6	0.076936	3.333718	2.211672
6	1.438070	1.503304	1.416192
6	1.057991	3.662678	3.128424
1	-0.836658	3.910096	2.149499
6	2.417576	1.836438	2.341481
1	1.605559	0.660220	0.755121
6	2.228934	2.912248	3.196047
1	0.913991	4.505791	3.791430
1	3.329633	1.255040	2.382297
1	2.996628	3.172668	3.913606
8	-1.859390	0.901425	-1.288514
6	-3.172573	2.892453	-1.573710
9	-2.119989	3.582705	-2.005023
9	-4.012311	3.747273	-0.990817
9	-3.784417	2.379044	-2.643012

6	-3.985058	1.023194	-0.139255
1	-4.655845	1.688536	0.396913
1	-4.496828	0.571338	-0.984886
3	-0.221190	-1.070101	-0.120887
6	0.624509	-1.455752	2.775326
8	-0.307933	-1.657390	1.701253
6	-1.224570	-2.657022	2.153876
6	-0.333780	-3.685500	2.828235
6	0.742289	-2.809966	3.496140
1	1.562464	-1.111028	2.345972
1	0.230488	-0.680007	3.436678
1	-1.918088	-2.209390	2.875016
1	-1.782647	-3.025047	1.294269
1	-0.881131	-4.302209	3.538557
1	0.110840	-4.336257	2.073514
1	0.549423	-2.688625	4.560612
1	1.736330	-3.238236	3.379814
6	-2.430508	-2.355089	-1.905178
8	-1.205190	-2.300508	-1.173780
6	-0.712059	-3.645076	-1.108216
6	-1.954841	-4.551001	-1.074658
6	-3.126182	-3.590349	-1.358094
1	-2.963549	-1.422107	-1.742345
1	-2.203471	-2.462311	-2.971497
1	-0.100779	-3.840389	-1.993603
1	-0.083147	-3.726561	-0.221201
1	-1.878889	-5.324403	-1.836924
1	-2.065672	-5.041086	-0.108468
1	-3.849915	-4.000624	-2.059831
1	-3.646667	-3.334988	-0.434197
6	1.930385	-1.563186	-2.083159
8	1.571674	-1.521769	-0.684205
6	2.427066	-2.404646	0.062969
6	2.943585	-3.408775	-0.950436
6	3.093322	-2.543297	-2.200054
1	1.049622	-1.890824	-2.640727
1	2.211398	-0.560955	-2.402895
1	3.242574	-1.821582	0.501058
1	1.834159	-2.858696	0.856973
1	3.879893	-3.863979	-0.632196
1	2.205388	-4.196762	-1.109148
1	4.038805	-2.000499	-2.167903
1	3.048868	-3.114395	-3.125727
35	-3.492089	-0.408000	1.067756
6	0.387451	1.456433	-1.666644
53	4.617698	1.030899	-0.866256
1	0.160842	2.473984	-1.986402
1	1.391336	1.408569	-1.248598
1	0.319800	0.775809	-2.511143

Energy -4486.291270 (Hartree)

TS Int-Br→4Br-EXPSOLV

Atom	X	Y	Z (Angstrom)
6	1.149709	-1.825526	0.409721
6	0.618320	0.288414	0.399430
7	1.590376	-0.524074	-0.007126
7	-0.383433	-0.243860	1.053269
6	0.572013	1.729843	0.075621
6	1.087844	2.185677	-1.135593
6	-0.032685	2.629713	0.952265
6	0.999070	3.530305	-1.467470
1	1.542200	1.478368	-1.818923
6	-0.117915	3.973579	0.618033

1	-0.428681	2.271546	1.893773
6	0.395545	4.425355	-0.592363
1	1.398149	3.878982	-2.411526
1	-0.581710	4.670387	1.305109
1	0.329758	5.474767	-0.851087
8	-0.042299	-1.630074	1.160253
6	2.150565	-2.483122	1.382725
9	2.424540	-1.682412	2.412487
9	3.309494	-2.778908	0.780989
9	1.665302	-3.623761	1.887075
6	0.915093	-2.796156	-0.751438
1	1.808396	-2.885821	-1.364087
1	0.593479	-3.774050	-0.401522
3	-2.238267	0.088002	0.403646
6	-2.423394	2.045745	-1.844075
8	-2.606260	0.711318	-1.365128
6	-3.357675	0.037482	-2.370086
6	-4.436059	1.042479	-2.754978
6	-3.722604	2.401105	-2.585701
1	-2.202800	2.682473	-0.987866
1	-1.567576	2.057614	-2.524557
1	-2.704445	-0.184094	-3.222009
1	-3.730683	-0.896277	-1.951934
1	-4.805751	0.880132	-3.765740
1	-5.276666	0.961401	-2.064263
1	-3.498935	2.862653	-3.545918
1	-4.331346	3.098007	-2.011695
6	-3.199399	-2.805824	0.736682
8	-3.389890	-1.391870	0.701021
6	-4.794555	-1.154689	0.571478
6	-5.371045	-2.378576	-0.164997
6	-4.167229	-3.327333	-0.311348
1	-2.150074	-3.003878	0.530984
1	-3.454447	-3.177845	1.735407
1	-5.231511	-1.046924	1.568206
1	-4.922889	-0.217854	0.027665
1	-6.162617	-2.836432	0.426129
1	-5.789736	-2.104927	-1.132428
1	-4.430290	-4.371481	-0.151622
1	-3.716262	-3.231320	-1.300121
6	-3.138058	1.193638	2.876580
8	-3.076916	1.444459	1.461824
6	-4.077179	2.398352	1.079691
6	-5.032160	2.468731	2.259585
6	-4.080031	2.249374	3.434452
1	-3.527513	0.183307	3.031178
1	-2.126967	1.247715	3.280882
1	-3.594722	3.364607	0.900956
1	-4.542570	2.057767	0.153641
1	-5.566294	3.416153	2.302969
1	-5.759383	1.655824	2.204632
1	-3.532723	3.168527	3.651938
1	-4.578836	1.915928	4.342504
35	-0.498469	-2.123912	-1.904478
6	3.648568	0.090599	-0.037649
53	6.079502	0.826021	-0.107348
1	3.308587	1.065718	-0.334361
1	3.723172	-0.148542	1.007277
1	3.819155	-0.663016	-0.783500

Energy -4486.220575 (Hartree)

4Br-EXPSOLV

Atom	X	Y	Z (Angstrom)
6	2.955865	-1.100657	-0.847496
6	1.286962	-1.275913	0.652100
7	2.401428	-1.915269	0.206890
7	1.099844	-0.126845	0.108498
6	0.373306	-1.787401	1.686665
6	0.871147	-2.249424	2.904158
6	-0.998636	-1.753278	1.453440
6	-0.009335	-2.681901	3.883173
1	1.938506	-2.251177	3.089583
6	-1.873161	-2.193669	2.436938
1	-1.382734	-1.396111	0.503471
6	-1.379880	-2.657875	3.648872
1	0.374361	-3.034938	4.831656
1	-2.939081	-2.172659	2.246178
1	-2.063813	-3.001790	4.414437
8	2.162637	0.063472	-0.812716
6	2.727970	-1.746454	-2.242419
9	1.460589	-2.132052	-2.375022
9	3.504440	-2.815969	-2.435614
9	2.993863	-0.872576	-3.212097
6	4.435710	-0.769615	-0.698159
1	5.044803	-1.669101	-0.734925
1	4.746973	-0.083100	-1.481337
3	-0.269623	1.339724	0.152096
6	-2.621916	1.259228	1.907368
8	-1.303955	1.776928	1.682422
6	-1.234810	3.015921	2.386988
6	-2.575025	3.680077	2.104193
6	-3.544522	2.484990	1.999794
1	-2.860742	0.583479	1.086728
1	-2.618647	0.695017	2.843860
1	-1.108390	2.813394	3.455946
1	-0.369517	3.567195	2.020513
1	-2.853881	4.385607	2.884873
1	-2.527283	4.217827	1.156766
1	-4.187453	2.410848	2.875071
1	-4.181859	2.567657	1.121022
6	2.028772	3.137881	0.313369
8	0.745210	2.872491	-0.280014
6	0.198146	4.072032	-0.850442
6	1.347284	5.067647	-0.868129
6	2.132917	4.653053	0.375333
1	2.065134	2.650899	1.288809
1	2.801404	2.705290	-0.325654
1	-0.194511	3.833407	-1.839285
1	-0.621363	4.422406	-0.214952
1	1.955152	4.927473	-1.763726
1	0.998146	6.098028	-0.838329
1	3.166631	4.993812	0.369260
1	1.643458	5.032437	1.274734
6	-1.272355	0.177492	-2.324666
8	-1.559135	0.973558	-1.159024
6	-2.648905	1.868906	-1.423814
6	-2.681107	1.999966	-2.935566
6	-2.308544	0.581140	-3.367983
1	-0.253760	0.408344	-2.647436
1	-1.334136	-0.876826	-2.050299

1	-3.574391	1.424158	-1.047163
1	-2.454690	2.803617	-0.894038
1	-3.656057	2.317546	-3.301238
1	-1.926739	2.715243	-3.270097
1	-3.181029	-0.070019	-3.299149
1	-1.909145	0.528516	-4.379178
35	4.772349	0.095292	1.000990
6	2.734442	-3.320083	0.371002
53	-4.479260	-1.687775	-0.622153
1	2.371673	-3.663625	1.334512
1	2.289729	-3.934059	-0.414718
1	3.817730	-3.434930	0.350514

Energy -4486.299618 (Hartree)

References

1. Buscemi, S.; Pace, A.; Pibiri, I.; Vivona, N.; Spinelli, D., Fluorinated heterocyclic compounds. An expedient route to 5-perfluoroalkyl-1, 2, 4-triazoles via an unusual hydrazinolysis of 5-perfluoroalkyl-1, 2, 4-oxadiazoles: First examples of an ANRORC-like reaction in 1, 2, 4-oxadiazole derivatives. *The Journal of Organic Chemistry* **2003,** *68* (2), 605-608.
2. Frisch, M. J.; Trucks, G. W.; Schlegel, H. B.; Scuseria, G. E.; Robb, M. A.; Cheeseman, J. R.; Scalmani, G.; Barone, V.; Petersson, G. A.; Nakatsuji, H.; Li, X.; Caricato, M.; Marenich, A. V.; Bloino, J.; Janesko, B. G.; Gomperts, R.; Mennucci, B.; Hratchian, H. P.; Ortiz, J. V.; Izmaylov, A. F.; Sonnenberg, J. L.; Williams; Ding, F.; Lipparini, F.; Egidi, F.; Goings, J.; Peng, B.; Petrone, A.; Henderson, T.; Ranasinghe, D.; Zakrzewski, V. G.; Gao, J.; Rega, N.; Zheng, G.; Liang, W.; Hada, M.; Ehara, M.; Toyota, K.; Fukuda, R.; Hasegawa, J.; Ishida, M.; Nakajima, T.; Honda, Y.; Kitao, O.; Nakai, H.; Vreven, T.; Throssell, K.; Montgomery Jr., J. A.; Peralta, J. E.; Ogliaro, F.; Bearpark, M. J.; Heyd, J. J.; Brothers, E. N.; Kudin, K. N.; Staroverov, V. N.; Keith, T. A.; Kobayashi, R.; Normand, J.; Raghavachari, K.; Rendell, A. P.; Burant, J. C.; Iyengar, S. S.; Tomasi, J.; Cossi, M.; Millam, J. M.; Klene, M.; Adamo, C.; Cammi, R.; Ochterski, J. W.; Martin, R. L.; Morokuma, K.; Farkas, O.; Foresman, J. B.; Fox, D. J. *Gaussian 16 Rev. B.01*, Wallingford, CT, 2016.
3. Zhao, Y.; Truhlar, D. G., The M06 suite of density functionals for main group thermochemistry, thermochemical kinetics, noncovalent interactions, excited states, and transition elements: two new functionals and systematic testing of four M06-class functionals and 12 other functionals. *Theor Chem Acc* **2008,** *120* (1-3), 215-241.
4. Weigend, F., Accurate Coulomb-fitting basis sets for H to Rn. *Physical chemistry chemical physics* **2006,** *8* (9), 1057-1065.
5. Weigend, F.; Ahlrichs, R., Balanced basis sets of split valence, triple zeta valence and quadruple zeta valence quality for H to Rn: Design and assessment of accuracy. *Physical Chemistry Chemical Physics* **2005,** *7* (18), 3297-3305.
6. Marenich, A. V.; Cramer, C. J.; Truhlar, D. G., Universal Solvation Model Based on Solute Electron Density and on a Continuum Model of the Solvent Defined by the Bulk Dielectric Constant and Atomic Surface Tensions. *The Journal of Physical Chemistry B* **2009,** *113* (18), 6378-6396.
7. Riplinger, C.; Neese, F., An efficient and near linear scaling pair natural orbital based local coupled cluster method. *The Journal of Chemical Physics* **2013,** *138* (3).
8. Neese, F., Software update: The ORCA program system—Version 5.0. *WIREs Computational Molecular Science* **2022,** *12* (5).
9. Pracht, P.; Grimme, S.; Bannwarth, C.; Bohle, F.; Ehlert, S.; Feldmann, G.; Gorges, J.; Müller, M.; Neudecker, T.; Plett, C.; Spicher, S.; Steinbach, P.; Wesolowski, P. A.; Zeller, F., CREST-A program for the exploration of low-energy molecular chemical space. *J Chem Phys* **2024,** *160* (11).
10. Lu, T.; Chen, F. W., Multiwfn: A multifunctional wavefunction analyzer. *J Comput Chem* **2012,** *33* (5), 580-592.
11. Scalfani, V. F.; Williams, A. J.; Tkachenko, V.; Karapetyan, K.; Pshenichnov, A.; Hanson, R. M.; Liddie, J. M.; Bara, J. E., Programmatic conversion of crystal structures into 3D printable files using Jmol. *Journal of Cheminformatics* **2016,** *8* (1).
12. Parr, R. G.; Yang, W., Density functional approach to the frontier-electron theory of chemical reactivity. *Journal of the American Chemical Society* **2002,** *106* (14), 4049-4050.
13. Liu, S., Steric effect: A quantitative description from density functional theory. *The Journal of Chemical Physics* **2007,** *126* (24).
14. Weizs[illegible]cker, C. F. v., Zur Theorie der Kernmassen. *Zeitschrift f[illegible]r Physik* **1935,** *96* (7-8), 431-458.

---

[i] Version 1.37.0.0 (STOE, 2021)

[ii] Version 1.31.186.0 (STOE, 2022)

[iii] Dolomanov, O.V., Bourhis, L.J., Gildea, R.J, Howard, J.A.K. & Puschmann, H. , OLEX2, (2009), J. Appl. Cryst. 42, 339-341

[iv] C. B. Huebschle, G. M. Sheldrick and B. Dittrich, ShelXle: a Qt graphical user interface for SHELXL, J. Appl. Cryst., 44, (2011) 1281-1284

[v] Sheldrick, G. M. (2015). *SHELXS v 2016/4* University of Göttingen, Germany.

[vi] Sheldrick, G. M. (2015). *SHELXL v 2016/4* University of Göttingen, Germany.

[vii] A. L. Spek, Acta Cryst. 2009, D65, 148-155
